# Chiral 3-acyl-2-pyrrolines as strategic hubs toward polycyclic pyrrolidines *via* enantioselective silver-catalyzed Michael addition–cyclization

**DOI:** 10.1039/d6sc04627d

**Published:** 2026-07-16

**Authors:** Satoshi Sakai, Kakeru Maeda, Yohei Shimizu, Masaya Sawamura

**Affiliations:** a Department of Chemistry, Faculty of Science, Hokkaido University Kita 10 Nishi 8, Kita-ku Sapporo Hokkaido 060-0810 Japan shimizu-y@sci.hokudai.ac.jp; b Institute for Chemical Reaction Design and Discovery (WPI-ICReDD), Hokkaido University Kita 21 Nishi 10, Kita-ku Sapporo Hokkaido 001-0021 Japan; c List Sustainable Digital Transformation Catalyst Collaboration Research Platform, Institute for Chemical Reaction Design and Discovery (ICReDD List-PF), Hokkaido University Kita 21 Nishi 10, Kita-ku Sapporo Hokkaido 001-0021 Japan

## Abstract

We present a synthetic strategy for the construction of chiral polycyclic pyrrolidines through enantioenriched 3-acyl-2-pyrrolines as multifunctional synthetic hub molecules. These intermediates were produced by a silver-catalyzed enantioselective Michael addition–cyclization between α-substituted isocyanoacetates and nonactivated β-substituted alkyl enones, enabled by a chiral prolinol-phosphine-*sec*-amine ligand. This catalysis overcame the inherent challenges associated with low electrophilicity of the alkyl enones and steric congestion in C–C bond formation, furnishing 3-acyl-2-pyrrolines bearing consecutive stereogenic carbon centers including a sterically congested tetrasubstituted center with high stereoselectivities. Notably, the reaction proceeded in a stereoconvergent manner, providing the same major stereoisomer regardless of the *E*/*Z* configuration of the enone. The resulting chiral pyrrolines served as useful intermediates for the construction of structurally complex polycyclic frameworks through intermolecular and intramolecular Diels–Alder reactions. This strategy enables efficient access to polycyclic pyrrolidines from readily available starting materials, contributing to diversity-oriented synthesis (DOS) of drug-like molecules.

## Abstract 2

We present a synthetic strategy for the construction of chiral polycyclic pyrrolidines through enantioenriched 3-acyl-2-pyrrolines as multifunctional synthetic hub molecules.

## Graphical abstract

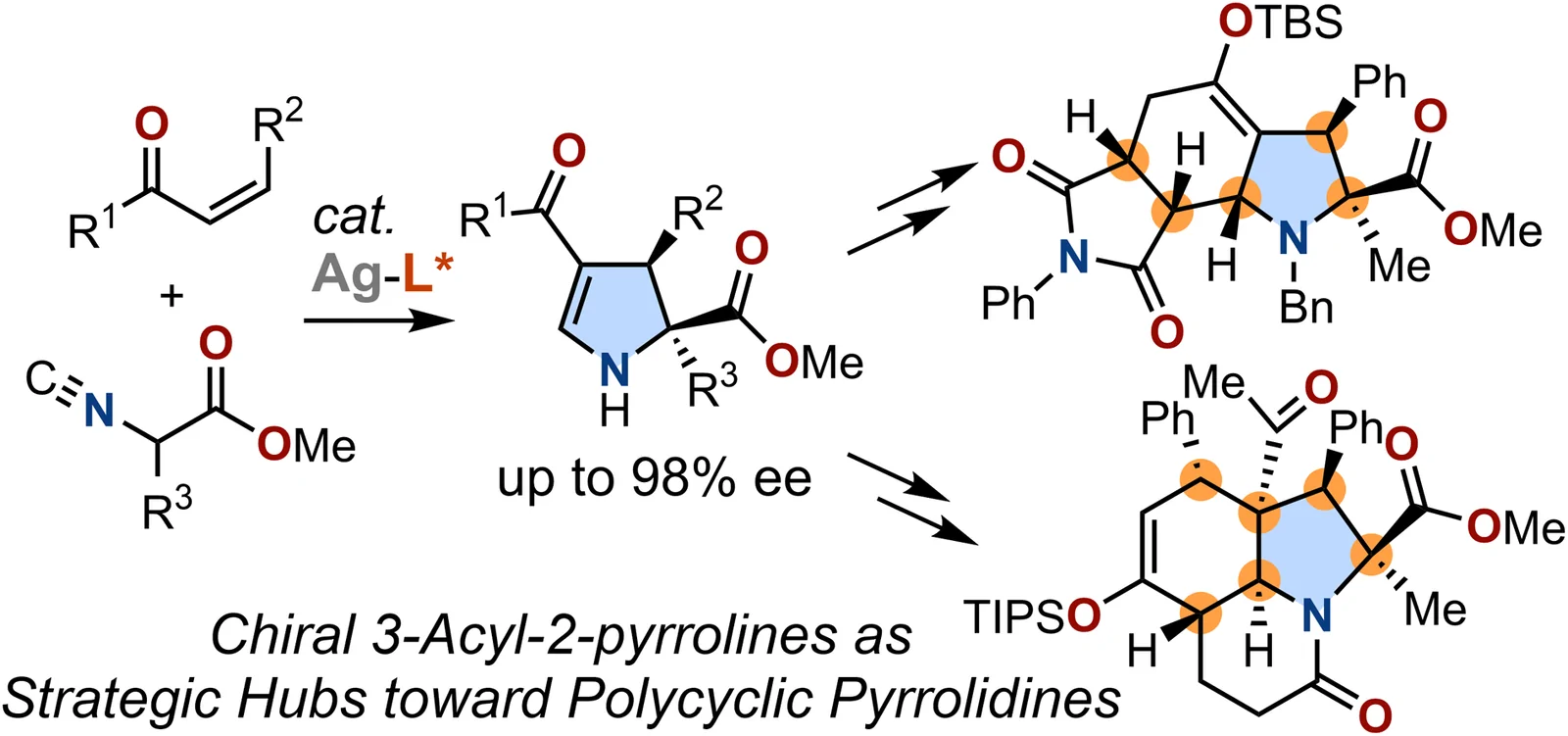

## Introduction

**Scheme 1:**
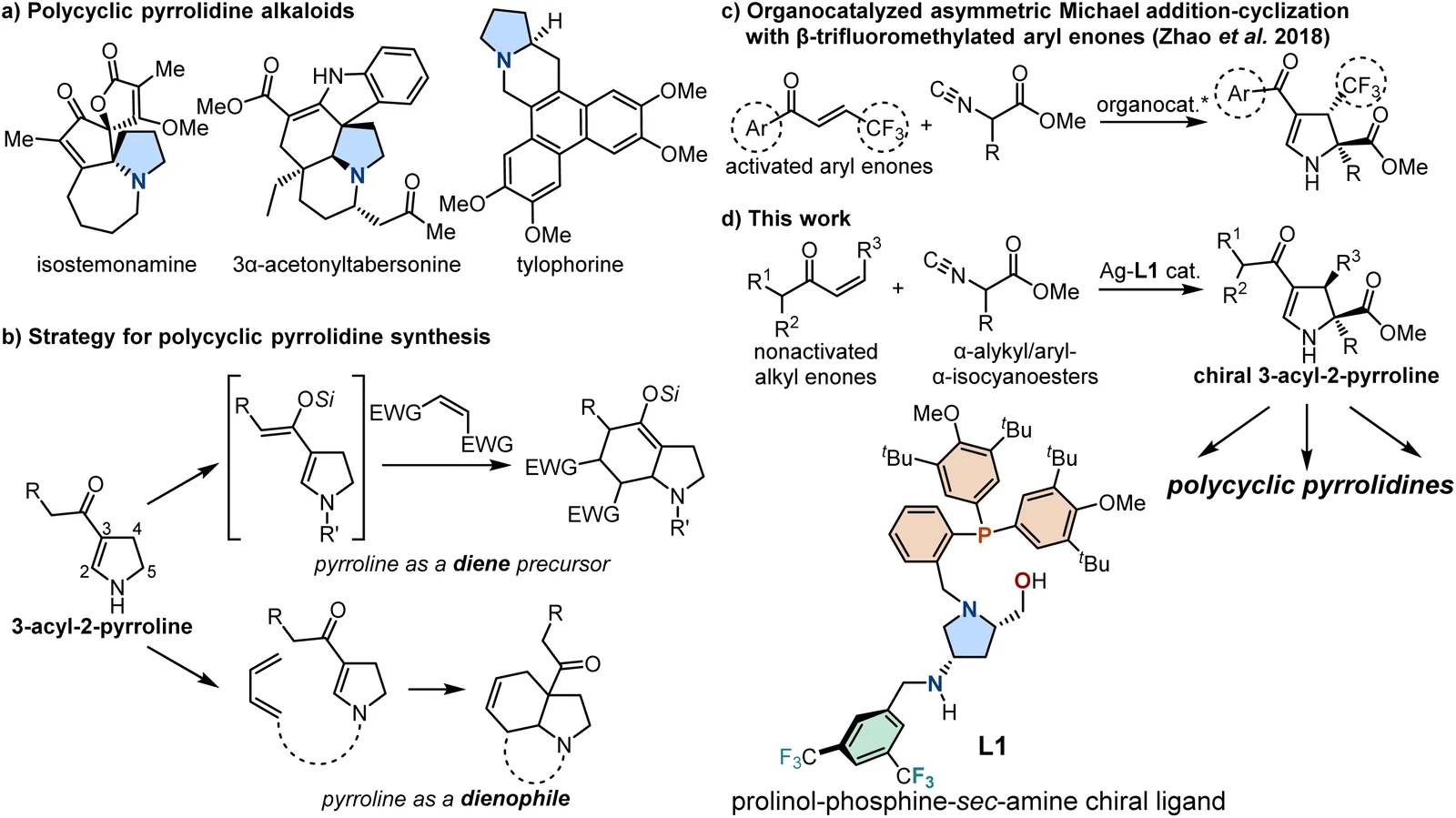
Toward polycyclic pyrrolidines.

Nitrogen-containing cyclic structures are frequently found in biologically active natural products and pharmaceuticals. In particular, polycyclic compounds bearing a pyrrolidine ring constitute important classes of alkaloids,^1^ including stemona,^2,3^ melodinus,^4,5^ and phenanthroindolizidine^6,7^ alkaloids (Scheme 1a). Given their diverse biological activities that strongly depend on subtle structural variations, there has been continuous demand for efficient synthetic methods which enable not only the construction of these privileged frameworks but also diversity-oriented synthesis (DOS) of structurally novel, unnatural analogues for drug discovery.^8–12^ From this perspective, the development of multifunctional synthetic hub molecules that allow rapid access to structurally distinct polycyclic systems is highly desirable. We envisioned that chiral 3-acyl-2-pyrrolines could serve as such attractive hub molecules (Scheme 1b). The α,β-unsaturated carbonyl moiety would provide pathways for constructing polycyclic structures. The exocyclic ketone moiety could be converted into an enol ether to generate an electron-rich diene moiety capable of engaging in Diels–Alder reactions with various dienophiles, while use of the electron-deficient C–C double bond as a dienophile for cycloadditions could allow a synthetic strategy for fusing additional ring systems to form polycyclic pyrrolidines.

Enantioselective tandem Michael addition–cyclization between isocyanoacetates and electron-deficient olefins represents a powerful and convergent approach to chiral 2-pyrrolines.^13^ In particular, the use of α-substituted isocyanoacetates as Michael donors is highly valuable, since it enables the construction of tetrasubstituted stereogenic carbon centers, structural motifs that are prevalent in biologically active molecules and represent important targets in modern synthetic and medicinal chemistry, while the reaction has to overcome the steric constraints during the C–C bond formation.^14^ After Gong's seminal report on the enantioselective variant of this reaction with nitroolefins using a quinine-derived organocatalyst,^15^ the scope of Michael acceptors has been expanded to include β,γ-unsaturated α-keto esters,^16^ enones,^17–19^ methyleneindolinones,^20^*N*-arylmaleimides,^21,22^ allenoates,^23^ and 1-azadienes.^24^ Nevertheless, while highly electrophilic Michael acceptors such as nitroolefins can tolerate β-substitution, the scope of conjugated enones has remained much more restricted. Specifically, acceptable β-substitution in the enones has essentially been limited to β-trifluoromethyl substitution on aryl ketones,^18,19^ which possess enhanced electrophilicity (Scheme 1c). Although chalcone-type β-substituted aryl enones are good Michael acceptors and have been widely employed in enantioselective conjugate additions,^25,26^ nonactivated β-substituted alkyl enones are considerably less explored in enantioselective isocyanoacetate-based Michael addition–cyclization reactions. In these substrates, the combination of reduced electrophilicity and steric congestion associated with the β-substitution makes selective 1,4-addition challenging, particularly in competition with undesired 1,2-addition pathways. Consequently, the application of the enantioselective catalysis for the synthesis of structurally complex molecules has been limited.

Herein, we report a robust silver-catalyzed enantioselective Michael addition–cyclization between α-substituted isocyanoacetates and nonactivated β-substituted enones, which produces enantioenriched 3-acyl-2-pyrrolines with consecutive stereogenic centers at the C4 and C5 positions (Scheme 1d). Notably, the latter is a sterically congested configurationally stable tetrasubstituted stereogenic center. The silver catalyst bearing prolinol-phosphine-*sec*-amine ligand L1 exhibited high reactivity,^27,28^ high diastereo- and enantioselectivities, and high chemoselectivity favoring 1,4-addition over competing 1,2-addition. Moreover, the resulting chiral pyrrolines can be transformed into a variety of polycyclic pyrrolidines, demonstrating that the enantioselective silver catalysis can serve as a platform for strategies to access structurally complex molecules.

## Results and discussion

**Fig. 1:**
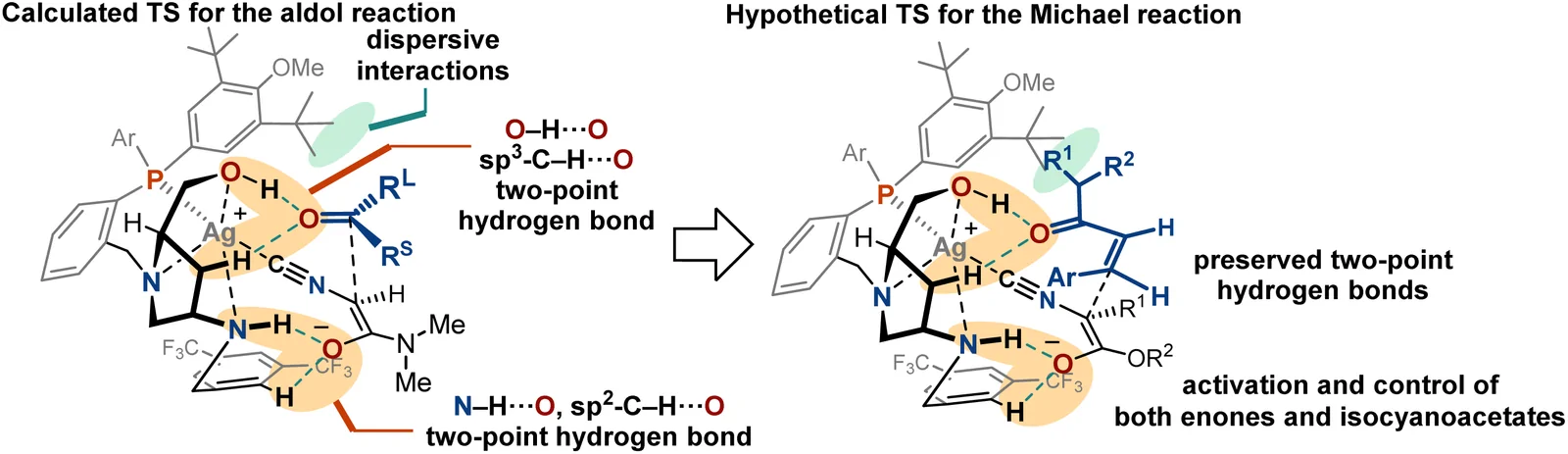
Hypothesis of a transition state for enantioselective Michael addition of isocyanoesters with Ag-L1.

We recently reported the silver-catalyzed enantioselective aldol reactions of isocyanoacetamides with nonactivated ketones using our originally developed prolinol-phosphine-*sec*-amine chiral ligand (L1).^27,29^ The high reactivity and stereoselectivity were achieved by cooperative action of the metal and the chiral ligand^30^ as shown in Fig. 1. Characteristically, the catalyst bound both the enolate anion and the ketone through their respective two-point hydrogen-bonding motifs consisting of a weak C–H⋯O interaction (nonclassical hydrogen bond) and a heteroatom-based conventional hydrogen bond.^31–37^ London dispersion interactions also contributed to the controlled catalysis.^38–41^

Then, we envisioned that a similar catalyst system would be a promising tool for exploring the enantioselective Michael addition–cyclization with isocyanoacetic acid derivatives, opening a route to enantioenriched 3-acyl-2-pyrrolines with tetrasubstituted stereogenic carbon centers (Fig. 1) (see SI for preliminary DFT calculations for the Michael reaction–cyclization).

In examining various chiral prolinol-phosphine and other ligands to evaluate their performance in the silver-catalyzed enantioselective Michael addition–cyclization, the prolinol-phosphine-*sec*-amine L1 was identified to be optimal. Specifically, the reaction between nonactivated enone (*Z*)-cinnamyl methyl ketone (1a, 0.38 mmol) and methyl α-isocyanopropionate (2a, 0.25 mmol) in the presence of Ag(acac) (2 mol%) and L1 (2 mol%) in toluene at −30 °C afforded 3-acetyl-2-pyrroline-5-carboxylate (4*R*,5*S*)-3aa preferentially in 70% yield with dr 92 : 8 and 96% ee over the corresponding 1,2-addition–cyclization product (2-oxazoline-4-carboxylate), which was formed only in 12% yield (Table 1, entry 1). When the *P*-3,5-di-*t*-butylphenyl (DTBM) groups of L1 were changed to simple *P*-phenyl groups (L2), the yield and stereoselectivities decreased (56% yield, dr 89 : 11, 92% ee, entry 2), indicating the effect of dispersive interactions in the catalysis employing bulky DTBM-type ligand L1. Replacement of the electron-deficient monobenzylamino group at the C4 position of the 2-pyrrolidinemethanol of L1 with an unsubstituted benzylamino group (L3) also decreased the yield, diastereo- and enantioselectivities (53% yield, dr 87 : 13, 51% ee, entry 3). 4-MeO-substituted ligand L4 further decreased the yield and stereoselectivity (21% yield, dr 78 : 22, 44% ee, entry 4), while 4-CF_3_-substituted ligand L5 exhibited moderate performance (57% yield, dr 84 : 16, 62% ee, entry 5). Thus, the electronic substituent effects on the benzene ring are similar to those observed in the silver-catalyzed aldol reaction that we reported recently,^27^ implying mechanistic similarity between the two reactions. When a carbamate-type ligand (L6) bearing a stronger hydrogen-bonding site with enhanced N–H acidity was employed, the stereoselectivity was higher (59% yield, dr 91 : 9, 90% ee, entry 6) than that obtained with simple benzylamino substituted ligand L3. When a hydroxy group was introduced (L7) instead of the benzylamino group of L1, the yield decreased significantly with retention of the high stereoselectivity (33% yield, dr 92 : 8, 94% ee, entry 7). In addition, the ligand lacking a C4 substitution (L8) gave lower stereoselectivity than that with L1 (72% yield, dr 85 : 15, 93% ee, entry 8).

**Table 1:** Optimization of silver-catalyzed enantioselective Michael addition–cyclization^a^

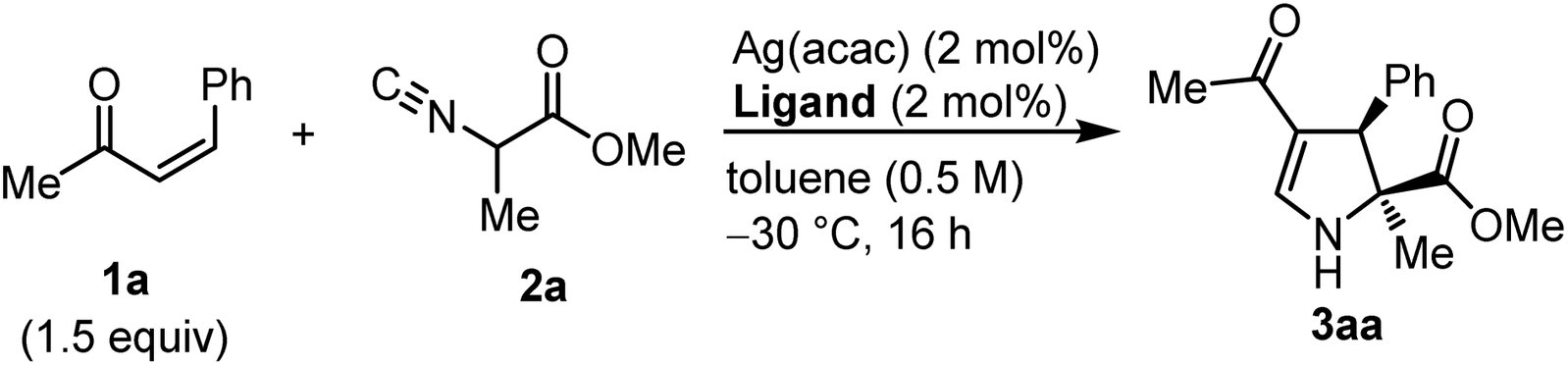				
Entry	Ligand	Yield (%)	dr	ee (%)
1	L1	70 (63)	92 : 8	96/30
2	L2	56	89 : 11	92/35
3	L3	53	87 : 13	51/21
4	L4	21	78 : 22	44/3
5	L5	57	84 : 16	62/17
6	L6	59	91 : 9	90/30
7	L7	33	92 : 8	94/34
8	L8	72	85 : 15	93/77
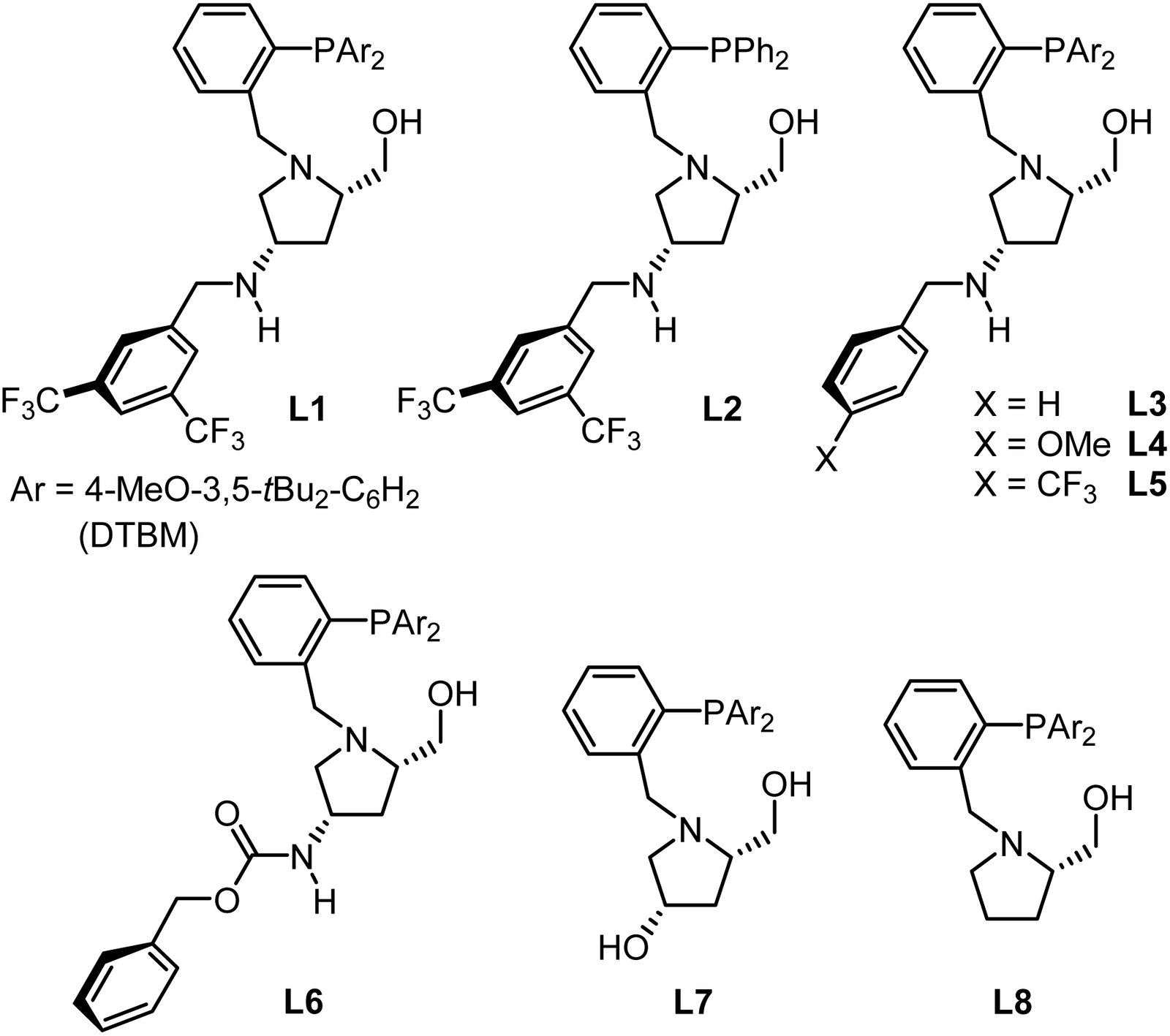				

a 1a (0.38 mmol), 2a (0.25 mmol), Ag(acac) (0.005 mmol), ligand (0.005 mmol), toluene (0.5 M), −30 °C, 16 h. Yields and dr were determined by ^1^H NMR analysis of crude material using 1,3,5-trimethoxybenzene as an internal standard.

**Scheme 2:**
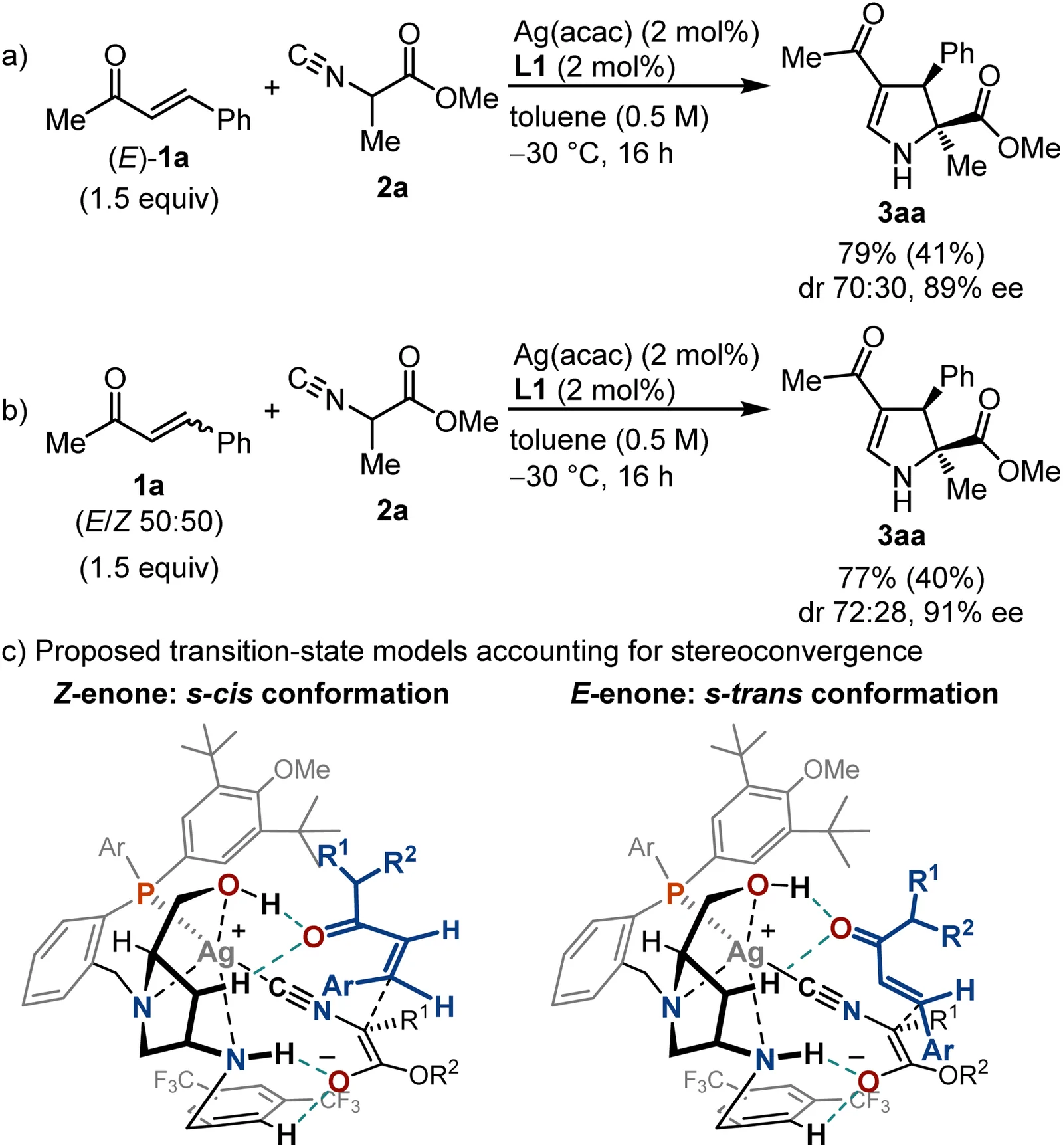
Effect of the *E*/*Z* configuration of 1a. (a) Reaction using (*E*)-1a. (b) Reaction using an *E*/*Z* mixture of 1a. (c) Proposed transition-state models accounting for the stereoconvergence.

We also evaluated the influence of the *E*/*Z*-configuration of the enone under the optimal conditions with the Ag-L1 system. The reaction of (*E*)-1a (0.38 mmol) and 2a (0.25 mmol) produced 3aa in a yield comparable to that obtained from (*Z*)-1a albeit with lower diastereo- and enantioselectivity (79% yield, dr 70 : 30, 89% ee, Scheme 2a). Notably, the stereochemistry of the product was identical regardless of the *E*/*Z* configuration of the enone. Thus, the reaction is stereoconvergent. Taking advantage of the stereoconvergence, the reaction was performed with a 50 : 50 *E*/*Z* mixture of 1a, which was more readily available than the pure isomers, affording (4*R*,5*S*)-3aa in 77% yield with dr 72 : 28 and 91% ee (Scheme 2b). Analysis of the crude mixture revealed that the *E*-isomer reacted more rapidly, whereas the *Z*-isomer was consumed only to a limited extent (∼10% conversion). A plausible origin of this stereoconvergence is conformational selection of the enone substrates within the chiral Ag-L1 environment (Scheme 2c). The *Z*-enone is expected to predominantly adopt an *s-cis* conformation, which is favored by an intramolecular sp^2^-C–H⋯O interaction (see SI). In contrast, for the *E*-enone, the *s-cis* and *s-trans* conformations are more readily accessible. However, the *s-cis* conformation would suffer from unfavorable steric interactions upon accommodation in the catalyst pocket. Therefore, the *E*-enone is expected to react preferentially through an *s-trans*-type conformation. Although the reactive conformations of the *E*- and *Z*-enones are different, both substrates can be organized by the Ag-L1 catalyst through O–H⋯O and sp^3^-C–H⋯O two-point hydrogen-bonding interactions. This catalyst-controlled organization directs Michael addition from the same enantioface, leading to the same major stereoisomer after cyclization. The faster consumption of the *E*-isomer than the *Z*-isomer is also consistent with this conformational-selection model.

**Scheme 3:**
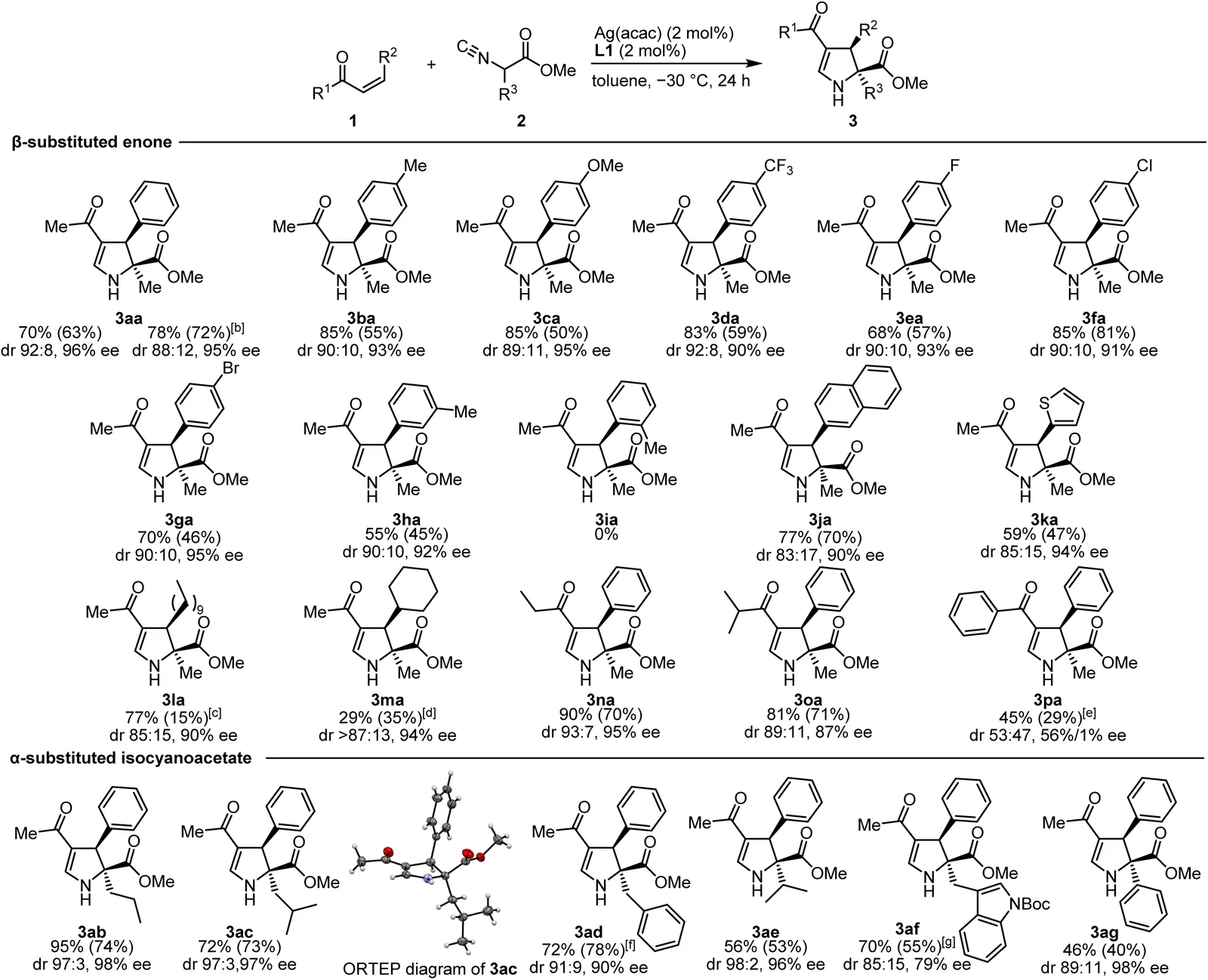
Substrate scope. ^*a*^1 (0.38 mmol), 2 (0.25 mmol), Ag(acac) (0.005 mmol), L1 (0.005 mmol), toluene (0.5 M), −30 °C, 24 h. Yields and dr were determined by ^1^H NMR analysis of crude materials using 1,3,5-trimethoxybenzene as an internal standard. Yields of isolated major diastereomers (dr ≧ 20 : 1) are shown in parentheses. Enantiomeric excesses determined by HPLC analysis on chiral stationary phases are shown for the major diastereomers. ^*b*^1 mmol-scale. ^*c*^30 : 70 *E*/*Z* mixture of 1l was used. ^*d*^65 : 35 *E*/*Z* mixture of 1m was used. ^*e*^28 : 72 *E*/*Z* mixture of 1p was used. Isolated yield for mixture of diastereomers (dr 70 : 30). ^*f*^Isolated yield for mixture of diastereomers (dr 89 : 11). ^*g*^Isolated yield for mixture of diastereomers (dr 84 : 16).

For investigating the scope of β-substituted enones, *Z*-isomers were employed as substrates. It should be noted that the *Z*-isomers were readily prepared by *Z*-selective Horner–Wadsworth–Emmons reaction of aldehydes and bis(2,2,2-trifluoroethyl) phosphonates.^42^ The conditions optimized for the reaction between (*Z*)-1a and 2a (Table 1, entry 1) were adopted except that the reaction time was extended to 24 h to ensure completion of the reaction (Scheme 3). The reaction between 1a and 2a (1a/2a 1.5 : 1) could be conducted at 1 mmol-scale, affording 3aa in 78% yield, dr 88 : 12, and 95% ee. Then, the effects of *para*-substitution on the β-aryl group were examined. Both electron-donating and -withdrawing substituents, methyl (1b), methoxy (1c), and trifluoromethyl (1d) groups, increased the yields with retention of the high stereoselectivity (3ba, 85% yield, dr 90 : 10, 93% ee; 3ca, 85% yield, dr 89 : 11, 95% ee; 3da, 83% yield, dr 92 : 8, 90% ee). 4-Fluoro- (1e), chloro- (1f), and bromo-substitution (1g) did not affect the stereoselectivities (3ea, 68% yield, dr 90 : 10, 93% ee; 3fa, 85% yield, dr 90 : 10, 91% ee; 3ga, 70% yield, dr 90 : 10, 95% ee). A *meta*-methyl substituent (1h) was competent (3ha, 55% yield, dr 90 : 10, 92% ee), but an *ortho*-methyl substituent (1i) completely inhibited the reaction. Meanwhile, a sterically demanding π-extended naphthyl group (1j) instead of the phenyl group at the β-position was acceptable albeit with moderate diastereoselectivity (3ja, 77% yield, dr 83 : 17, 90% ee). The reaction with β-thiophenyl enone 1k also proceeded in 59% yield with moderate diastereoselectivity and high enantioselectivity (3ka, dr 85 : 15, 94% ee). β-Substituents of the enone were not limited to aryl groups, but aliphatic decyl and cyclohexyl groups (1l, 30 : 70 *E*/*Z* mixture, 1m, 65 : 35 *E*/*Z* mixture) were acceptable as the substituents at the β-position of the enone, producing 3la (77% yield, dr 85 : 15, 90% ee) and 3ma (29% yield, dr > 87 : 13, 94% ee), respectively. It should be noted that 3la was unstable and gradually decomposed during the isolation by preparative TLC (thin-layer-chromatography, silica gel), reducing the isolated yield to 15%. Moreover, the enone was not restricted to methyl ketone; ethyl (1n) and sterically more demanding isopropyl (1o) ketones afforded the corresponding products in high yields and with high stereoselectivities (3na, 90% yield, dr 93 : 7, 95% ee, 3oa, 81% yield, dr 89 : 11, 87% ee). Chalcone (1p, 28 : 72 *E*/*Z* mixture), a representative aryl enone, also showed moderate reactivity while diastereo- and enantioselectivities were significantly lower than those of alkyl enones (3pa, 45% yield, dr 53 : 47, 56% ee).

Next, the scope of α-substituted isocyanoacetates (2) was investigated (Scheme 3). The reaction of 2b bearing an α-propyl group proceeded efficiently, affording 3ab in 96% yield, dr 97 : 3, and 98% ee. α-Isobutyl (2c) and α-benzyl (2d) groups were tolerated, giving the products 3ac and 3ad, respectively, in high stereoselectivities (3ac, 72% yield, dr 97 : 3, 97% ee; 3ad, 72% yield, dr 91 : 9, 90% ee). Single-crystal X-ray diffraction analysis of 3ac established the absolute configurations of the major diastereomer to be 4*R*,5*S*.^43^ Moreover, a sterically more demanding α-isopropyl group (2e) was also acceptable, affording the corresponding pyrroline (3ae) with a highly congested tetrasubstituted stereocenter in 56% yield with excellent stereoselectivities (dr 98 : 2, 96% ee). An *N*-Boc indolyl group was tolerated under the reaction conditions, providing 3af in 70% yield with slightly diminished stereoselectivity (dr 85 : 15, 79% ee). In addition to these isocyanoacetates with α-alkyl groups, α-phenyl isocyanoacetate 2g was also a competent substrate, affording 3ag in 46% yield with dr 89 : 11 and 98% ee.

**Scheme 4:**
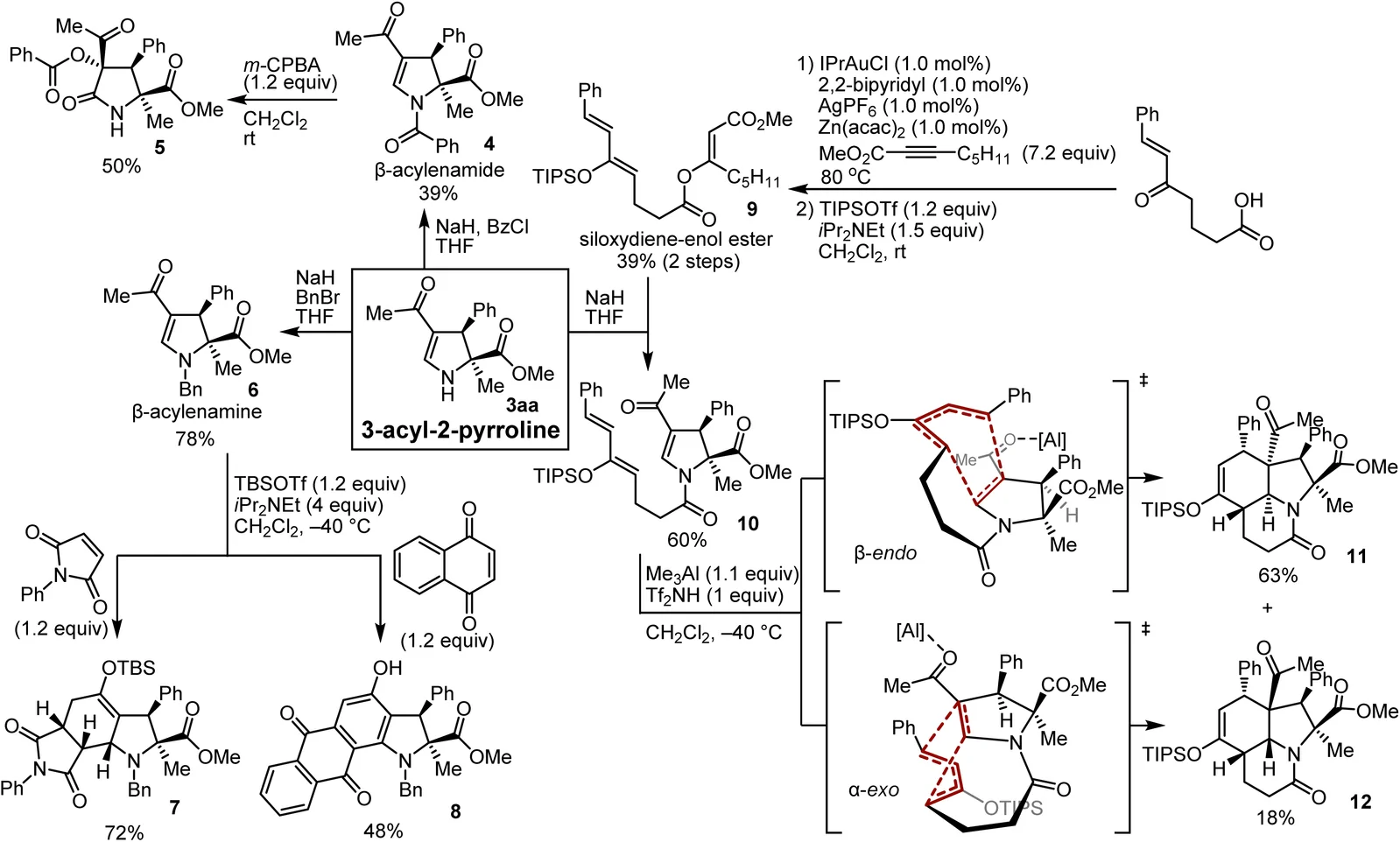
Transformations of 3-acyl-2-pyrroline 3aa to polycyclic pyrrolidines.

With the efficient catalysis providing access to various chiral 3-acyl-2-pyrrolines in hand, we then explored the transformations of 3aa (Scheme 4). After *N*-benzoylation of 3aa, the resulting β-acylenamide 4 was treated with *meta*-chloroperoxybenzoic acid (*m*-CPBA), affording densely substituted 2-pyrrolidone 5 stereoselectively in 50% yield. The formation of this compound can reasonably be explained by the initial epoxidation followed by epoxy-ring-opening and successive N-to-O aroyl migration, producing a cyclic imine intermediate, which would undergo the second oxidation by *m*-CPBA to yield the pyrrolidone (5).

Next, 3aa was used as a competent diene precursor for intermolecular Diels–Alder reactions (Scheme 4). After *N*-benzylation of 3aa, the β-acylenamine 6 was *in situ* converted into a siloxydiene by treatment with *i*Pr_2_NEt and TBSOTf in the presence of *N*-phenylmaleimide at −30 °C to afford tricyclic pyrrolidine 7 in 72% yield with exclusive α-*endo* selectivity.

Upon switching the dienophile to naphthoquinone, the Diels–Alder reaction proceeded smoothly at the same temperature, affording after desilylation-aerobic oxidation during workup an interesting tetracyclic anthraquinone–pyrrolidine hybrid molecule (8) in 48% yield.

Then, we also investigated an intramolecular Diels–Alder reaction, using the endocyclic alkene of the acylpyrroline as a dienophile (Scheme 4). First, *N*-acylation of 3aa was performed with activated enol ester 9 bearing a siloxydiene moiety, giving the product 10 in 60% yield. Note that the siloxydiene-enol ester (9) was prepared through the gold-catalyzed alkynoate hydrocarboxylation, which we developed recently as a means for peptide synthesis.^44^ This activated ester was particularly suitable for the *N*-acylation, whereas attempted reactions with a common activated ester, pentafluorophenyl ester, gave the product in at most 39% yield with less reproducibility. Next, various Lewis acids were examined for promotion of the intramolecular Diels–Alder reaction (see SI for details), and we found that Me_2_AlNTf_2_ (Me_3_Al/HNTf_2_ 1.1 : 1) showed^45^ good reactivity in CH_2_Cl_2_ at −40 °C to form pyrrolidine-containing tricyclic frameworks with six stereogenic carbon centers, two of which are tetrasubstituted. Two major diastereomers (dr 72 : 28) could be separated by silica gel column chromatography, giving β-*endo* product 11 and α-*exo* product 12 in 63% and 18% yields, respectively.

## Conclusions

We developed a silver-catalyzed diastereo- and enantioselective Michael reaction between α-substituted isocyanoacetates and nonactivated β-substituted enones using a chiral prolinol-phosphine-*sec*-amine ligand. The catalysis afforded various chiral 3-acyl-2-pyrrolines with consecutive tertiary and tetrasubstituted stereogenic centers with different substituents and high stereoselectivities. In this study, we showed that weak nonclassical C–H⋯O hydrogen bonds (induced dipole–dipole interactions) played a crucial role in stereocontrol as partners of proximal stronger heteroatom-based normal hydrogen bonds, constituting two-point hydrogen bond motifs that orient carbonyl groups of the substrates in a well-defined manner. Utility of this silver-catalyzed enantioselective reaction was demonstrated by using a representative chiral acylpyrroline as a hub molecule for synthesizing pyrrolidine-containing polycyclic molecules, aiming at diversity-oriented synthesis (DOS) of drug-like molecules. Further studies to enlarge the library and evaluating biological activities are underway.

## Author contributions

YS and MS conceived the work and designed the experiments. SS and KM performed the experiments. SS performed the DFT calculations. SS, KM, YS and MS analysed the data and cowrote the manuscript.

## Conflicts of interest

There are no conflicts to declare.

## Supplementary Material

SC-017-D6SC04627D-s001

SC-017-D6SC04627D-s002

## Data Availability

CCDC 2530817 contains the supplementary crystallographic data for this paper.^43^ The data supporting this article have been included as part of the supplementary information (SI). Supplementary information is available. See DOI: https://doi.org/10.1039/d6sc04627d.
