# Supplementary material for: Chiral 3-acyl-2-pyrrolines as strategic hubs toward polycyclic pyrrolidines via enantioselective silver-catalyzed Michael addition–cyclization
Source: Chem Sci. 2026 Jul 16;17(35):16892–8. doi: 10.1039/d6sc04627d (PMC13387387; doi:10.1039/d6sc04627d)
Supplement: Supplementary file 1 [file SC-017-D6SC04627D-s001.pdf]

Supplementary Information (SI) for Chemical Science.
This journal is © The Royal Society of Chemistry 2026

Chiral 3-Acyl-2-pyrrolines as Strategic Hubs toward Polycyclic Pyrrolidines *via* Enantioselective Silver-Catalyzed Michael Addition–Cyclization

Supporting Information

Table of Contents

Instrumentation and chemicals	**S1**
Effects of silver sources	**S2**
DFT calculations	**S3-S4**
Effects of Lewis acids for intramolecular Diels-Alder reaction	**S5**
Experimental procedure	**S6**
Characterization of substrates and products	**S7-S29**
Transformations	**S29-S38**
Single crystal XRD	**S38-S39**
References	**S40**
NMR spectra	**S41-S138**

Instrumentation and chemicals

NMR spectra were recorded on a JEOL ECX-400, operating at 400 MHz for ^{1}H NMR and 100.5 MHz for ^{13}C NMR. Chemical shift values for ^{1}H and ^{13}C are referenced to Me_4Si or a residual solvent peak. Chemical shifts are reported in δ ppm. Mass spectra were obtained with Thermo Scientific Exactive or JMS-T2000GC at Global Facility Center, Creative Research Institution, Hokkaido University. HPLC analyses were conducted on a SHIMADZU HPLC system with a SHIMADZU SPD-M40 photo diode array detector. TLC analyses were performed on commercial glass plates bearing a 0.25-mm layer of Merck Silica gel $60F_{254}$. Kanto Chemical Co., Silica gel 60 N, spherical, neutral and Merck, Silica Gel 60 PF254 for PLC were used for column chromatography and preparative thin-layer chromatography, respectively.

All reactions were carried out under a nitrogen or argon atmosphere. Materials were obtained from commercial suppliers or prepared according to standard procedures unless otherwise noted. Ag(acac) was purchased from Sigma-Aldrich Co. Chiral prolinol-phosphine ligands **L1**-**L8** were prepared according to the previous report from our group.[1] Isocyanoacetates were prepared according to the reported procedure.[2] Toluene was purchased from FUJIFILM Wako Pure Chemical Corporation.

Effects of silver sources

Silver sources were investigated using the reaction between (*Z*)-cinnamyl methyl ketone (**1a**, 0.20 mmol, 1 equiv) and methyl α-isocyanopropionate (**2a**, 0.20 mmol) in the presence of chiral ligand **L1** (Table S1). With 5 mol% Ag(acac)-**L1** catalyst, Michael addition-cyclization product **3aa** was obtained in 68% yield and high stereoselectivity (dr 94:6, 97% ee, entry 1). The catalyst loading could be reduced to 2 mol% by using 1.5 equiv of **1a** without eroding the yield and the stereoselectivity (70% yield, dr 92:8, 96% ee, entry 2). Ag_2CO_3 (2.5 mol%) was also a competent silver source, affording **3aa** in comparable yield and stereoselectivity (68% yield, dr 93:7, 96% ee, entry 3), while AgOAc (5 mol%) showed significantly lower reactivity and stereoselectivities (24% yield, dr 80:20, 77% ee, entry 4). The combination of a cationic silver source $AgBF_4$ (5 mol%) and K_2CO_3 (10 mol%) was competent in terms of stereoselectivity (dr 92:8, 96% ee) but the yield was reduced to 30% (entry 5).

Table S1. Effects of silver sources.[a]

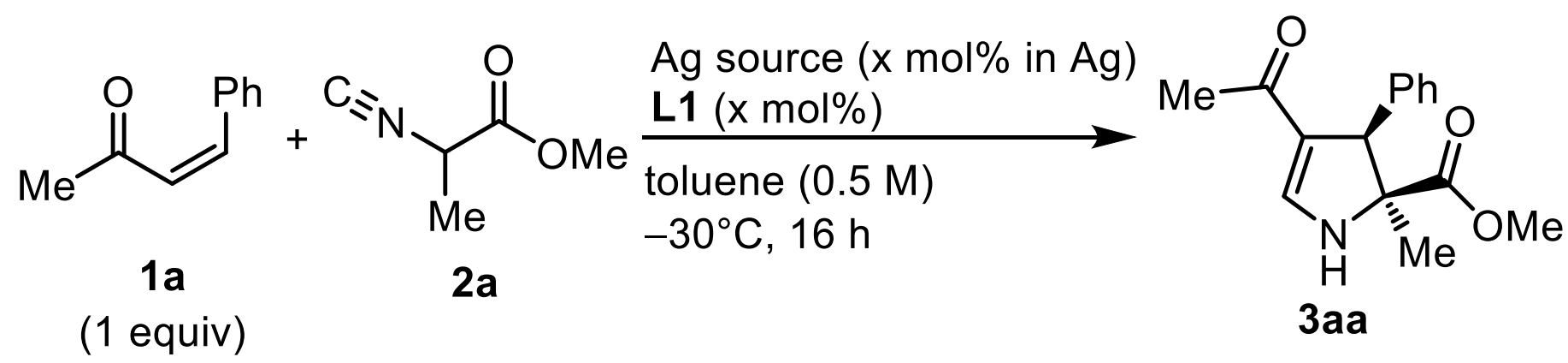

Entry	Ag source	Yield (%)	Dr	Ee (%)
1	Ag(acac) (5 mol%)	68	94:6	97/24
2[b]	Ag(acac) (2 mol%)	70 (63)	92:8	96/30
3	Ag_2CO_3 (2.5 mol%)	68	93:7	96/24
4	AgOAc (5 mol%)	24	80:20	77/14
5[c]	$AgBF_4$ (5 mol%)	30	92:8	96/28

[a] **1a** (0.20 mmol), **2a** (0.20 mmol), Ag source (x mol% in Ag), **L1** (x mol%), toluene (0.5 M), –30 °C, 16 h. Yields and dr were determined by ^{1}H NMR analysis of crude material using 1,3,5-trimethoxybenzene as an internal standard. Isolated yield of the major diastereomer (4*R*,5*S*)-**3aa** is shown in parentheses. Enantiomeric excesses determined by HPLC analysis on a chiral stationary phase are shown. [b] **1a** (0.38 mmol) and **2a** (0.25 mmol) were used. [c] K_2CO_3 (0.02 mmol) was added.

DFT calculations

Preliminary DFT calculations for Ag-**L1**-catalyzed enantioselective Michael reaction of (*Z*)-enone **1a** with isocyanoacetate **2a** to produce (4*R*,5*S*)-**3aa** were conducted at the ωB97X-D/6-31G(d), SDD level of theory.[3] All geometry optimizations and single-point calculations were performed by Gaussian 16 package.[4] The geometry optimizations as well as frequency calculations of all structures were conducted at the ωB97XD functional in conjunction with the SDD (for Ag) and 6-31G(d) (for others) basis set. Frequency analyses were performed at the same level of theory as geometry optimization, in which the thermal free energy corrections were provided. All transition states have a single imaginary frequency, and these transition states were traced with intrinsic reaction coordinate (IRC) analyses using Global Reaction Route Mapping (GRRM) program[5] to describe the reaction pathway.

A reaction pathway diagram with relative Gibbs free energies at 25 °C is shown in Figure S1. Regarding the conformation of (*Z*)-enone **1a**, the *s-cis* form is more stable than the *s-trans* form by 1.0 kcal/mol due to the existence of an intramolecular sp^2-C–H···O hydrogen bond in the former. In the precursor (**Int1**) of enolate addition, the carbonyl group of **1a** forms a two-point hydrogen bond with **L1** through the Ag-bound OH group and the proximal sp^3-C–H bond at the ring C3-position of 2-pyrrolidinemethanol. In addition, the oxyanion of the enolate forms a two-point hydrogen bond donated by the NH group and the sp^2-C–H bond of the benzylamino group of **L1**. The precursor complex (**Int1**) undergoes enolate addition, producing Michael adducts **Int2** (–5.1 kcal/mol) through **TS1** (6.6 kcal/mol). The successive pyrroline ring formation proceeds through **TS2** (5.3 kcal/mol), producing C2-metalated pyrroline intermediate (**Int3**, –17.5 kcal/mol). The energy barrier of **TS1** is higher than that of **TS2**, indicating that C–C bond formation is the rate-determining step of the reaction. The transition states (**TS1** and **TS2**) leading to the major isomer are stabilized by multiple hydrogen bonds including nonclassical hydrogen bonds, sp^3-C–H···O and sp^2-C–H···O. The carbonyl group of **1a** forms a two-point hydrogen bond with **L1** through the Ag-bound OH group and the proximal sp^3-C–H bond at the ring C3-position of 2-pyrrolidinemethanol. In addition, the carbonyl group of **2a** forms a two-point hydrogen bond donated by the NH group and the sp^2-C–H bond of **L1**. The two sets of two-point hydrogen bonds are maintained in both **TS1** and **TS2**, leading to the stabilization of the transition states.

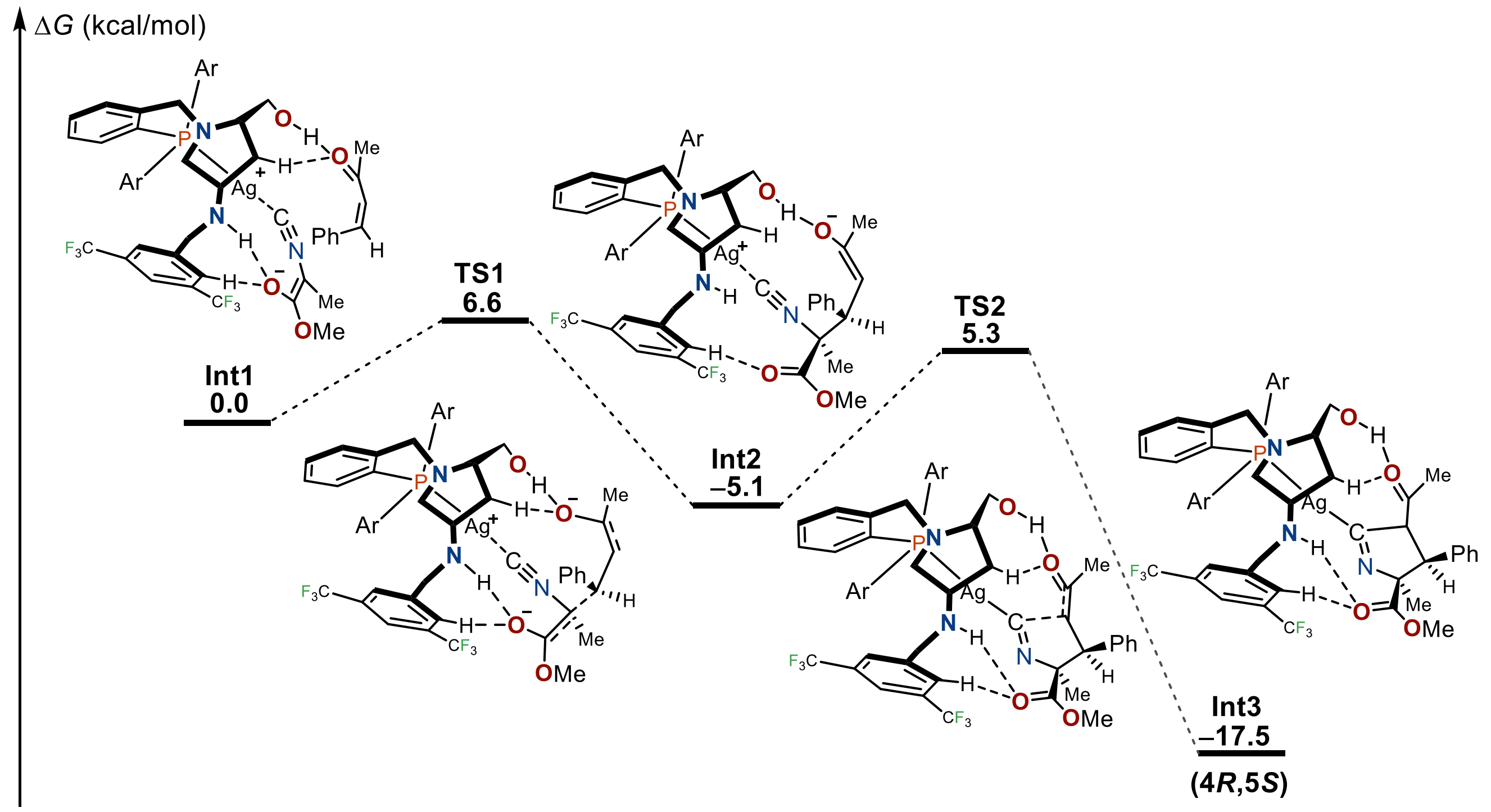

Figure S1. Energy diagram of Ag-**L1**-catalyzed enantioselective Michael reaction of **1a** and **2a**. The relative free energies at 25 °C are given in kcal/mol.

Table S2. Summary of Energies

	Gibbs Free Energy (G) (Hartree)
**Int1**	-4265.639095
**TS1**	-4265.628566
**Int2**	-4265.647235
**TS2**	-4265.630694
**Int3**	-4265.667023

Effects of Lewis acids for intramolecular Diels-Alder reaction

Table S3. Effects of Lewis acids.[a]

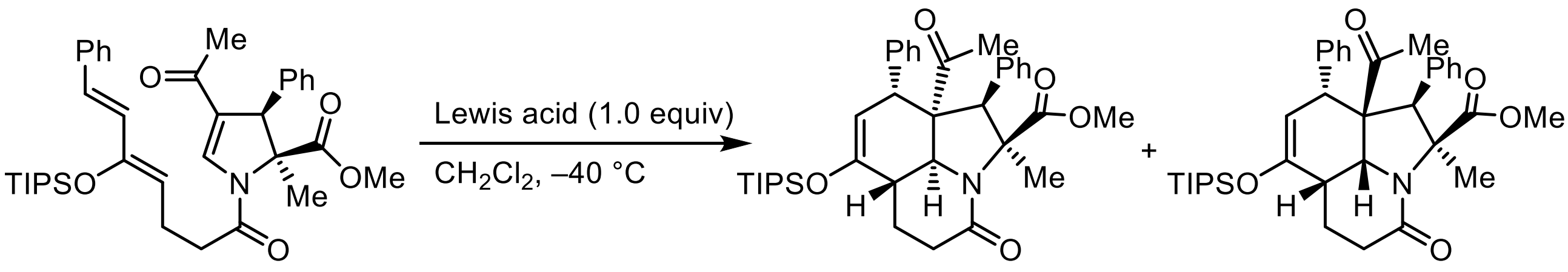

Entry	Lewis acid	Yield (%)	**11**:**12**
1	Et_2AlCl	50	16:84
2	$i$$Bu_2AlCl$	16	6:94
3	$i$$Bu_3AlCl$	0	–
4	$Me_3Al/HNTf_2$ (1.2:1)	65	65:35
5	$i$$Bu_3Al/HNTf_2$ (1.2:1)	62	50:50
6	$Me_3Al/HNTf_2$ (1.2:2)	9	75:25
7	$TiCl_3$	24	1:>20
8	$B(C_6F_5)_3$	16	>20:1
9	$Sc(OTf)_3$	0	–
10[b]	$Me_3Al/HNTf_2$ (1.1:1)	90 (81)	72:28 (78:22)

[a] **10** (0.05 mmol), Lewis acid (0.05 mmol), CH_2Cl_2 (1.0 mL), –40 °C, 3 h. Yields and dr were determined by ^{1}H NMR analysis of crude materials using 1,1,2,2-tetrachloroethane as an internal standard. [b] 0.1 mmol scale. Yield and diastereomeric ratio of isolated product are shown in parentheses.

Experimental procedure

General procedure for the preparation of (*Z*)-enones

(*Z*)-Enones (**1a-1l**) were synthesized by *Z*-selective HWE reactions according to the literature.[40]

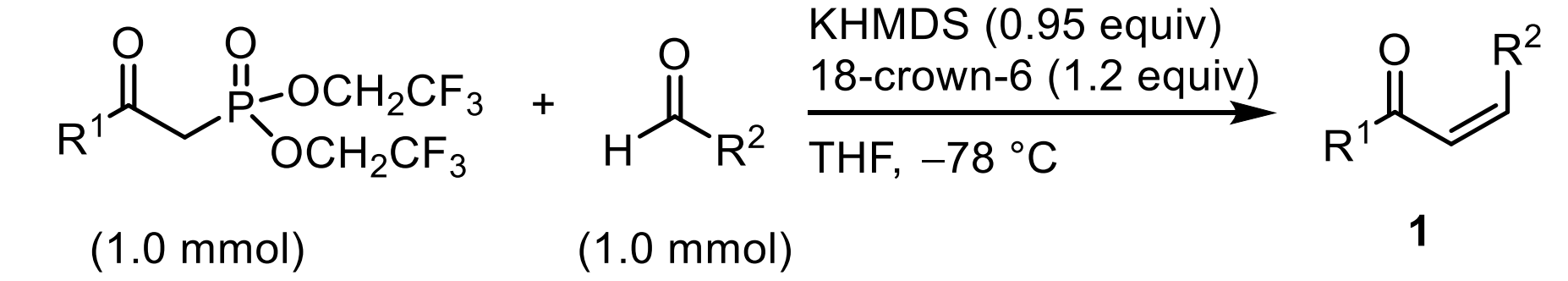

To a solution of KHMDS (189 mg, 0.95 mmol) and 18-crown-6 (317 mg, 1.2 mmol) in 5 mL anhydrous THF at –78 °C was added bis(2,2,2-trifluoroethyl)-phosphonate (302 mg, 1.0 mmol) under argon atmosphere. After stirring the resulting mixture for 20 min at the same temperature, aldehyde (1.0 mmol) was added. The reaction was stirred at –78 °C, typically for 2 hours, with the progress monitored by TLC analysis. After completion, the reaction was quenched with saturated aqueous NH_4Cl solution (5 mL), extracted with ether three times, dried over anhydrous $MgSO_4$, filtered and concentrated under reduced pressure. The residue was purified by flash column chromatography to give the product **1** as yellow liquid or solid.

General procedure for the silver-catalyzed asymmetric Michael reactions

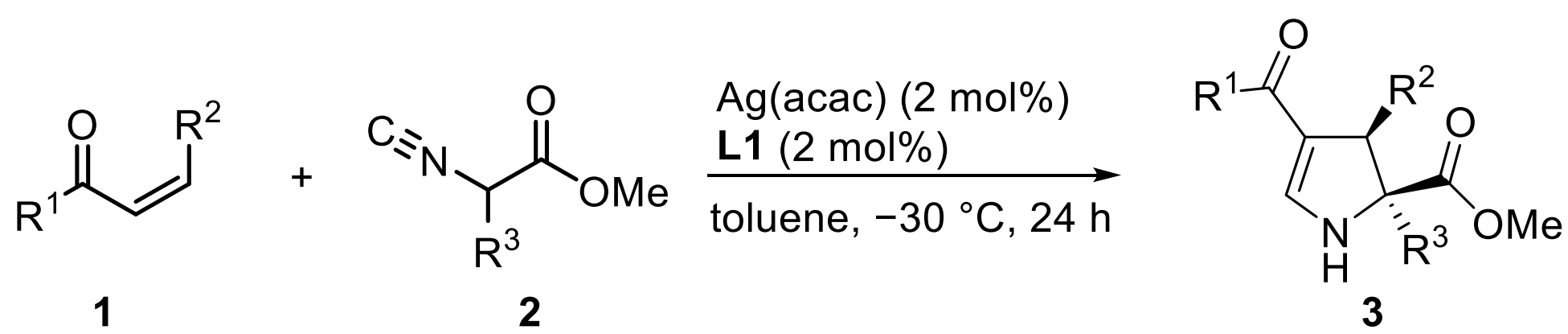

To a solution of Ag(acac) (1.0 mg, 0.005 mmol) and **L1** (4.5 mg, 0.005 mmol) in toluene (0.5 mL) in a vial was added α,β-unsaturated ketone **1** (0.38 mmol, 1.5 equiv). The mixture was cooled to –30 °C and α-substituted isocyanoacetate **2** (0.25 mmol, 1.0 equiv) was added with a micro syringe. After stirring at the same temperature for 24 hours, the reaction mixture was filtered through a short plug of silica gel (0.5 g) with EtOAc as an eluent and concentrated under reduced pressure. A residue was purified by preparative TLC (eluent: EtOAc) to give the product **3**. The ee value was determined by chiral HPLC analysis.

General procedure for the preparation of racemic products

To a solution of Ag_2CO_3 (1.4 mg, 0.005 mmol) and PPh_3 (5.2 mg, 0.01 mmol) in toluene (0.5 mL) in a vial was added α,β-unsaturated ketone **1** (*trans* or *cis,* 0.05 mmol, 1.0 equiv) and then α-substituted isocyanoacetate **2** (0.05 mmol, 1.0 equiv). After stirring at room temperature for 24 hours, the reaction mixture was filtered through a short plug of silica gel (0.5 g) using EtOAc as the eluent and concentrated under reduced pressure. The resulting residue was purified by preparative TLC (eluent: EtOAc) to give the product **3**.

Characterization of substrates and products

(*Z*)-4-Phenylbut-3-en-2-one (1a)

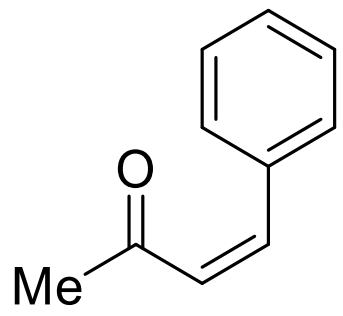

1a

62.9 mg, 43% yield, yellow oil.

^{1}H NMR (400 MHz, $CDCl_3$) δ 7.49-7.47 (m, 2H), 7.38-7.35 (m, 3H), 6.91 (d, *J* = 12.8 Hz, 1H), 6.18 (d, *J* = 12.8 Hz, 1H), 2.16 (s, 3H).

^{13}C NMR (101 MHz, $CDCl_3$) δ 201.1, 140.1, 135.3, 129.35, 129.31, 129.1, 128.3, 30.9.

IR(ATR) 3025, 1691, 1667, 1604, 1353,1163, 973, 774, 690 cm^{-1}.

HRMS (ESI) *m/z*: $[M+Na]^+$ Calcd for $C_{10}H_{10}ONa$ 169.0624; Found. 169.0623.

(*Z*)-4-(p-Tolyl)but-3-en-2-one (1b)

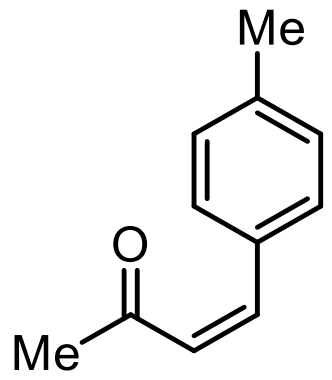

1b

63.3 mg, 40% yield, yellow oil.

^{1}H NMR (400 MHz, $CDCl_3$) δ 7.41 (d, *J* = 8.4 Hz, 2H), 7.16 (d, *J* = 8.4 Hz, 2H), 6.84 (d, *J* = 12.8 Hz, 1H), 6.13 (d, *J* = 12.8 Hz, 1H), 2.36 (s, 3H), 2.16 (s, 3H).

^{13}C NMR (101 MHz, $CDCl_3$) δ 200.9, 140.3, 139.4, 132.4, 129.5, 128.9, 128.2, 30.9, 21.3.

IR(ATR) 3020, 1689, 1594, 1511, 1352, 1182, 1160, 824 702 cm^{-1}.

HRMS (ESI) *m/z*: $[M+Na]^+$ Calcd for $C_{11}H_{12}ONa$ 183.0780; Found. 183.0779.

(*Z*)-4-(4-Methoxyphenyl)but-3-en-2-one (1c)

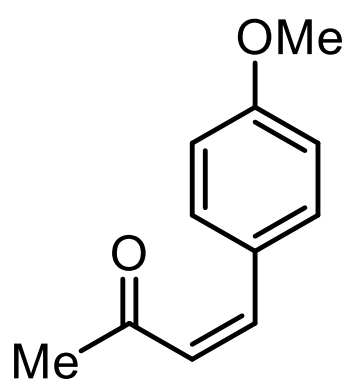

1c

73.9 mg, 42% yield, yellow oil.

^{1}H NMR (400 MHz, $CDCl_3$) δ 7.63-7.60 (m, 2H), 6.89-6.85 (m, 2H), 6.76 (d, *J* = 12.8 Hz, 1H), 6.11 (d, *J* = 12.8 Hz, 1H), 3.83 (s, 3H), 2.21 (s, 3H).

^{13}C NMR (101 MHz, $CDCl_3$) δ 200.3, 160.5, 140.6, 131.9, 127.7, 126.4, 113.5, 55.2, 31.2.

IR(ATR) 3006, 1683, 1587, 1509, 1253, 1158, 1027, 836, 696 cm^{-1}.
HRMS (ESI) *m/z*: $[M+Na]^+$ Calcd for $C_{11}H_{12}O_2Na$ 199.0730; Found. 199.0727.

(*Z*)-4-(4-(Trifluoromethyl)phenyl)but-3-en-2-one (1d)

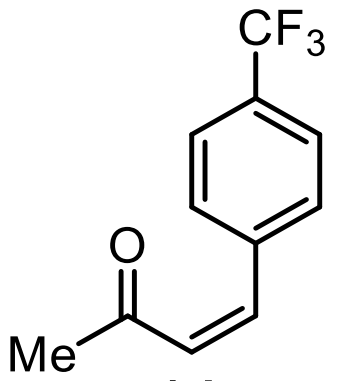

1d

83.9 mg, 39% yield, yellow oil.
^{1}H NMR (400 MHz, $CDCl_3$) δ 7.60 (s, 4H), 6.85 (d, J = 12.8 Hz, 1H), 6.31 (d, J = 12.8 Hz, 1H), 2.20 (s, 3H).
^{13}C NMR (101 MHz, $CDCl_3$) δ 199.9, 138.7, 138.2, 130.6 (q, J_{F-C} = 32.7 Hz), 130.5, 129.5, 125.1 (q, J_{F-C} = 3.9 Hz), 123.9 (q, J_{F-C} = 273.5 Hz), 31.1.
^{19}F NMR (376 MHz, C_6D_6) δ –62.7.
IR(ATR) 3012, 1698, 1615, 1321, 1110, 1065, 1017, 842, 731 cm^{-1}.
HRMS (EI) *m/z*: $[M]^+$ Calcd for $C_{11}H_9F_3O$ 214.0600; Found. 214.0605.

(*Z*)-4-(4-Fluorophenyl)but-3-en-2-one (1e)

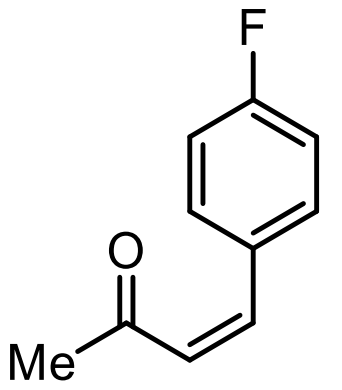

1e

66.6 mg, 41% yield, yellow oil.
^{1}H NMR (400 MHz, $CDCl_3$) δ 7.61-7.56 (m, 2H), 7.06-7.01 (m, 2H), 6.78 (d, J = 12.8 Hz, 1H), 6.19 (d, J = 12.8 Hz, 1H), 2.20 (s, 3H).
^{13}C NMR (101 MHz, $CDCl_3$) δ 200.1, 163.1 (d, J_{F-C} = 251.4 Hz), 139.2, 131.8 (d, J_{F-C} = 7.8 Hz), 131.2 (d, J_{F-C} = 3.8 Hz), 128.2, 115.2 (d, J_{F-C} = 22.1 Hz), 31.1.
IR(ATR) 3003, 1691, 1669, 1598, 1506, 1355, 1227, 1159, 972, 840, 819, 702 cm^{-1}.
HRMS (ESI) *m/z*: $[M+H]^+$ Calcd for $C_{10}H_{10}OF$ 165.0710; Found. 165.0710.

(*Z*)-4-(4-Chlorophenyl)but-3-en-2-one (1f)

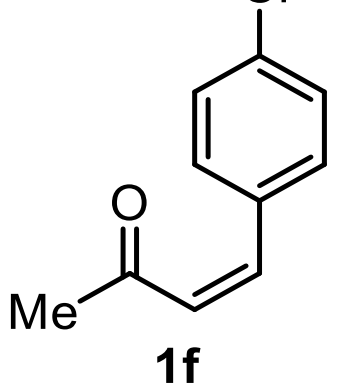

1f

110.3 mg, 60% yield, yellow oil.

^{1}H NMR (400 MHz, $CDCl_3$) δ 7.51-7.48 (m, 2H), 7.33-7.29 (m, 2H), 6.76 (d, *J* = 12.8 Hz, 1H), 6.21 (d, *J* = 12.8 Hz, 1H), 2.19 (s, 3H).

^{13}C NMR (101 MHz, $CDCl_3$) δ 200.0, 138.9, 135.0, 133.5, 130.9, 129.0, 128.3, 31.1.

IR(ATR) 3002, 1690, 1590, 1489, 1354, 1182, 1164, 1090, 1014, 834, 774 cm^{-1}.

HRMS (ESI) *m/z*: $[M+Na]^+$ Calcd for $C_{10}H_9OClNa$ 203.0234; Found. 203.0232.

(*Z*)-4-(*m*-Tolyl)but-3-en-2-one (1h)

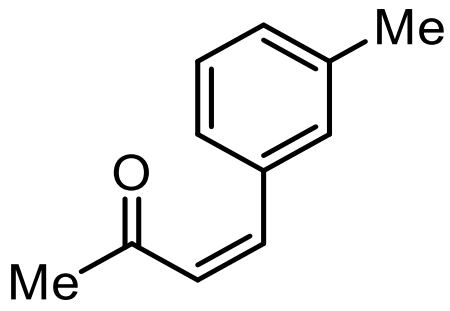

1h

88.8 mg, 56% yield, yellow oil.

^{1}H NMR (400 MHz, $CDCl_3$) δ 7.28-7.22 (m, 3H), 7.16 (d, *J* = 6.8 Hz, 1H), 6.89 (d, *J* = 12.8 Hz, 1H), 6.15 (d, *J* = 12.8 Hz, 1H), 2.35 (s, 3H), 2.14 (s, 3H).

^{13}C NMR (101 MHz, $CDCl_3$) δ 201.2, 140.2, 137.9, 135.3, 129.91, 129.85, 129.2, 128.2, 126.4, 30.8, 21.3.

IR(ATR) 3017, 1691, 1598, 1577, 1353, 1172, 973, 798, 689 cm^{-1}.

HRMS (ESI) *m/z*: $[M+H]^+$ Calcd for $C_{11}H_{13}O$ 161.0961; Found. 161.0960.

(*Z*)-4-(Naphthalen-2-yl)but-3-en-2-one (1j)

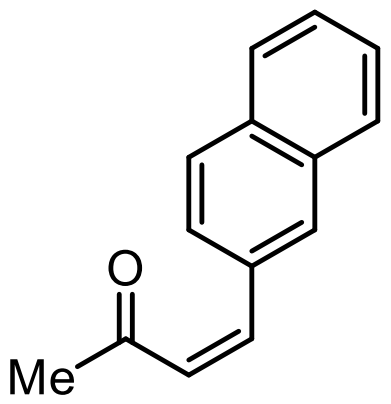

1j

125.8 mg, 64% yield, yellow solid.

^{1}H NMR (400 MHz, $CDCl_3$) δ 7.94 (s, 1H), 7.82-7.77 (m, 3H), 7.58 (dd, *J* = 8.7, 1.8 Hz, 1H), 7.50-7.44 (m, 2H), 7.00 (d, *J* = 13.2 Hz, 1H), 6.23 (d, *J* = 13.2 Hz, 1H), 2.16 (s, 3H).

^{13}C NMR (101 MHz, $CDCl_3$) δ 200.9, 140.1, 133.3, 132.8, 132.7, 129.5, 129.2, 128.3, 127.7, 127.5, 126.8, 126.5, 126.3, 30.9.

IR(ATR) 3056, 1668, 1593, 1366, 1246, 1178, 1158, 973, 827, 742 cm^{-1}.

HRMS (ESI) *m/z*: $[M+Na]^+$ Calcd for $C_{14}H_{12}ONa$ 219.0780; Found. 219.0778.

melting point 57-60 ℃

(*Z*)-1-Phenylpent-1-en-3-one (1l)

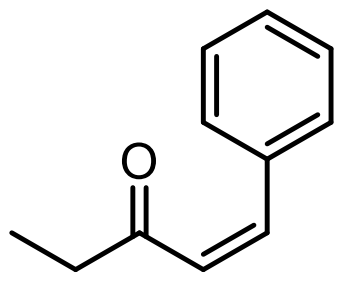

1l

127.6 mg, 53% yield, yellow oil.

^{1}H NMR (400 MHz, $CDCl_3$) δ 7.53-7.49 (m, 2H), 7.35-7.32 (m, 3H), 6.82 (d, J = 12.8 Hz, 1H), 6.18 (d, J = 12.8 Hz, 1H), 2.47 (q, J = 7.6 Hz, 2H), 1.06 (t, J = 7.6 Hz, 3H).

^{13}C NMR (101 MHz, $CDCl_3$) δ 203.9, 139.5, 135.4, 129.4, 129.1, 128.5, 128.2, 36.7, 8.1.

IR(ATR) 2976, 1690, 1605, 1493, 1114, 1036, 785, 690 cm^{-1}.

HRMS (ESI) *m/z*: $[M+Na]^+$ Calcd for $C_{11}H_{12}ONa$ 183.0780; Found. 183.0778.

(*Z*)-4-Methyl-1-phenylpent-1-en-3-one (1m)

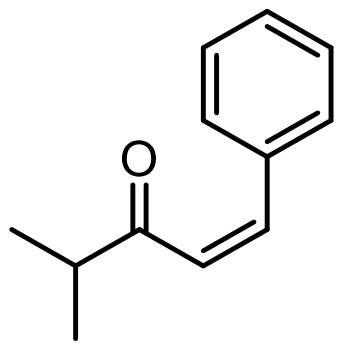

1m

138.8 mg, 53% yield, yellow oil.

^{1}H NMR (400 MHz, $CDCl_3$) δ 7.54-7.50 (m, 2H), 7.34-7.31 (m, 3H), 6.83 (d, J = 12.8 Hz, 1H), 6.25 (d, J = 12.8 Hz, 1H), 2.68 (hep, J = 6.8 Hz, 1H), 1.11 (d, J = 6.8 Hz, 6H).

^{13}C NMR (101 MHz, $CDCl_3$) δ 206.9, 140.0, 135.3, 129.5, 129.1, 128.2, 127.5, 41.1, 18.3.

IR(ATR) 2969, 1687, 1602, 1465, 1041, 805, 765, 696 cm^{-1}.

HRMS (ESI) *m/z*: $[M+Na]^+$ Calcd for $C_{12}H_{14}ONa$ 197.0937; Found. 197.0934.

Synthesis of methyl 2-isocyanopentanoate (2b)

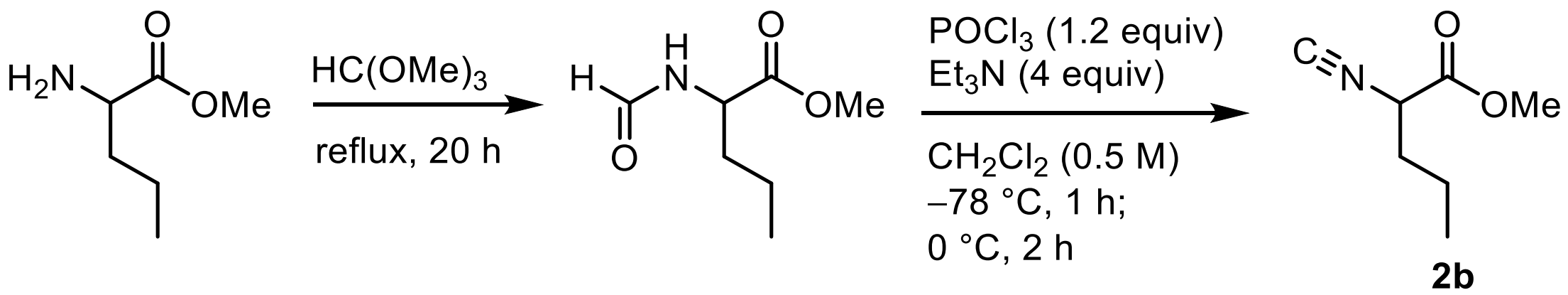

A mixture of methyl 2-aminopentanoate (262 mg, 2.0 mmol) and trimethyl orthoformate (8 mL, 0.25 M) was refluxed for 20 h. Evaporation of the reaction mixture gave the crude product of methyl 2-formamidopentanoate as brown oil.

To the crude product were added CH_2Cl_2 (8 mL) and Et_3N (0.66 mL, 4.8 mmol, 2.4 equiv), and $POCl_3$ was then slowly added (0.2 mL, 2.2 mmol, 1.1 equiv) at 0 °C. After stirring the mixture at 0 °C for 1 hour and at room temperature for 3 hours, the mixture was quenched with saturated $NaHCO_3$ aqueous solution (10 ml) at 0 °C. The organic phase was separated, and the aqueous phase was

extracted with CH_2Cl_2 (10 mL, 3 times). The combined organic phases were dried over $MgSO_4$ and evaporated. Chromatography of the residue on silica gel (EtOAc/hexane 5% to 15%) gave methyl 2-isocyanopentanoate (220 mg) in 78% yield in 2 steps as pale yellow oil.

^{1}H NMR (400 MHz, $CDCl_3$) δ 4.29 (t, J = 6.8 Hz, 1H), 3.83 (s, 3H), 1.94-1.87 (m, 2H), 1.60-1.50 (m, 2H), 0.99 (t, J = 7.6 Hz, 3H).

^{13}C NMR (101 MHz, $CDCl_3$) δ 167.3, 159.8, 56.3, 53.3, 34.6, 18.5, 13.1.

IR(ATR) 2965, 2147, 1750, 1439, 1232, 1206, 1130, 999 cm^{-1}.

HRMS (ESI) *m/z*: $[M+Na]^+$ Calcd for $C_7H_{11}NO_2Na$ 164.0682; Found. 164.0681.

(4*R*,5*S*)-3-Acetyl-5-methyl-4-phenyl-5-methoxycarbonyl-2-pyrroline (3aa)

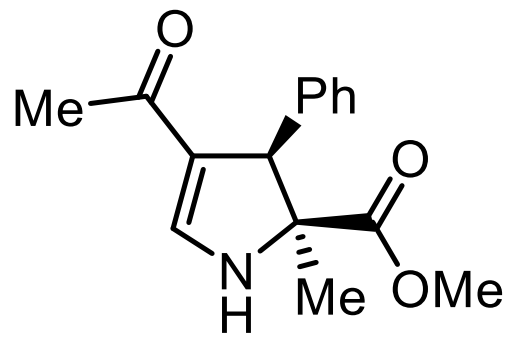

3aa

NMR yield: 70% yield, dr 92:8

Isolated yield: 41.0 mg, 63% yield, dr >20:1, 96% ee. Colorless oil.

^{1}H NMR (400 MHz, $CDCl_3$) δ 7.39 (d, J = 3.2 Hz, 1H), 7.24-7.20 (m, 2H), 7.17-7.12 (m, 3H), 5.16 (brs, 1H), 4.05 (s, 1H), 3.13 (s, 3H), 2.11 (s, 3H), 1.60 (s, 3H).

^{13}C NMR (101 MHz, $CDCl_3$) δ 191.3, 172.4, 147.0, 139.5, 127.9, 127.8, 127.0, 117.4, 73.9, 56.9, 51.7, 26.7, 25.8.

IR(ATR) 3219, 2969, 1739, 1559, 1440, 1280, 1126, 951, 879, 698 cm^{-1}.

HRMS (ESI) *m/z*: $[M+H]^+$ Calcd for $C_{15}H_{18}NO_3$ 260.1281; Found. 260.1276.

$[\alpha]_D$ $^{20.8}$ –122.1 (*c* 0.80, $CHCl_3$).

The ee value was determined by chiral HPLC analysis: CHIRALPACK® IE-3 column, 4.6 mm × 250, Daicel Chemical Industries, hexane/2-propanol = 75:25, 1.0 mL/min, 40 °C, 220 nm UV detector, retention time = 10.3 min (major), 11.3 min (minor).

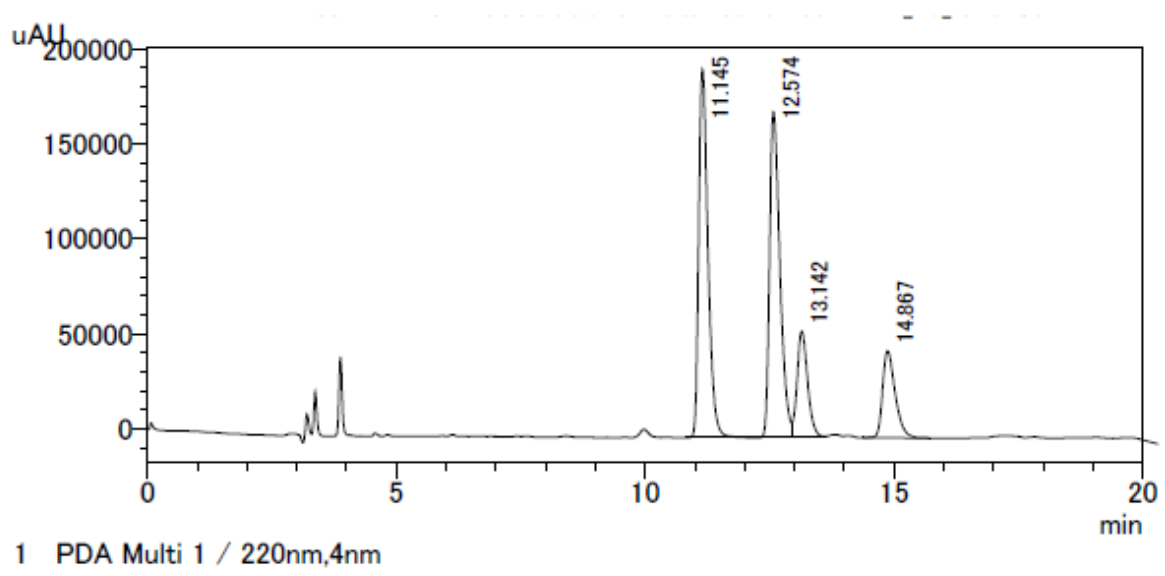

Peak Table

PDA Ch1 220nm

Peak#	Ret. Time	Area	Area%
1	11.145	2509381	37.853
2	12.574	2481683	37.435
3	13.142	830710	12.531
4	14.867	807520	12.181
‡Œv		6629294	100.000

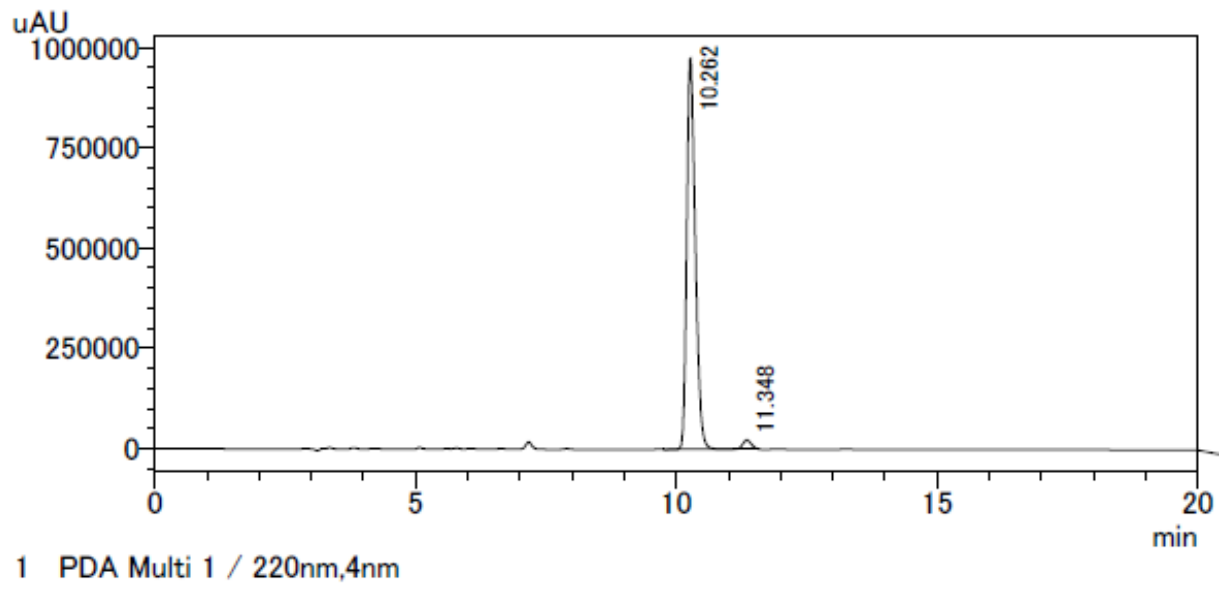

Peak Table

PDA Ch1 220nm

Peak#	Ret. Time	Area	Area%
1	10.262	11097260	97.751
2	11.348	255284	2.249
‡Œv		11352543	100.000

(4*R*,5*S*)-3-Acetyl-5-methyl-4-(*p*-tolyl)-5-methoxycarbonyl-2-pyrroline (3ba)

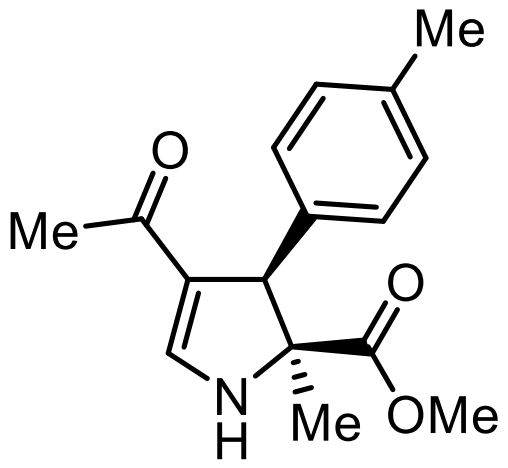

3ba

NMR yield: 85% yield, dr 90:10

Isolated yield: 37.5 mg, 55% yield, dr >20:1, 93% ee. Colorless oil.

^{1}H NMR (400 MHz, $CDCl_3$)δ 7.38 (d, *J* = 3.2 Hz, 1H), 7.02 (s, 4H), 5.25 (brs, 1H), 4.01 (s, 1H), 3.16 (s, 3H), 2.26 (s, 3H), 2.09 (s, 3H), 1.58 (s, 3H).

^{13}C NMR (101 MHz, $CDCl_3$) δ 191.5, 172.5, 146.8, 136.4, 128.6, 127.6, 117.6, 73.9, 56.6, 51.7, 26.7, 25.8, 21.0.

IR(ATR) 3203, 2950, 1738, 1558, 1435, 1279, 1172, 1126, 751, 729 cm^{-1}.

HRMS (ESI) *m/z*: $[M+H]^+$ Calcd for $C_{16}H_{20}NO_3$ 274.1438; Found. 274.1433.

$[\alpha]_D$ $^{20.5}$ –101.7 (*c* 1.71, $CHCl_3$).

The ee value was determined by chiral HPLC analysis: CHIRALPACK® IE-3 column, 4.6 mm × 250 mm, Daicel Chemical Industries, hexane/2-propanol = 75:25, 1.0 mL/min, 40 °C, 220 nm UV detector, retention time = 11.7 min (major), 13.0 min (minor).

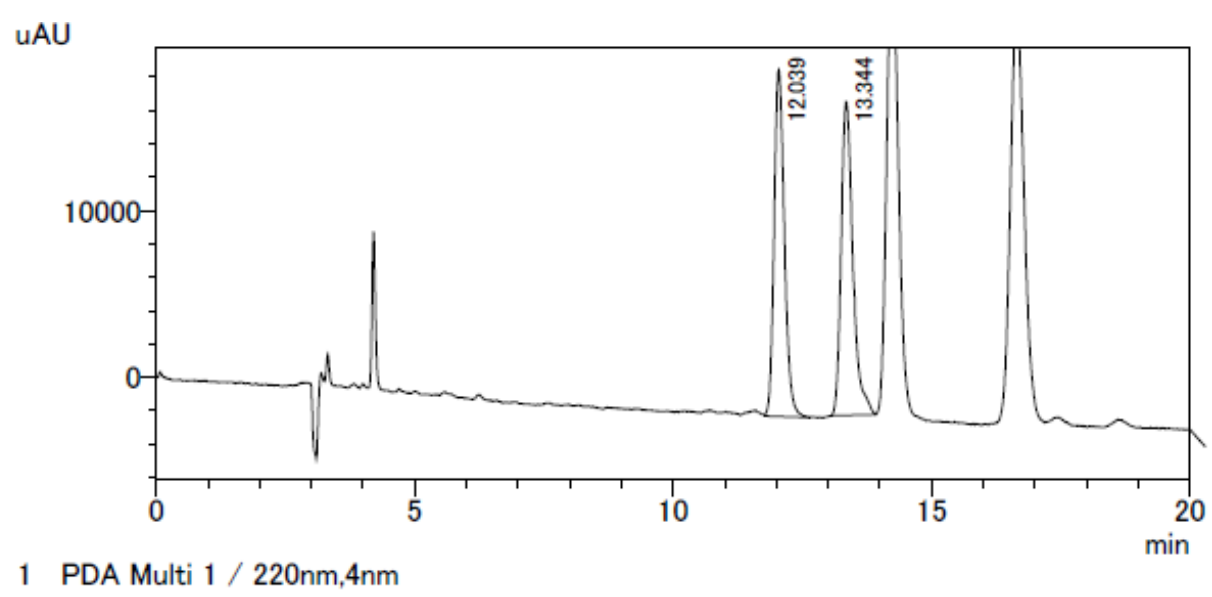

Peak Table

PDA Ch1 220nm

Peak#	Ret. Time	Area	Area%
1	12.039	287128	49.169
2	13.344	296831	50.831
‡Œv		583959	100.000

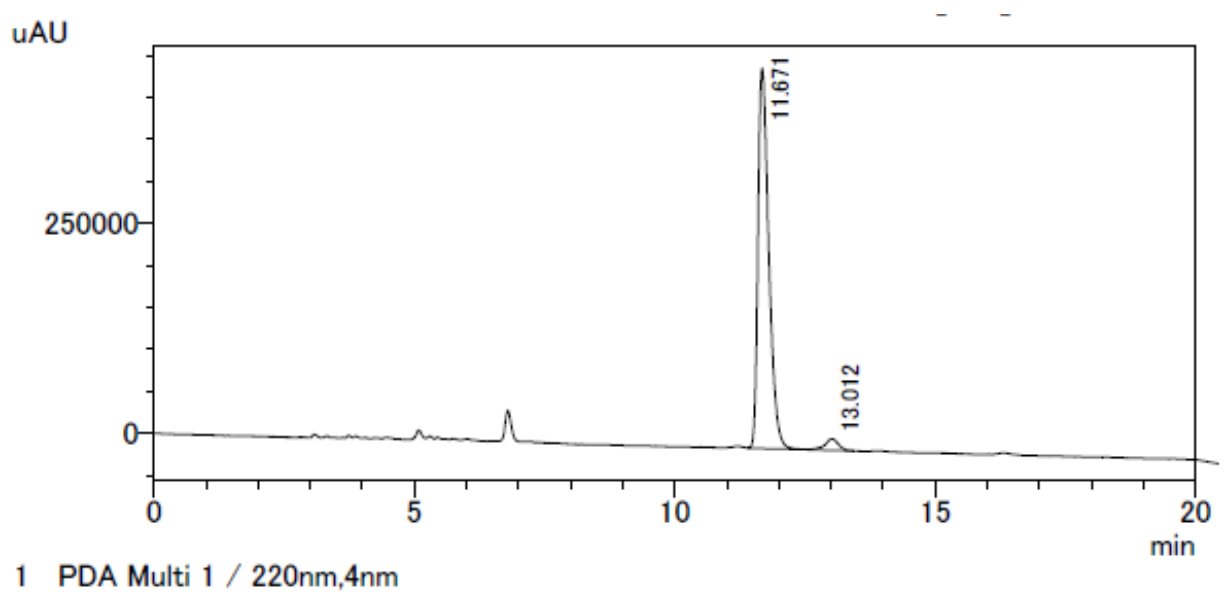

Peak Table

PDA Ch1 220nm

Peak#	Ret. Time	Area	Area%
1	11.671	6516174	96.314
2	13.012	249375	3.686
‡Œv		6765549	100.000

(4*R*,5*S*)-3-Acetyl-5-methyl-4-(4-methoxyphenyl)-5-methoxycarbonyl-2-pyrroline (3ca)

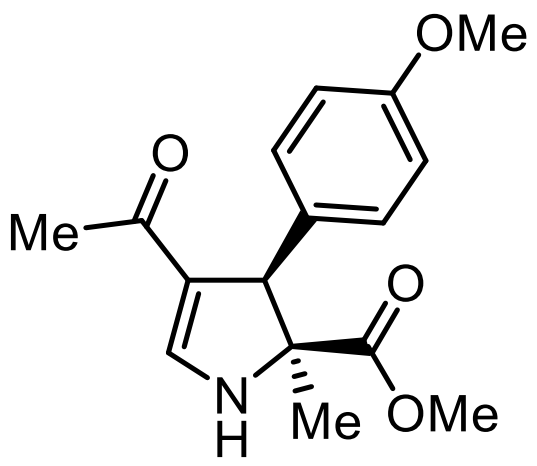

3ca

NMR yield: 85% yield, dr 89:11

Isolated yield: 36.0 mg, 50% yield, dr 20:1, 95% ee. Colorless oil.

¹H NMR (400 MHz, $CDCl_3$) δ 7.38 (d, *J* = 3.2 Hz, 1H), 7.07-7.04 (m, 2H), 6.77-6.74 (m, 2H), 5.18 (brs, 1H), 4.01 (s, 1H), 3.74 (s, 3H), 3.19 (s, 3H), 2.10 (s, 3H), 1.58 (s, 3H).

¹³C NMR (101 MHz, $CDCl_3$) δ 191.5, 172.5, 158.5, 146.6, 131.6, 128.8, 117.6, 113.3, 73.8, 56.2, 55.1, 51.8, 26.7, 25.8.

IR(ATR) 3219, 2951, 1737, 1559, 1510, 1439, 1242, 1175, 1126, 1033, 842, 728 cm^{-1}.

HRMS (ESI) *m/z*: $[M+H]^+$ Calcd for $C_{16}H_{20}NO_4$ 290.1387; Found. 290.1382.

$[\alpha]_D$ ²⁰·⁴ –148.8 (*c* 0.34, $CHCl_3$).

The ee value was determined by chiral HPLC analysis: CHIRALPACK® IE-3 column, 4.6 mm × 250 mm, Daicel Chemical Industries, hexane/2-propanol = 75:25, 1.0 mL/min, 40 °C, 220 nm UV detector, retention time = 14.2 min (major), 16.5 min (minor).

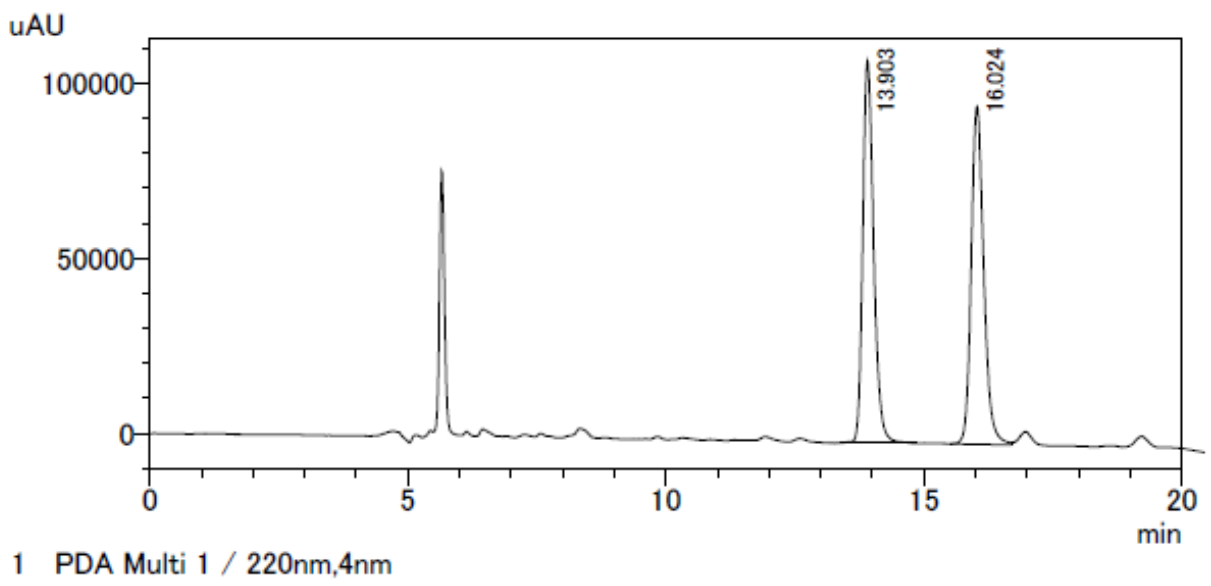

Peak Table

PDA Ch1 220nm

Peak#	Ret. Time	Area	Area%
1	13.903	1607522	49.600
2	16.024	1633435	50.400
‡Œv		3240957	100.000

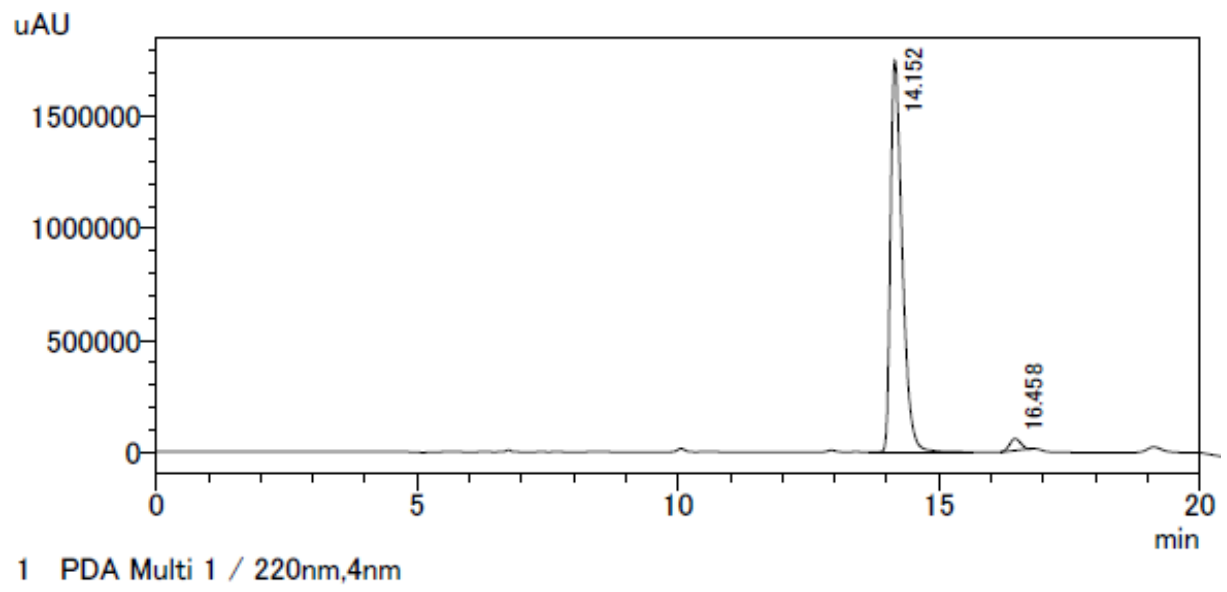

Peak Table

PDA Ch1 220nm

Peak#	Ret. Time	Area	Area%
1	14.152	27637412	97.309
2	16.458	764397	2.691
‡Œv		28401809	100.000

(4*R*,5*S*)-3-Acetyl-5-methyl-4-(4-trifluoromethylphenyl)-5-methoxycarbonyl-2-pyrroline (3da)

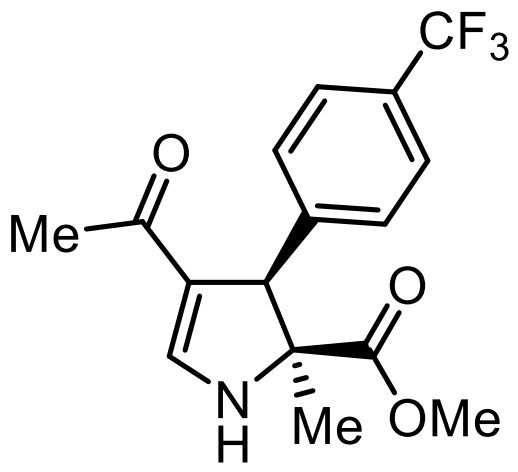

3da

NMR yield: 83% yield, dr 92:8

Isolated yield: 48.0 mg, 59% yield, dr >20:1, 90% ee. Colorless oil.

¹H NMR (400 MHz, $CDCl_3$) δ 7.48 (d, *J* = 8.4 Hz, 2H), 7.40 (d, *J* = 2.8 Hz, 1H), 7.26 (d, *J* = 8.4 Hz, 2H), 5.33 (brs, 1H), 4.10 (s, 1H), 3.14 (s, 3H), 2.13 (s, 3H), 1.61 (s, 3H).

¹³C NMR (101 MHz, $CDCl_3$) δ 191.1, 172.1, 147.0, 144.0, 129.1 (q, $J_{F\text{-}C}$ = 32.4 Hz), 128.1, 124.8 (q, $J_{F\text{-}C}$ = 3.57 Hz), 123.1 (q, $J_{F\text{-}C}$ = 273.5 Hz) 117.4, 73.8, 56.4, 51.8, 26.9, 25.6.

¹⁹F NMR (376 MHz, C_6D_6) δ –62.3.

IR(ATR) 3201, 2953, 1740, 1560, 1439, 1323, 1162, 1108, 1065, 861, 731 cm^{-1}.

HRMS (ESI) *m/z*: $[M+H]^+$ Calcd for $C_{16}H_{17}F_3NO_3$ 328.1155; Found. 328.1151.

$[\alpha]_D$ ²⁰·⁴ –95.3 (*c* 2.69, $CHCl_3$).

The ee value was determined by chiral HPLC analysis: CHIRALPACK® IE-3 column, 4.6 mm × 250

mm, Daicel Chemical Industries, hexane/2-propanol = 75:25, 1.0 mL/min, 40 °C, 220 nm UV detector, retention time = 5.8 min (major), 6.4 min (minor).

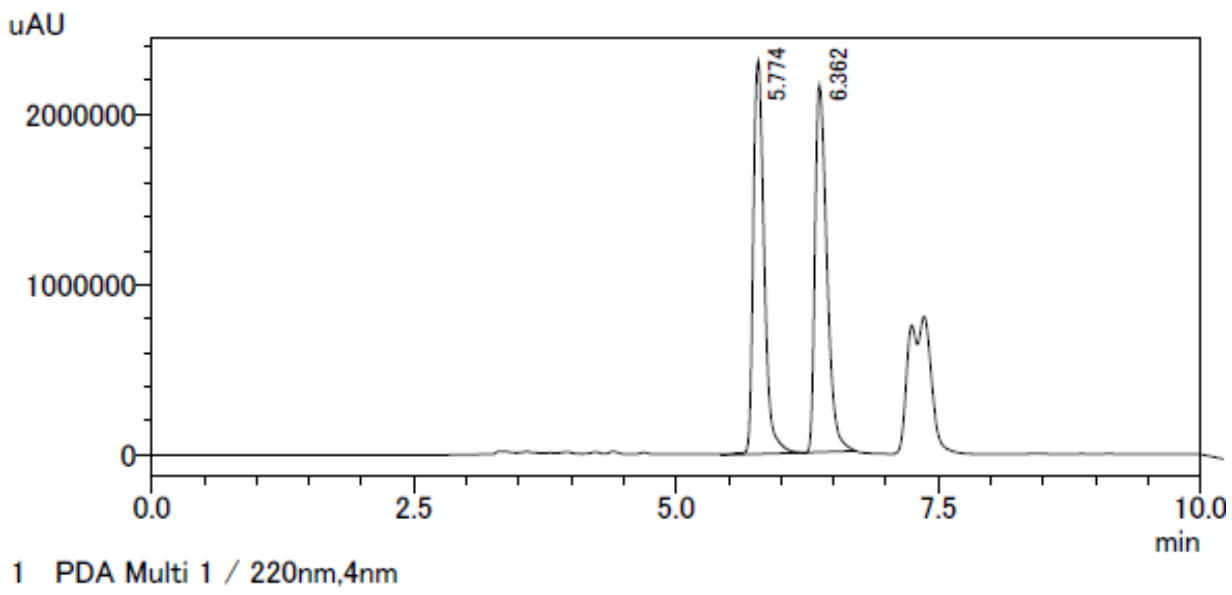

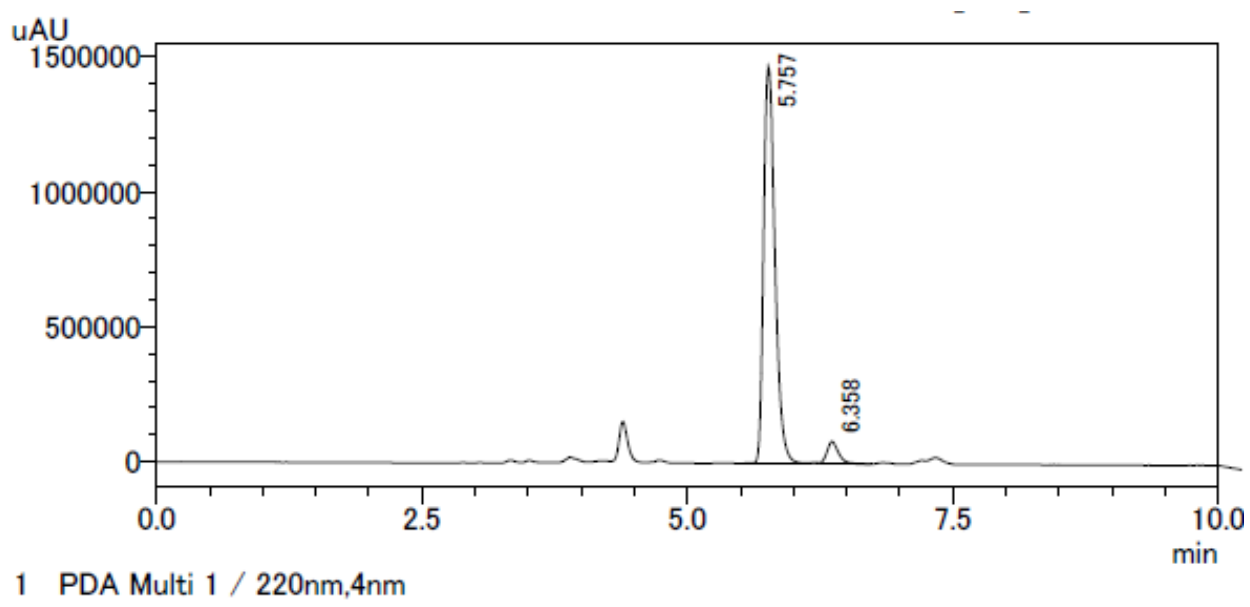

Peak Table

PDA Ch1 220nm

Peak#	Ret. Time	Area	Area%
1	5.774	16801643	49.560
2	6.362	17099674	50.440
‡Œv		33901317	100.000

Peak Table

PDA Ch1 220nm

Peak#	Ret. Time	Area	Area%
1	5.757	10990729	94.848
2	6.358	597057	5.152
‡Œv		11587786	100.000

(4*R*,5*S*)-3-Acetyl-5-methyl-4-(4-fluorophenyl)-5-methoxycarbonyl-2-pyrroline (3ea)

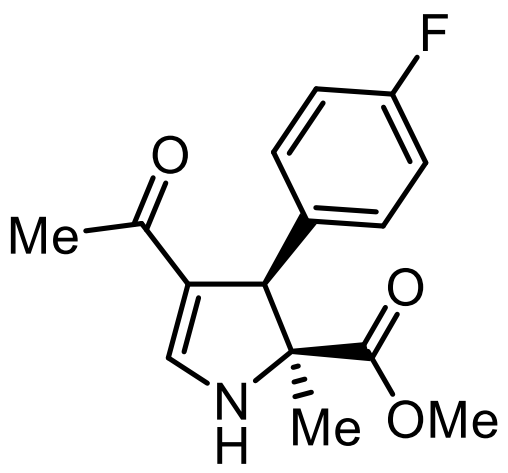

3ea

NMR yield: 68% yield, dr 90:10

Isolated yield: 49.0 mg, 57% yield, dr >20:1, 93% ee. Yellow oil.

^{1}**H NMR** (400 MHz, $CDCl_3$) δ 7.39 (d, J = 2.8 Hz, 1H), 7.13-7.08 (m, 2H), 6.94-6.88 (m, 2H), 5.26 (brs, 1H), 4.04 (s, 1H), 3.19 (s, 3H), 2.12 (s, 3H), 1.59 (s, 3H).

^{13}**C NMR** (101 MHz, $CDCl_3$) δ 191.3, 172.3, 161.8 (d, $J_{F\text{-}C}$ = 245.5 Hz), 146.8, 135.4 (d, $J_{F\text{-}C}$ = 2.9 Hz), 129.3 (d, $J_{F\text{-}C}$ = 7.7 Hz), 117.6, 114.7 (d, $J_{F\text{-}C}$ = 21.2 Hz), 73.7, 56.1, 51.8, 26.8, 25.7.

^{19}**F NMR** (376 MHz, C_6D_6) δ –115.7.

IR(ATR) 3199, 2952, 1737, 1559, 1507, 1436, 1280, 1220, 1127, 847, 753, 729 cm^{-1}.

HRMS (ESI) *m/z*: $[M+H]^+$ Calcd for $C_{15}H_{17}FNO_3$ 278.1187; Found. 278.1182.

$[\alpha]_D^{20.4}$ –88.2 (*c* 1.41, $CHCl_3$).

The ee value was determined by chiral HPLC analysis: CHIRALPACK® IE-3 column, 4.6 mm × 250 mm, Daicel Chemical Industries, hexane/2-propanol = 75:25, 1.0 mL/min, 40 °C, 220 nm UV detector, retention time = 8.3 min (major), 9.5 min (minor).

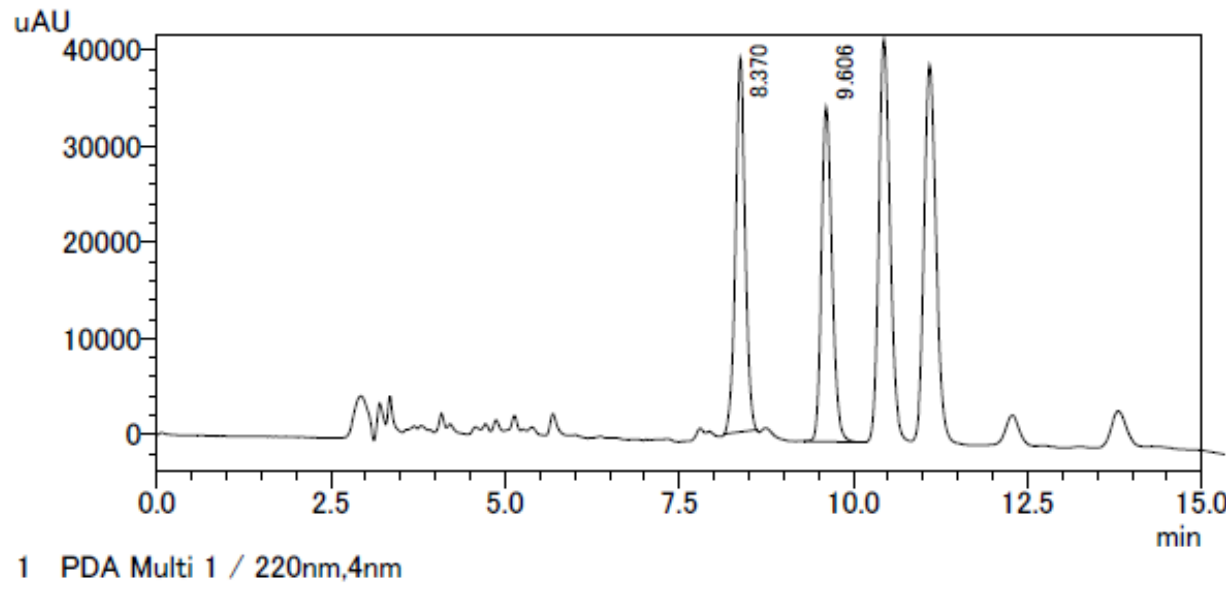

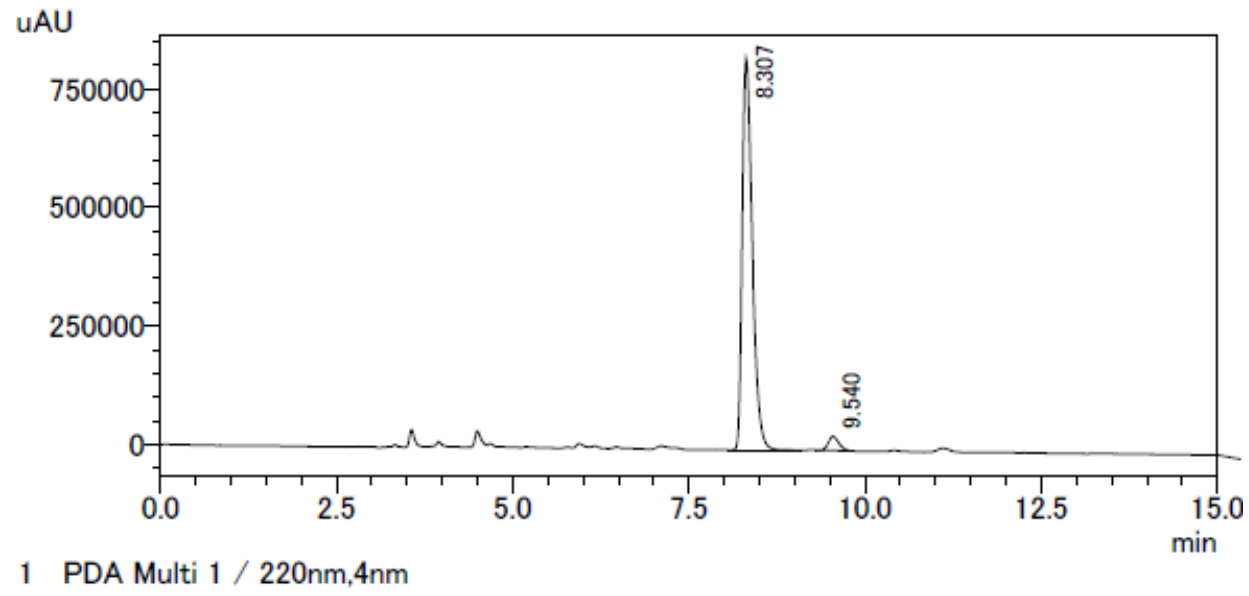

Peak Table

PDA Ch1 220nm

Peak#	Ret. Time	Area	Area%
1	8.370	377016	50.606
2	9.606	367980	49.394
‡Œv		744996	100.000

Peak Table

PDA Ch1 220nm

Peak#	Ret. Time	Area	Area%
1	8.307	8244080	96.397
2	9.540	308101	3.603
‡Œv		8552181	100.000

(4*R*,5*S*)-3-Acetyl-5-methyl-4-(4-chlorophenyl)-5-methoxycarbonyl-2-pyrroline (3fa)

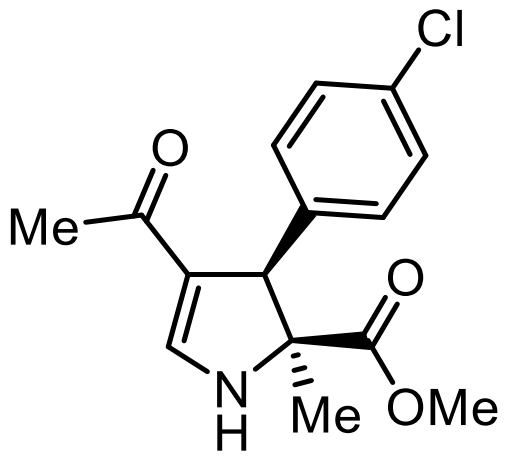

3fa

NMR yield: 85% yield, dr 90:10

Isolated yield: 59.4 mg, 81% yield, dr >20:1, 91% ee. Colorless oil.

^{1}H NMR (400 MHz, $CDCl_3$) δ 7.36 (d, J = 3.6 Hz, 1H), 7.19 (d, J = 8.0 Hz, 2H), 7.07 (d, J = 8.0 Hz, 2H), 5.42 (brs, 1H), 4.02 (s, 1H), 3.20 (s, 3H), 2.11 (s, 3H), 1.57 (s, 3H).

^{13}C NMR (101 MHz, $CDCl_3$) δ 191.1, 172.1, 147.1, 138.3, 132.6, 129.2, 128.0, 117.2, 73.7, 56.1, 51.8, 26.7, 25.6.

IR(ATR) 3214, 2951, 1738, 1558, 1436, 1277, 909, 728 cm^{-1}.

HRMS (ESI) *m/z*: $[M+Na]^+$ Calcd for $C_{15}H_{16}NO_3ClNa$ 316.0711; Found. 316.0706.

$[\alpha]_D^{20.4}$ –55.5 (*c* 0.55, $CHCl_3$).

The ee value was determined by chiral HPLC analysis: CHIRALPACK® IG-3 column, 4.6 mm × 250 mm, Daicel Chemical Industries, hexane/2-propanol = 75:25, 1.0 mL/min, 40 °C, 220 nm UV detector, retention time = 6.0 min (major), 11.9 min (minor).

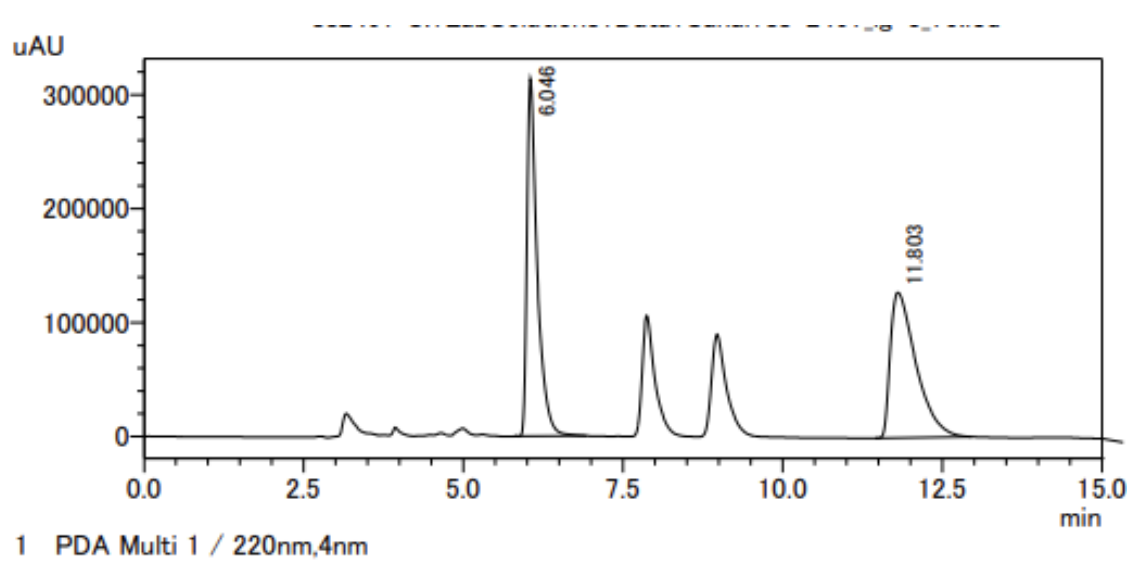

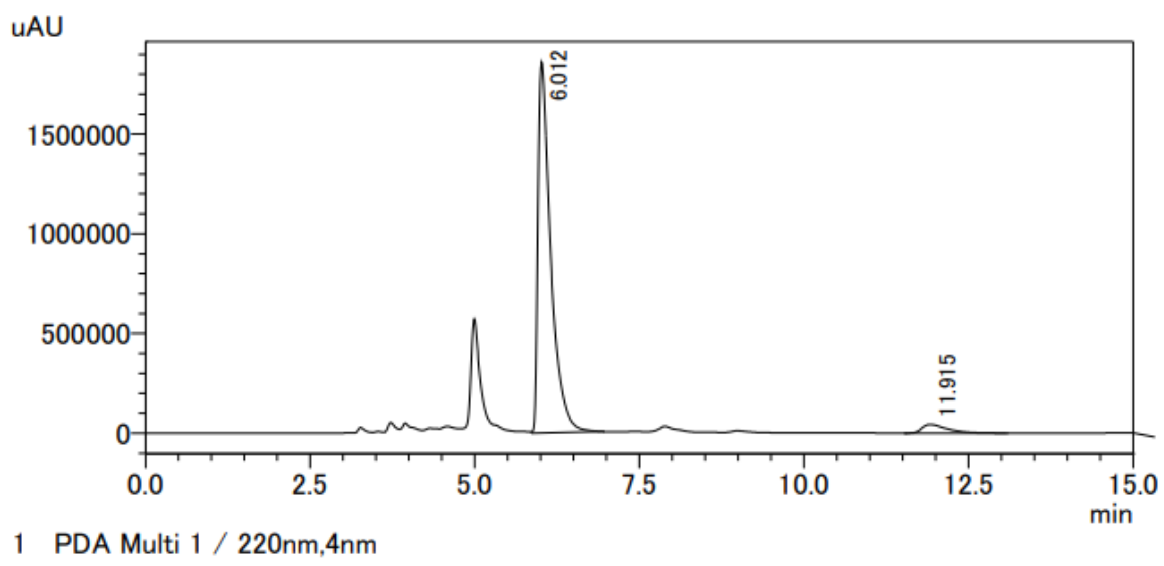

Peak Table

PDA Ch1 220nm

Peak#	Ret. Time	Area	Area%
1	6.046	3506000	49.579
2	11.803	3565610	50.421
‡Œv		7071610	100.000

Peak Table

PDA Ch1 220nm

Peak#	Ret. Time	Area	Area%
1	6.012	24910617	95.640
2	11.915	1135568	4.360
‡Œv		26046184	100.000

(4*R*,5*S*)-3-Acetyl-5-methyl-4-(4-bromophenyl)-5-methoxycarbonyl-2-pyrroline (3ga)

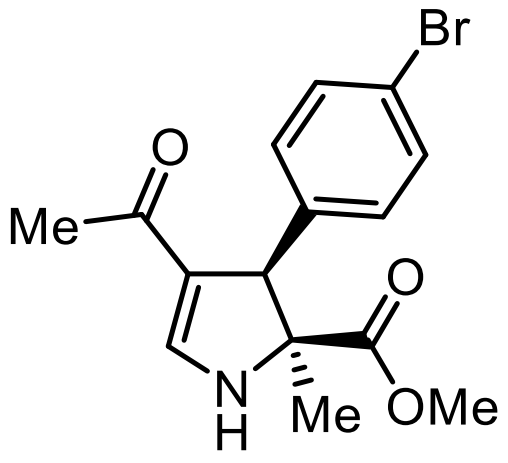

3ga

NMR yield: 70% yield, dr 90:10

Isolated yield: 38.8 mg, 46% yield, dr >20:1, 95% ee. Orange solid.

^{1}H NMR (400 MHz, $CDCl_3$) δ 7.38-7.37 (m, 1H), 7.34 (d, *J* = 8.4 Hz, 2H), 7.02 (d, *J* = 8.4 Hz, 2H), 5.22 (brs, 1H), 4.01 (s, 1H), 3.21 (s, 3H), 2.12 (s, 3H), 1.60 (s, 3H).

^{13}C NMR (101 MHz, $CDCl_3$) δ 191.2, 172.2, 146.8, 138.8, 131.0, 129.5, 120.8, 117.5, 73.7, 56.2, 51.9, 26.8, 25.7.

IR(ATR) 3204, 2951, 1737, 1559, 1486, 1435, 1127, 1011, 910, 751, 729 cm^{-1}.

HRMS (ESI) *m/z*: $[M+H]^+$ Calcd for $C_{15}H_{17}NO_3Br$ 338.0386; Found. 338.0385.

melting point 54.5-56.5 °C

$[\alpha]_D$ $^{22.1}$ –118.1 (*c* 0.56, $CHCl_3$).

The ee value was determined by chiral HPLC analysis: CHIRALPACK® IA-3 column, 4.6 mm × 250 mm, Daicel Chemical Industries, hexane/2-propanol = 80:20, 1.0 mL/min, 40 °C, 220 nm UV detector, retention time = 7.2 min (major), 11.6 min (minor).

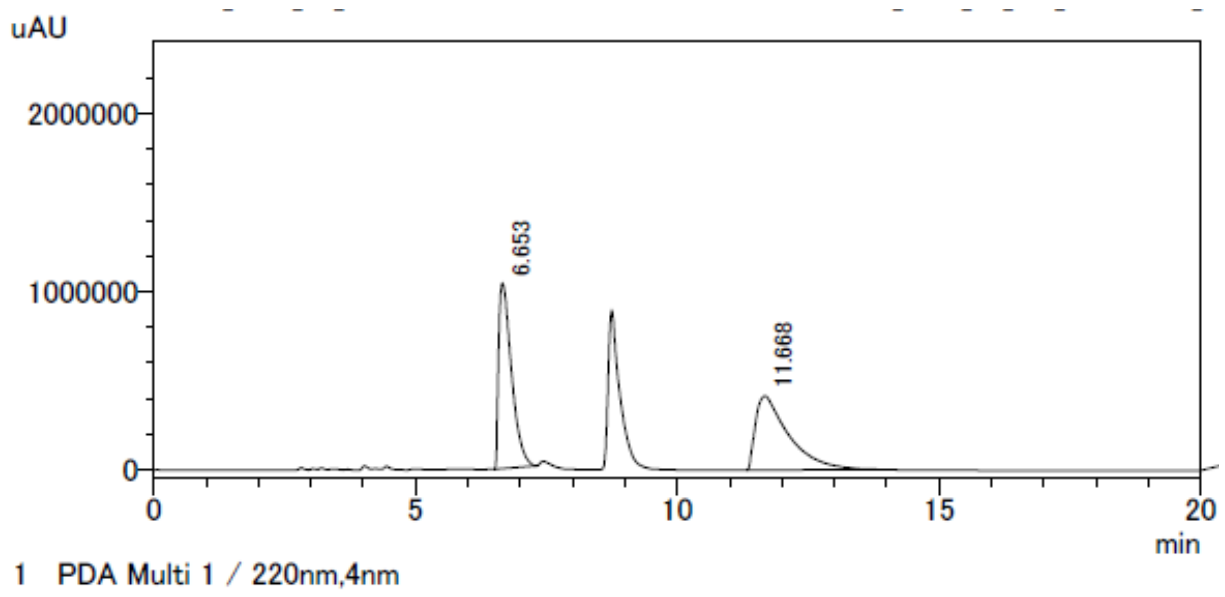

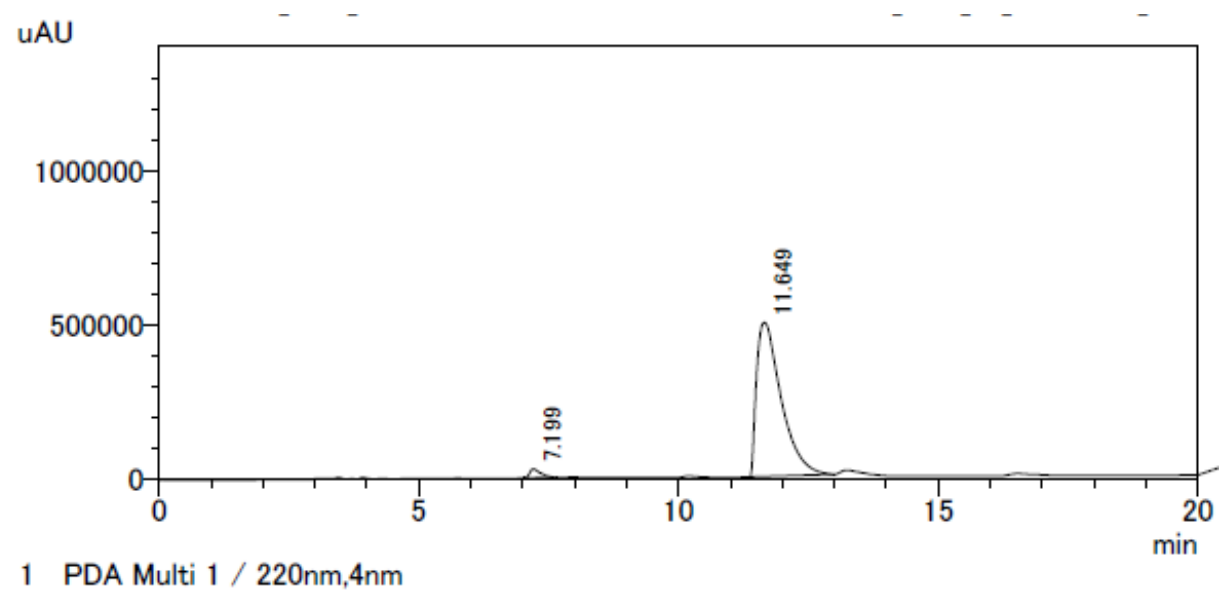

Peak Table

PDA Ch1 220nm

Peak#	Ret. Time	Area	Area%
1	6.653	17818842	49.259
2	11.668	18354834	50.741
‡Œv		36173675	100.000

Peak Table

PDA Ch1 220nm

Peak#	Ret. Time	Area	Area%
1	7.199	426932	2.538
2	11.649	16393110	97.462
‡Œv		16820042	100.000

(4*R*,5*S*)-3-Acetyl-5-methyl-4-(*m*-tolyl)-5-methoxycarbonyl-2-pyrroline (3ha)

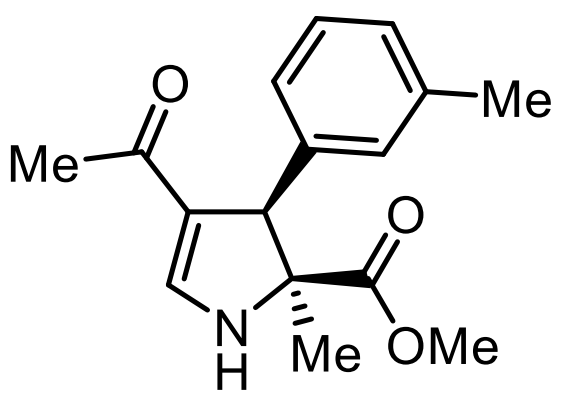

3ha

NMR yield: 55% yield, dr 90:10

Isolated yield: 30.5 mg, 45% yield, dr >20:1, 92% ee. Colorless oil.

^{1}H NMR (400 MHz, $CDCl_3$) δ 7.38 (d, J = 2.8 Hz, 1H), 7.10 (t, J = 7.2 Hz, 1H), 6.97-6.91 (m, 3H), 5.12 (brs, 1H), 4.01 (s, 1H), 3.14 (s, 3H), 2.28 (s, 3H), 2.10 (s, 3H), 1.59 (s, 3H).
^{13}C NMR (101 MHz, $CDCl_3$) δ 191.5, 172.5, 146.6, 139.4, 137.2, 128.6, 127.8, 124.8, 117.7, 73.9, 57.0, 51.7, 26.9, 25.9, 21.3.
IR(ATR) 3159, 2926, 1733, 1556, 1432, 1281, 1188, 1127, 1108, 962, 865 cm^{-1}.
HRMS (ESI) *m/z*: $[M+H]^+$ Calcd for $C_{16}H_{20}NO_3$ 274.1438; Found. 274.1438.
$[α]_D$ $^{19.6}$ –195.6 (*c* 0.80, $CHCl_3$).
The ee value was determined by chiral HPLC analysis: CHIRALPACK® IG-3 column, 4.6 mm × 250 mm, Daicel Chemical Industries, hexane/2-propanol = 75:25, 1.0 mL/min, 40 °C, 220 nm UV detector, retention time = 6.1 min (major), 9.9 min (minor).

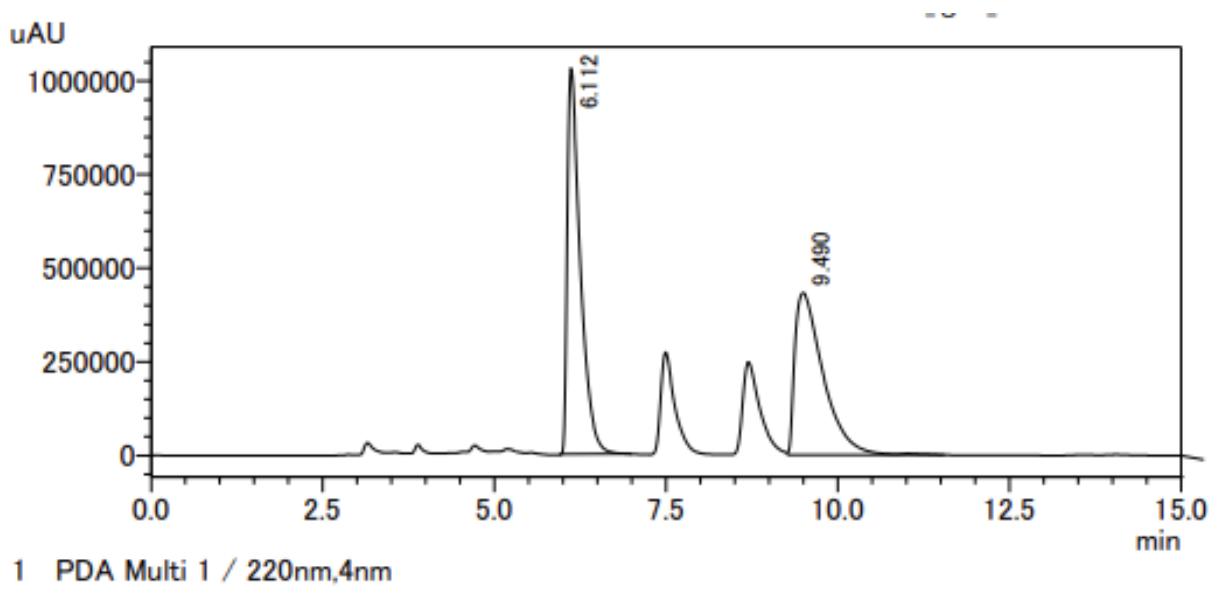

Peak Table

PDA Ch1 220nm

Peak#	Ret. Time	Area	Area%
1	6.112	13613363	52.237
2	9.490	12447523	47.763
‡Œv		26060886	100.000

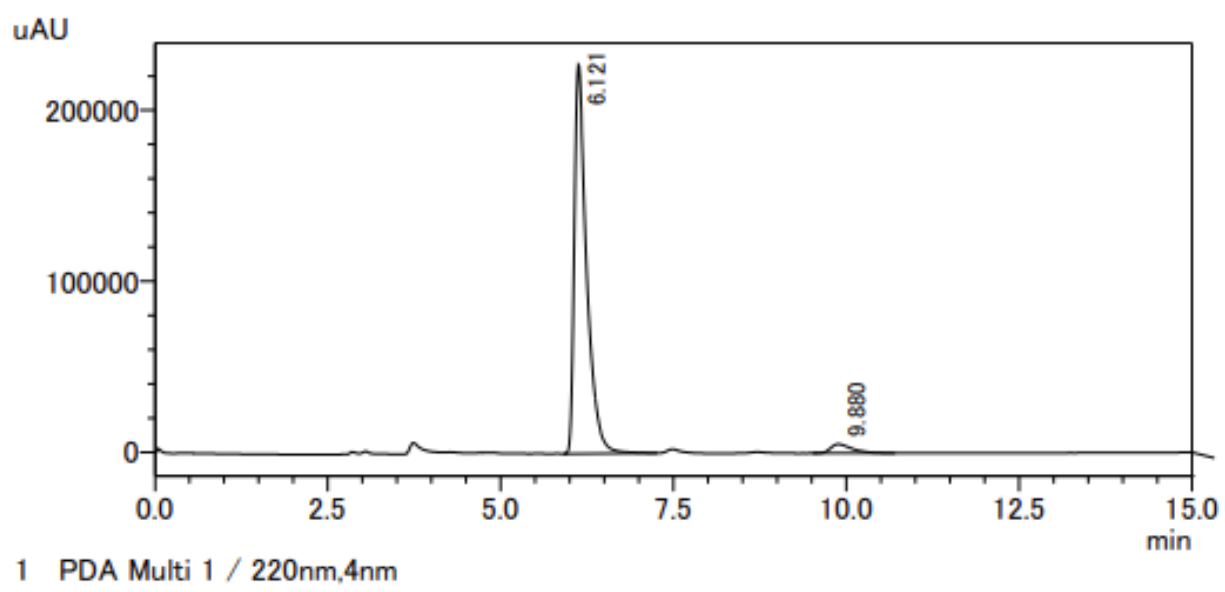

Peak Table

PDA Ch1 220nm

Peak#	Ret. Time	Area	Area%
1	6.121	2825614	96.140
2	9.880	113444	3.860
‡Œv		2939059	100.000

(4*R*,5*S*)-3-Acetyl-5-methyl-4-naphthyl-5-methoxycarbonyl-2-pyrroline (3ja)

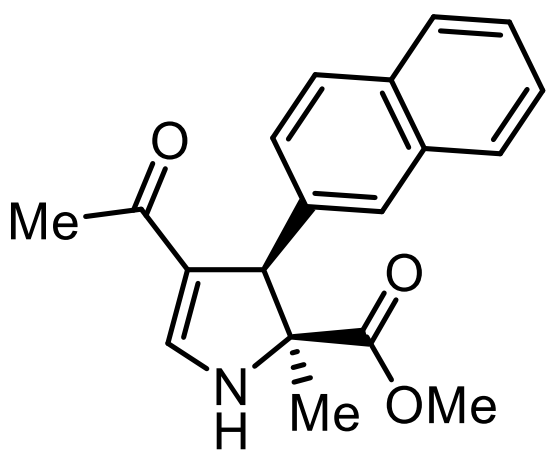

3ja

NMR yield: 77% yield, dr 83:17
Isolated yield: 54.1 mg, 70% yield, dr >20:1, 90% ee. Colorless oil.
^{1}H NMR (400 MHz, $CDCl_3$) δ 7.77-7.73 (m, 2H), 7.70 (d, J = 8.4 Hz, 1H), 7.61 (s, 1H), 7.41-7.37 (m, 3H), 7.29-7.25 (m, 1H), 5.39 (brs, 1H), 4.21 (s, 1H), 2.98 (s, 3H), 2.08 (s, 3H), 1.61 (s, 3H).
^{13}C NMR (101 MHz, $CDCl_3$) δ 191.4, 172.3, 147.0, 137.2, 133.1, 132.6, 127.8, 127.4, 126.6, 126.2, 125.6, 125.4, 117.5, 74.0, 56.9, 51.7, 26.8, 25.8.
IR(ATR) 3219, 2951, 1737, 1558, 1436, 1271, 1171, 1126, 909, 727 cm^{-1}.
HRMS (ESI) *m/z*: $[M+Na]^+$ Calcd for $C_{19}H_{19}NO_3Na$ 332.1257; Found. 332.1253.
$[α]_D$ $^{20.3}$ –197.5 (*c* 1.97, $CHCl_3$).
The ee value was determined by chiral HPLC analysis: CHIRALPACK® IE-3 column, 4.6 mm × 250

mm, Daicel Chemical Industries, hexane/2-propanol = 75:25, 1.0 mL/min, 40 °C, 220 nm UV detector, retention time = 13.1 min (major), 14.4 min (minor).

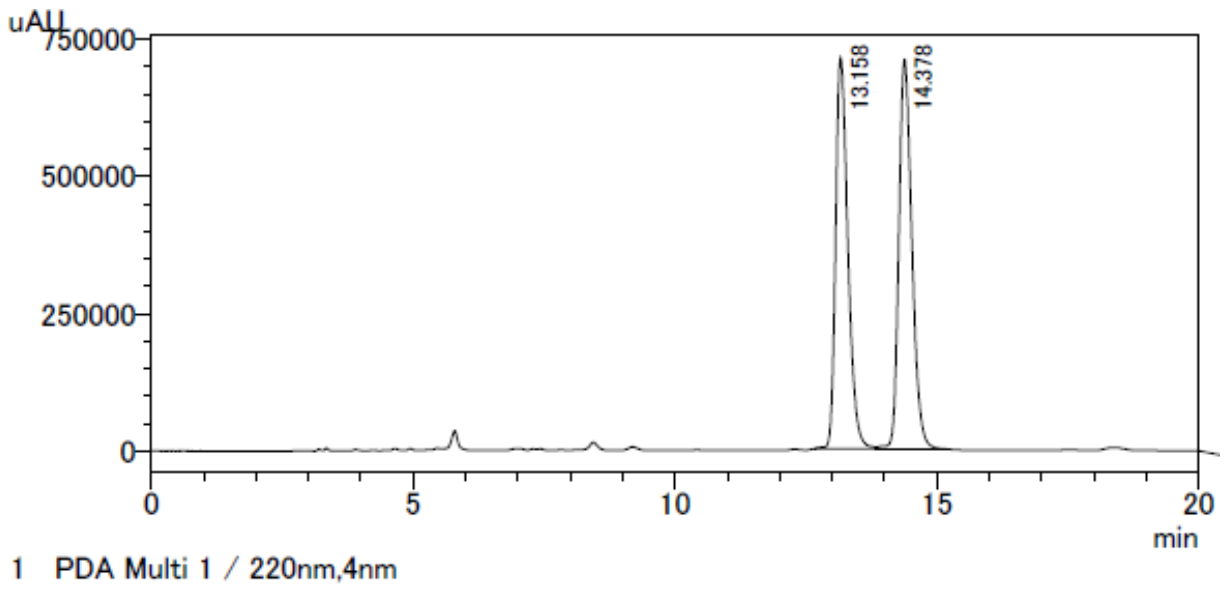

Peak Table

PDA Ch1 220nm

Peak#	Ret. Time	Area	Area%
1	13.158	11624677	48.610
2	14.378	12289625	51.390
‡Œv		23914302	100.000

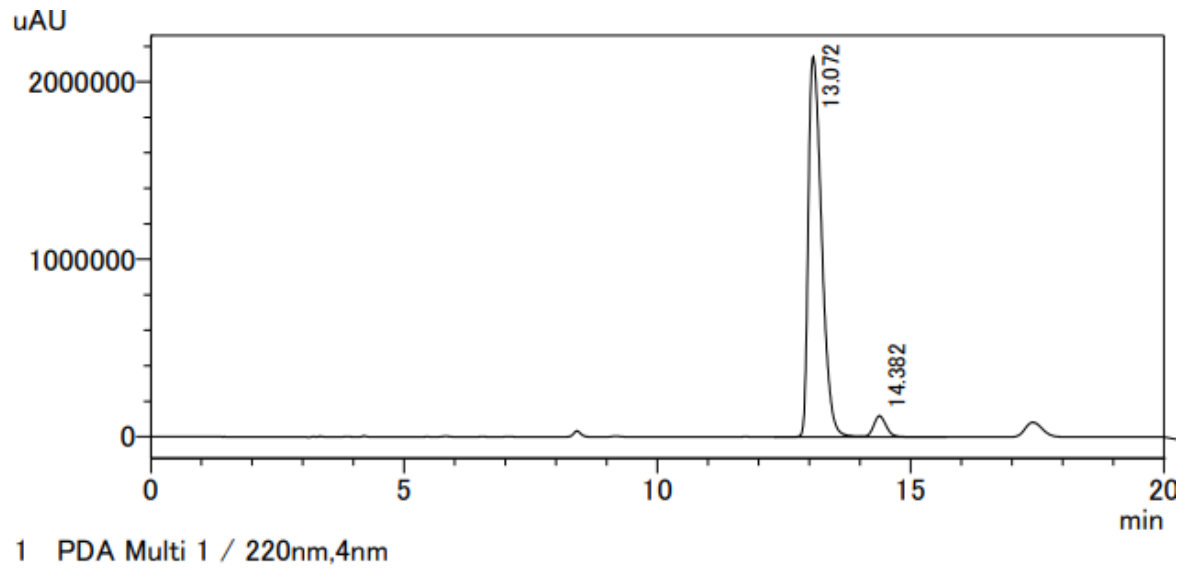

Peak Table

PDA Ch1 220nm

Peak#	Ret. Time	Area	Area%
1	13.072	39195217	94.959
2	14.382	2080917	5.041
‡Œv		41276134	100.000

(4*R*,5*S*)-3-Acetyl-5-methyl-4-(thiophen-2-yl)-5-methoxycarbonyl-2-pyrroline (3ka)

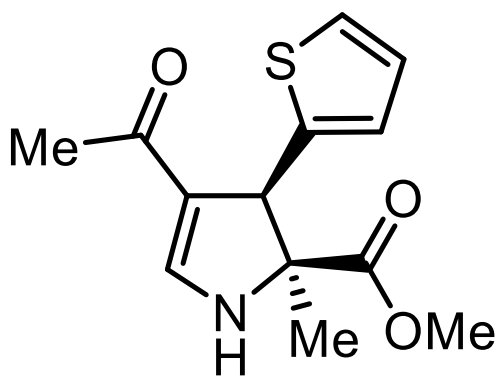

3ka

NMR yield: 59% yield, dr 85:15

Isolated yield: 31.3 mg (major), 3.1 mg (minor), total 52% yield, dr 90:10, 94% ee/30% ee. Yellow foamy solid.

^{1}H NMR (400 MHz, $CDCl_3$) δ 7.36 (d, *J* = 3.2 Hz, 1H), 7.09 (dd, *J* = 4.8, 0.8 Hz, 1H), 6.87 (dd, *J* = 4.8, 3.6 Hz, 1H), 6.83 (dd, *J* = 3.6, 0.8 Hz, 1H), 5.56 (brs, 1H), 4.37 (s, 1H), 3.35 (s, 3H), 2.13 (s, 3H), 1.58 (s, 3H).

^{13}C NMR (101 MHz, $CDCl_3$) δ 191.2, 172.0, 147.1, 143.1, 126.4, 125.4, 124.0, 117.2, 73.9, 52.0, 51.3, 25.8, 25.7.

IR(ATR) 3141, 2953, 1740, 1567, 1435, 1191, 1106, 961 cm^{-1}.

HRMS (ESI) *m/z*: $[M+H]^+$ Calcd for $C_{13}H_{16}NO_3S$ 266.0845; Found. 266.0845.

melting point 104.7-105.1 °C

$[\alpha]_D$ $^{25.4}$ –95.7 (*c* 1.0, $CHCl_3$).

The ee value was determined by chiral HPLC analysis: CHIRALPACK® IH-3 column, 4.6 mm × 250 mm, Daicel Chemical Industries, hexane/2-propanol = 75:25, 1.0 mL/min, 40 °C, 300 nm UV detector, retention time = 11.9 min (major), 20.9 min (minor).

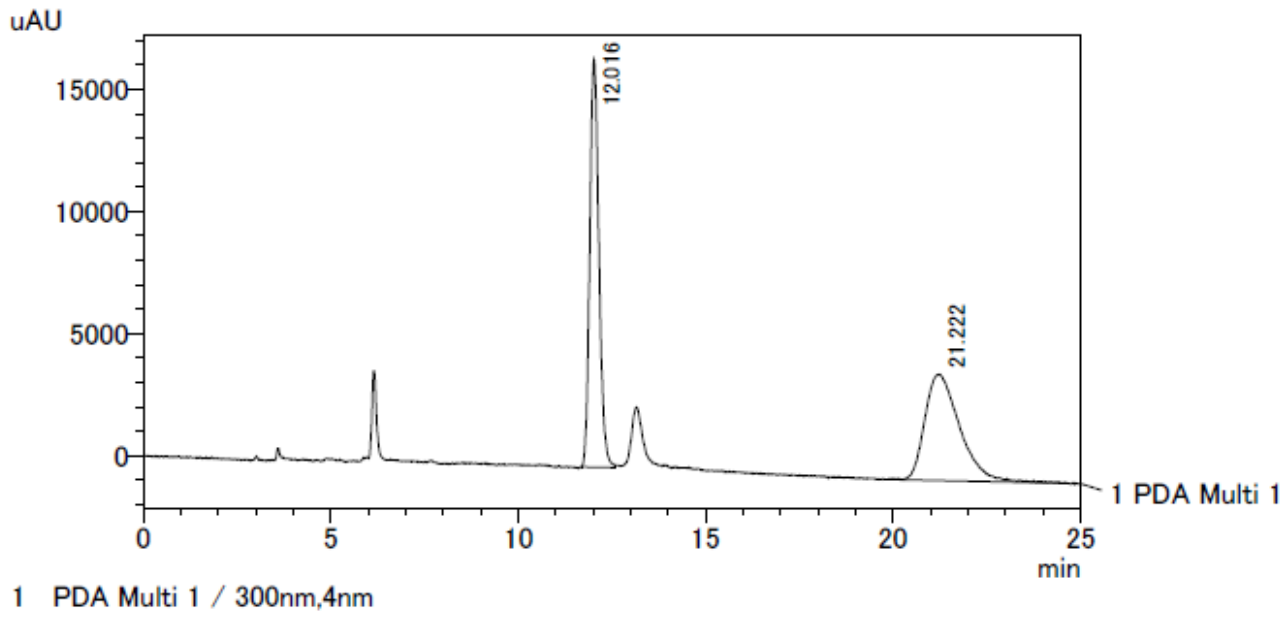

Peak Table

PDA Ch1 300nm

Peak#	Ret. Time	Area	Height	Area%	Height%
1	12.016	284041	16712	50.078	79.341
2	21.222	283151	4351	49.922	20.659
‡Œv		567192	21063	100.000	100.000

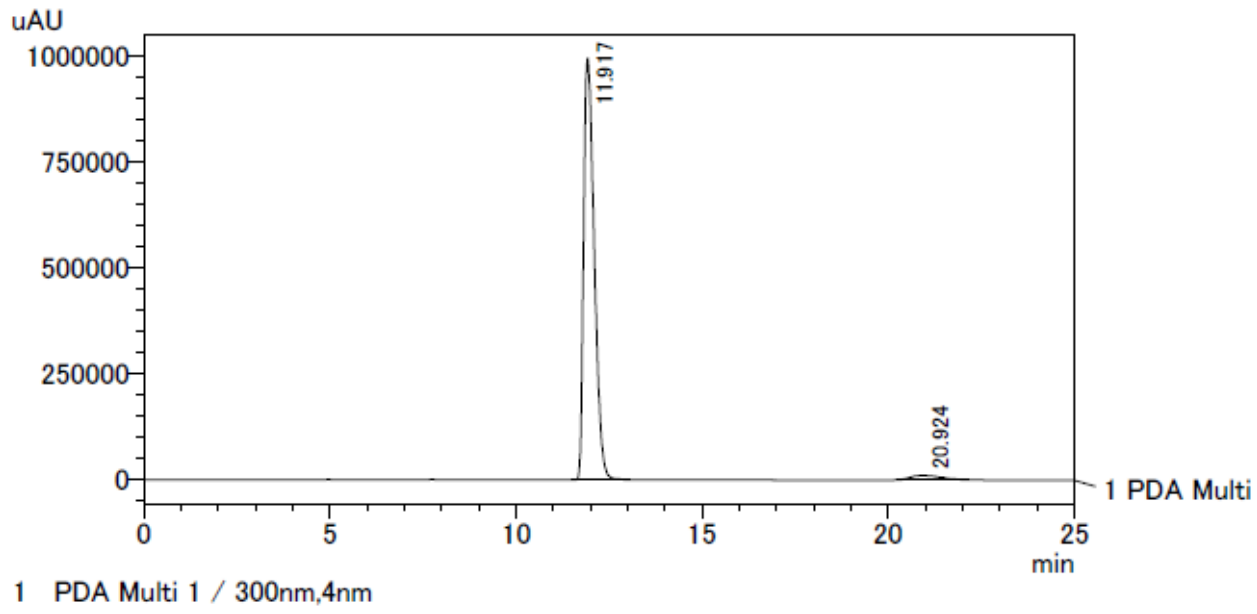

Peak Table

PDA Ch1 300nm

Peak#	Ret. Time	Area	Height	Area%	Height%
1	11.917	19751265	993948	97.054	98.942
2	20.924	599503	10633	2.946	1.058
‡Œv		20350768	1004581	100.000	100.000

(4*R*,5*S*)-3-Acetyl-4-decyl-5-methyl -5-methoxycarbonyl-2-pyrroline (3la)

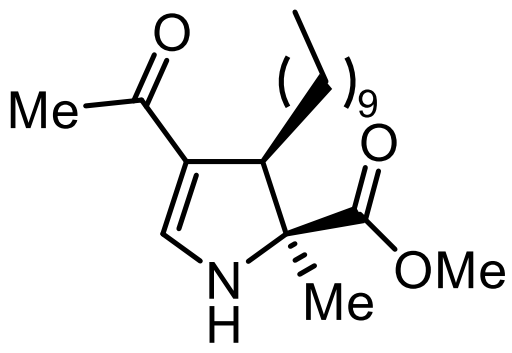

3la

NMR yield: 77% yield, dr 85:15

Isolated yield: 12.3 mg (major), 4.5 mg (minor), total 21% yield, dr 71:29, 90% ee/6% ee. Yellow oil.

^{1}H NMR (400 MHz, $CDCl_3$) δ7.17 (d, *J* = 3.2 Hz, 1H), 4.90 (brs, 1H), 3.77 (s, 3H), 3.04 (t, *J* = 5.6 Hz, 1H), 2.17 (s, 3H), 1.60-1.47 (m, 2H), 1.44 (s, 3H), 1.30-1.10 (m, 16H), 0.89-0.85 (m, 3H).

^{13}C NMR (101 MHz, $CDCl_3$) δ 191.8, 173.6, 146.3, 117.3, 72.0, 52.2, 50.0, 31.9, 30.1, 30.1, 29.7, 29.6, 29.5, 29.3, 29.3, 26.8, 25.6, 22.7, 14.1.

IR(ATR) 3291, 2923, 2854, 1737, 1567, 1436, 1130, 755 cm^{-1}.

HRMS (ESI) *m/z*: $[M+H]^+$ Calcd for $C_{19}H_{34}NO_3$ 324.2533; Found. 324.2530.

$[\alpha]_D$ $^{26.5}$ –13.3 (*c* 1.24, $CHCl_3$).

The ee value was determined by chiral HPLC analysis: CHIRALPACK® IC-3 column, 4.6 mm × 250 mm, Daicel Chemical Industries, hexane/2-propanol = 75:25, 1.0 mL/min, 40 °C, 300 nm UV detector, retention time = 5.5 min (minor), 7.4 min (major).

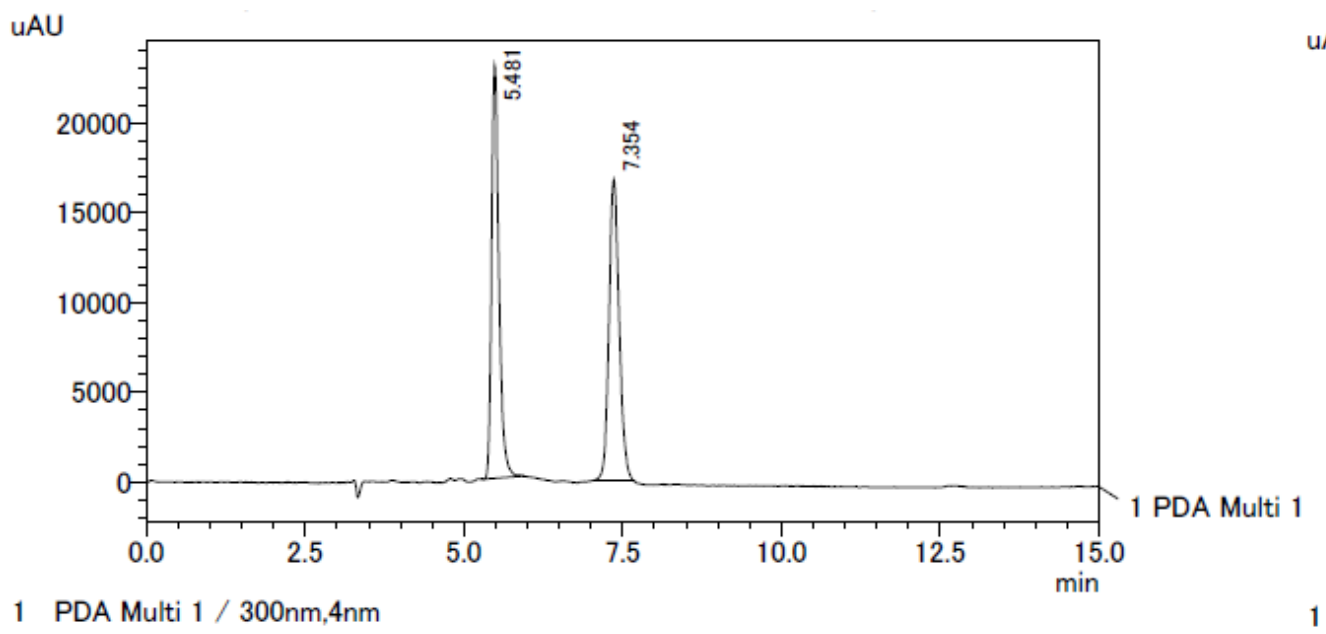

Peak Table

PDA Ch1 300nm

Peak#	Ret. Time	Area	Height	Area%	Height%
1	5.481	185070	23022	49.754	57.896
2	7.354	186900	16742	50.246	42.104
‡Œv		371970	39764	100.000	100.000

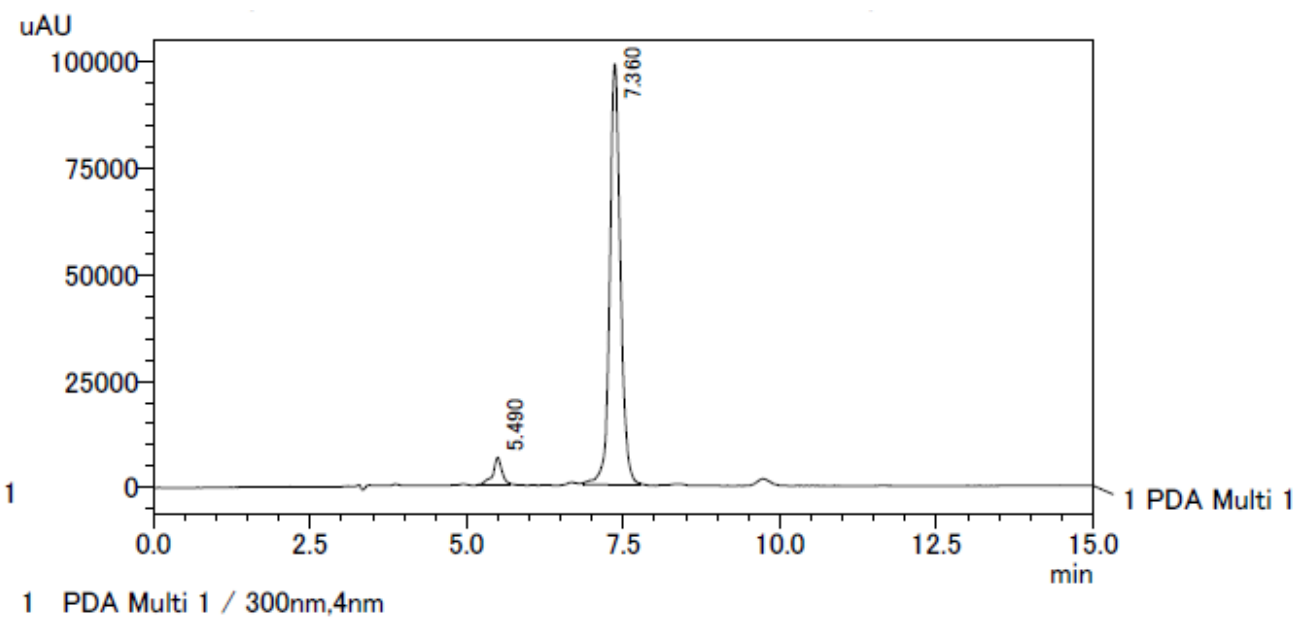

Peak Table

PDA Ch1 300nm

Peak#	Ret. Time	Area	Height	Area%	Height%
1	5.490	62928	6334	5.098	6.040
2	7.360	1171507	98539	94.902	93.960
‡Œv		1234435	104874	100.000	100.000

(4*R*,5*S*)-3-Acetyl-5-methyl-4-cyclohexyl-5-methoxycarbonyl-2-pyrroline (3ma)

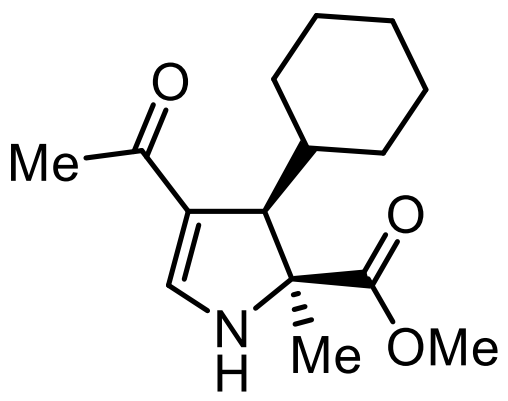

3ma

NMR yield: 29% yield, dr >87:13

Isolated yield: 22.3 mg, 35% yield, dr >20:1, 94% ee. Colorless oil.

^{1}H NMR (400 MHz, $CDCl_3$) δ 7.21 (d, *J* = 3.2 Hz, 1H), 4.86 (brs, 1H), 3.80 (s, 3H), 2.93 (d, *J* = 2.0 Hz, 1H), 2.20 (s, 3H), 1.70-1.47 (m, 5H), 1.42 (s, 3H), 1.36-0.84 (m, 6H).

^{13}C NMR (101 MHz, $CDCl_3$) δ 191.9, 173.7, 147.1, 115.9, 73.2, 54.5, 52.2, 41.1, 32.9, 27.7, 27.3, 27.2, 26.7, 26.2, 25.7.

IR(ATR) 3345, 3145, 2917, 2846, 1736, 1548, 1428, 1276, 1186, 1110, 952 cm^{-1}.

HRMS (ESI) *m/z*: $[M+H]^+$ Calcd for $C_{15}H_{24}NO_3$ 266.1751; Found. 266.1750.

$[\alpha]_D$ $^{19.5}$ –95.2 (*c* 0.50, $CHCl_3$).

The ee value was determined by chiral HPLC analysis: CHIRALPACK® IG-3 column, 4.6 mm × 250 mm, Daicel Chemical Industries, hexane/2-propanol = 95:5, 1.0 mL/min, 40 °C, 300 nm UV detector, retention time = 38.3 min (minor), 41.3 min (major).

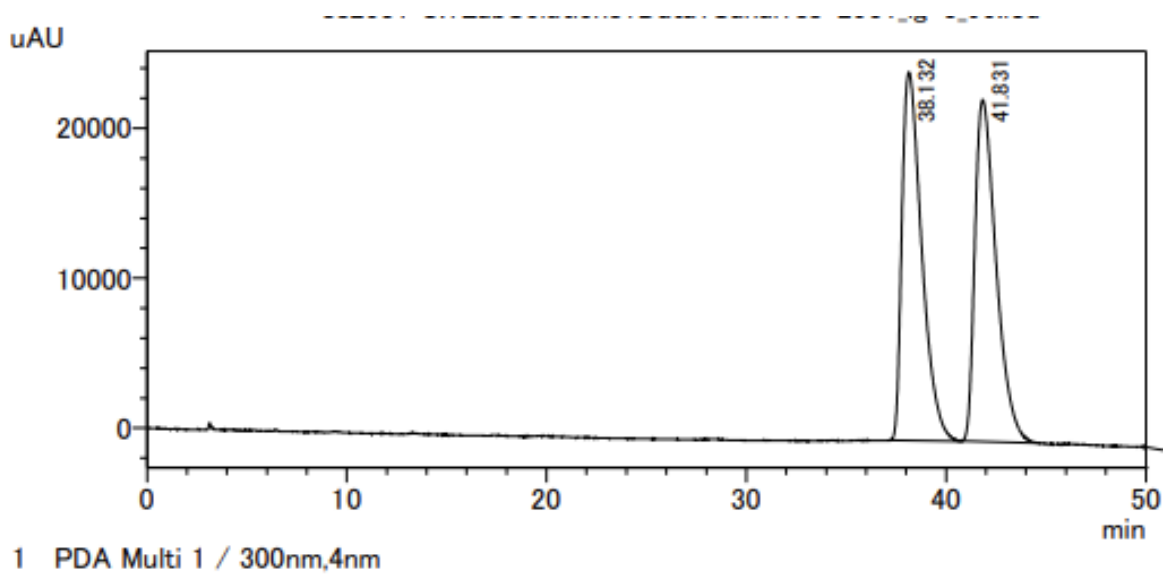

Peak Table

PDA Ch1 300nm

Peak#	Ret. Time	Area	Area%
1	38.132	1693150	50.099
2	41.831	1686449	49.901
‡Œv		3379599	100.000

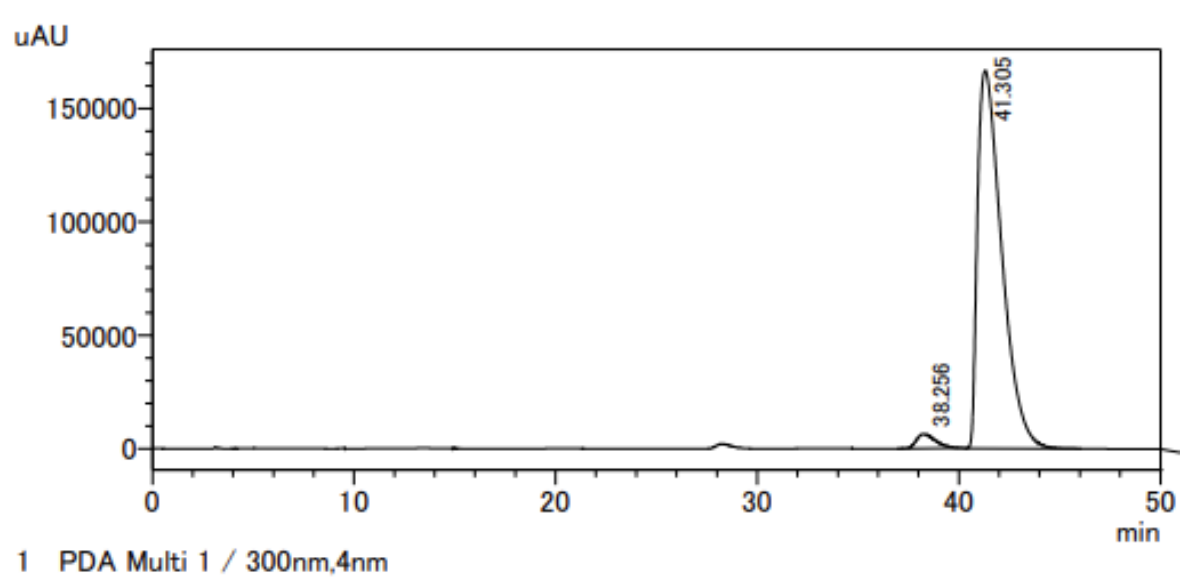

Peak Table

PDA Ch1 300nm

Peak#	Ret. Time	Area	Area%
1	38.256	420422	2.932
2	41.305	13917364	97.068
‡Œv		14337786	100.000

(4*R*,5*S*)-3-Propionyl-5-methyl-4-phenyl-5-methoxycarbonyl-2-pyrroline (3na)

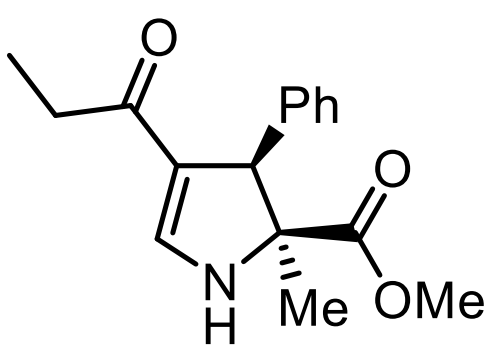

3na

NMR yield: 90% yield, dr 93:7

Isolated yield: 48.1 mg, 70% yield, dr >20:1, 95% ee. Colorless oil.

^{1}H NMR (400 MHz, $CDCl_3$) δ 7.39 (d, *J* = 3.2 Hz, 1H), 7.23-7.19 (m, 2H), 7.16-7.12 (m, 3H), 5.24

(brs, 1H), 4.04 (s, 1H), 3.12 (s, 3H), 2.42 (q, *J* = 7.2 Hz, 2H), 1.59 (s, 3H), 1.02 (t, *J* = 7.2 Hz, 3H).
^{13}C NMR (101 MHz, $CDCl_3$) δ 195.0, 172.5, 146.0, 139.7, 127.9, 127.8, 126.9, 116.7, 73.7, 57.1, 51.7, 31.2, 26.8, 9.3.
IR(ATR) 3235, 2950, 1737, 1556, 1452, 1280, 1170, 1126, 865, 748, 698 cm^{-1}.
HRMS (ESI) *m/z*: $[M+H]^+$ Calcd for $C_{16}H_{20}NO_3$ 274.1438; Found. 274.1431.
$[\alpha]_D$ $^{19.7}$ –80.2 (*c* 0.60, $CHCl_3$).
The ee value was determined by chiral HPLC analysis: CHIRALPACK® IE-3 column, 4.6 mm × 250 mm, Daicel Chemical Industries, hexane/2-propanol = 75:25, 1.0 mL/min, 40 °C, 220 nm UV detector, retention time = 8.7 min (major), 9.8 min (minor).

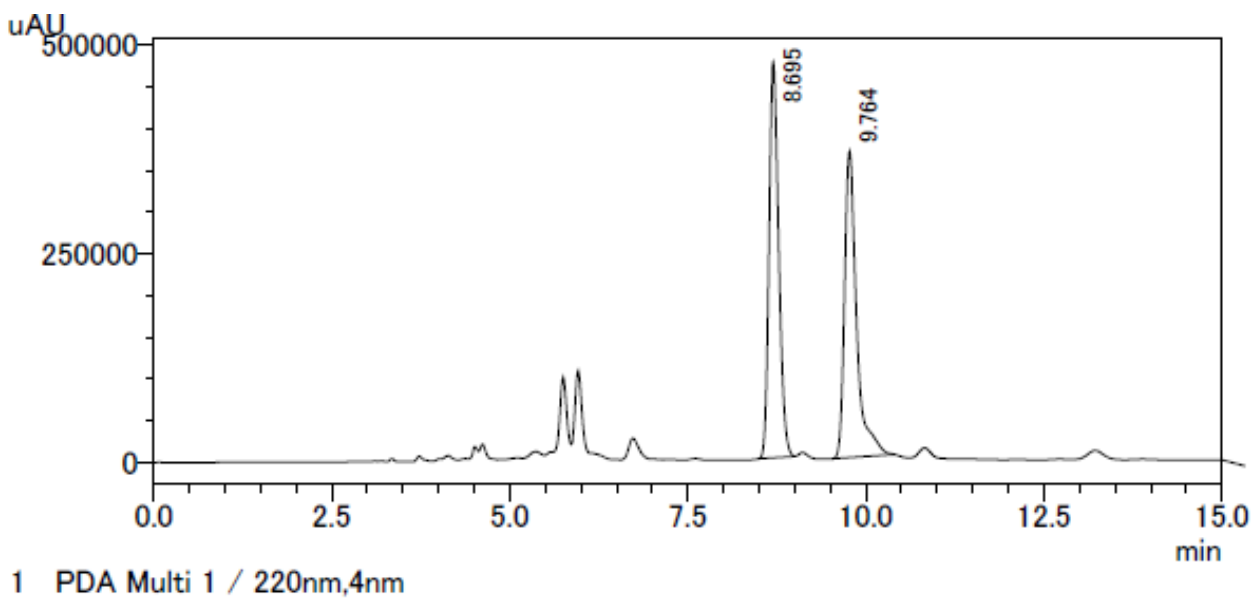

Peak Table

PDA Ch1 220nm

Peak#	Ret. Time	Area	Area%
1	8.695	4529786	50.991
2	9.764	4353630	49.009
‡Œv		8883416	100.000

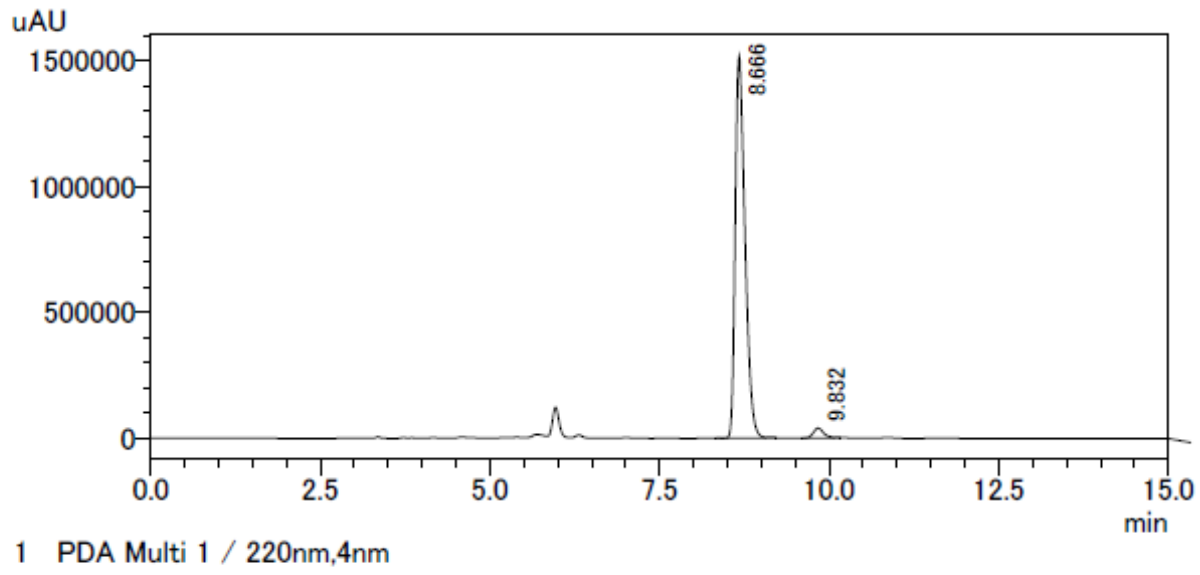

Peak Table

PDA Ch1 220nm

Peak#	Ret. Time	Area	Area%
1	8.666	15403078	97.373
2	9.832	415491	2.627
‡Œv		15818570	100.000

(4*R*,5*S*)-3-Isobutyryl-5-methyl-4-phenyl-5-methoxycarbonyl-2-pyrroline (3oa)

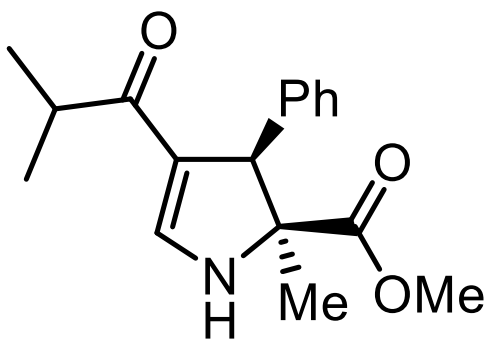

3oa

NMR yield: 81% yield, dr 89:11
Isolated yield: 48.3 mg, 71% yield, dr 20:1, 87% ee. Colorless oil.
^{1}H NMR (400 MHz, $CDCl_3$) δ 7.40 (d, *J* = 3.2 Hz, 1H), 7.22-7.10 (m, 5H), 5.09 (brs, 1H), 4.06 (s, 1H), 3.12 (s, 3H), 2.92 (sep, *J* = 6.8 Hz, 1H), 1.60 (s, 3H), 1.01 (d, *J* = 7.2 Hz, 6H).
^{13}C NMR (101 MHz, $CDCl_3$) δ 198.5, 172.5, 146.0, 139.8, 127.8, 127.7, 126.8, 115.6, 73.5, 57.0, 51.6, 35.3, 26.7, 20.1, 19.3.
IR(ATR) 3220, 2969, 1737, 1556, 1453, 1280, 1127, 1085, 984, 840, 731, 698 cm^{-1}.
HRMS (ESI) *m/z*: $[M+H]^+$ Calcd for $C_{17}H_{22}NO_3$ 288.1594; Found. 288.1588.
$[\alpha]_D$ $^{20.3}$ –100.7 (*c* 1.08, $CHCl_3$).
The ee value was determined by chiral HPLC analysis: CHIRALPACK® IG-3 column, 4.6 mm × 250 mm, Daicel Chemical Industries, hexane/2-propanol = 85:15, 1.0 mL/min, 40 °C, 220 nm UV detector, retention time = 8.3 min (major), 16.6 min (minor).

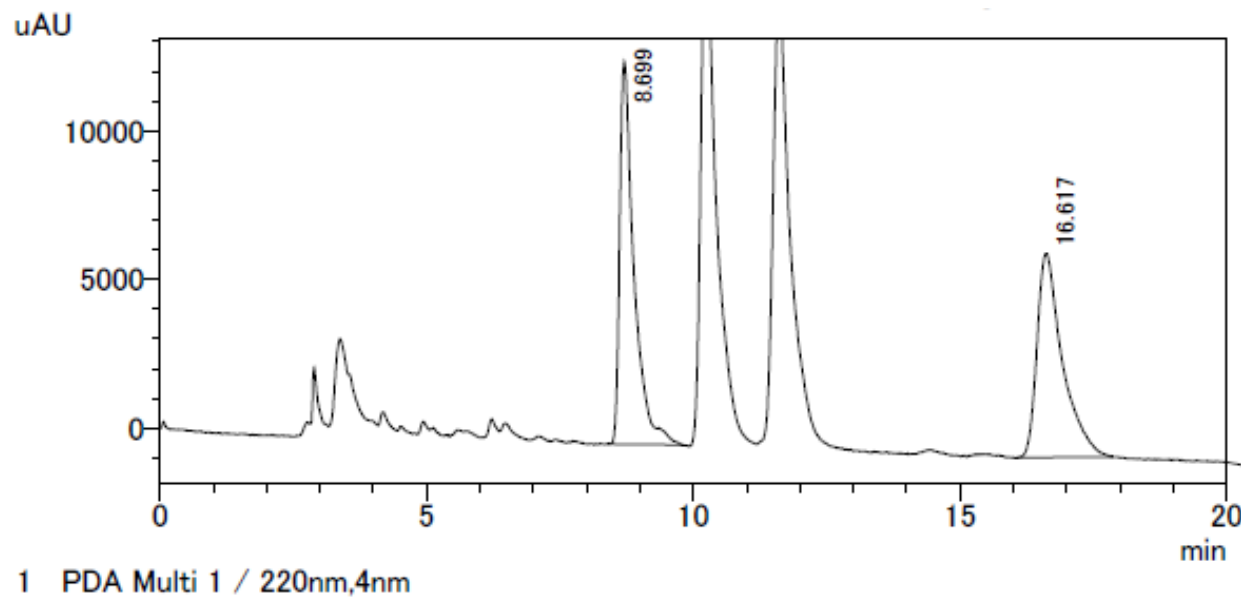

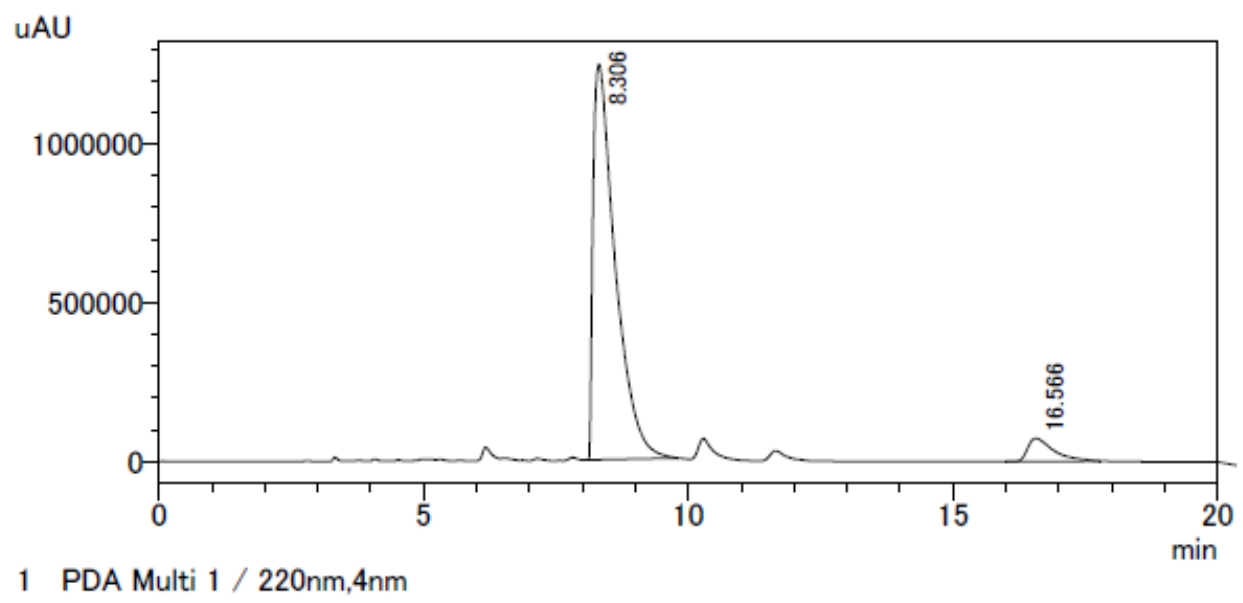

Peak Table

PDA Ch1 220nm

Peak#	Ret. Time	Area	Area%
1	8.699	240017	51.352
2	16.617	227382	48.648
‡Œv		467399	100.000

Peak Table

PDA Ch1 220nm

Peak#	Ret. Time	Area	Area%
1	8.306	36241661	93.629
2	16.566	2466048	6.371
‡Œv		38707709	100.000

(4*R*,5*S*)-3-benzoyl-5-methyl-4-phenyl-5-methoxycarbonyl-2-pyrroline (3pa)

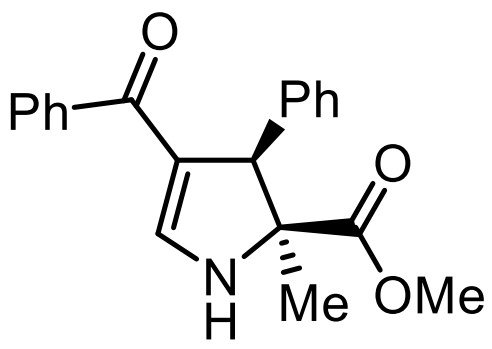

3pa

NMR yield: 45% yield, dr 53:47

Isolated yield (mixture of diastereomers): 23.3 mg, 29% yield, dr 70:30, 57% ee/1% ee. Yellow foamy solid.

^{1}H NMR (400 MHz, $CDCl_3$) δ 7.61-7.57 (m, 2H), 7.47-7.35 (m, 3H), 7.33-7.12 (m, 6H), 5.26-5.17 (m, 1H), 4.77 (s, 0.3H), 4.23 (s, 0.7H), 3.83 (s, 0.9H), 3.15 (s, 2.1H), 1.66 (s, 2.1H), 1.04 (s, 0.9H).

^{13}C NMR (101 MHz, $CDCl_3$) δ 189.5, 176.4, 172.4, 151.7, 150.1, 140.8, 140.7, 139.5, 137.8, 130.4, 130.3, 128.6, 128.2, 128.1, 127.9, 127.9, 127.8, 127.1, 127.0, 117.8, 116.3, 73.8, 71.3, 57.3, 53.0, 52.3, 51.8, 26.8, 21.6. (All detected peaks are described.)

IR(ATR) 3190, 2950, 1737, 1540, 1440, 1393, 1128, 720, 698 cm^{-1}.

HRMS (ESI) *m/z*: $[M+H]^+$ Calcd for $C_{20}H_{0}NO_3$ 322.1438; Found. 322.1433.

melting point 51.8-54.9 ℃

The ee value was determined by chiral HPLC analysis: CHIRALPACK® IH-3 column, 4.6 mm × 250 mm, Daicel Chemical Industries, hexane/2-propanol = 75:25, 1.0 mL/min, 40 ℃, 220 nm UV detector, retention time (major diastereomer) = 7.6 min (major), 13.0 min (minor), retention time (minor diastereomer) = 8.9 min (minor), 9.6 min (major).

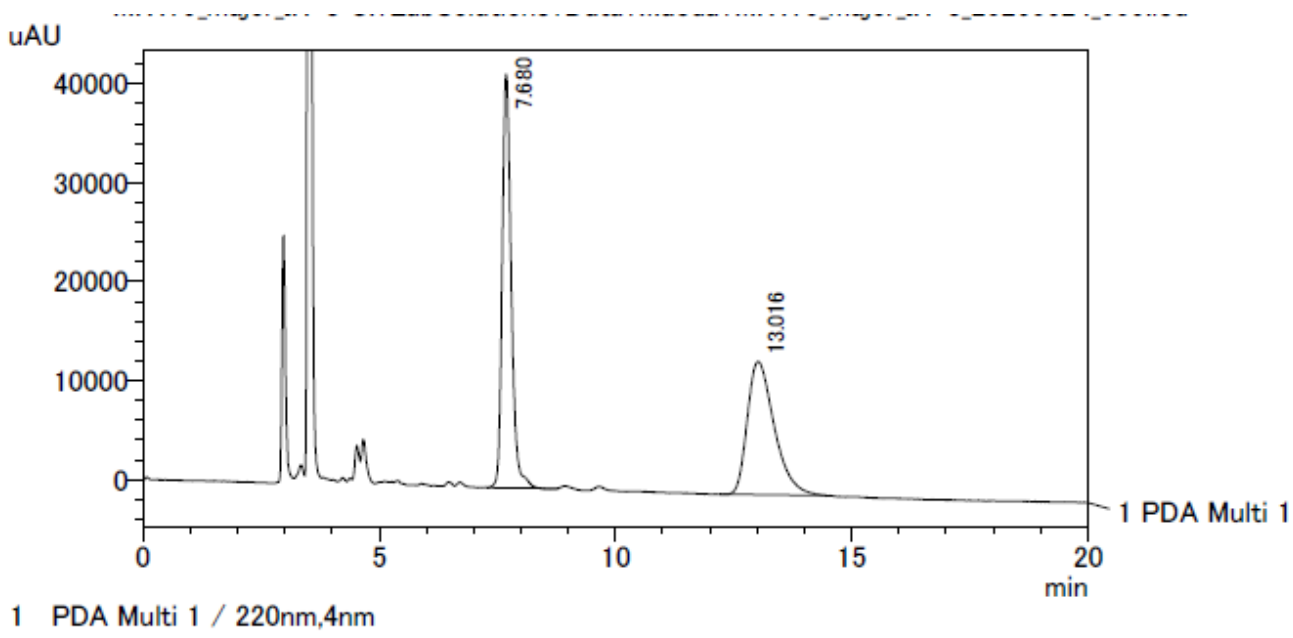

Peak Table

PDA Ch1 220nm

Peak#	Ret. Time	Area	Height	Area%	Height%
1	7.680	555062	41755	50.939	75.627
2	13.016	534592	13457	49.061	24.373
‡Œv		1089654	55212	100.000	100.000

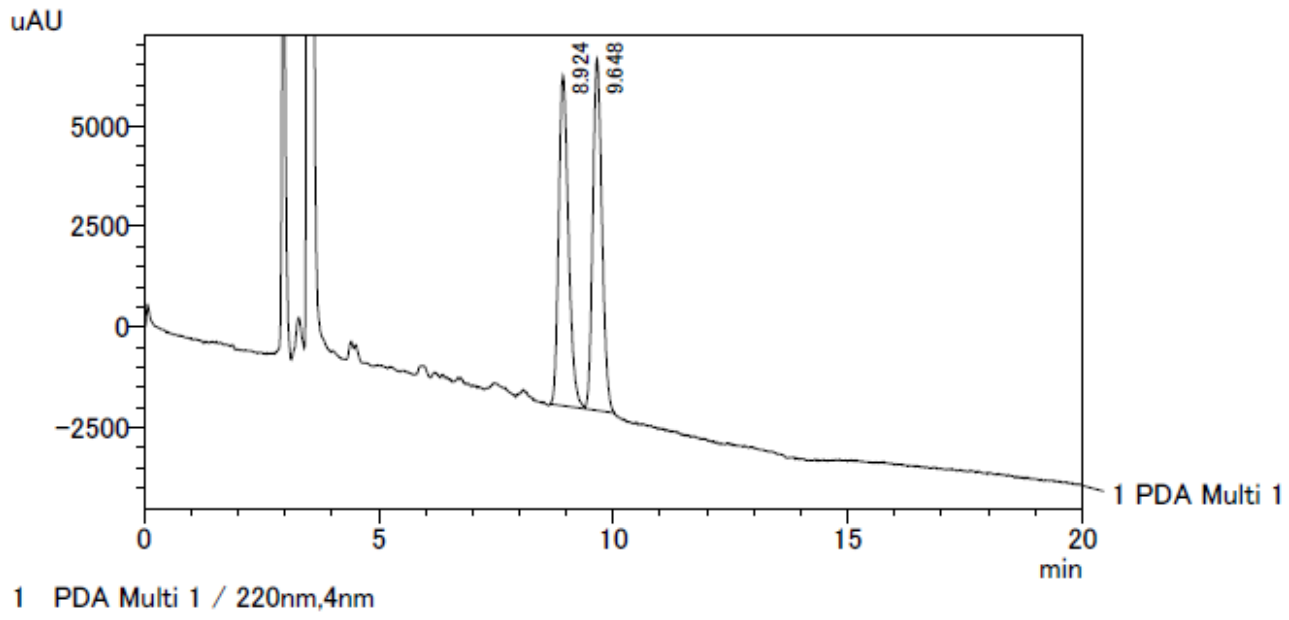

Peak Table

PDA Ch1 220nm

Peak#	Ret. Time	Area	Height	Area%	Height%
1	8.924	119203	8197	50.042	48.479
2	9.648	119005	8712	49.958	51.521
‡Œv		238207	16909	100.000	100.000

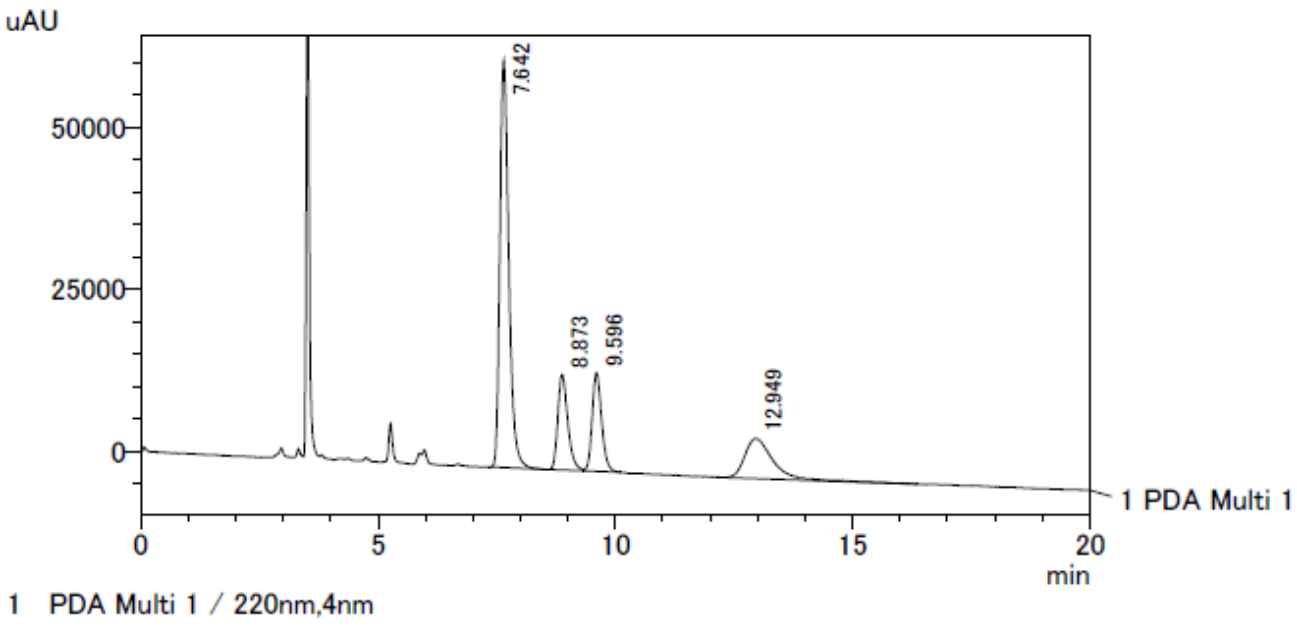

Peak Table

PDA Ch1 220nm

Peak#	Ret. Time	Area	Height	Area%	Height%
1	7.642	816982	62936	54.450	63.551
2	8.873	216317	14748	14.417	14.893
3	9.596	212189	15202	14.142	15.350
4	12.949	254950	6146	16.992	6.206
‡Œv		1500437	99032	100.000	100.000

(4*R*,5*S*)-3-Acetyl-5-propyl-4-phenyl-5-methoxycarbonyl-2-pyrroline (3ab)

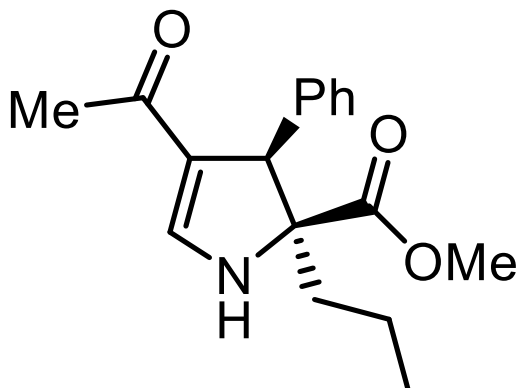

3ab

NMR yield: 95% yield, dr 97:3

Isolated yield: 52.8 mg, 74% yield, dr >20:1, 98% ee. Colorless oil.

^{1}H NMR (400 MHz, $CDCl_3$) δ 7.37 (d, *J* = 2.7 Hz, 1H), 7.23-7.19 (m, 2H), 7.16-7.12 (m, 3H), 5.54 (brs, 1H), 4.05 (s, 1H), 3.09 (s, 3H), 2.08 (s, 3H), 1.98-1.85 (m, 2H), 1.45-1.32 (m, 1H), 1.19-1.06 (m, 1H), 0.92 (t, *J* = 7.2 Hz, 3H).

^{13}C NMR (101 MHz, $CDCl_3$) δ 191.0, 172.0, 147.3, 139.8, 128.1, 127.8, 126.8, 117.9, 77.6, 56.7, 51.5, 42.4, 25.7, 17.2, 14.0.

IR(ATR) 3219, 2959, 1737, 1556, 1454, 1436, 1222, 1138, 750, 731, 698 cm^{-1}.

HRMS (ESI) *m/z*: $[M+H]^+$ Calcd for $C_{17}H_{22}NO_3$ 288.1594; Found. 288.1587.

$[\alpha]_D$ $^{19.4}$–144.6 (*c* 0.68, $CHCl_3$).

The ee value was determined by chiral HPLC analysis: CHIRALPACK® IG-3 column, 4.6 mm × 250 mm, Daicel Chemical Industries, hexane/2-propanol = 85:15, 1.0 mL/min, 40 °C, 220 nm UV detector,

retention time = 11.4 min (major), 17.8 min (minor).

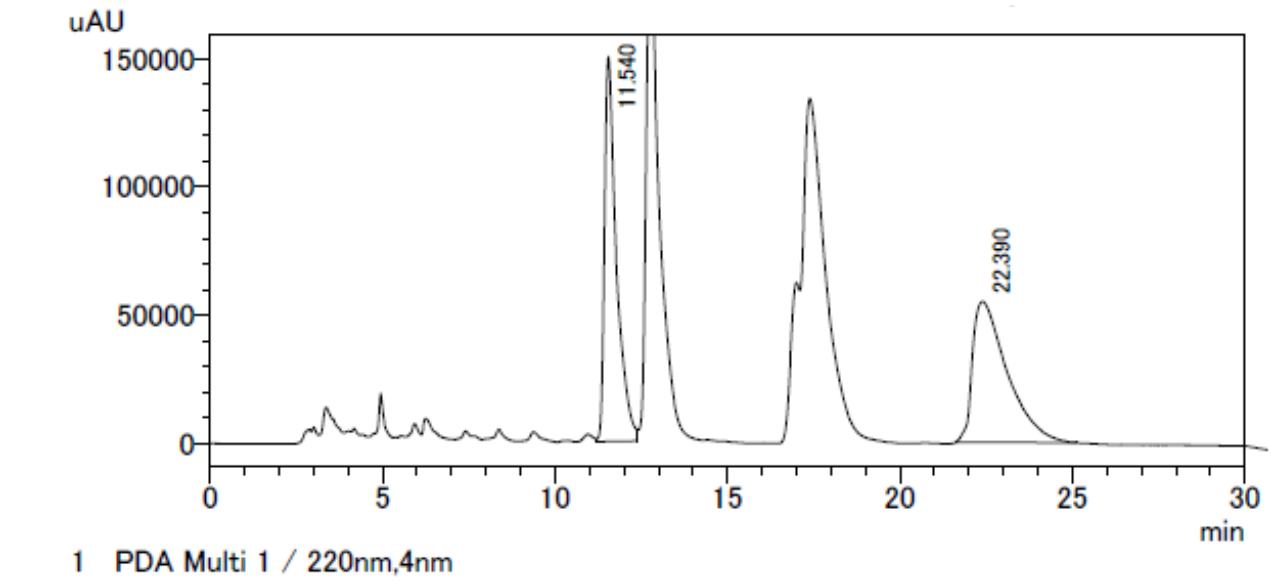

Peak Table

PDA Ch1 220nm

Peak#	Ret. Time	Area	Area%
1	11.540	3583602	48.301
2	22.390	3835715	51.699
‡Œv		7419317	100.000

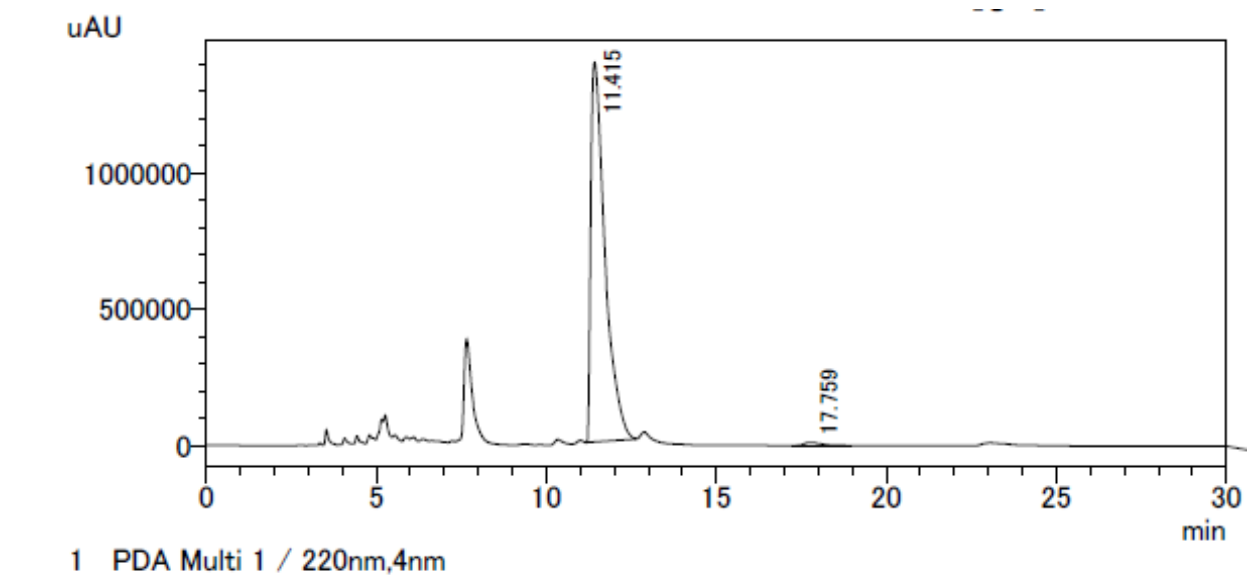

Peak Table

PDA Ch1 220nm

Peak#	Ret. Time	Area	Area%
1	11.415	39993453	98.972
2	17.759	415475	1.028
‡Œv		40408928	100.000

(4*R*,5*S*)-3-Acetyl-5-isobutyl-4-phenyl-5-methoxycarbonyl-2-pyrroline (3ac)

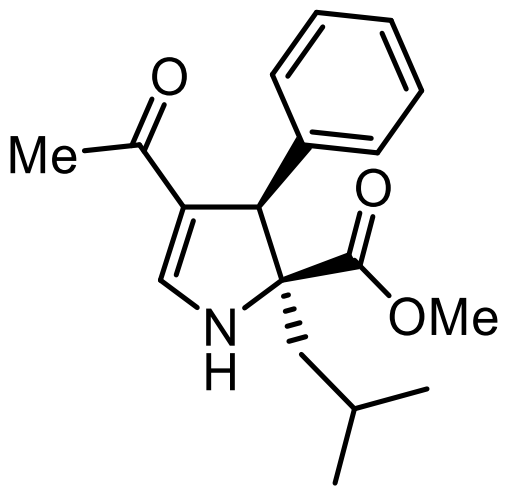

3ac

NMR yield: 72% yield, dr 97:3

Isolated yield: 54.8 mg, 73% yield, dr >20:1, 97% ee. White solid.

^{1}H NMR (400 MHz, $CDCl_3$) δ 7.39 (d, *J* = 3.2 Hz, 1H), 7.23-7.19 (m, 2H), 7.17-7.11 (m, 3H), 5.50 (brs, 1H), 4.00 (s, 1H), 3.09 (s, 3H), 2.08 (s, 3H), 1.93 (d, *J* = 6.4 Hz, 2H), 1.74-1.64 (m, 1H), 0.94 (d, *J* = 6.4 Hz, 3H), 0.80 (d, *J* = 6.4 Hz, 3H).

^{13}C NMR (101 MHz, $CDCl_3$) δ 191.1, 172.5, 147.1, 139.5, 128.0, 127.8, 126.9, 117.9, 58.1, 51.4, 48.2, 25.8, 24.4, 22.3.

IR(ATR) 3220, 2949, 1749, 1556, 1465, 1455, 1227, 1176, 1131, 891, 698 cm^{-1}.

HRMS (ESI) *m/z*: $[M+H]^+$ Calcd for $C_{18}H_{24}NO_3$ 302.1751; Found. 302.1747.

melting point 159.9-162.5 °C

$[\alpha]_D$ $^{19.5}$ –178.8 (*c* 0.80, $CHCl_3$).

The ee value was determined by chiral HPLC analysis: CHIRALPACK® IE-3 column, 4.6 mm × 250 mm, Daicel Chemical Industries, hexane/2-propanol = 80:20, 1.0 mL/min, 40 °C, 220 nm UV detector, retention time = 10.5 min (major), 13.0 min (minor).

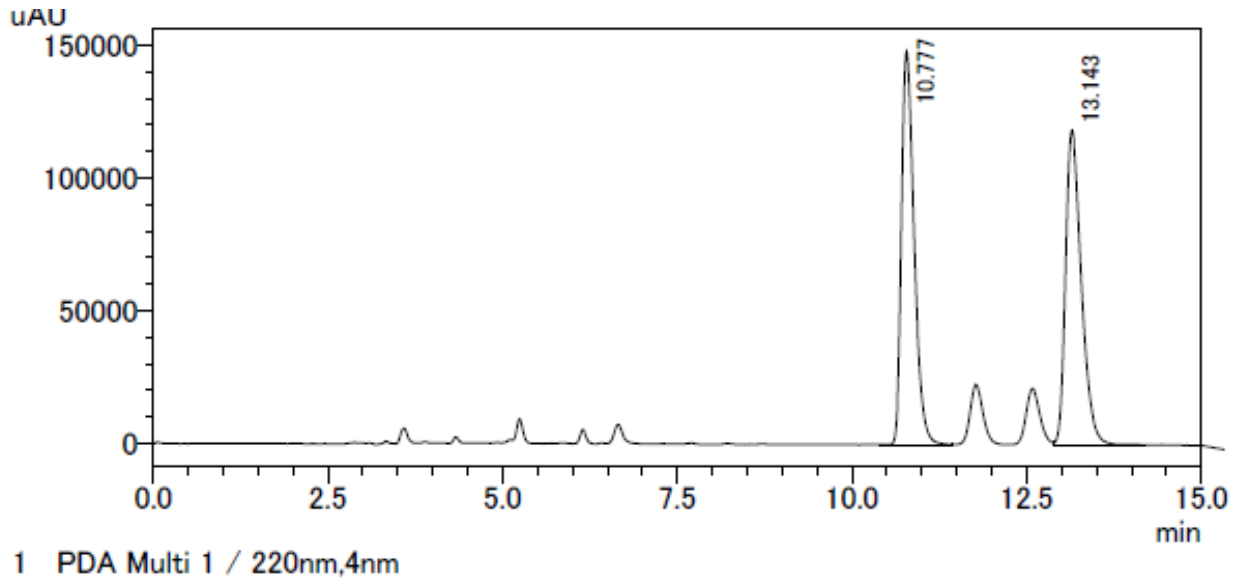

Peak Table

PDA Ch1 220nm

Peak#	Ret. Time	Area	Area%
1	10.777	1861241	49.977
2	13.143	1862977	50.023
‡Œv		3724218	100.000

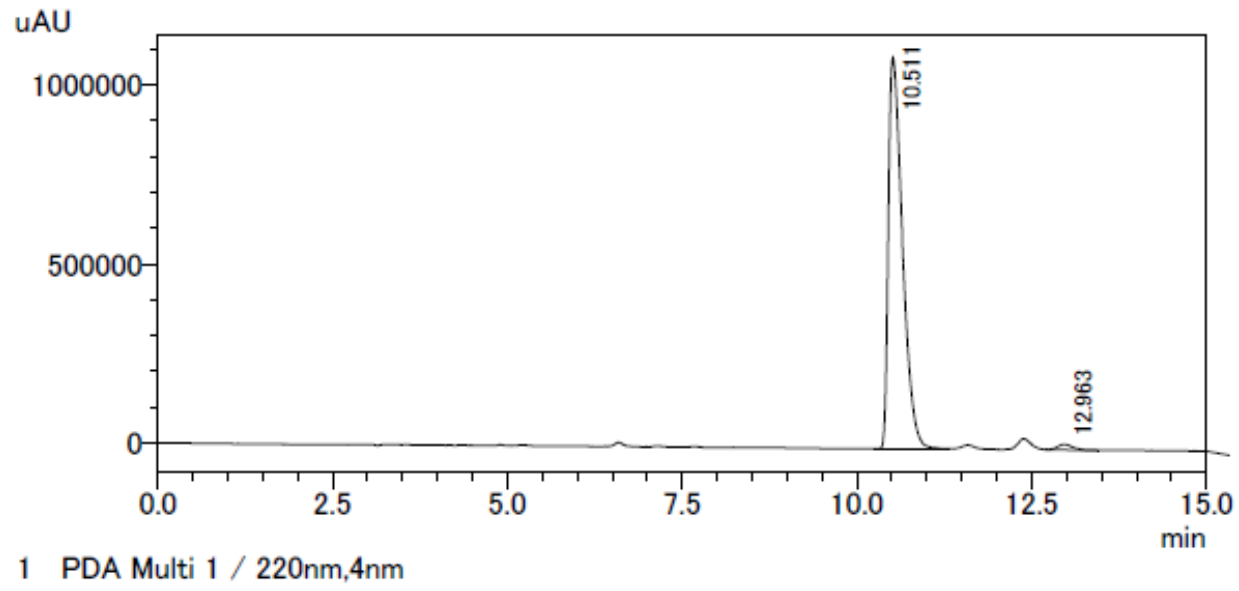

Peak Table

PDA Ch1 220nm

Peak#	Ret. Time	Area	Area%
1	10.511	15239813	98.588
2	12.963	218234	1.412
‡Œv		15458046	100.000

(4*R*,5*S*)-3-Acetyl-5-benzyl-4-phenyl-5-methoxycarbonyl-2-pyrroline (3ad)

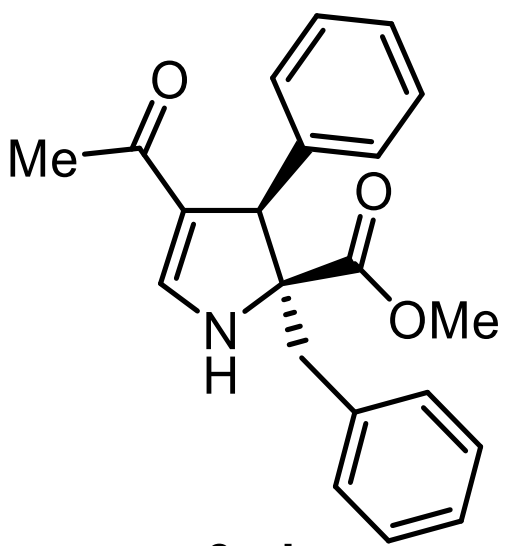

3ad

NMR yield: 72% yield, dr 91:9

Isolated yield: 65.0 mg, 78% yield, dr 89:11, 90% ee. White solid.

^{1}H NMR (400 MHz, $CDCl_3$) for the major diastereomer δ 7.39-7.35 (m, 1H), 7.30-7.13 (m, 8H), 7.10-7.02 (m, 2H), 5.03 (brs, 1H), 4.18 (s, 1H), 3.37 (d, J = 13.2 Hz, 1H), 3.145 (s, 3H), 3.07 (d, J = 13.2 Hz, 1H), 2.09 (s, 3H).

^{13}C NMR (101 MHz, $CDCl_3$) δ 191.2, 170.8, 146.5, 139.2, 135.0, 129.7, 128.5, 128.0, 127.9, 127.3, 127.0, 118.2, 78.0, 56.7, 51.5, 44.8, 25.8.

IR(ATR) 3236, 3030, 1737, 1562, 1454, 1435, 1149, 1087, 895, 729, 698 cm^{-1}.

HRMS (ESI) *m/z*: $[M+H]^+$ Calcd for $C_{21}H_{22}NO_3$ 336.1594; Found. 336.1588.

melting point 47.8-51.6 °C

$[\alpha]_D$ $^{19.4}$ –144.6 (*c* 0.68, $CHCl_3$).

The ee value was determined by chiral HPLC analysis: CHIRALPACK® IE-3 column, 4.6 mm × 250 mm, Daicel Chemical Industries, hexane/2-propanol = 75:25, 1.0 mL/min, 40 °C, 220 nm UV detector, retention time = 12.6 min (major), 15.9 min (minor).

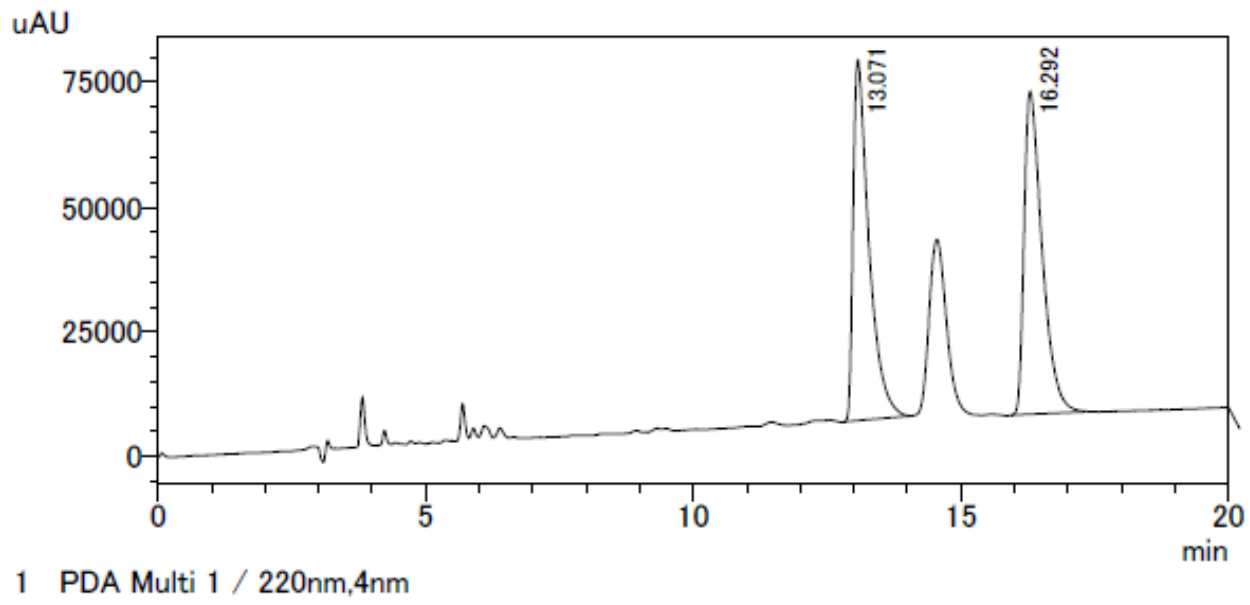

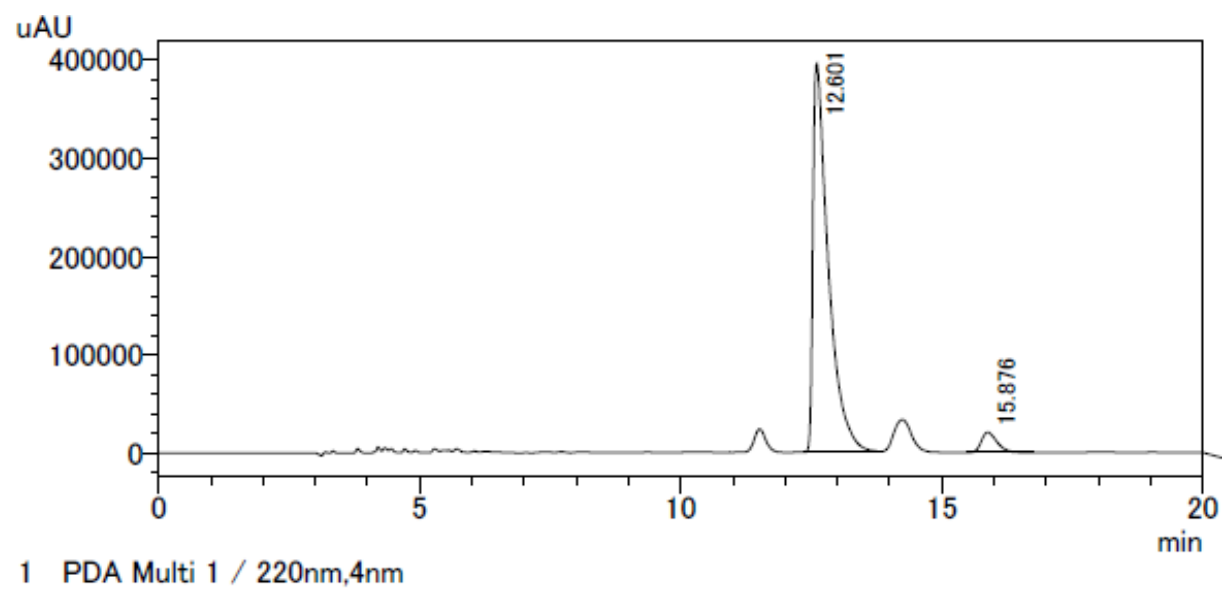

Peak Table

PDA Ch1 220nm

Peak#	Ret. Time	Area	Area%
1	13.071	1484361	49.531
2	16.292	1512472	50.469
‡Œv		2996833	100.000

Peak Table

PDA Ch1 220nm

Peak#	Ret. Time	Area	Area%
1	12.601	8144073	94.959
2	15.876	432348	5.041
‡Œv		8576422	100.000

(4*R*,5*S*)-3-Acetyl-5-isopropyl-4-phenyl-5-methoxycarbonyl-2-pyrroline (3ae)

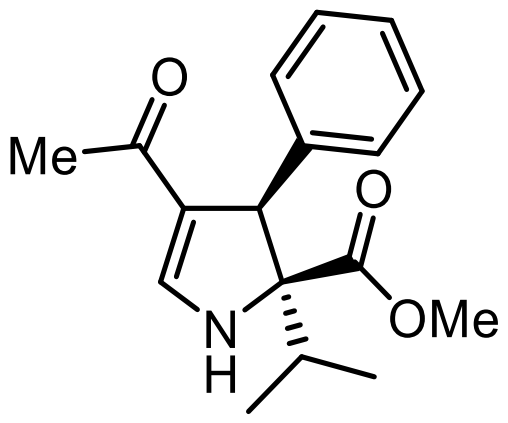

3ae

NMR yield: 56% yield, dr 98:2

Isolated yield: 38.3 mg, 53% yield, dr >20:1, 96% ee. White solid.

^{1}H NMR (400 MHz, $CDCl_3$) δ 7.43 (d, *J* = 3.2 Hz, 1H), 7.23-7.20 (m, 2H), 7.16-7.12 (m, 3H), 5.30 (brs, 1H), 4.17 (s, 1H), 3.02 (s, 3H), 2.32-2.22 (m, 1H), 2.06 (s, 3H), 0.96 (d, *J* = 6.8 Hz, 3H), 0.81 (d, *J* = 6.4 Hz, 3H).

^{13}C NMR (101 MHz, $CDCl_3$) δ 190.6, 172.5, 148.1, 140.9, 127.8, 126.8, 118.5, 80.7, 54.7, 51.4, 37.2, 25.8, 17.6, 15.7.

IR(ATR) 3190, 2965, 1735, 1556, 1489, 1270, 1247, 1171, 1130, 1041, 892, 754, 700 cm^{-1}.

HRMS (ESI) *m/z*: $[M+H]^+$ Calcd for $C_{17}H_{22}NO_3$ 288.1594; Found. 288.1587.

melting point 143.1-145.8 °C

$[\alpha]_D^{20.4}$ –79.5 (*c* 1.59, $CHCl_3$).

The ee value was determined by chiral HPLC analysis: CHIRALPACK® IH-3 column, 4.6 mm × 250 mm, Daicel Chemical Industries, hexane/2-propanol = 85:15, 1.0 mL/min, 40 °C, 220 nm UV detector, retention time = 12.4 min (major), 26.1 min (minor).

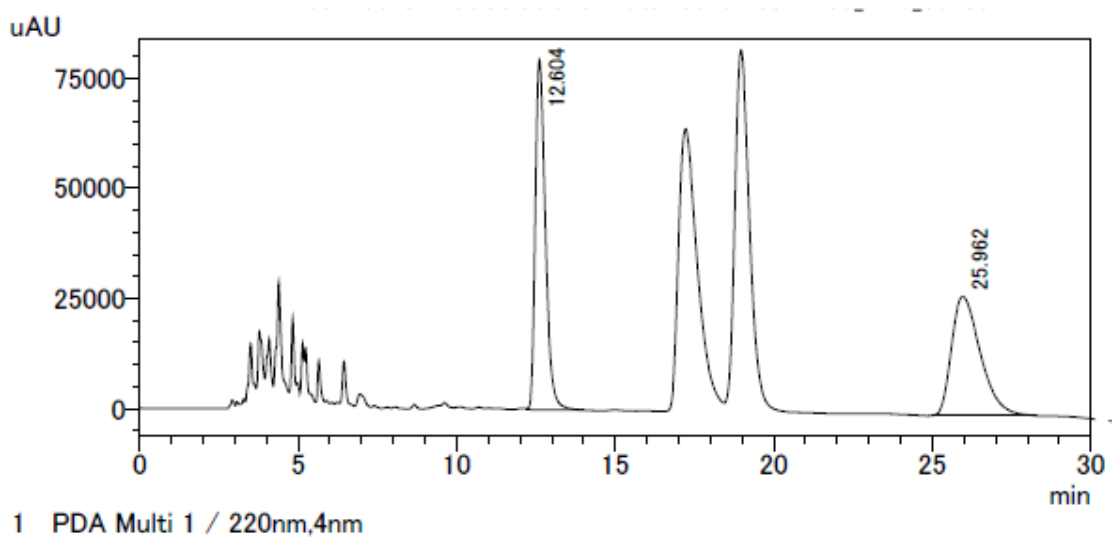

Peak Table

PDA Ch1 220nm

Peak#	Ret. Time	Area	Area%
1	12.604	1743302	50.435
2	25.962	1713263	49.565
‡Œv		3456565	100.000

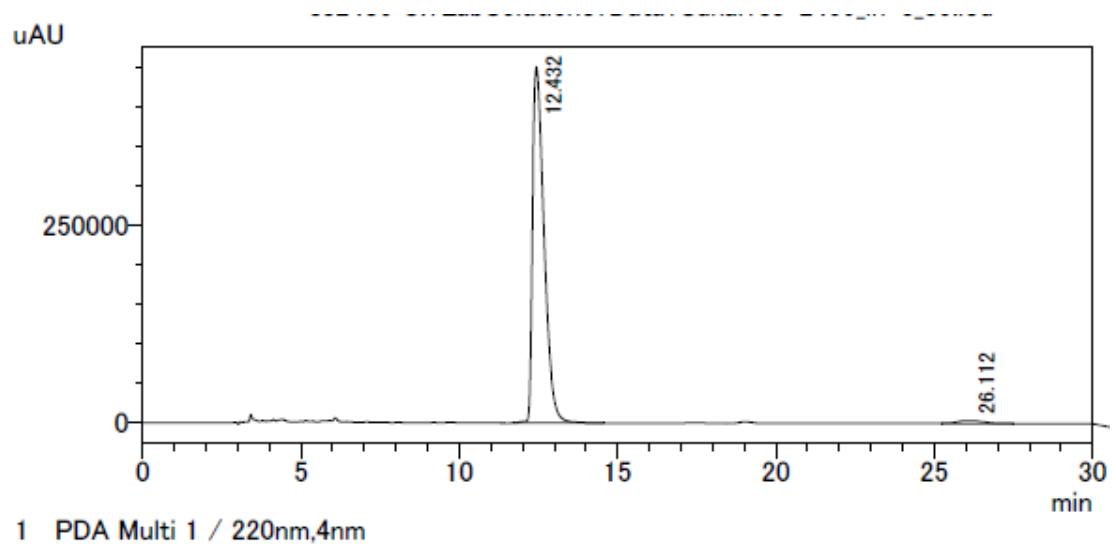

Peak Table

PDA Ch1 220nm

Peak#	Ret. Time	Area	Area%
1	12.432	11433260	98.153
2	26.112	215105	1.847
‡Œv		11648365	100.000

(4*R*,5*S*)-3-Acetyl-2-[(1-(*tert*-butoxycarbonyl)-1*H*-indol-3-yl)methyl]-5-(methoxycarbonyl)-4-phenyl-2-pyrroline (3af)

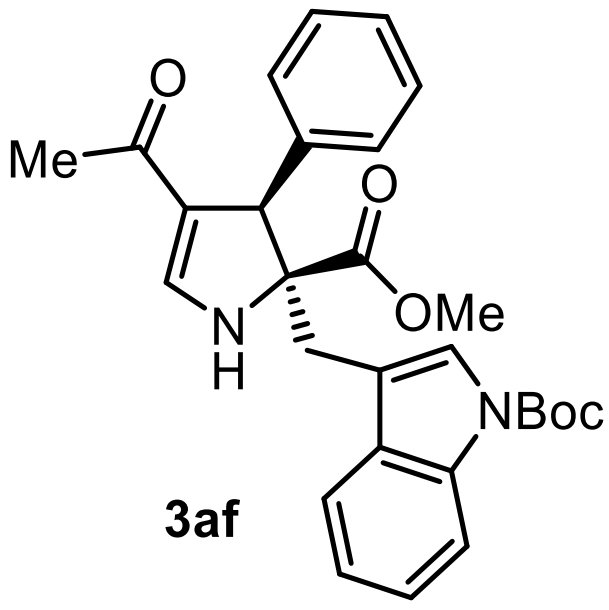

3af

NMR yield: 70% yield, dr 85:15

Isolated yield: 65.6 mg, 55% yield, dr 84:16, 79% ee. Pale yellow foamy solid.

^{1}H NMR (400 MHz, $CDCl_3$) for the major diastereomer δ 8.12-8.04 (m, 1H), 7.49-7.46 (m, 2H), 7.41-7.27 (m, 3H), 7.25-7.14 (m, 5H), 4.98 (d, *J* = 2.4 Hz, 1H), 4.28 (s, 1H), 3.47 (d, *J* = 14.4 Hz, 1H), 3.31 (d, *J* = 14.4 Hz, 1H), 3.12 (s, 3H), 2.16 (s, 3H), 1.66 (s, 9H).

^{13}C NMR (101 MHz, $CDCl_3$) δ 191.4, 175.5, 171.0, 149.5, 148.6, 146.3, 139.2, 137.5, 135.1, 130.7, 130.3, 128.6, 128.0, 127.5, 127.2, 124.6, 124.5, 123.9, 122.6, 122.5, 120.2, 119.0, 119.0, 118.5, 115.3, 115.3, 114.9, 114.5, 84.0, 78.2, 75.8, 56.7, 53.0, 52.8, 51.7, 34.6, 30.3, 28.2, 26.0, 25.9. (all the detected peaks were described)

IR(ATR) 2971, 1736, 1567, 1452, 1367, 1217, 1150, 1090, 731, 698 cm^{-1}.

HRMS (ESI) *m/z*: $[M+H]^+$ Calcd for $C_{28}H_{31}N_2O_5$ 475.2228; Found. 475.2223.

melting point 88.6-91.3 °C

[α]$_D$ $^{22.1}$ –40.9 (*c* 1.10, $CHCl_3$).

The ee value was determined by chiral HPLC analysis: CHIRALPACK® IE-3 column, 4.6 mm × 250 mm, Daicel Chemical Industries, hexane/2-propanol = 75:25, 1.0 mL/min, 40 °C, 220 nm UV detector, retention time = 14.0 min (major), 19.8 min (minor).

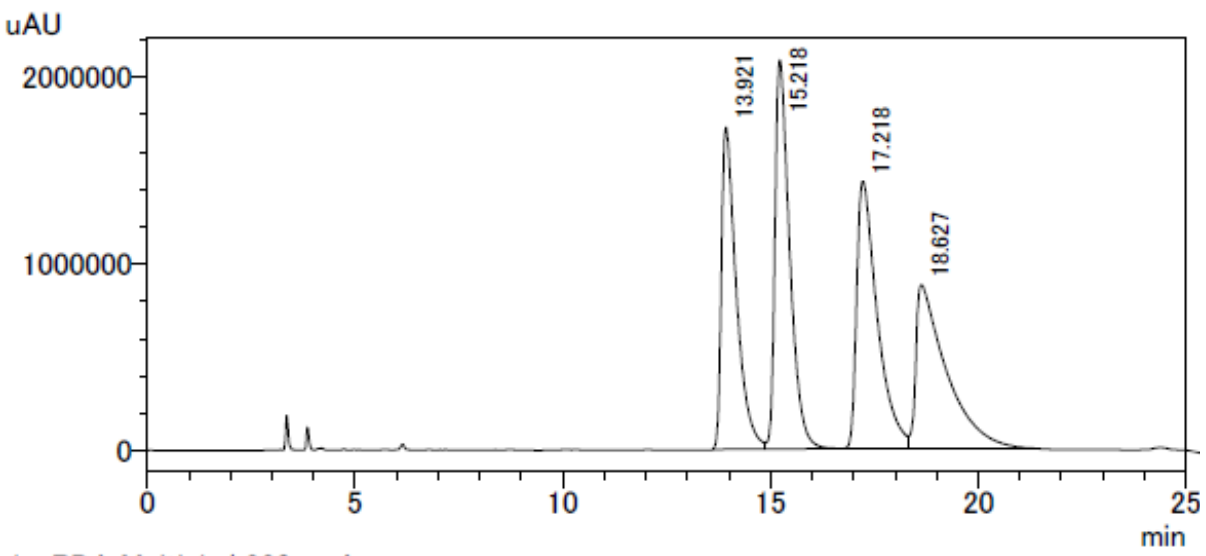

1 PDA Multi 1 / 220nm,4nm

Peak Table

PDA Ch1 220nm

Peak#	Ret. Time	Area	Area%
1	13.921	43042398	22.778
2	15.218	51431175	27.218
3	17.218	49520973	26.207
4	18.627	44967765	23.797
‡Œv		188962312	100.000

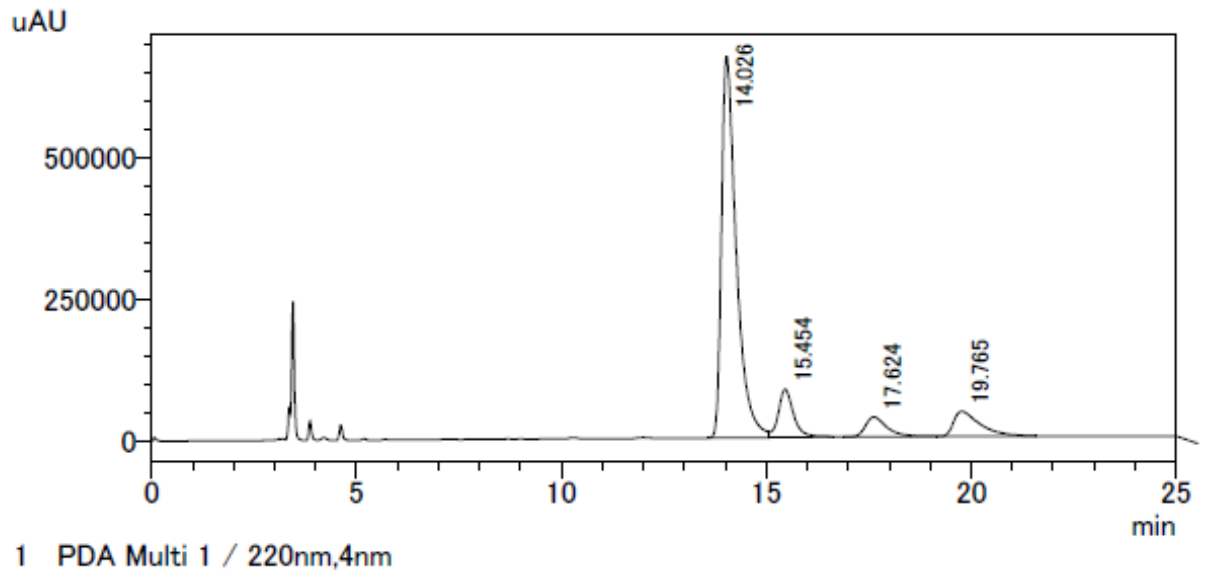

1 PDA Multi 1 / 220nm,4nm

Peak Table

PDA Ch1 220nm

Peak#	Ret. Time	Area	Area%
1	14.026	16875736	75.855
2	15.454	2143354	9.634
3	17.624	1258908	5.659
4	19.765	1969426	8.852
‡Œv		22247424	100.000

(4*R*,5*S*)-3-Acetyl-4,5-diphenyl-5-methoxycarbonyl-2-pyrroline (3ag)

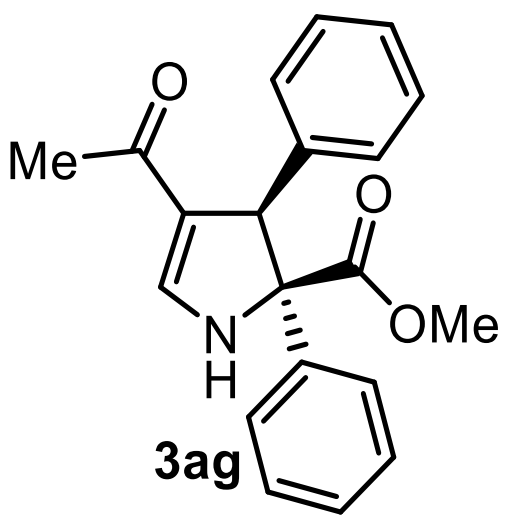

3ag

NMR yield: 46% yield, dr 89:11

Isolated yield: 31.8 mg, 40% yield, dr >20:1, 98% ee. White solid.

^{1}H NMR (400 MHz, $CDCl_3$) δ 7.59 (d, *J* = 7.6 Hz, 2H), 7.43-7.26 (m, 8H), 7.19 (t, *J* = 6.0 Hz, 1H), 5.76 (brs, 1H), 4.68 (s, 1H), 3.11 (s, 3H), 2.06 (s, 3H).

^{13}C NMR (101 MHz, $CDCl_3$) δ 191.0, 170.9, 145.8, 141.9, 140.1, 128.6, 128.3, 128.1, 128.0, 127.2, 125.5, 119.4, 79.1, 57.5, 52.1, 25.8.

IR(ATR) 3181, 2947, 1738, 1558, 1433, 1266, 1243, 1159, 1066, 890, 696 cm^{-1}.

HRMS (ESI) *m/z*: $[M+H]^+$ Calcd for $C_{20}H_{20}NO_3$ 322.1438; Found. 322.1436.

melting point 209.7-210.1 °C

$[\alpha]_D$ $^{19.6}$ –571.6 (*c* 0.61, $CHCl_3$).

The ee value was determined by chiral HPLC analysis: CHIRALPACK® IG-3 column, 4.6 mm × 250 mm, Daicel Chemical Industries, hexane/2-propanol = 75:25, 1.0 mL/min, 40 °C, 220 nm UV detector, retention time = 8.4 min (major), 13.1 min (minor).

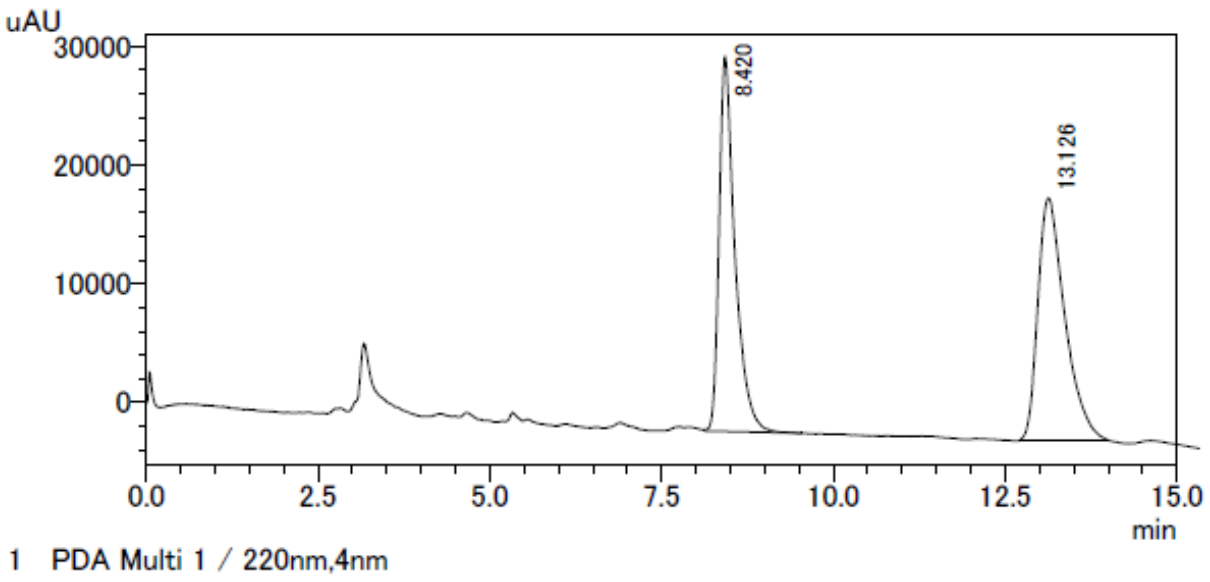

Peak Table

PDA Ch1 220nm

Peak#	Ret. Time	Area	Area%
1	8.420	508393	47.855
2	13.126	553979	52.145
‡Œv		1062372	100.000

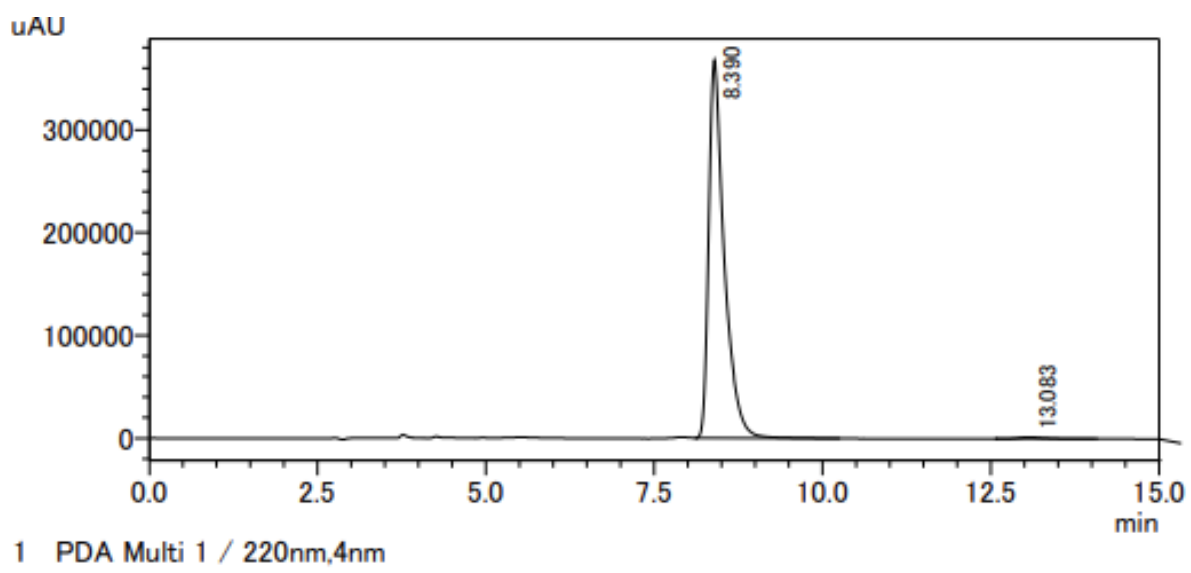

Peak Table

PDA Ch1 220nm

Peak#	Ret. Time	Area	Area%
1	8.390	5930945	99.242
2	13.083	45319	0.758
‡Œv		5976264	100.000

Transformations

(4*R*,5*S*)-3-Acetyl-1-benzoyl-5-methyl-4-phenyl-5-methoxycarbonyl-2-pyrroline (4)

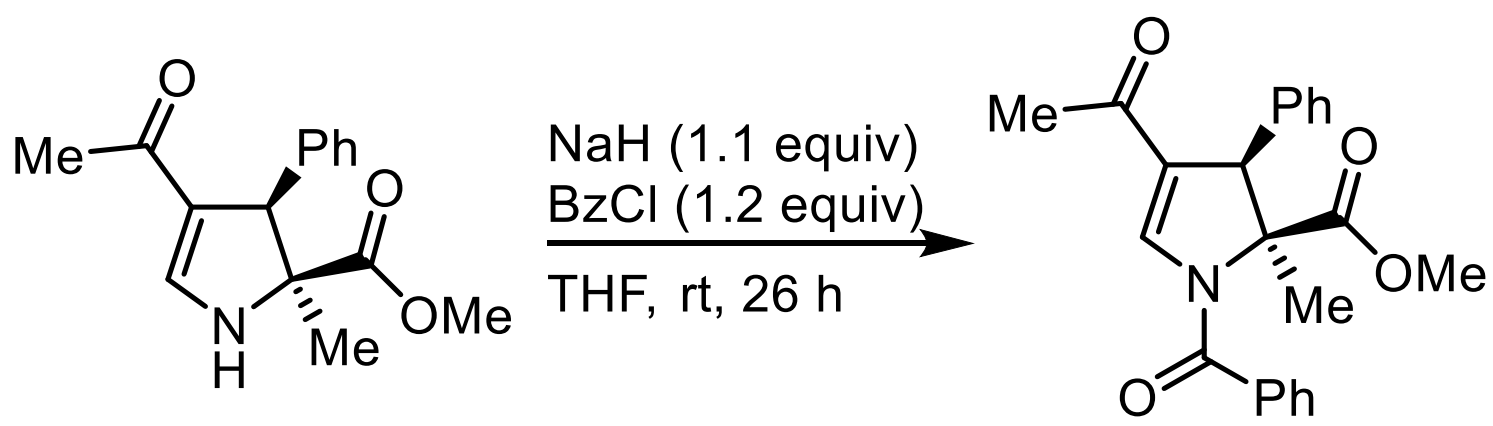

3aa **4**

To a solution of NaH (156 mg, 0.66 mmol) in THF (3 mL) a solution of **3aa** (15.8 mg, 0.60 mmol) in THF (1 mL) was slowly added at –78 °C and benzoyl chloride (86µL, 0.72 mmol) was then added at the same temperature. Next, the reaction mixture was warmed to room temperature. After stirring for 26 hours at room temperature, the reaction mixture was quenched with H_2O (5 mL). The aqueous layer was extracted with EtOAc (5 mL × 5), and the combined organic layer was dried over $MgSO_4$ and concentrated under reduced pressure. The residue was purified by silica gel chromatography (hexane/EtOAc 4:1 to 2:1) to give **4** as a yellow solid (84.9 mg, 39% yield).

^{1}H NMR (400 MHz, $CDCl_3$) δ 7.68-7.51 (m, 6H), 7.31-7.23 (m, 3H), 7.17-7.11 (m, 2H), 4.27 (s, 1H), 3.08 (s, 3H), 2.12 (s, 3H), 1.99 (s, 3H).
^{13}C NMR (101 MHz, $CDCl_3$) δ 193.2, 168.7, 168.0, 141.3, 136.9, 134.0, 131.7, 128.9, 128.10, 128.06, 127.8, 123.4, 75.1, 59.0, 51.8, 26.9, 23.8.
IR(ATR) 2950, 1740, 1654, 1599, 1400, 1359, 1300, 1223, 1116, 1062, 855, 699 cm^{-1}.
HRMS (ESI) *m/z*: $[M+H]^+$ Calcd for $C_{22}H_{22}NO_4$ 364.1543; Found. 364.1540.
melting point 42.5-44.7 °C
$[\alpha]_D$ $^{19.7}$ +53.3 (*c* 0.30, $CHCl_3$).

(3*S*,4*S*,5*S*)-3-Acetyl-3-(benzoyloxy)-5-(methoxycarbonyl)-5-methyl-4-phenyl-2-pyrrolidinone (5)

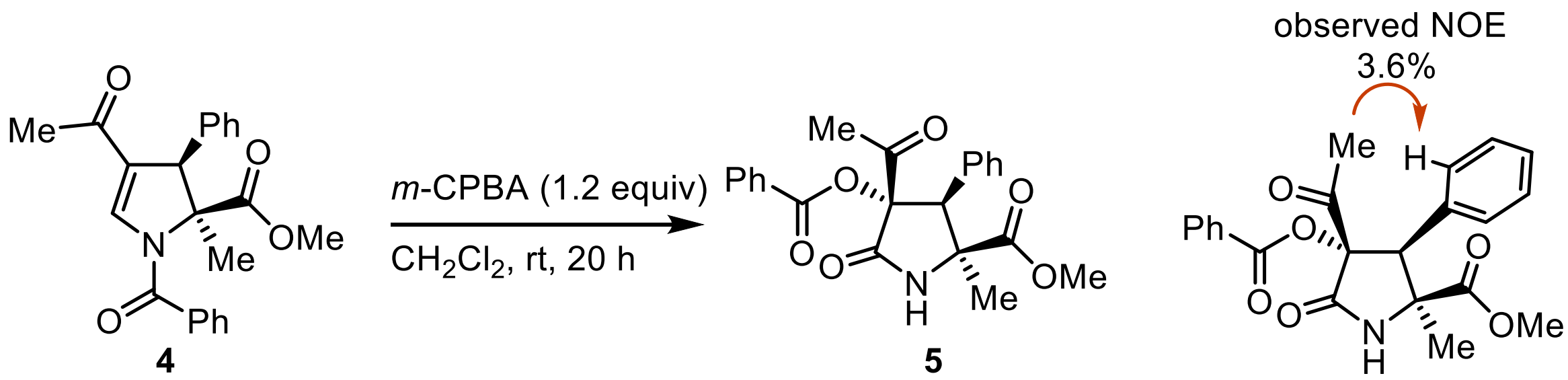

To a solution of **4** (29.0 mg, 0.08 mmol) in CH_2Cl_2 (0.5 mL) was added 3-chloroperoxybenzoic acid (contains ca. 30% water, 17.0 mg, 0.10 mmol) at room temperature. After stirring for 20 hours at room temperature, the reaction mixture was diluted with CH_2Cl_2 (1.0 mL) and then quenched with aqueous $Na_2S_2O_8$ solution (1.0 mL). The aqueous layer was extracted with CH_2Cl_2 (1 mL × 3), and the combined organic layer was dried over $MgSO_4$ and concentrated under reduced pressure. The residue was purified by preparative TLC (EtOAc/hexane 3:1) to give **5** (15.7 mg, 50% yield).

^{1}H NMR (400 MHz, $CDCl_3$) δ 7.88-7.85 (m, 2H), 7.62-7.56 (m, 1H), 7.51-7.46 (m, 2H), 7.33-7.30 (m, 3H), 7.16-7.13 (m, 2H), 4.35 (brs, 1H), 3.63 (s, 1H), 3.40 (s, 3H), 2.04 (s, 3H), 1.94 (s, 3H).
^{13}C NMR (101 MHz, $CDCl_3$) δ 206.0, 173.0, 170.8, 170.1, 133.7, 132.8, 132.2, 129.9, 129.3, 128.8, 128.7, 128.3, 85.6, 70.2, 62.3, 52.4, 27.2, 26.5.
IR(ATR) 3404, 2952, 1743, 1693, 1274, 1100, 910, 726, 697 cm^{-1}.
HRMS (ESI) *m/z*: $[M+Na]^+$ Calcd for $C_{22}H_{21}NO_6Na$ 418.1261; Found. 418.1258.
$[\alpha]_D$ $^{19.7}$ +53.3 (*c* 0.30, $CHCl_3$).

Methyl (2*S*,3*R*)-4-Acetyl-1-benzyl-2-methyl-3-phenyl-2,3-dihydro-1*H*-pyrrole-2-carboxylate (6)

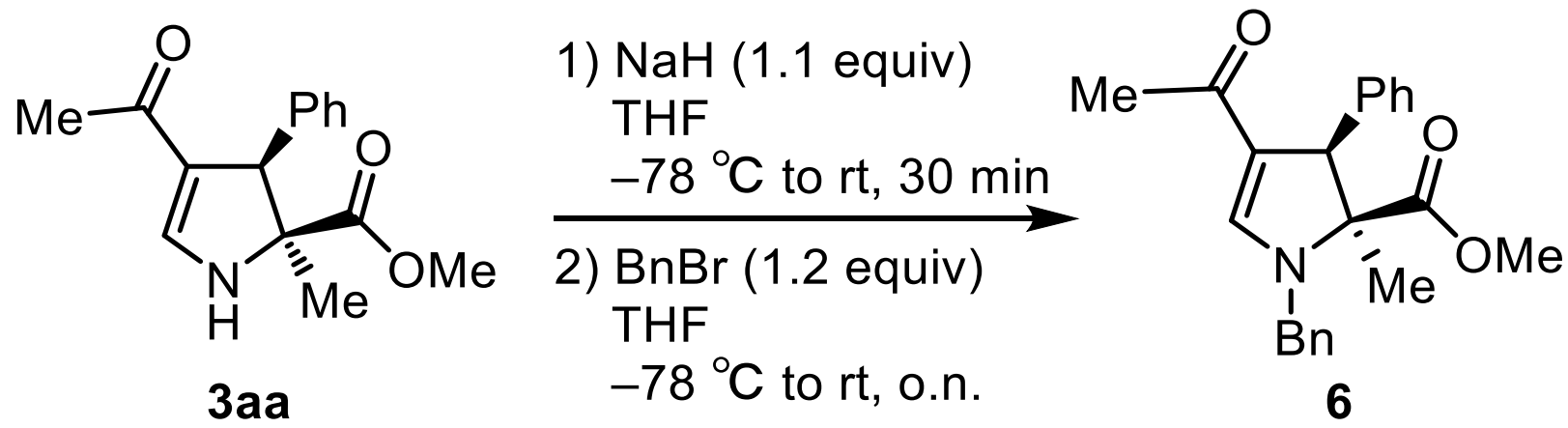

To a suspension of sodium hydroxide (53 mg, 2.2 mmol, 1.1 equiv) in THF (5 mL) was added a solution of **3aa** (520 mg, 2 mmol, 1.0 equiv) in THF (5 mL) at –78 °C. The reaction mixture was stirred at room temperature for 30 minutes. Benzyl bromide (0.3 mL, 2.4 mmol, 1.2 equiv) was added dropwise at –78 °C and the reaction was warmed to room temperature and stirred overnight. The reaction was quenched with saturated aqueous NH_4Cl and extracted with EtOAc three times. The combined organic layer was washed with brine and dried over Na_2SO_4. After concentrating *in vacuo*, the crude product was purified by silica gel column chromatography (EtOAc/hexane 3:7 to 7:3) to give **6** (546 mg, 78% yield) as a pale brown solid.

^{1}H NMR (400 MHz, $CDCl_3$) δ 7.45-7.31 (m, 5H), 7.25-7.21 (m, 2H), 7.19-7.09 (m, 4H), 4.41 (d, *J* 14.4 Hz, 1H), 4.21 (s, 1H), 4.14 (d, *J* = 14.4 Hz, 1H), 3.11 (s, 3H), 1.97 (s, 3H), 1.68 (s, 3H).

^{13}C NMR (101 MHz, $CDCl_3$) δ 190.2, 170.7, 149.7, 138.9, 136.5, 128.8, 128.5, 128.1, 128.0, 127.7, 127.0, 115.5, 78.0, 58.9, 51.5, 49.9, 25.6, 22.9.
IR (ATR) 3029, 2950, 1737, 1574, 1454, 1376, 1210, 1106, 911, 726, 696 cm^{-1}.
HRMS (ESI) *m/z*: $[M+H]^+$ Calcd for $C_{22}H_{24}O_3N$ 350.1751; Found 350.1750.
melting point 85.5-87.2 °C
$[\alpha]_D^{20.6}$ –58.1 (*c* 1.05, $CHCl_3$).

Methyl (2*S*,3*R*,5a*R*,8a*R*,8b*R*)-1-Benzyl-4-((*tert*-butyldimethylsilyl)oxy)-2-methyl-6,8-dioxo-3,7-diphenyl-1,2,3,5,5a,6,7,8,8a,8b-decahydropyrrolo[3,4-*g*]indole-2-carboxylate (7)

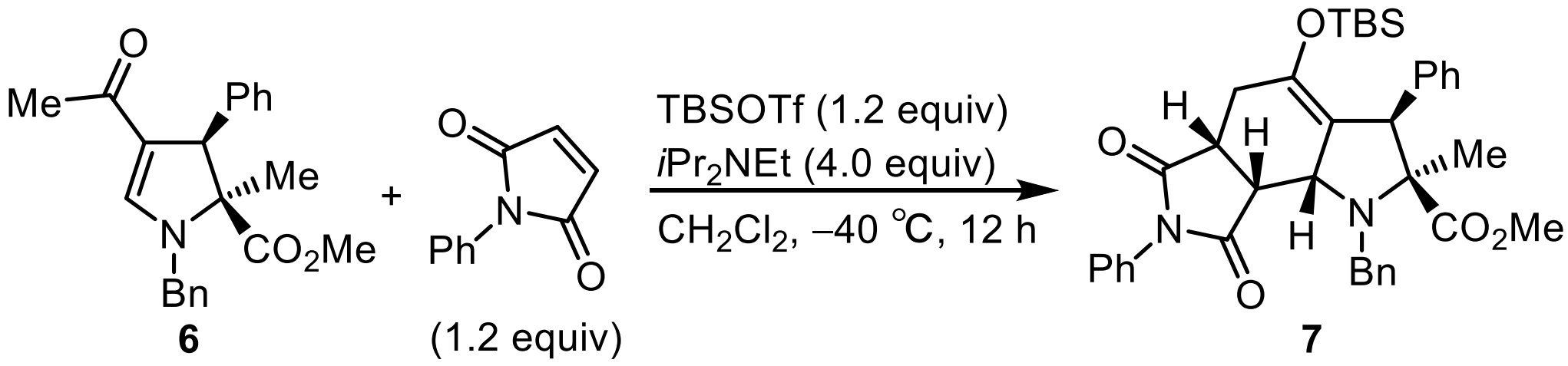

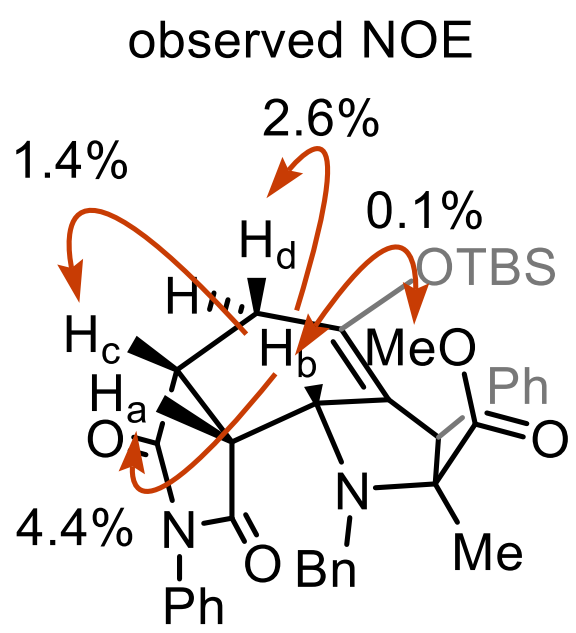

To a solution of **6** (35 mg, 0.1 mmol, 1.0 equiv) in CH_2Cl_2 (2.0 mL, 0.05 M) was added *N*-phenylmaleimide (20.8 mg, 0.12 mmol, 1.2 equiv) and *i*Pr_2NEt (70 µL, 0.4 mmol, 4.0 equiv). The mixture was cooled to –40 °C and added dropwise TBSOTf (28 µL, 0.12 mmol, 1.2 equiv). After stirring for 12 h, the resulting mixture was quenched with saturated aqueous $NaHCO_3$ and extracted with CH_2Cl_2 three times. The combined organic layer was dried over Na_2SO_4, filtered, and concentrated under reduced pressure. The residue was purified by preparative thin-layer chromatography (EtOAc/hexane 3:7) to give **7** (46 mg, 72% yield) as a colorless foamy solid.

^{1}H NMR (400 MHz, $CDCl_3$) δ 7.59 (d, *J* = 7.6 Hz, 2H), 7.47-7.42 (m, 2H), 7.38-7.33 (m, 1H), 7.31-7.26 (m, 2H), 7.23-7.11 (m, 8H), 4.17-4.14 (m, 1H), 3.98 (d, *J* = 14.0 Hz, 1H), 3.86 (d, *J* = 14.0 Hz, 1H), 3.84-3.82 (m, 1H), 3.23 (s, 3H), 3.13 (t, *J* = 8.8 Hz, 1H), 2.87 (dd, *J* = 8.8, 4.8 Hz, 1H), 2.84-2.74 (m, 1H), 2.60 (d, *J* = 15.6 Hz, 1H), 1.43 (s, 3H), 0.43 (s, 9H), –0.12 (s, 3H), –0.26 (s, 3H).
^{13}C NMR (101 MHz, $CDCl_3$) δ 178.0, 174.5, 173.2, 140.1, 139.9, 136.1, 132.1, 129.3, 129.0, 128.3, 128.0, 127.7, 127.1, 127.0, 126.3, 117.3, 77.9, 66.7, 57.3, 54.6, 50.7, 43.8, 41.0, 31.1, 25.1, 22.4, 17.4, –3.8, –3.9.
IR (ATR) 3027, 2930, 2857, 1709, 1496, 1374, 1202, 1163, 832, 747, 700, 690 cm^{-1}.

HRMS (ESI) *m/z*: $[M+H]^+$ Calcd for $C_{38}H_{45}O_5N_2$ 637.3092; Found 637.3086.
melting point 56.7-57.7 °C
[α]$_D$ $^{21.8}$ +7.7 (*c* 1.07, $CHCl_3$).

Methyl (2*S*,3*R*)-1-Benzyl-4-hydroxy-2-methyl-6,11-dioxo-3-phenyl-2,3,6,11-tetrahydro-1*H*-naphtho[2,3-*g*]indole-2-carboxylate (8)

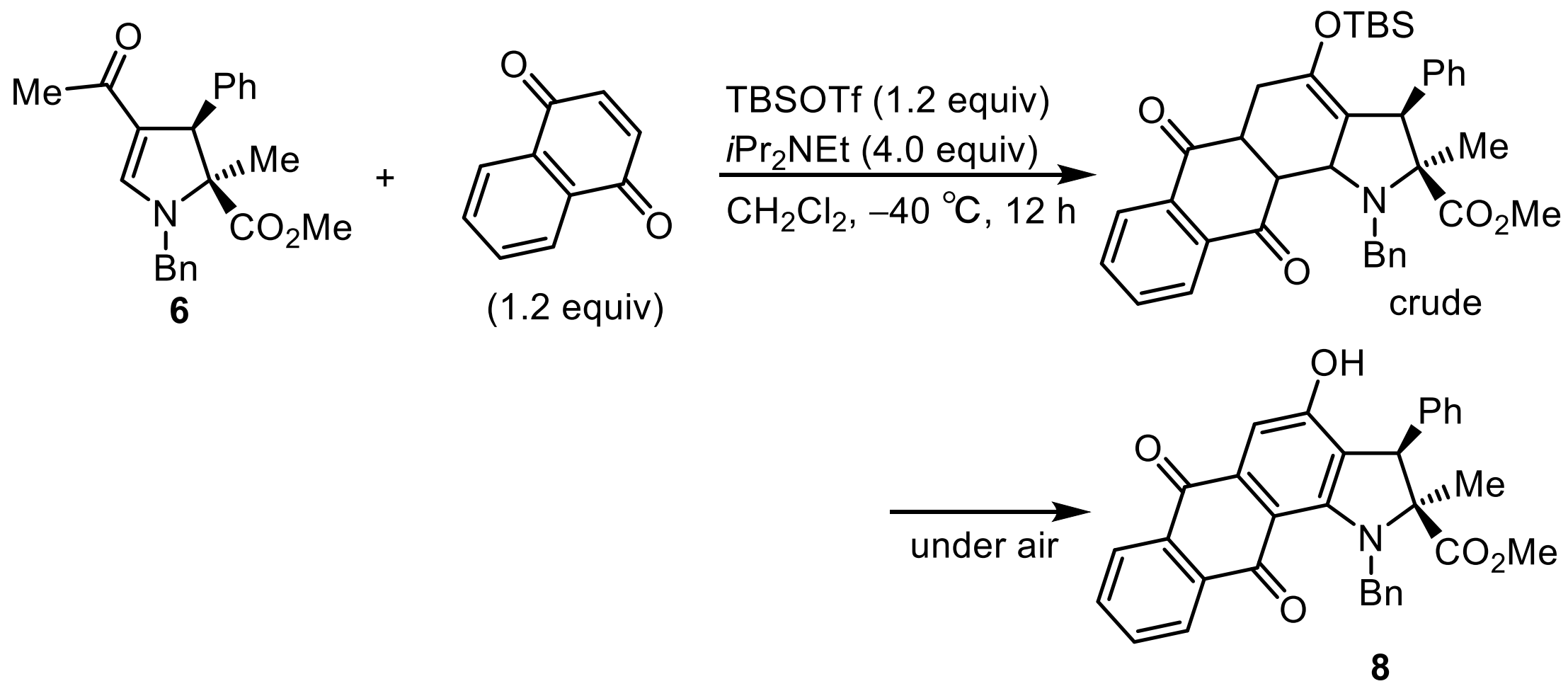

To a solution of compound **6** (35 mg, 0.1 mmol, 1.0 equiv) in CH_2Cl_2 (2.0 mL, 0.05 M) were added naphthoquinone (19.0 mg, 0.12 mmol, 1.2 equiv) and *i*Pr_2NEt (70 μL, 0.4 mmol, 4.0 equiv). The mixture was cooled to –40 °C, and TBSOTf (28 μL, 0.12 mmol, 1.2 equiv) was added dropwise. After stirring for 12 h, the resulting mixture was quenched with saturated aqueous $NaHCO_3$ and extracted with CH_2Cl_2 three times. The organic layer was dried over Na_2SO_4, filtered, and concentrated under reduced pressure. The crude product was oxidized by air during purification with GPC. After evaporation, the residue was further purified by preparative thin-layer chromatography (EtOAc/hexane 1:4) to give **8** (24.4 mg, 48% yield) as a red solid.

^{1}H NMR (400 MHz, $CDCl_3$) δ 8.25-8.16 (m, 2H), 7.84 (d, *J* = 1.2 Hz, 1H), 7.75-7.66 (m, 2H), 7.43-7.27 (m, 8H), 7.24-7.20 (m, 2H), 7.14 (s, 1H), 4.70 (d, *J* = 16.8 Hz, 1H), 4.62 (d, *J* = 1.6 Hz, 1H), 4.22 (d, *J* = 16.8 Hz, 1H), 3.22 (s, 3H), 1.76 (s, 3H).
^{13}C NMR (101 MHz, $CDCl_3$) δ 183.8, 181.8, 171.3, 156.9, 137.4, 133.8, 133.1, 129.2, 128.8, 128.7, 128.2, 127.5, 126.84, 126.79, 126.7, 123.8, 102.9, 79.5, 59.7, 51.8, 49.8, 22.7.
IR (ATR) 3029, 2949, 1735,1666, 1591, 1578, 1494, 1396, 1324, 1280, 1227, 1102, 910, 714, 698 cm^{-1}.
HRMS (ESI) *m/z*: $[M–H]^-$ Calcd for $C_{32}H_{24}O_5N$ 502.1660; Found. 502.1667.
melting point 59.4-64.2 °C
[α]$_D$ $^{20.6}$ +149.0 (*c* 1.83, $CHCl_3$).

S-Ethyl (E)-3-Phenylprop-2-enethioate (S1)

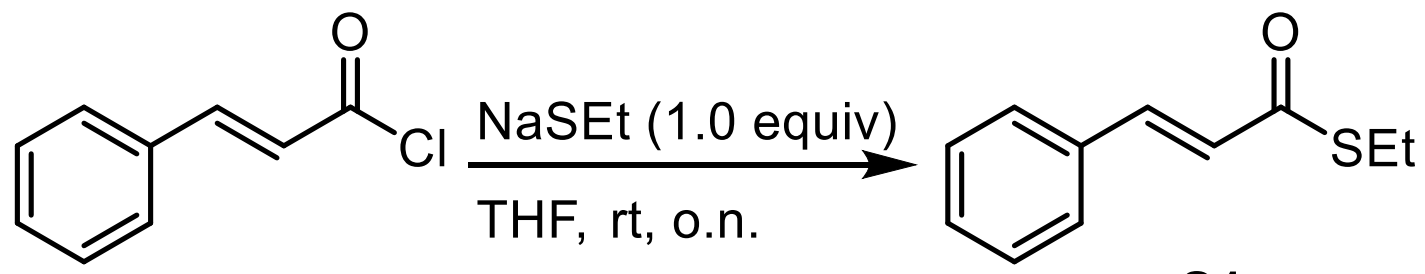

S1

To a solution of (E)-cinnamoyl chloride (4.8 g, 28.5 mmol, 1.2 equiv) in dry THF (100 mL) was added sodium ethanethiolate (2.0 g, 23.8 mmol, 1.0 equiv). After stirring at room temperature overnight, water was added to the reaction mixture. The aqueous layer was extracted with EtOAc three times and the combined organic layer was dried over Na_2SO_4, filtered, and concentrated under reduced pressure. The residue was purified by silica gel column chromatography (EtOAc/hexane 1:49) to give the product **S1** as a yellow oil (2.5 g, 55% yield).

^{1}H NMR (400 MHz, $CDCl_3$) δ 7.60 (d, J = 15.6 Hz, 1H), 7.56-7.51 (m, 2H), 7.43-7.35 (m, 3H), 6.71 (d, J = 15.6 Hz, 1H), 3.02 (q, J = 7.2 Hz, 2H), 1.32 (t, J = 7.2 Hz, 3H).
^{13}C NMR (101 MHz, $CDCl_3$) δ 189.9, 140.2, 134.1, 130.5, 128.9, 128.3, 125.1, 23.4, 14.8.
Spectroscopic data were consistent with those reported in the literature.[6]

Ethyl (E)-5-Oxo-7-phenylhept-6-enoate (S2)

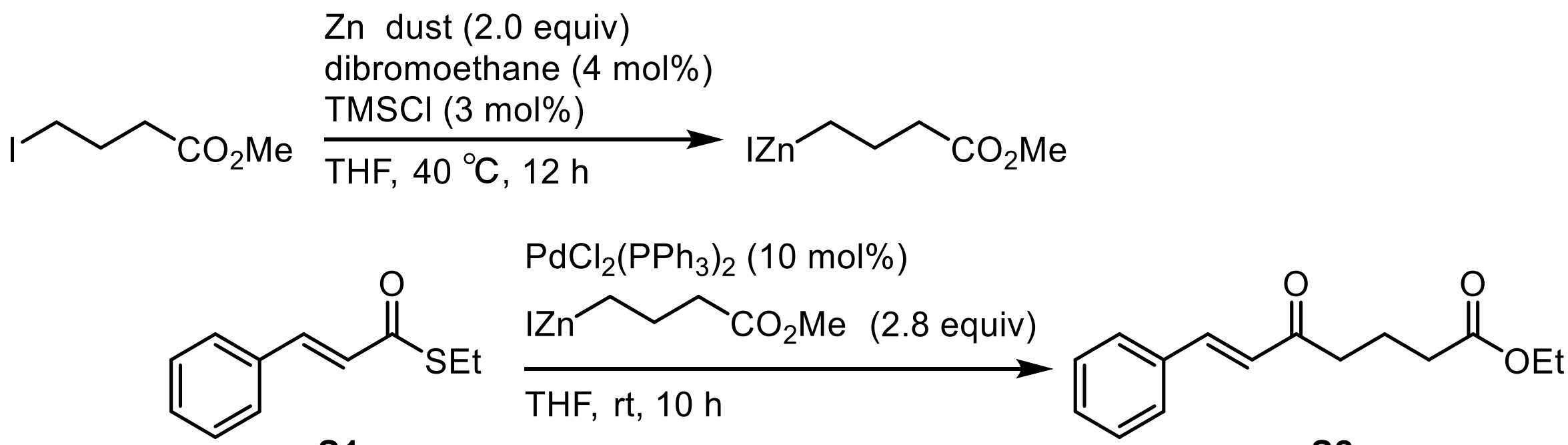

S1 **S2**

Activated zinc powder (6.5 g, 100 mmol, 2.0 equiv) was suspended in anhydrous THF (5 mL) under N_2 atmosphere, and then 1,2-dibromoethane (0.17 mL, 2.0 mmol, 4 mol%) was added. The mixture was heated at 65 °C for 5 min. After the mixture was cooled to room temperature, TMSCl (0.2 mL, 1.5 mmol, 3 mol%), was added, and the mixture was stirred for another 15 min before the solution of methyl 4-iodobutanoate (50 mmol) in anhydrous THF (25 mL) was added dropwise over 30 min. The resulting mixture was stirred for 12 h at 40 °C to give the alkylzinc iodide as a THF solution.

To a suspension of $PdCl_2(PPh_3)_2$ (1.26 g, 1.8 mmol, 10 mol%), **S1** (3.46 g, 18 mmol, 1.0 equiv) in THF (70 mL) was added the prepared alkylzinc iodide THF solution at room temperature and stirred for 10 h. The resulting mixture was passed through a pad of celite. The filtrate was concentrated under reduced pressure. The residue was purified by silica gel column chromatography (EtOAc/hexane 3:97 to 13:87) to give **S2** as a yellow solid (2.7 g, 60% yield).

^{1}**H NMR** (400 MHz, $CDCl_3$) δ 7.59-7.53 (m, 3H), 7.41-7.39 (m, 3H), 6.73 (d, *J* = 16.0 Hz, 1H), 4.14 (q, *J* = 6.8 Hz, 2H), 2.76 (t, *J* = 7.2 Hz, 2H), 2.40 (t, *J* = 7.2 Hz, 2H), 2.02 (tt, *J* = 7.2, 7.2 Hz, 2H), 1.27 (t, *J* = 6.8 Hz, 3H).
^{13}**C NMR** (101 MHz, $CDCl_3$) δ 199.5, 173.3, 142.7, 134.4, 130.5, 128.9, 128.3, 126.1, 60.4, 39.6, 33.4, 19.3, 14.2.
Spectroscopic data were consistent with those reported in the literature.[7]

(*E*)-5-Oxo-7-phenylhept-6-enoic Acid (S3)

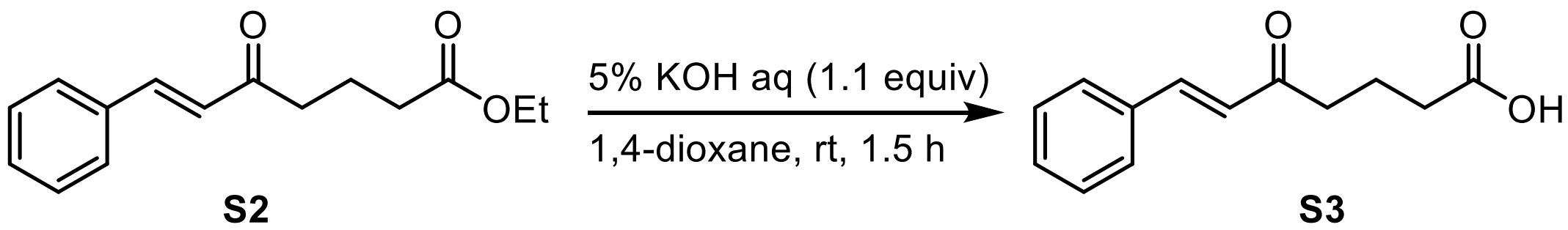

To a solution of **S2** (2.0 g, 8.12 mmol, 1.0 equiv) in 1,4-dioxane (9.5 mL) was added 5% aqueous KOH (9.5 mL, 8.93 mmol, 1.1 equiv) at 0 °C. After stirring at room temperature for 1.5 h, the resulting mixture was washed with Et_2O three times. The aqueous layer was acidified with 1 M HCl and extracted with DCM three times. The organic layer was dried over Na_2SO_4, filtered and concentrated under reduced pressure. The residue was purified by silica gel column chromatography (EtOAc) to give **S3** as a yellow solid (1.2 g, 69% yield).

^{1}**H NMR** (400 MHz, $CDCl_3$) δ 7.60-7.52 (m, 3H), 7.43-7.37 (m, 3H), 6.74 (d, *J* = 16.0 Hz, 1H), 2.79 (t, *J* = 7.2 Hz, 2H), 2.48 (t, *J* = 7.2 Hz, 2H), 2.03 (tt, *J* = 7.2, 7.2 Hz, 2H).
^{13}**C NMR** (101 MHz, $CDCl_3$) δ 199.4, 178.4, 142.9, 134.4, 130.6, 129.0, 128.3, 126.0, 39.4, 32.9, 19.0.
IR (ATR) 2949, 1698, 1650, 1613, 1450, 1412, 1284, 1208, 1106, 976, 756, 689 cm^{-1}.
HRMS (ESI) *m/z*: $[M–H]^-$ Calcd for $C_{13}H_{13}O_3$ 217.0870; Found. 217.0871.
melting point 104.0-105.7 °C

Methyl (*E*)-3-(((*E*)-5-Oxo-7-phenylhept-6-enoyl)oxy)oct-2-enoate (S4)

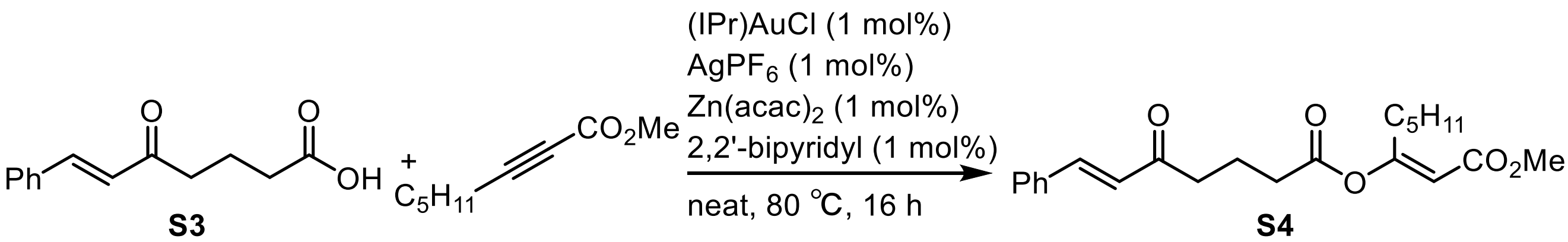

In a nitrogen-filled glove box, **S3** (546 mg, 2.5 mmol), (IPr)AuCl (15.5 mg, 0.025 mmol, 1 mol%), $Zn(acac)_2$ (6.6 mg, 0.025 mmol, 1 mol%), $AgPF_6$ (6.4 mg, 0.025 mmol, 1 mol%) and 2,2'-bipyridyl (3.9 mg, 0.025 mmol, 1 mol%) were placed in a vial containing a magnetic stirring bar. After addition of methyl 2-octynoate (3.0 mL, 18 mmol, 7.2 equiv), the reaction vial was sealed with a screw-cap and taken out of the glove box and stirred at 80 °C for 16 hours. The resulting mixture was passed through

a pad of silica and the filtrate was concentrated *in vacuo*. The crude product was purified by silica-gel column chromatography (EtOAc/hexane 3:97 to 1:9) to give **S4** as a pale yellow liquid (456 mg, 49% yield).

^{1}H NMR (400 MHz, $CDCl_3$) δ 7.59 (d, J = 16.8 Hz,1H), 7.57-7.53 (m, 2H), 7.41-7.37 (m, 3H), 6.75 (d, J = 16.8 Hz, 1H), 5.61-5.60 (m, 1H), 3.65 (s, 3H), 2.85 (t, J = 7.2 Hz, 2H), 2.64 (t, J = 7.2 Hz, 2H), 2.26 (t, J = 8.0 Hz, 2H), 2.10 (tt, J = 7.2, 7.2 Hz, 2H), 1.56-1.45 (m, 2H), 1.34-1.27 (m, 4H), 0.91-0.86 (m, 3H).
^{13}C NMR (101 MHz, $CDCl_3$) δ 199.6, 170.3, 164.3, 163.8, 142.7, 134.5, 130.4, 128.9, 128.2, 126.2, 106.7, 51.2, 39.4, 35.3, 33.1, 31.0, 25.4, 22.3, 19.0, 13.9.
IR (ATR) 2953, 1761, 1721, 1691, 1663, 1611, 1450, 1435, 1202, 1099, 1028, 978, 748, 690 cm^{-1}.
HRMS (ESI) *m/z*: $[M+Na]^+$ Calcd for $C_{22}H_{28}O_5Na$ 395.1829; Found 395.1827.

Methyl (*E*)-3-(((4*Z*,6*E*)-7-phenyl-5-((triisopropylsilyl)oxy)hepta-4,6-dienoyl)oxy)oct-2-enoate (9)

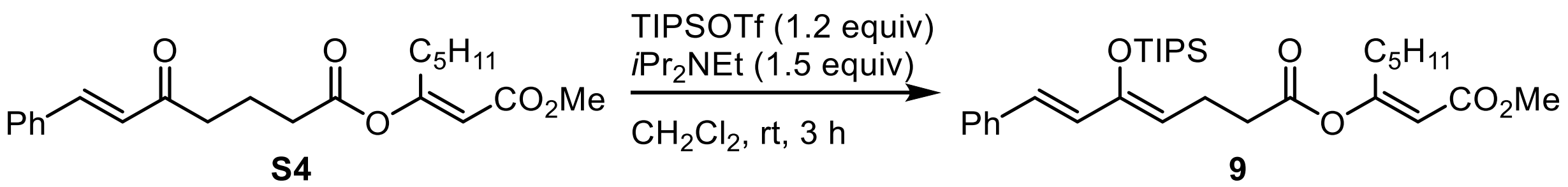

TIPSOTf (650 µL, 2.4 mmol, 1.2 equiv) was added to a solution of **S4** (745 mg, 2 mmol) in dry CH_2Cl_2 (8 mL, 0.25 M) at 0 °C and the solution was stirred for 10 minutes. DIPEA (510 µL, 3.0 mmol, 1.5 equiv) was added dropwise and the mixture was allowed to warm to room temperature. After stirring for 3 h, the resulting mixture was quenched with saturated aqueous NH_4Cl (10 mL). The aqueous layer was extracted with CH_2Cl_2 three times and combined organic layer was dried over Na_2SO_4, filtered, and concentrated under reduced pressure. The residue was purified by silica gel column chromatography (EtOAc/hexane 0:100 to 3:97) to give **9** as a pale yellow oil (835 mg, 79% yield).

^{1}H NMR (400 MHz, $CDCl_3$) δ 7.39-7.29 (m, 4H), 7.25-7.19 (m, 1H), 6.73 (d, J = 16.0 Hz, 1H), 6.56 (d, J = 16.0 Hz, 1H), 5.60 (s, 3H), 4.96-4.91 (m, 1H), 3.66 (s, 3H), 2.66-2.57 (m, 4H), 2.26 (t, J = 7.6 Hz, 2H), 1.55-1.47 (m, 2H), 1.34-1.21 (m,7H), 1.16 (s, 9H), 1.14 (s, 6H), 1.05 (s, 3H), 0.90-0.85 (m, 3H).
^{13}C NMR (101 MHz, $CDCl_3$) δ 170.1, 164.3, 163.9, 150.4, 137.0, 128.6, 127.5, 127.41, 127.39, 126.40, 112.1, 106.8, 51.2, 35.4, 34.0, 31.1, 25.4, 22.3, 21.5, 18.1, 17.7, 13.9, 13.8, 12.3, –0.01.
IR (ATR) 3075, 3024, 1607, 1574, 1496, 1445, 1221, 937, 727, 688 cm^{-1}.
HRMS (ESI) *m/z*: $[M+H]^+$ Calcd for $C_{31}H_{49}O_5Si$ 529.3344; Found. 529.3343.

Methyl (2*S*,3*R*)-4-Acetyl-2-methyl-3-phenyl-1-((4*Z*,6*E*)-7-phenyl-5-((triisopropylsilyl)oxy)hepta-4,6-dienoyl)-2,3-dihydro-1*H*-pyrrole-2-carboxylate (10)

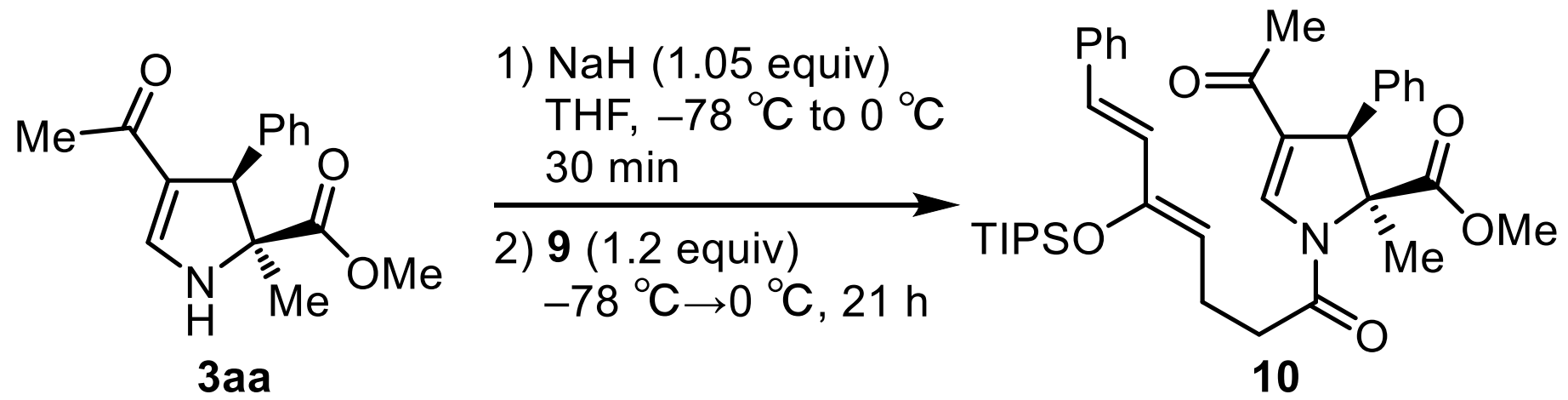

To a suspension of sodium hydroxide (7.5 mg, 0.32 mmol, 1.05 equiv) in THF (1.5 mL) was added a solution of **3aa** (71 mg, 0.28 mmol, 1.0 equiv) in THF (0.75 mL) at –78 °C. The reaction mixture was stirred at 0 °C for 0.5 h. A solution of compound **9** (175 mg, 0.33 mmol, 1.2 equiv) in THF (0.5 mL) was added dropwise at –78 °C and the reaction was warmed to 0 °C and stirred for 21 h. The reaction was quenched with saturated aqueous $NaHCO_3$ and extracted with EtOAc three times. The combined organic layer was washed with brine and dried over Na_2SO_4. After concentrating *in vacuo*, the crude product was purified by silica gel column chromatography (EtOAc/hexane (1% NEt_3) 1:9 to 2:8) to give **10** (101 mg, 60% yield) as a colorless foamy solid.

^{1}H NMR (400 MHz, $CDCl_3$) δ 7.64 (s, 1H), 7.42-7.31 (m, 5H), 7.28-7.21 (m, 4H), 7.05 (br, 2H), 6.76 (d, *J* = 15.6 Hz, 1H), 6.56 (d, *J* = 16.0 Hz, 1H), 4.96 (t, *J* = 6.8 Hz, 1H), 4.20 (s, 1H), 3.01 (s, 3H), 2.71-2.51 (m, 4H), 2.19 (s, 3H), 1.84 (s, 3H), 1.36-1.05 (m, 21H).
^{13}C NMR (101 MHz, $CDCl_3$) δ 193.1, 169.6, 168.8, 150.8, 139.3, 137.1, 136.8, 128.6, 127.9, 127.8, 127.6, 127.5, 127.2, 126.4, 123.3, 111.5, 74.5, 58.7, 51.6, 34.1, 26.9, 24.2, 21.4, 18.0, 13.7
IR (ATR) 2946, 2867, 1744, 1656, 1602, 1408, 1363, 1217, 1117, 1015, 959, 882, 777, 753, 681 cm^{-1}.
HRMS (ESI) *m/z*: $[M+H]^+$ Calcd for $C_{37}H_{50}NO_5Si$ 616.3453; Found. 616.3446.
melting point 49.6-52.7 °C
$[\alpha]_D^{20.7}$ +50.3 (*c* 0.98, $CHCl_3$).

Intramolecular Diels-Alder reaction products:
β-*endo* product (11) and α-*exo* product (12)

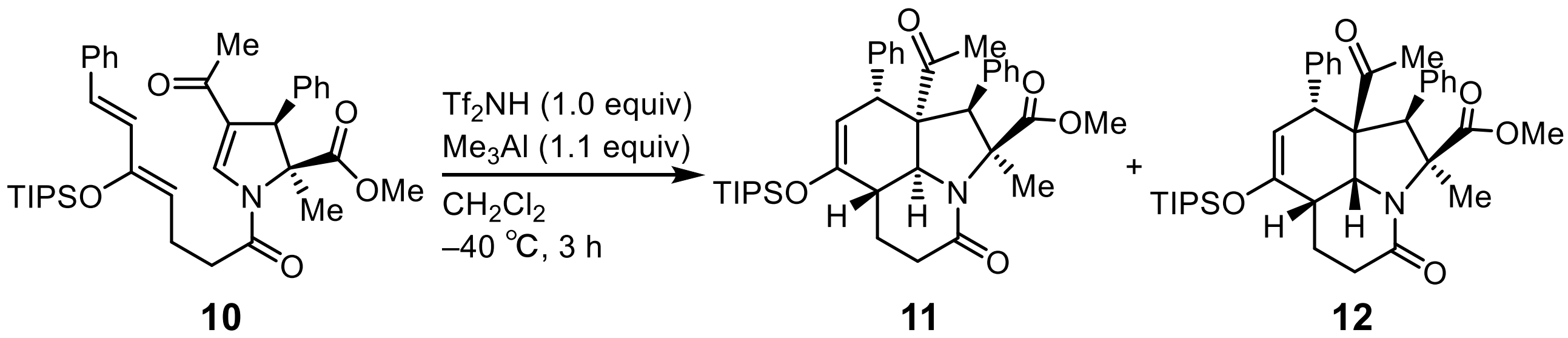

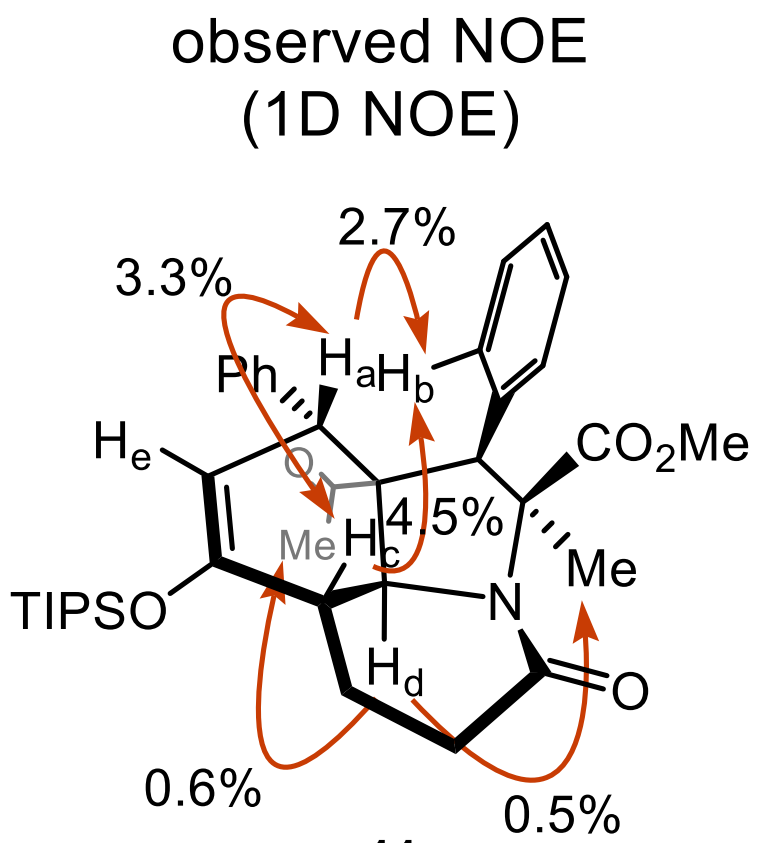

11

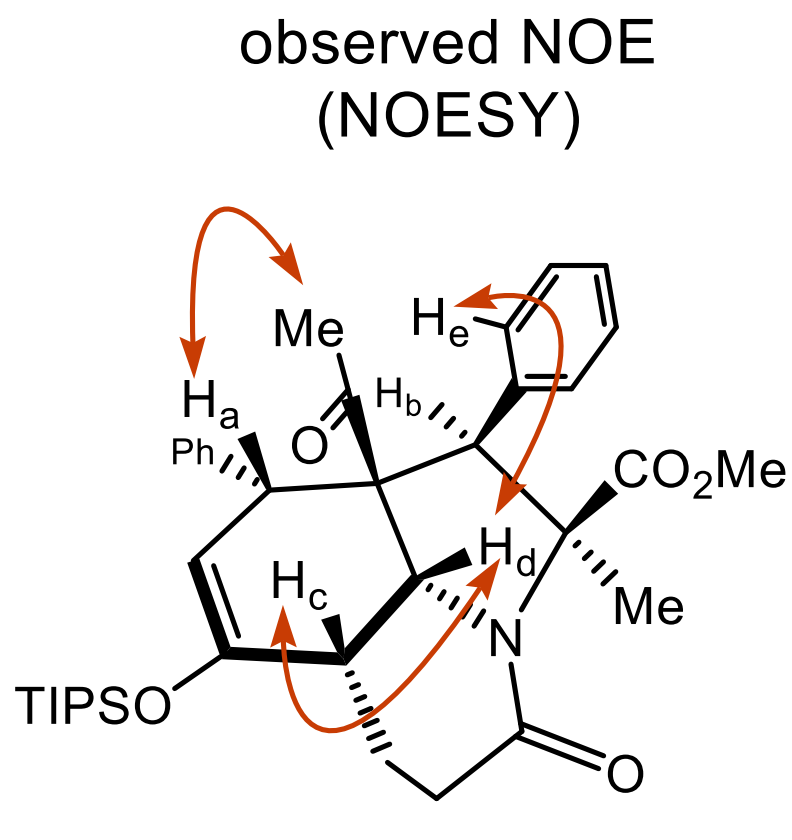

12

Me_2AlNTf_2 was prepared according to a reported procedure.[43] Trimethylaluminum (80 μL, 0.11 mmol, 1.1 equiv) was added to Tf_2NH (28 mg, 0.1 mmol, 1.0 equiv) in CH_2Cl_2 (0.6 mL, 0.33 M) at room temperature, and the solution was stirred for 30 minutes. The reaction system was cooled to –78 °C and the solution of **10** (61.6 mg, 0.1 mmol, 1.0 equiv) in CH_2Cl_2 (1.4 mL) was added. The reaction mixture was warmed to –40 °C and stirred for 3 h. The reaction was quenched with triethylamine (2.0 mL) at –78 °C and filtered through a pad of silica gel. After removal of the solvents *in vacuo*, the residue was purified by preparative thin-layer chromatography (EtOAc/hexane 9:11) to give **11** (39 mg, 63% yield) as a colorless foamy solid and **12** (11 mg, 18% yield) as a colorless foamy solid.

β-*endo* Product (11)

^{1}H NMR (400 MHz, $CDCl_3$) δ 7.53-7.47 (m, 1H), 7.42-7.29 (m 3H), 7.14-7.04 (m, 4H), 6.28 (d, *J* 7.2 Hz, 2H), 4.75 (t, *J* = 2.8 Hz, 1H), 4.40 (d, *J* = 10.0 Hz, 1H), 4.15 (s, 1H), 4.04 (s, 1H), 3.17-3.09 (m, 1H), 3.05 (s, 3H), 2.71-2.54 (m, 2H), 2.50-2.41 (m, 1H), 2.15-2.02 (m, 1H), 1.81 (s, 3H), 1.55 (s, 3H), 1.29-1.03 (m, 2H).

^{13}C NMR (101 MHz, $CDCl_3$) δ 209.3, 170.4, 169.6, 151.6, 140.1, 136.0, 133.3, 132.8, 129.1, 128.2, 128.1, 127.9, 127.2, 127.1, 104.6, 67.4, 66.1, 60.7, 51.4, 44.9, 39.5, 31.7, 30.8, 23.2, 22.5, 18.1, 18.0, 12.6.

IR (ATR) 2946, 2867, 1735, 1699, 1660, 1603, 1457, 1355, 1205, 909, 882, 755, 731, 702, 687 cm^{-1}.

HRMS (ESI) *m/z*: $[M+Na]^+$ Calcd for $C_{37}H_{49}NO_5SiNa$ 638.3272; Found. 638.3267.

melting point 80.8-83.2 °C

$[\alpha]_D$ $^{21.8}$ +40.3 (*c* 0.85, $CHCl_3$).

α-*exo* Product (12)

^{1}H NMR (400 MHz, $CDCl_3$) δ 7.44-7.38 (m, 4H), 7.35-7.29 (m, 1H), 7.24-7.22 (m, 3H), 7.03-6.98 (m, 2H), 5.03 (d, *J* = 7.2 Hz, 1H), 4.84 (dd, *J* = 6.0, 1.2 Hz, 1H), 3.99 (d, *J* = 6.0 Hz, 1H), 3.67 (s, 1H), 3.21 (dd, *J* = 15.2, 8.0 Hz, 1H), 2.99 (s, 3H), 2.56-2.49 (m, 1H), 2.41-2.33 (m, 2H), 2.31-2.21 (m, 1H), 1.60 (s, 3H), 1.23-1.03 (m, 21H), 0.61 (s, 3H).

^{13}**C NMR** (101 MHz, $CDCl_3$) δ 207.1, 173.5, 171.7, 152.4, 140.8, 137.6, 131.8, 129.5, 128.5, 127.9, 127.8, 101.4, 73.5, 64.2, 63.2, 59.0, 51.7, 45.7, 34.9, 32.3, 27.8, 23.8, 21.1, 18.0, 12.7.
IR (ATR) 2946, 2867, 1736, 1703, 1667, 1456, 1217, 1172, 883, 761, 730, 702, 684 cm^{-1}.
HRMS (ESI) *m/z*: $[M+Na]^+$ Calcd for $C_{37}H_{49}NO_5SiNa$ 638.3272; Found. 638.3267.
melting point 79.0-82.8 °C
$[\alpha]_D^{22.0}$ +94.6 (*c* 1.1, $CHCl_3$).

Single crystal XRD

A crystal of **3ac** was prepared by vapor diffusion method with EtOAc/hexane. The crystal was mounted with liquid paraffin on a MiTeGen MicroMounts and transferred to the goniometer in a nitrogen stream at 133 K. Measurement was made on a RIGAKU XtaLAB Synergy-DW system with 1.2 kW PhotonJet-DW microfocus rotating anode using graphite monochromated Cu-K_a radiation (λ = 1.54184 Å) and HyPix-6000HE detector. Cell parameters were determined and refined, and raw frame data were integrated using CrysAlis^{Pro} (Agilent Technologies, 2010). The structures were solved by direct methods with SHELXT[8]and refined by full-matrix least-squares techniques against F^2 with SHELXL-2018/3[9] by using Olex2 software package.[10] The non-hydrogen atoms were anisotropically refined, and hydrogen atoms were placed using AFIX instructions.

Crystal data and structure refinement parameters are given in Table S4. The Mercury program was used to draw the molecular structures in Figure S2.[11]

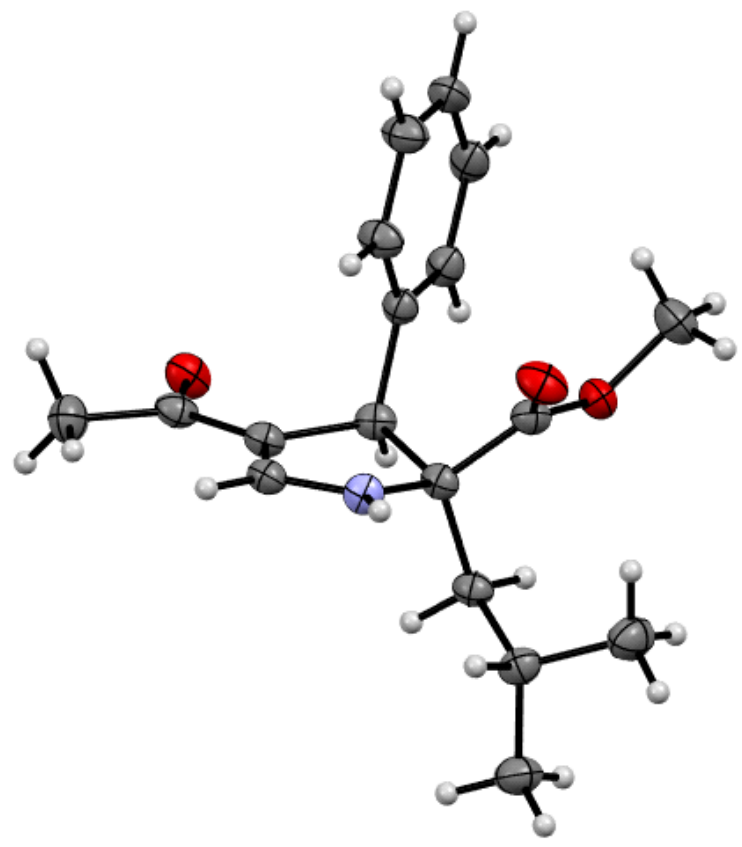

3ac

Figure S2. ORTEP drawings showing 50% probability thermal ellipsoids.

Table S4. Crystal Data and Data Collection Parameters.

	3ac
empirical formula	$C_{18}H_{23}NO_3$
CCDC number	2530817
formula weight	301.37
crystal system	tetragonal
space group	$P4_12_12$ (#92)
a, Å	14.21980(10)
b, Å	14.21980(10)
c, Å	16.38090(10)
α, deg.	90
β, deg.	90
γ, deg.	90
V, $Å^3$	3312.26(5)
Z	8
Dcalcd, g/cm^3	1.209
radiation, mm^{-1}	Cu-*Kα*: 1.54184
T, K	133(2)
crystal size, mm	0.14 × 0.04 × 0.02
2*θ* range for data collection (deg.)	8.234 to 152.518
no. of reflections measured	55816
unique data (R_{int})	3445 (0.0388)
data/restraints/parameters	3445 / 0 / 203
*R*1 (*I*>=2σ (*I*))	0.0288
*wR*2 (*I*>=2σ (*I*))	0.0715
*R*1 (all data)	0.0295
*wR*2 (all data)	0.0719
GOF on F^2	1.086
Flack parameter	–0.03(4)

a) $R1=(\Sigma||Fo|-|Fc||)/(\Sigma|Fo|)$ b) $wR2=[\{\Sigma w(Fo^2-Fc^2)^2\}]/(\Sigma w(Fo^4)\}]^{1/2}$

References

1. S. Sakai, K. Uchiyama, K. Imai, K. Yato, M. Onizawa, K. Higashida, H. Sato, Y. Aoyama, Y. Shimizu and M. Sawamura, *ACS Catal.*, 2025, **15**, 16039–16051.
2. N. Elders, R. F. Schmitz, F. J. J. de Kanter, E. Ruijter, M. B. Groen and R. V. A. Orru, *J. Org. Chem.*, 2007, **72**, 6135–6142.
3. J.-D. Chai and M. Head-Gordon, *Phys. Chem. Chem. Phys.*, 2008, **10**, 6615–6620.
4. M. J. Frisch, G. W. Trucks, H. B. Schlegel, G. E. Scuseria, M. A. Robb, J. R. Cheeseman, G. Scalmani, V. Barone, G. A. Petersson, H. Nakatsuji, X. Li, M. Caricato, A. V. Marenich, J. Bloino, B. G. Janesko, R. Gomperts, B. Mennucci, H. P. Hratchian, J. V. Ortiz, F. Izmaylov, J. L. Sonnenberg, D. Williams-Young, F. Ding, F. Lipparini, F. Egidi, J. Goings, B. Peng, A. Petrone, T. Henderson, D. Ranasinghe, V. G. Zakrzewski, J. Gao, N. Rega, G. Zheng, W. Liang, M. Hada, M. Ehara, K. Toyota, R. Fukuda, J. Hasegawa, M. Ishida, T. Nakajima, Y. Honda, O. Kitao, H. Nakai, T. Vreven, K. Throssell, J. A. Montgomery Jr., J. E. Peralta, F. Ogliaro, M. J. Bearpark, J. J. Heyd, E. N. Brothers, K. N. Kudin, V. N. Staroverov, T. A. Keith, R. Kobayashi, J. Normand, K. Raghavachari, A. P. Rendell, J. C. Burant, S. S. Iyengar, J. Tomasi, M. Cossi, J. M. Millam, M. Klene, C. Adamo, R. Cammi, L. R. Martin, K. Morokuma, O. Farkas, J. B. Foresman and D. J. Fox, *Gaussian 16, Revision C.01*, Gaussian, Inc., Wallingford CT, 2016.
5. S. Maeda, Y. Harabuchi, Y. Sumiya, M. Takagi, K. Suzuki, K. Sugiyama, Y. Ono, M. Hatanaka, Y. Osada, T. Taketsugu, K. Morokuma and K. Ohno, *Global Reaction Route Mapping (GRRM) Program*, version 17-A01.
6. R. D. Mazery, M. Pullez, F. López, S. R. Harutyunyan, A. J. Minnaard and B. L. Feringa, *J. Am. Chem. Soc.*, 2005, **127**, 9966–9967.
7. P. Das and A. T. Hamme, *Tetrahedron Lett.*, 2017, **58**, 1086–1089.
8. G. M. Sheldrick, Acta Crystallogr., Sect. A: Found. Adv., 2015, **71**, 3–8.
9. G. M. Sheldrick, Acta Crystallogr., Sect. C: Struct. Chem., 2015, **71**, 3–8.
10. O. V. Dolomanov, L. J. Bourhis, R. J. Gildea, J. A. K. Howard and H. Puschmann, *J. Appl. Crystallogr.*, 2009, **42**, 339–341.
11. C. F. Macrae, I. Sovago, S. J. Cottrell, P. T. A. Galek, P. McCabe, E. Pidcock, M. Platings, G. P. Shields, J. S. Stevens, M. Towler and P. A. Wood, *J. Appl. Crystallogr.*, 2020, **53**, 226–235.

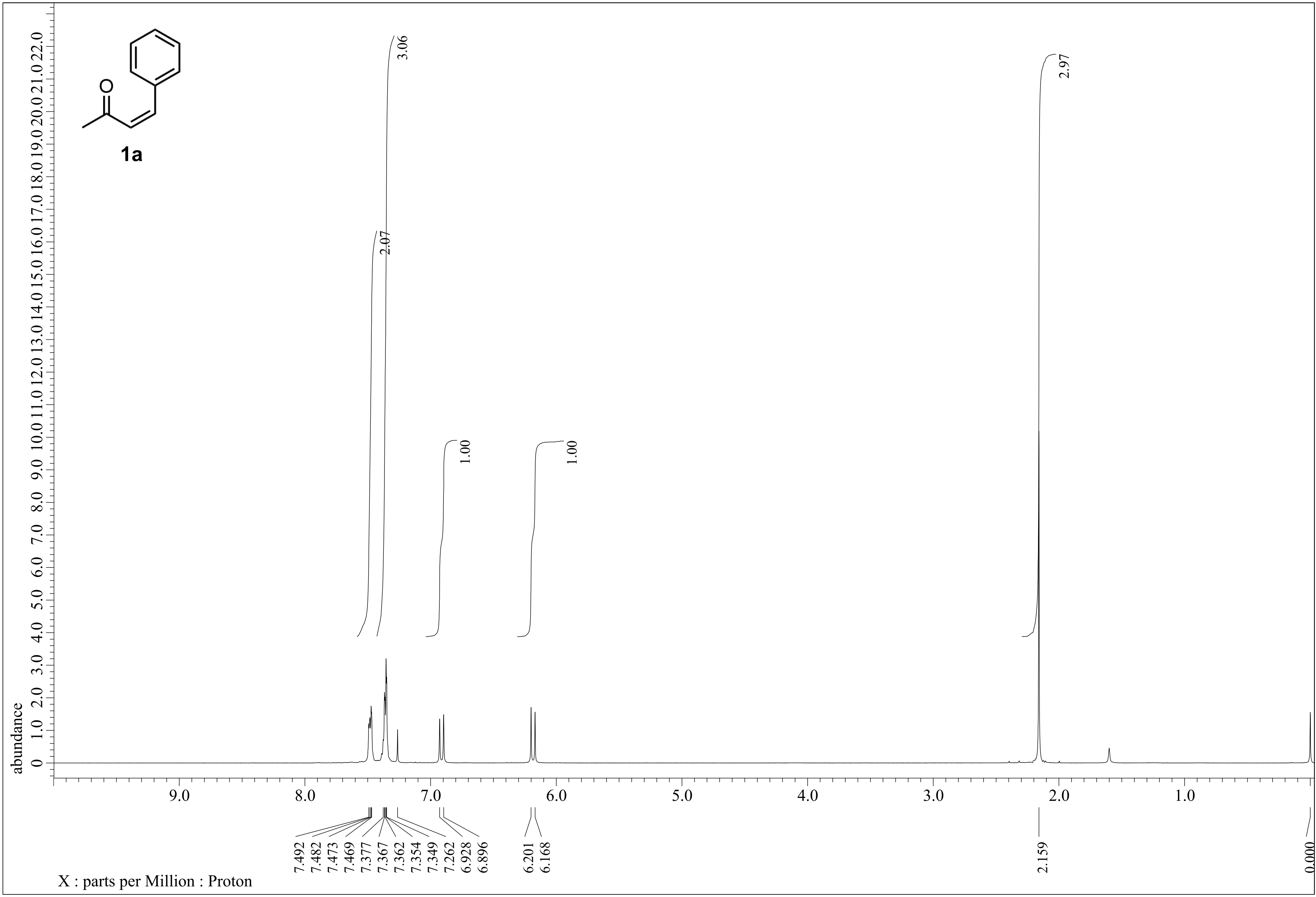
1a
abundance
X : parts per Million : Proton
9.0
8.0
7.0
6.0
5.0
4.0
3.0
2.0
1.0
3.06
2.07
1.00
1.00
2.97
7.492
7.482
7.473
7.469
7.377
7.367
7.362
7.354
7.349
7.262
6.928
6.896
6.201
6.168
2.159
0.000

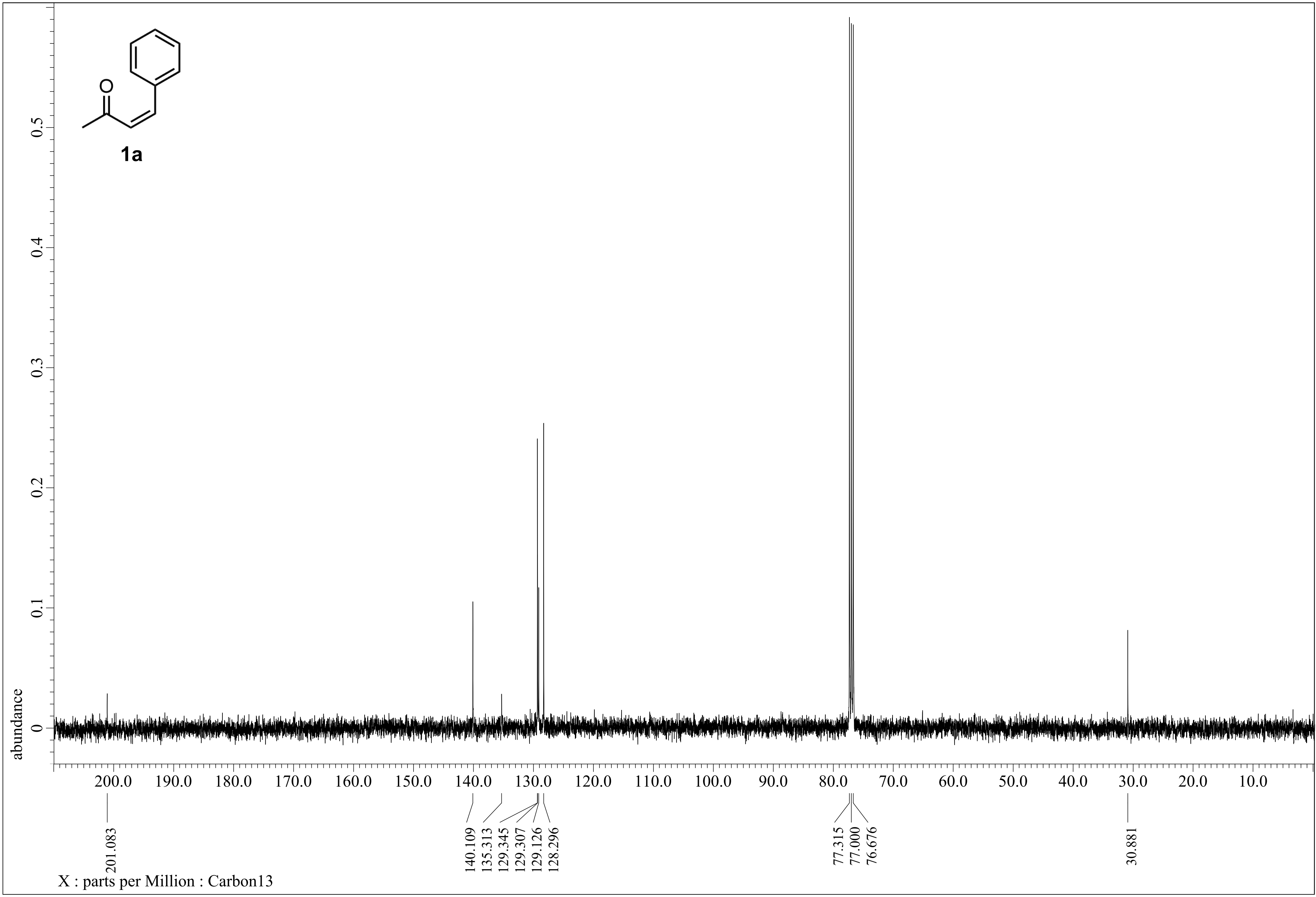
1a
abundance
0
0.1
0.2
0.3
0.4
0.5
200.0
190.0
180.0
170.0
160.0
150.0
140.0
130.0
120.0
110.0
100.0
90.0
80.0
70.0
60.0
50.0
40.0
30.0
20.0
10.0
201.083
140.109
135.313
129.345
129.307
129.126
128.296
77.315
77.000
76.676
30.881
X : parts per Million : Carbon13

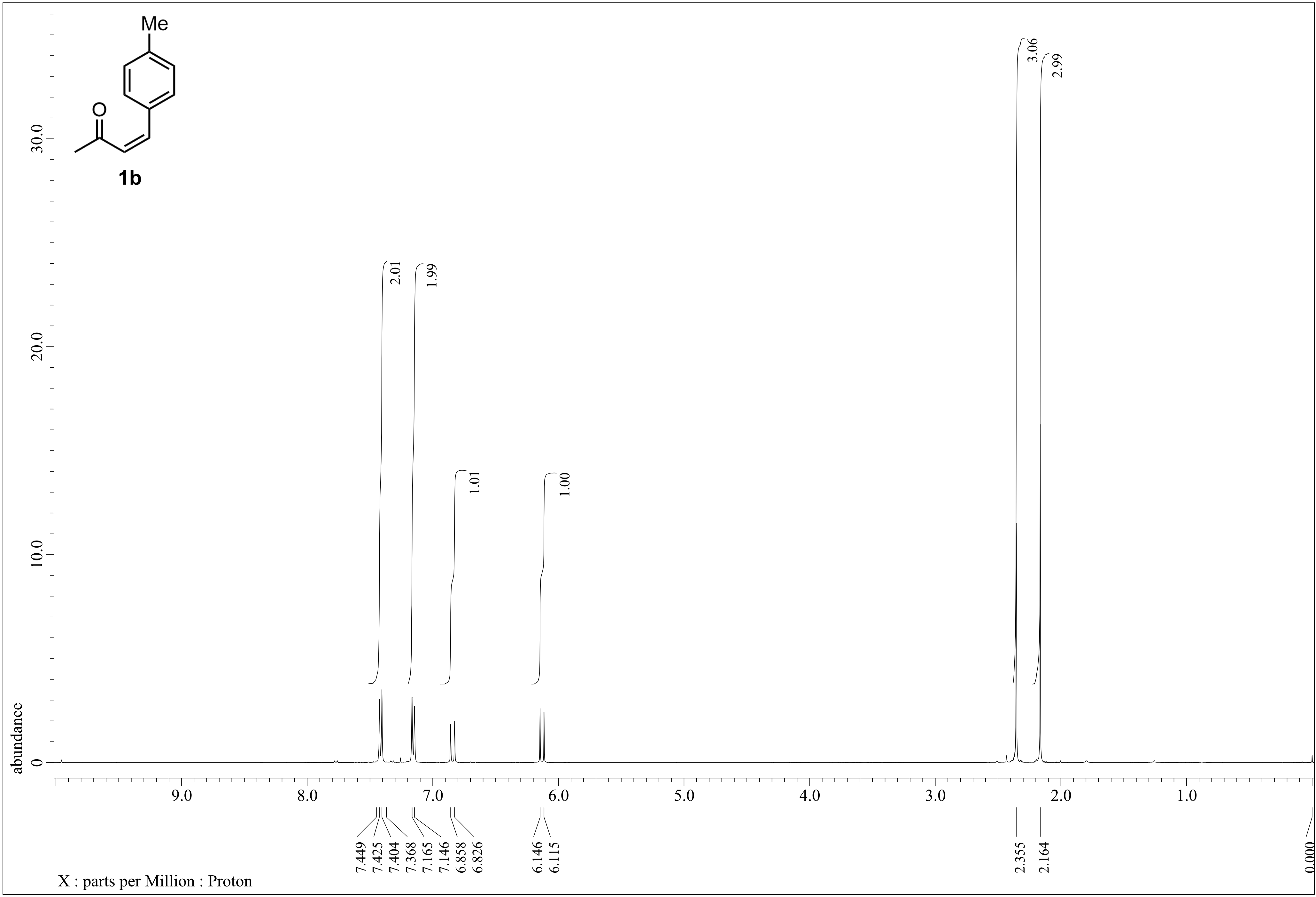
Me
O
1b
30.0
20.0
10.0
0
abundance
2.01
1.99
1.01
1.00
3.06
2.99
9.0
8.0
7.0
6.0
5.0
4.0
3.0
2.0
1.0
7.449
7.425
7.404
7.368
7.165
7.146
6.858
6.826
6.146
6.115
2.355
2.164
0.000
X : parts per Million : Proton

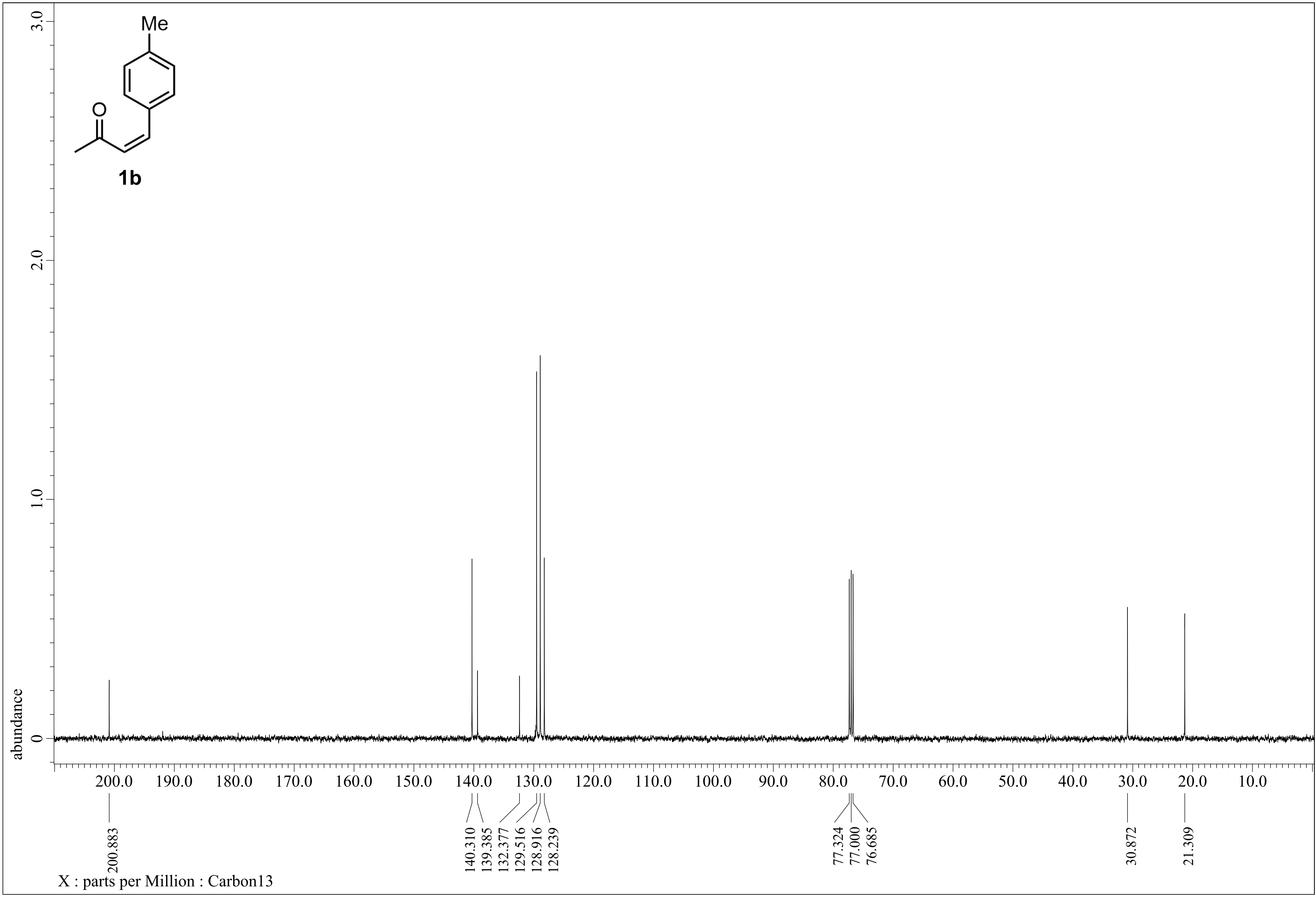
Me
O
1b
3.0
2.0
1.0
0
abundance
200.0
190.0
180.0
170.0
160.0
150.0
140.0
130.0
120.0
110.0
100.0
90.0
80.0
70.0
60.0
50.0
40.0
30.0
20.0
10.0
200.883
140.310
139.385
132.377
129.516
128.916
128.239
77.324
77.000
76.685
30.872
21.309
X : parts per Million : Carbon13

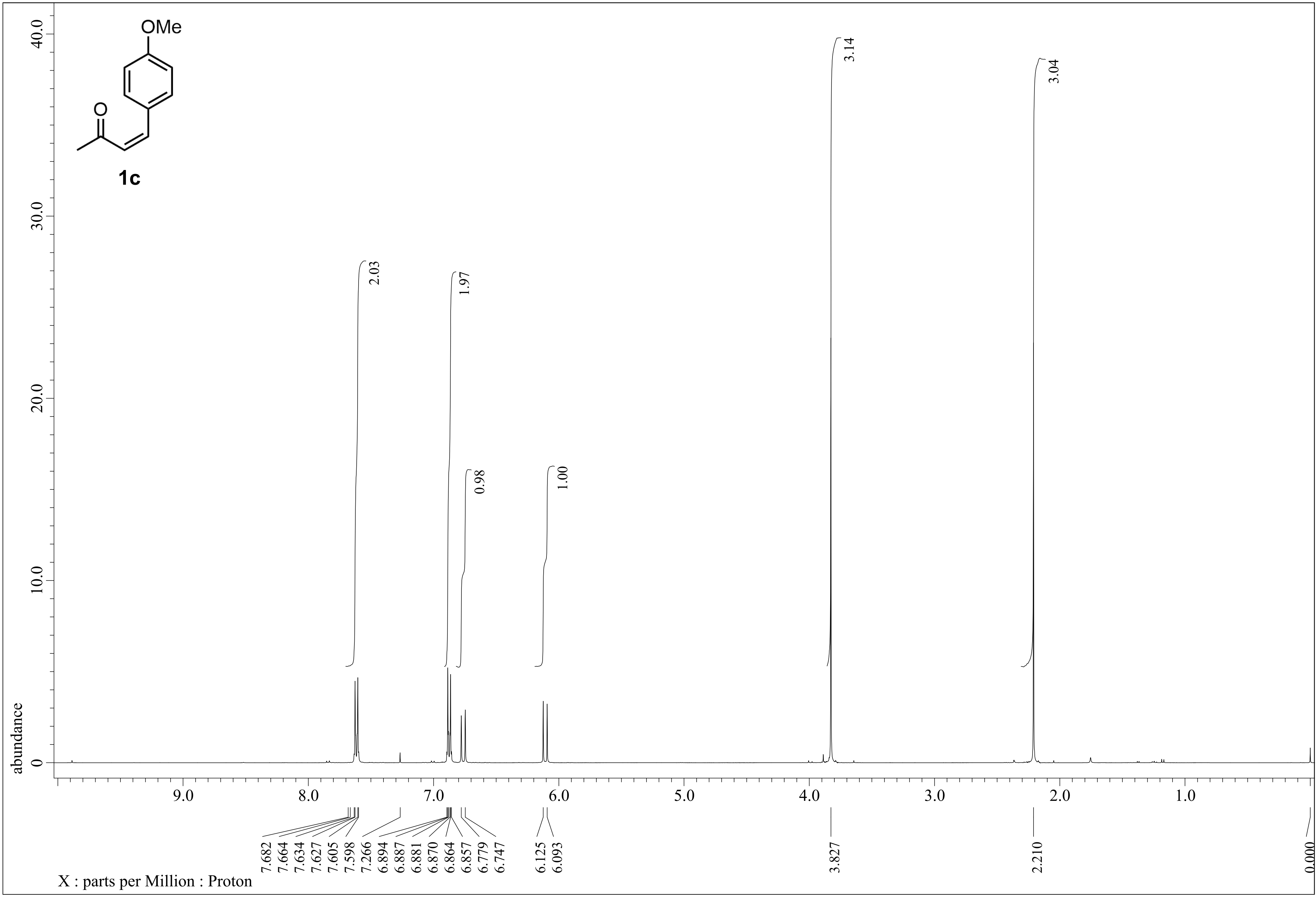
OMe
1c
2.03
1.97
0.98
1.00
3.14
3.04
40.0
30.0
20.0
10.0
0
abundance
9.0
8.0
7.0
6.0
5.0
4.0
3.0
2.0
1.0
7.682
7.664
7.634
7.627
7.605
7.598
7.266
6.894
6.887
6.881
6.870
6.864
6.857
6.779
6.747
6.125
6.093
3.827
2.210
0.000
X : parts per Million : Proton

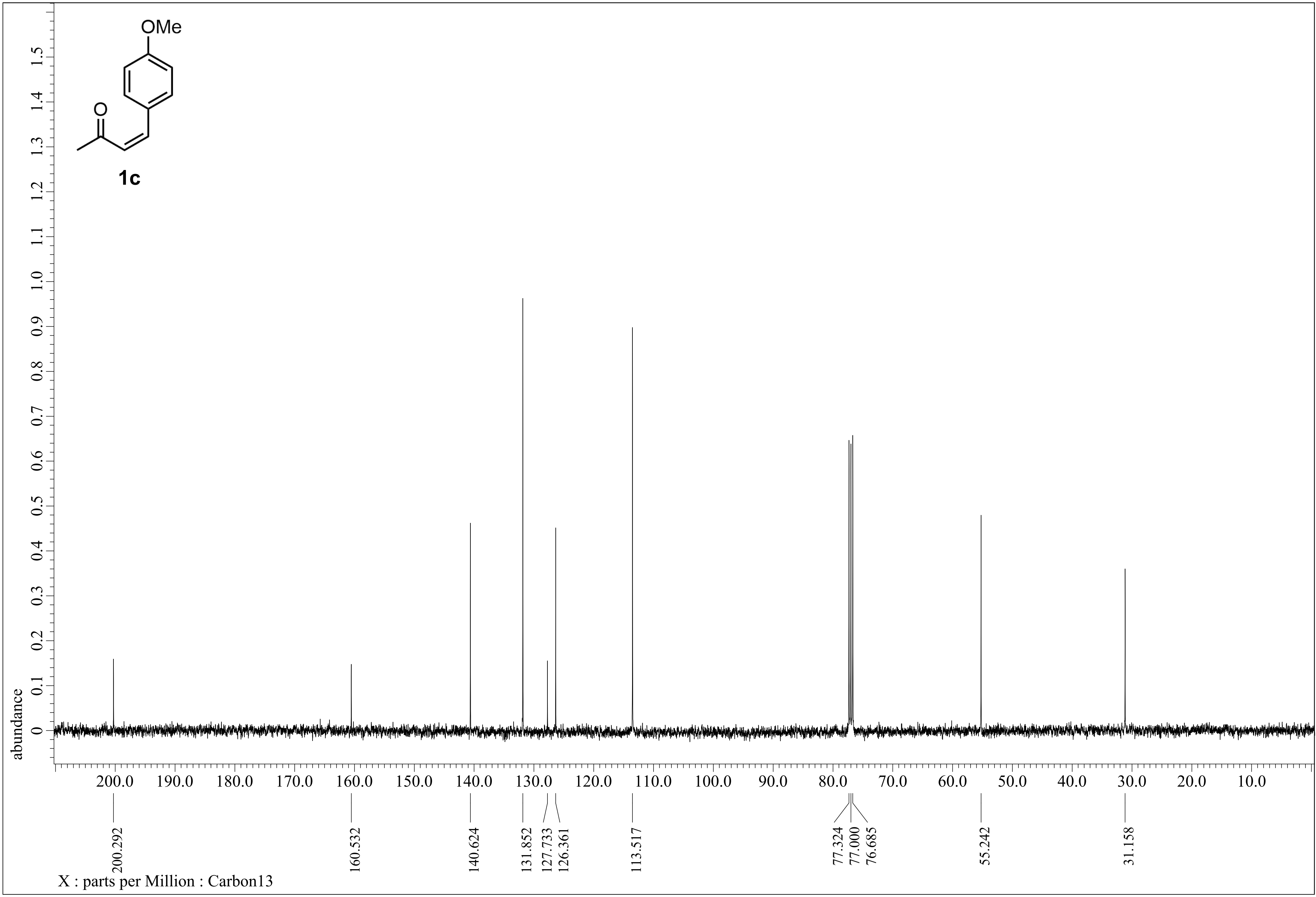
OMe
O
1c
abundance
X : parts per Million : Carbon13
200.292
160.532
140.624
131.852
127.733
126.361
113.517
77.324
77.000
76.685
55.242
31.158

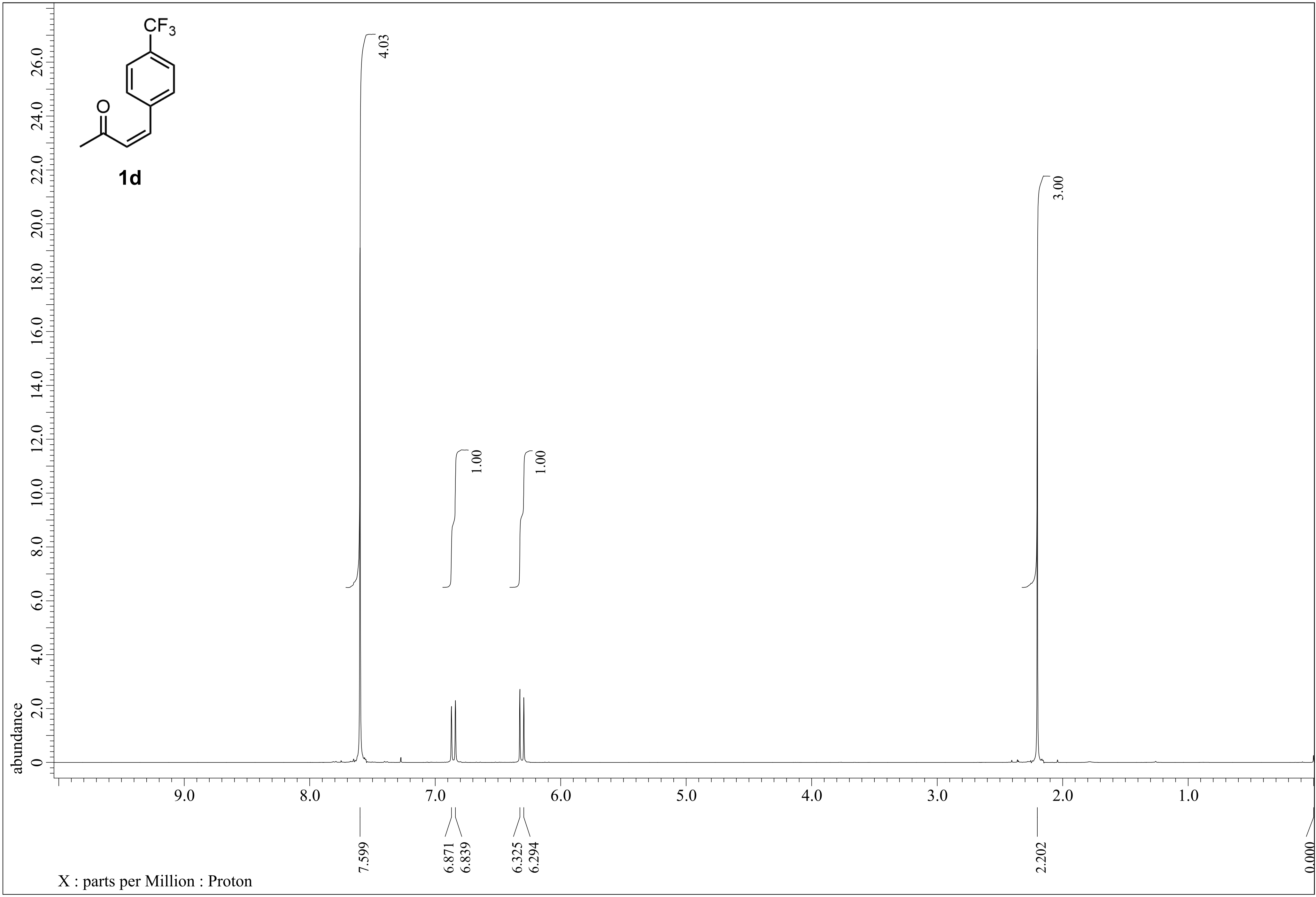
CF3
1d
4.03
1.00
1.00
3.00
abundance
X : parts per Million : Proton
7.599
6.871
6.839
6.325
6.294
2.202
0.000

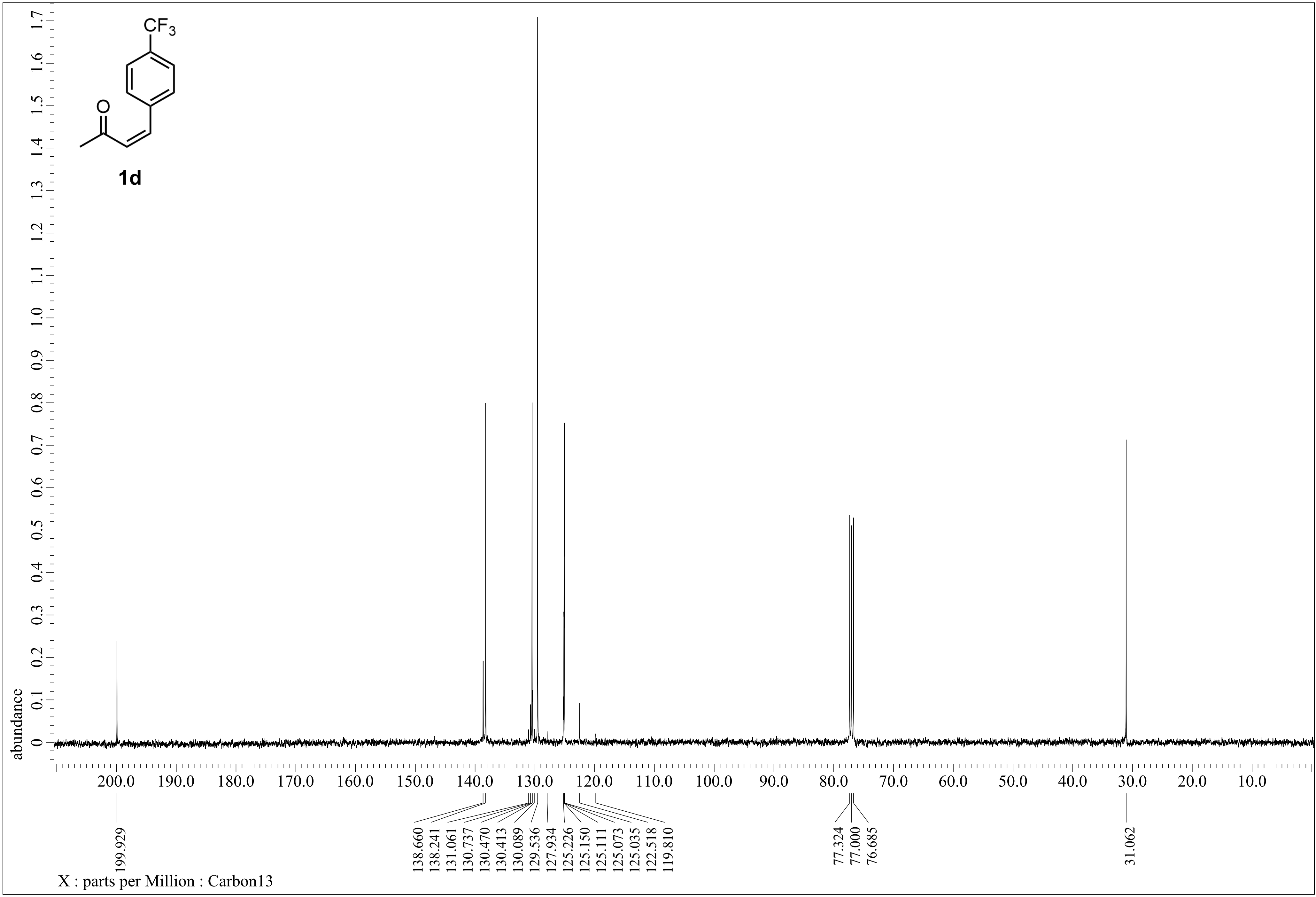
CF3
O
1d
abundance
0 0.1 0.2 0.3 0.4 0.5 0.6 0.7 0.8 0.9 1.0 1.1 1.2 1.3 1.4 1.5 1.6 1.7
200.0 190.0 180.0 170.0 160.0 150.0 140.0 130.0 120.0 110.0 100.0 90.0 80.0 70.0 60.0 50.0 40.0 30.0 20.0 10.0
199.929
138.660 138.241 131.061 130.737 130.470 130.413 130.089 129.536 127.934 125.226 125.150 125.111 125.073 125.035 122.518 119.810
77.324 77.000 76.685
31.062
X : parts per Million : Carbon13

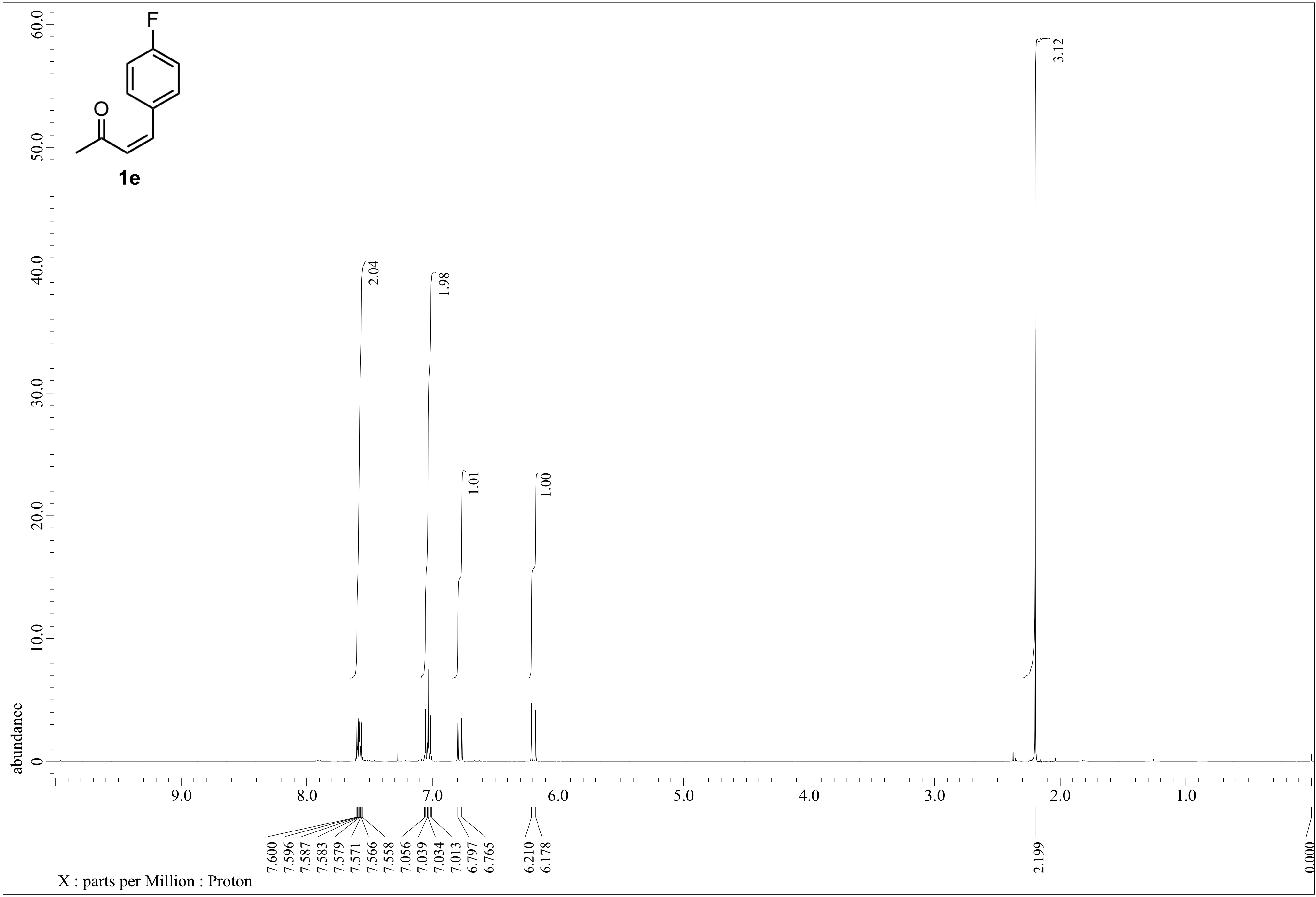

1e
F
O
abundance
X : parts per Million : Proton
2.04
1.98
1.01
1.00
3.12
7.600
7.596
7.587
7.583
7.579
7.571
7.566
7.558
7.056
7.039
7.034
7.013
6.797
6.765
6.210
6.178
2.199
0.000

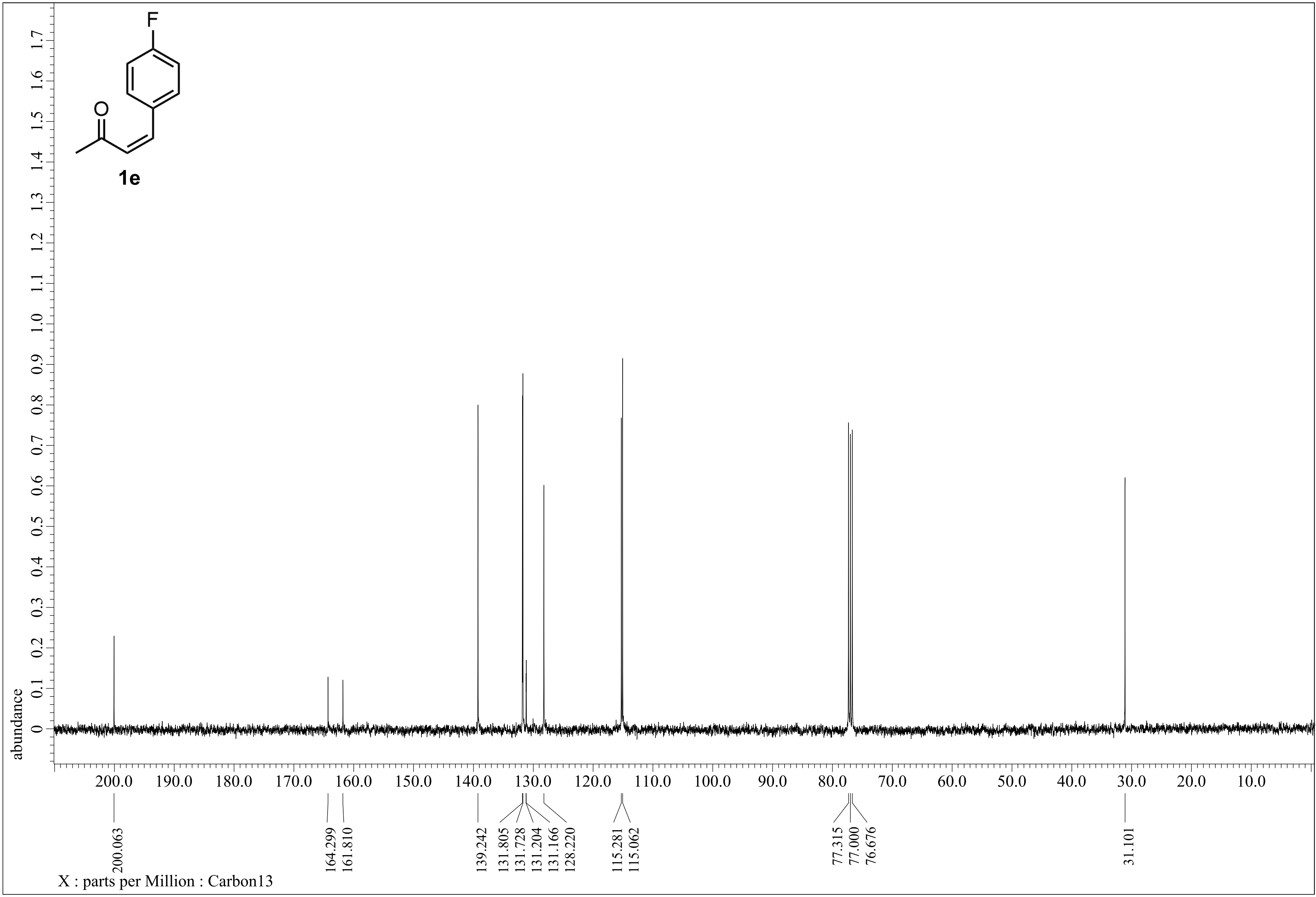

F
O
1e
abundance
X : parts per Million : Carbon13
200.063
164.299
161.810
139.242
131.805
131.728
131.204
131.166
128.220
115.281
115.062
77.315
77.000
76.676
31.101

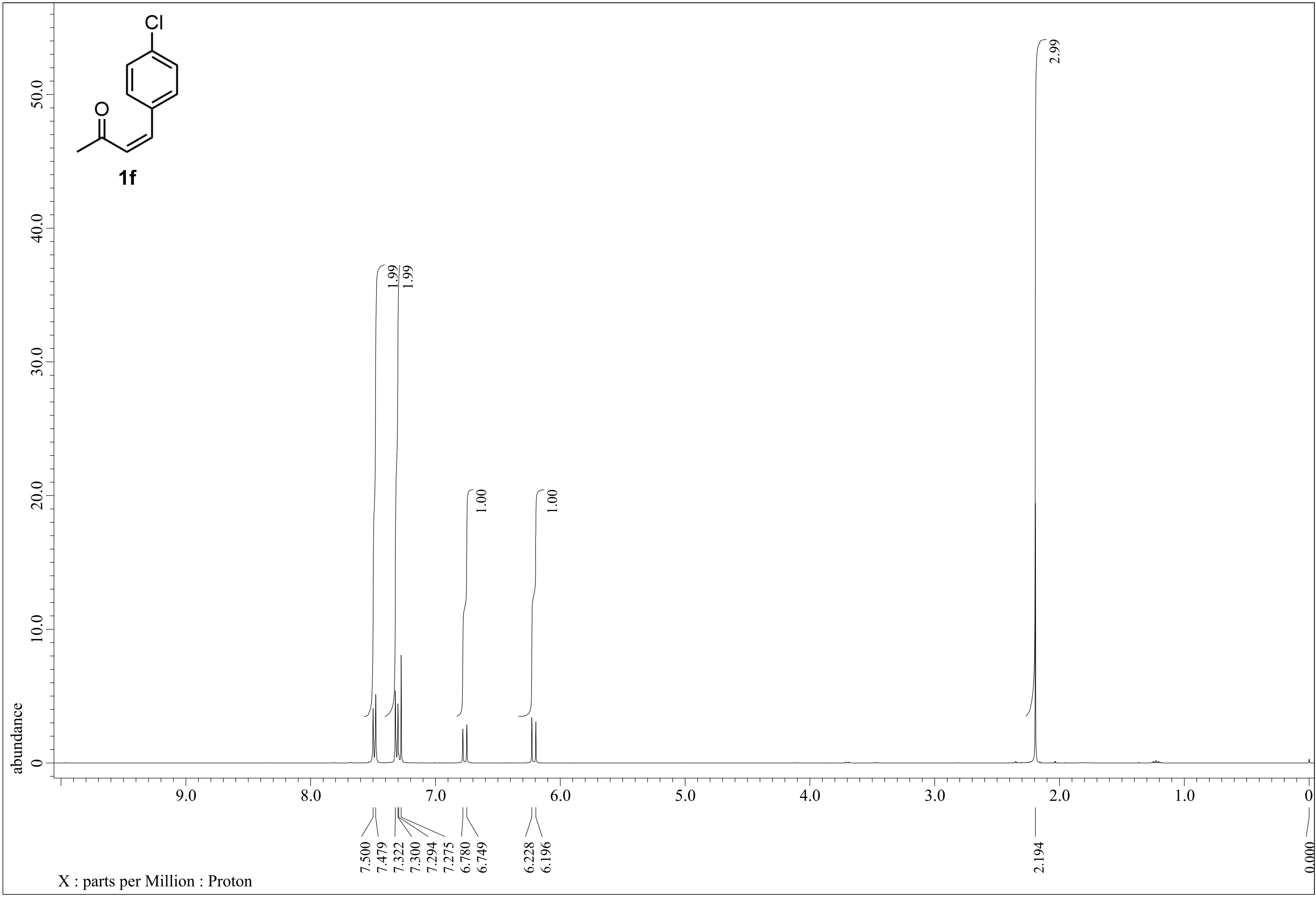

Cl
O
1f
abundance
50.0
40.0
30.0
20.0
10.0
0
9.0
8.0
7.0
6.0
5.0
4.0
3.0
2.0
1.0
0
1.99
1.99
1.00
1.00
2.99
7.500
7.479
7.322
7.300
7.294
7.275
6.780
6.749
6.228
6.196
2.194
0.000
X : parts per Million : Proton

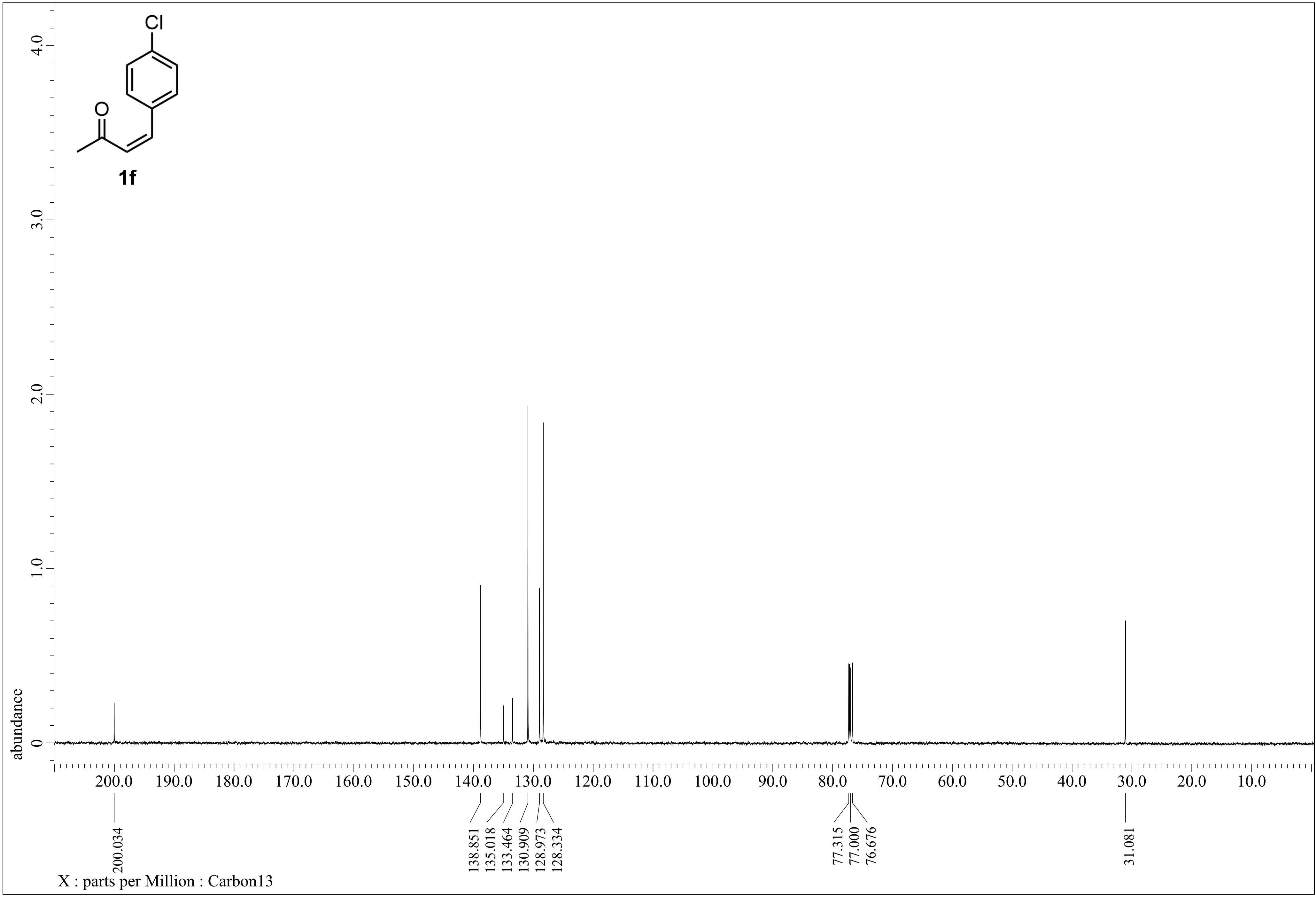

Cl
O
1f
4.0
3.0
2.0
1.0
0
abundance
200.0
190.0
180.0
170.0
160.0
150.0
140.0
130.0
120.0
110.0
100.0
90.0
80.0
70.0
60.0
50.0
40.0
30.0
20.0
10.0
200.034
138.851
135.018
133.464
130.909
128.973
128.334
77.315
77.000
76.676
31.081
X : parts per Million : Carbon13

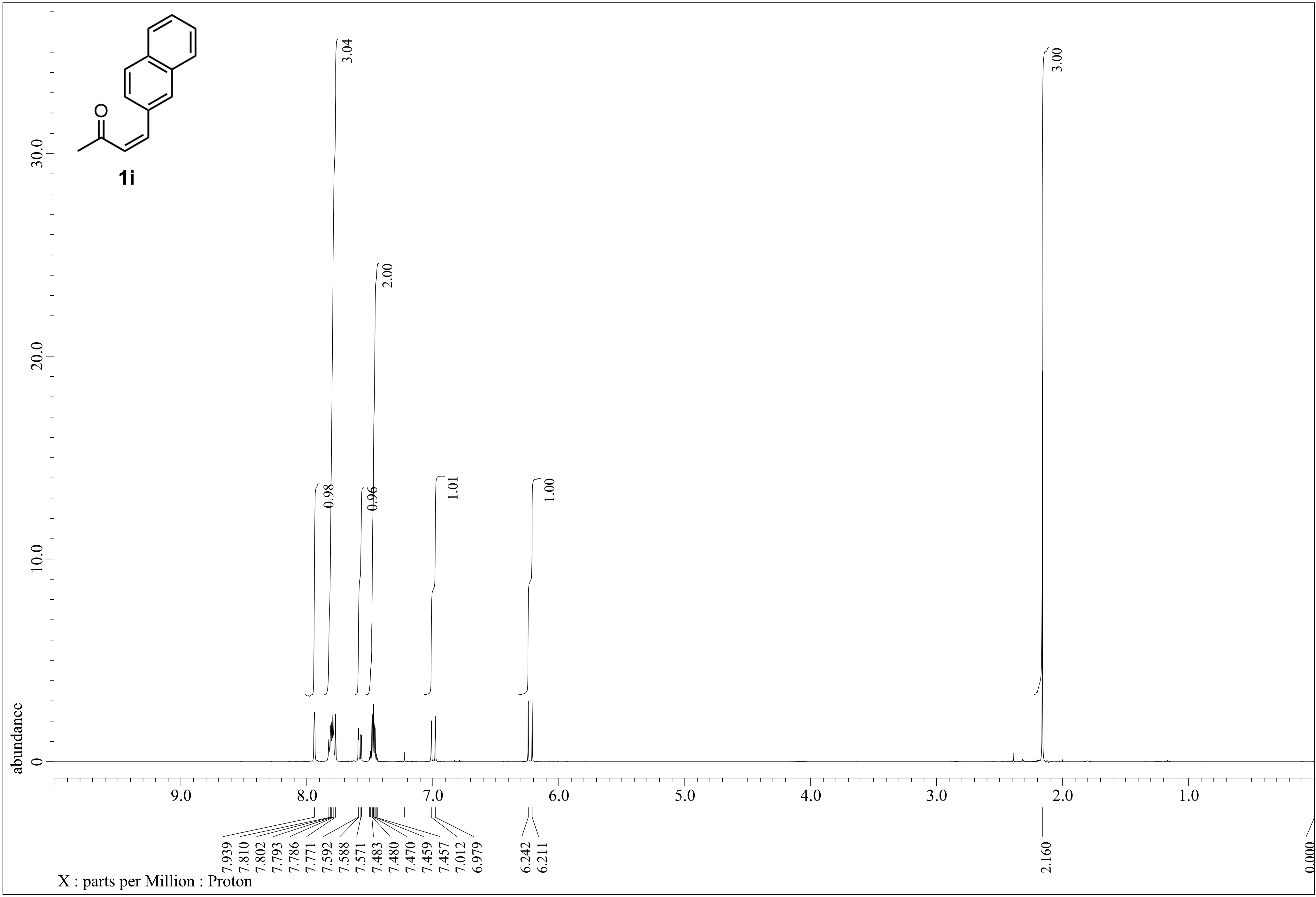
1i
abundance
30.0
20.0
10.0
0
3.04
2.00
0.98
0.96
1.01
1.00
3.00
9.0
8.0
7.0
6.0
5.0
4.0
3.0
2.0
1.0
7.939
7.810
7.802
7.793
7.786
7.771
7.592
7.588
7.571
7.483
7.480
7.470
7.459
7.457
7.012
6.979
6.242
6.211
2.160
0.000
X : parts per Million : Proton

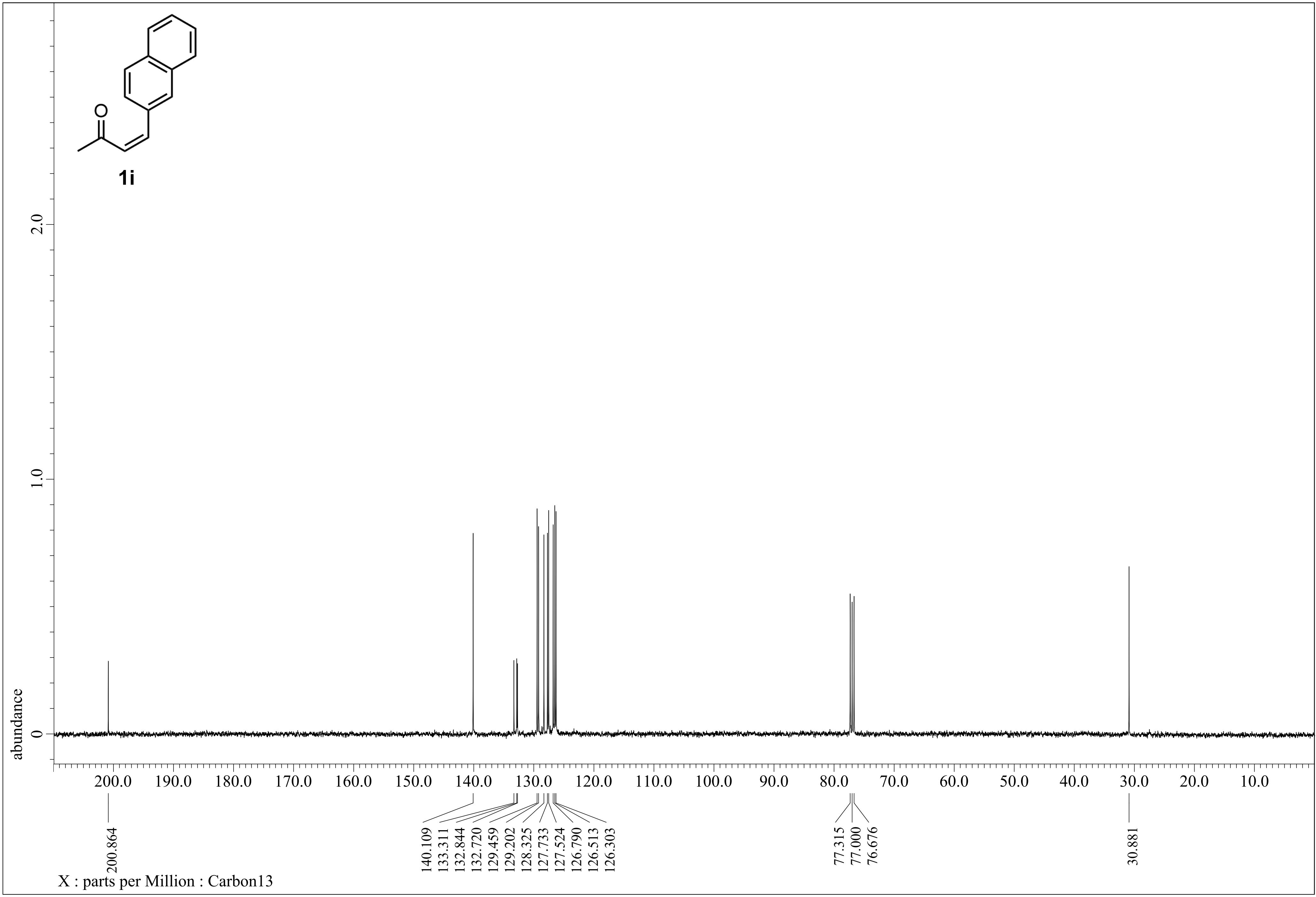

1i
2.0
1.0
0
abundance
200.0
190.0
180.0
170.0
160.0
150.0
140.0
130.0
120.0
110.0
100.0
90.0
80.0
70.0
60.0
50.0
40.0
30.0
20.0
10.0
200.864
140.109
133.311
132.844
132.720
129.459
129.202
128.325
127.733
127.524
126.790
126.513
126.303
77.315
77.000
76.676
30.881
X : parts per Million : Carbon13

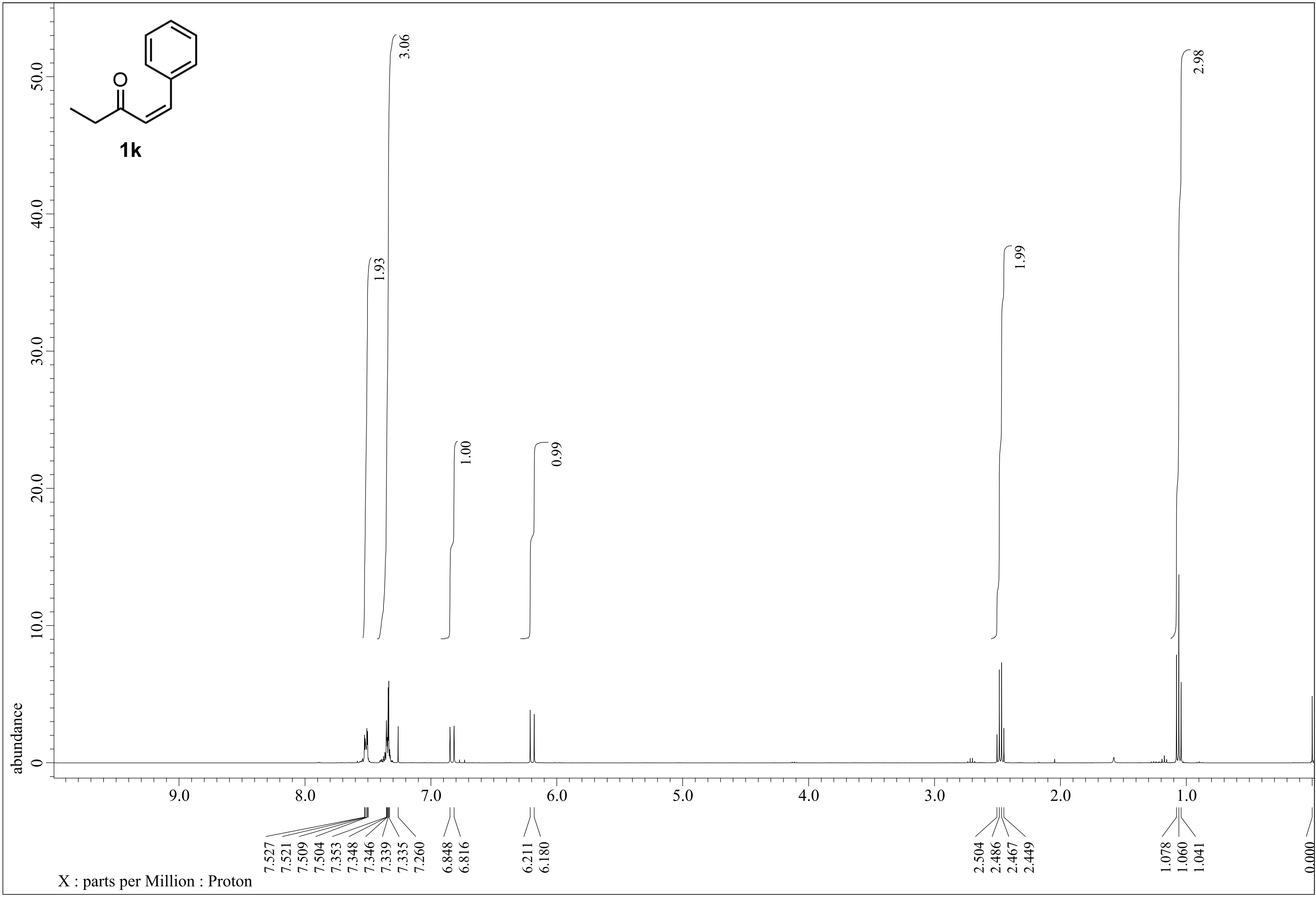
1k
abundance
X : parts per Million : Proton
3.06
1.93
1.00
0.99
1.99
2.98
7.527
7.521
7.509
7.504
7.353
7.348
7.346
7.339
7.335
7.260
6.848
6.816
6.211
6.180
2.504
2.486
2.467
2.449
1.078
1.060
1.041
0.000

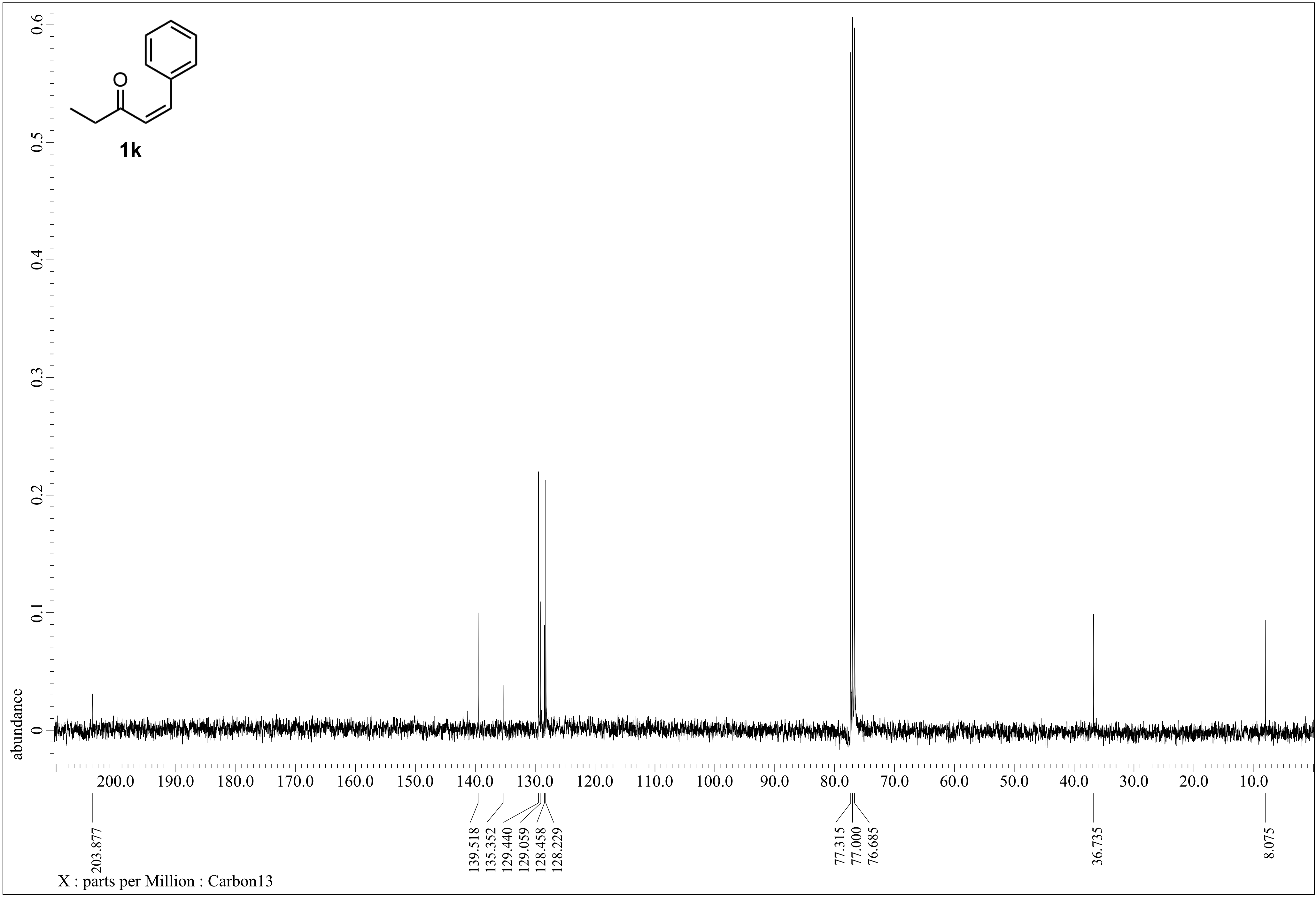
1k
abundance
X : parts per Million : Carbon13
203.877
139.518
135.352
129.440
129.059
128.458
128.229
77.315
77.000
76.685
36.735
8.075

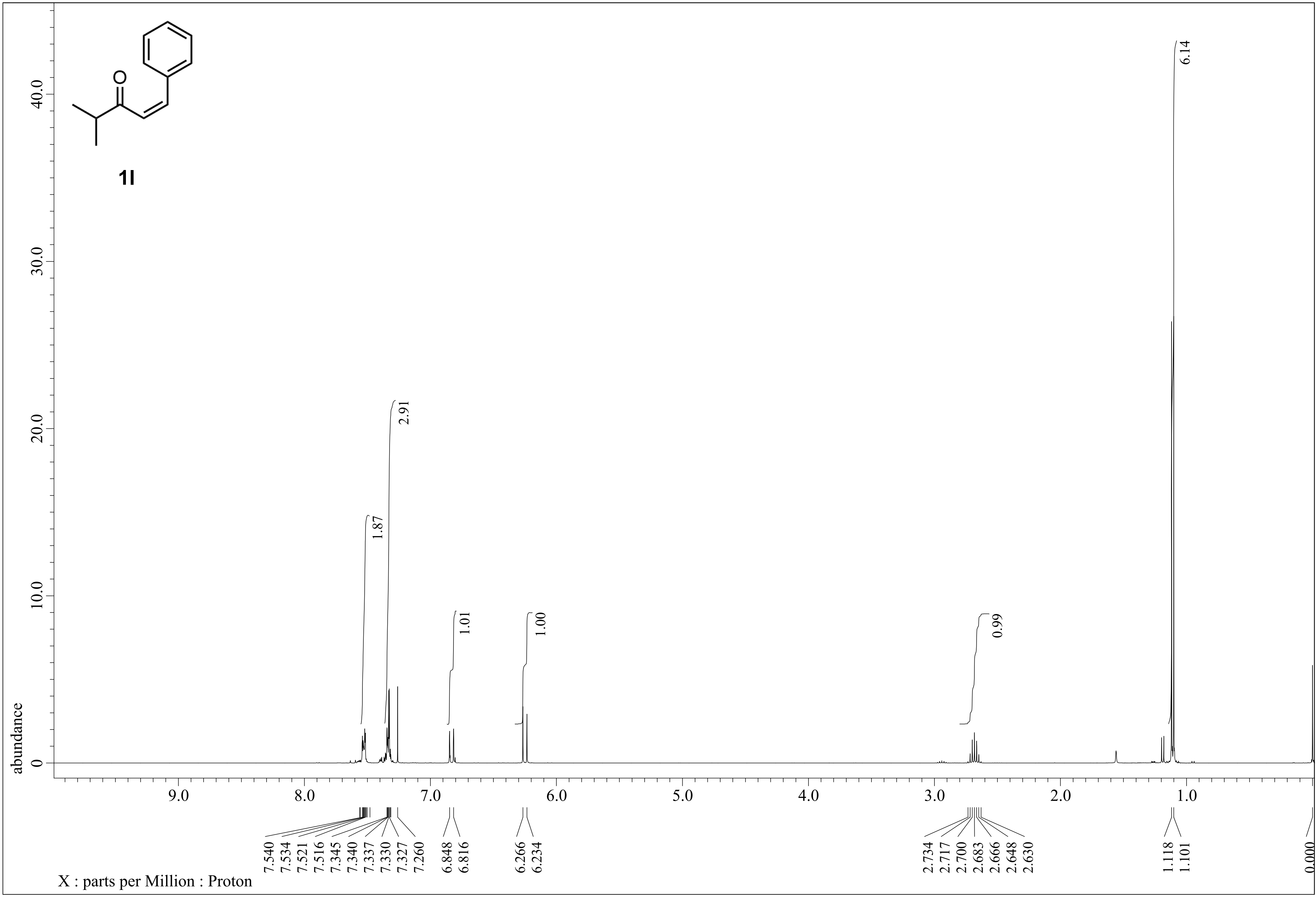
1l
abundance
0
10.0
20.0
30.0
40.0
9.0
8.0
7.0
6.0
5.0
4.0
3.0
2.0
1.0
1.87
2.91
1.01
1.00
0.99
6.14
7.540
7.534
7.521
7.516
7.345
7.340
7.337
7.330
7.327
7.260
6.848
6.816
6.266
6.234
2.734
2.717
2.700
2.683
2.666
2.648
2.630
1.118
1.101
0.000
X : parts per Million : Proton

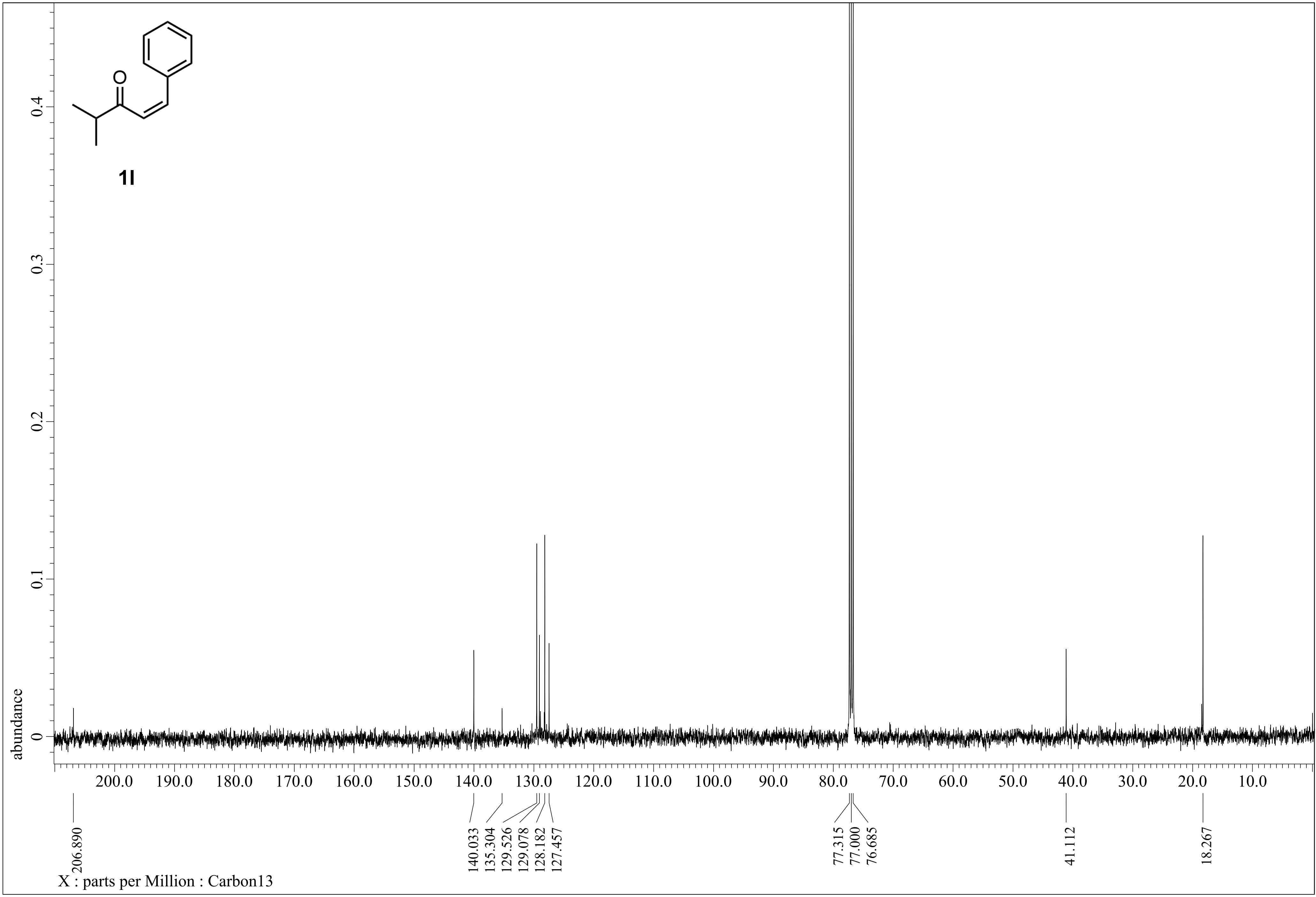
1l
abundance
0.4
0.3
0.2
0.1
0
200.0 190.0 180.0 170.0 160.0 150.0 140.0 130.0 120.0 110.0 100.0 90.0 80.0 70.0 60.0 50.0 40.0 30.0 20.0 10.0
206.890
140.033
135.304
129.526
129.078
128.182
127.457
77.315
77.000
76.685
41.112
18.267
X : parts per Million : Carbon13

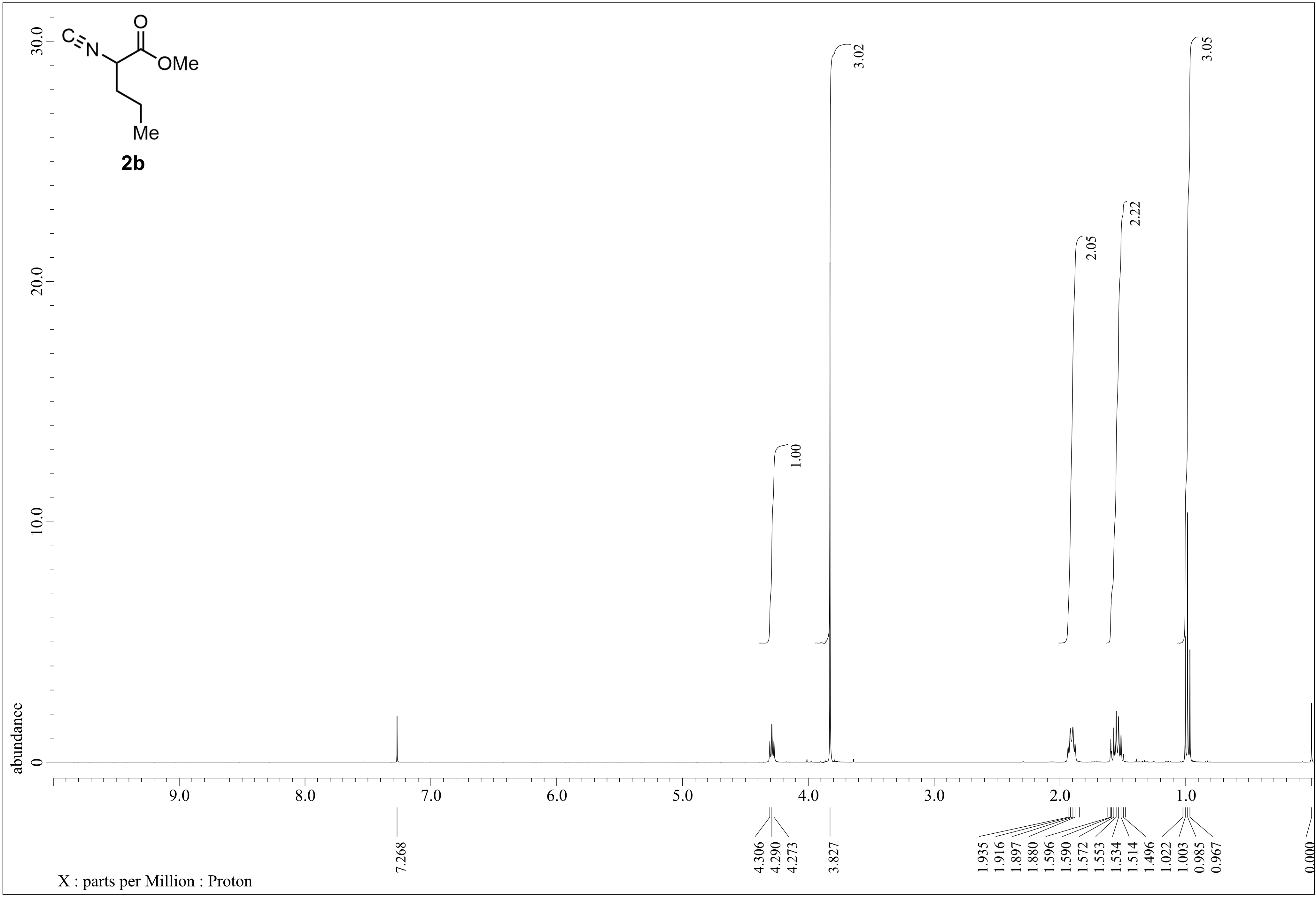
2b
abundance
X : parts per Million : Proton
7.268
4.306
4.290
4.273
3.827
1.935
1.916
1.897
1.880
1.596
1.590
1.572
1.553
1.534
1.514
1.496
1.022
1.003
0.985
0.967
0.000
1.00
3.02
2.05
2.22
3.05

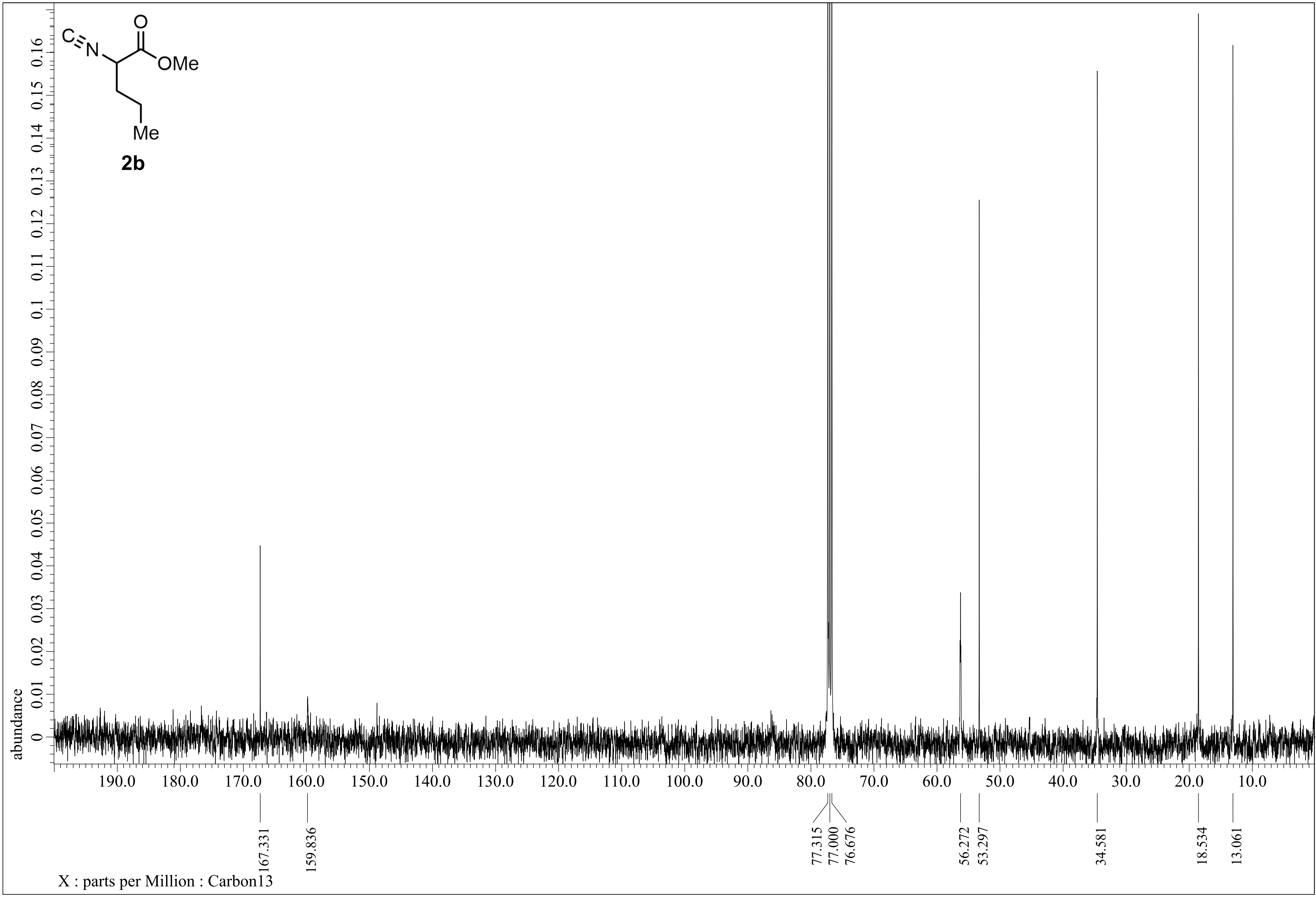

O
C≡N
OMe
Me
2b
abundance
0
0.01
0.02
0.03
0.04
0.05
0.06
0.07
0.08
0.09
0.1
0.11
0.12
0.13
0.14
0.15
0.16
190.0
180.0
170.0
160.0
150.0
140.0
130.0
120.0
110.0
100.0
90.0
80.0
70.0
60.0
50.0
40.0
30.0
20.0
10.0
167.331
159.836
77.315
77.000
76.676
56.272
53.297
34.581
18.534
13.061
X : parts per Million : Carbon13

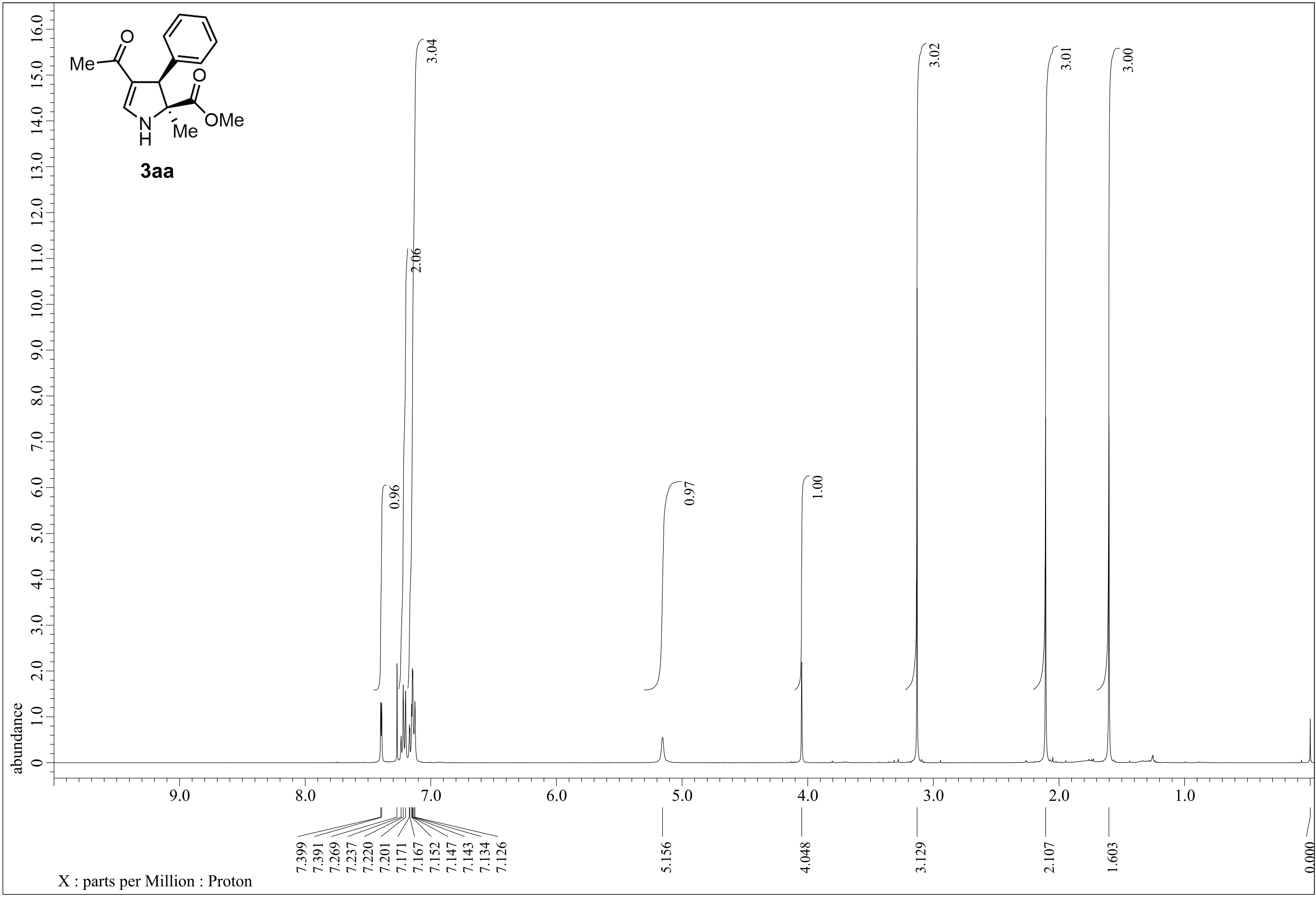

3aa

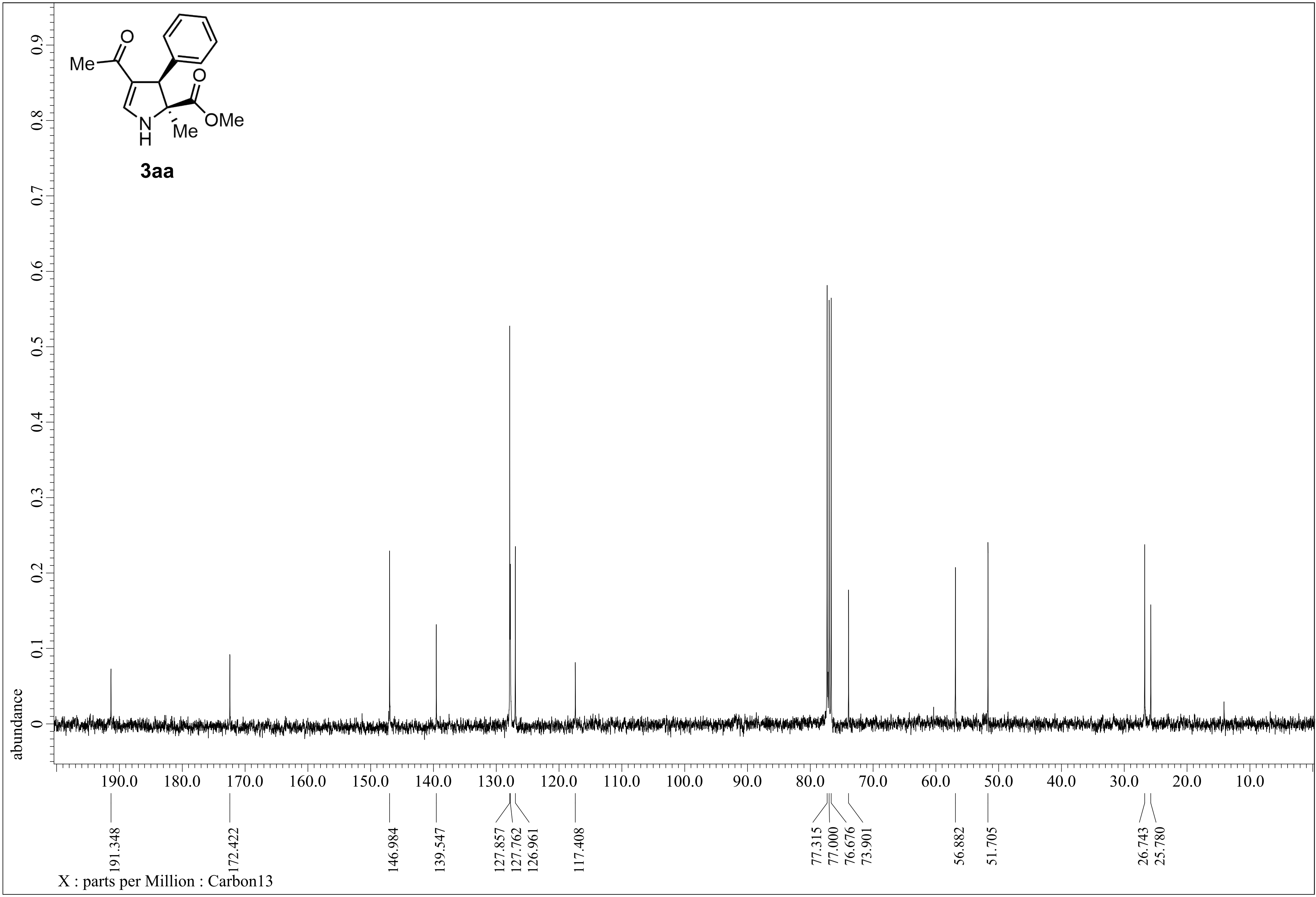
Me
O
O
OMe
N
H
Me
3aa
abundance
0.9
0.8
0.7
0.6
0.5
0.4
0.3
0.2
0.1
0
190.0
180.0
170.0
160.0
150.0
140.0
130.0
120.0
110.0
100.0
90.0
80.0
70.0
60.0
50.0
40.0
30.0
20.0
10.0
191.348
172.422
146.984
139.547
127.857
127.762
126.961
117.408
77.315
77.000
76.676
73.901
56.882
51.705
26.743
25.780
X : parts per Million : Carbon13

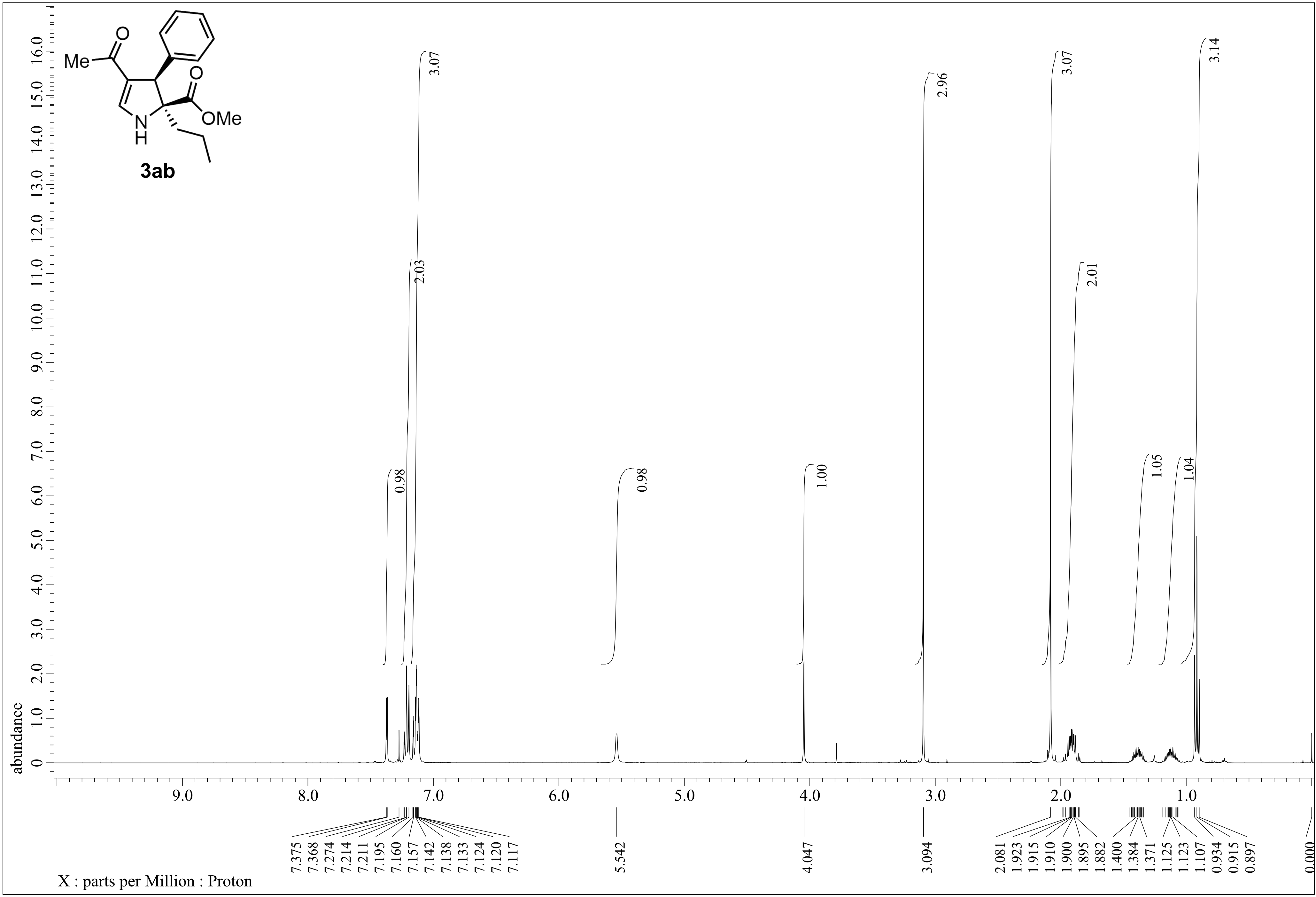
3ab
Me
OMe
N
H
O
abundance
X : parts per Million : Proton
0.98
2.03
3.07
0.98
1.00
2.96
3.07
2.01
1.05
1.04
3.14
7.375
7.368
7.274
7.214
7.211
7.195
7.160
7.157
7.142
7.138
7.133
7.124
7.120
7.117
5.542
4.047
3.094
2.081
1.923
1.915
1.910
1.900
1.895
1.882
1.400
1.384
1.371
1.125
1.123
1.107
0.934
0.915
0.897
0.000

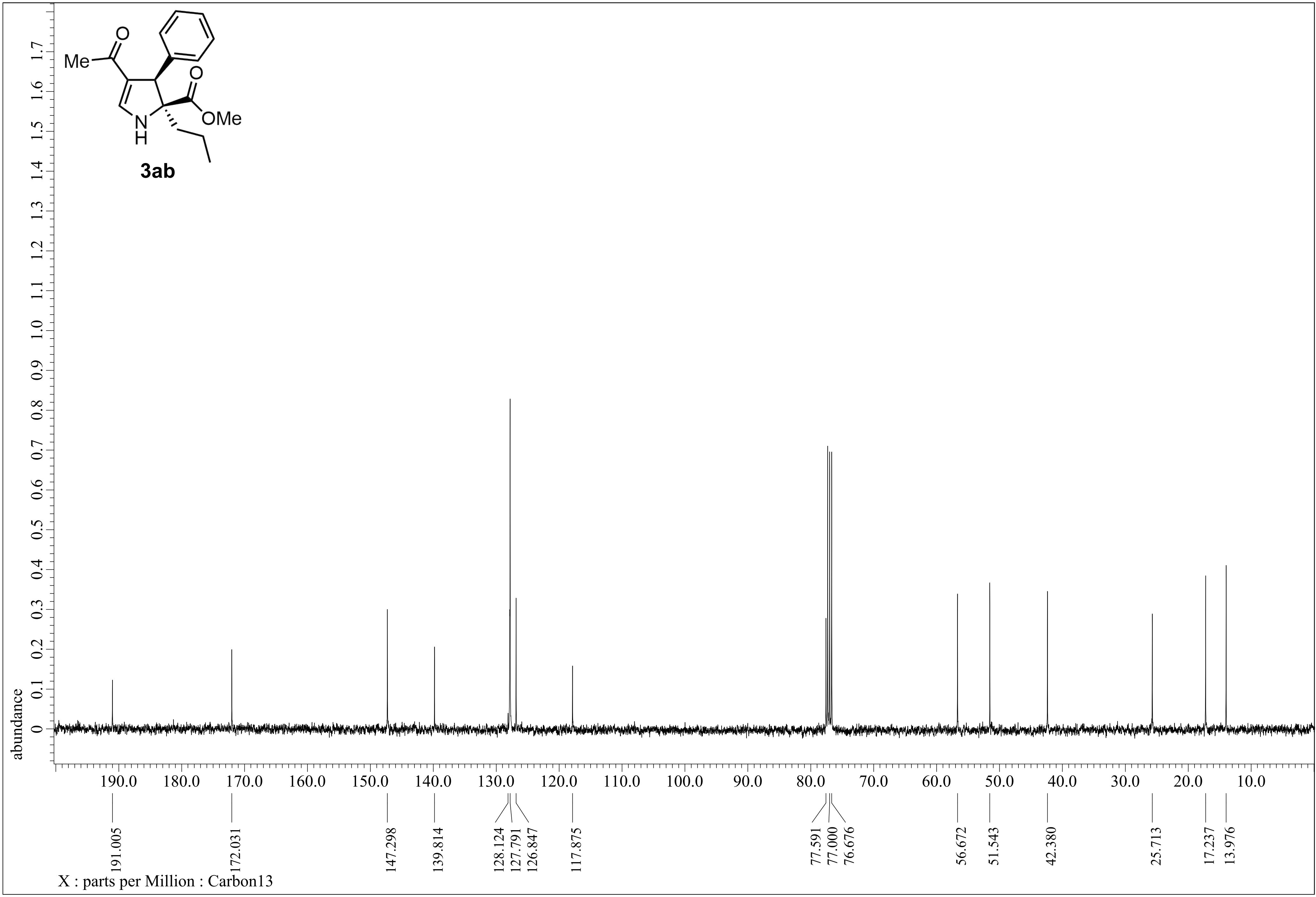
Me
O
O
OMe
N
H
3ab
abundance
1.7
1.6
1.5
1.4
1.3
1.2
1.1
1.0
0.9
0.8
0.7
0.6
0.5
0.4
0.3
0.2
0.1
0
190.0 180.0 170.0 160.0 150.0 140.0 130.0 120.0 110.0 100.0 90.0 80.0 70.0 60.0 50.0 40.0 30.0 20.0 10.0
191.005
172.031
147.298
139.814
128.124
127.791
126.847
117.875
77.591
77.000
76.676
56.672
51.543
42.380
25.713
17.237
13.976
X : parts per Million : Carbon13

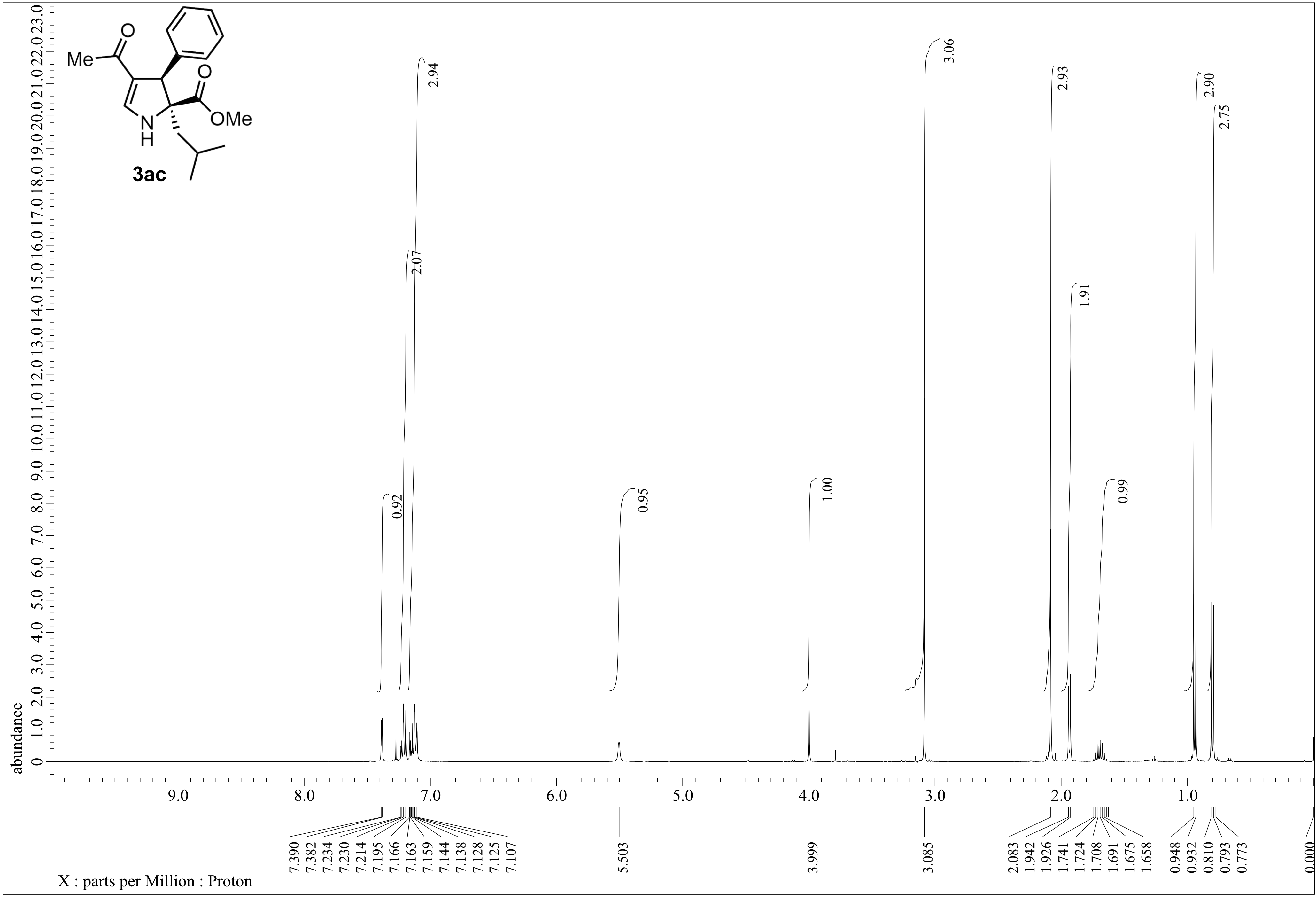
Me
O
O
OMe
N
H
3ac
abundance
X : parts per Million : Proton
0.92
2.07
2.94
0.95
1.00
3.06
2.93
1.91
0.99
2.90
2.75
7.390
7.382
7.234
7.230
7.214
7.195
7.166
7.163
7.159
7.144
7.138
7.128
7.125
7.107
5.503
3.999
3.085
2.083
1.942
1.926
1.741
1.724
1.708
1.691
1.675
1.658
0.948
0.932
0.810
0.793
0.773
0.000

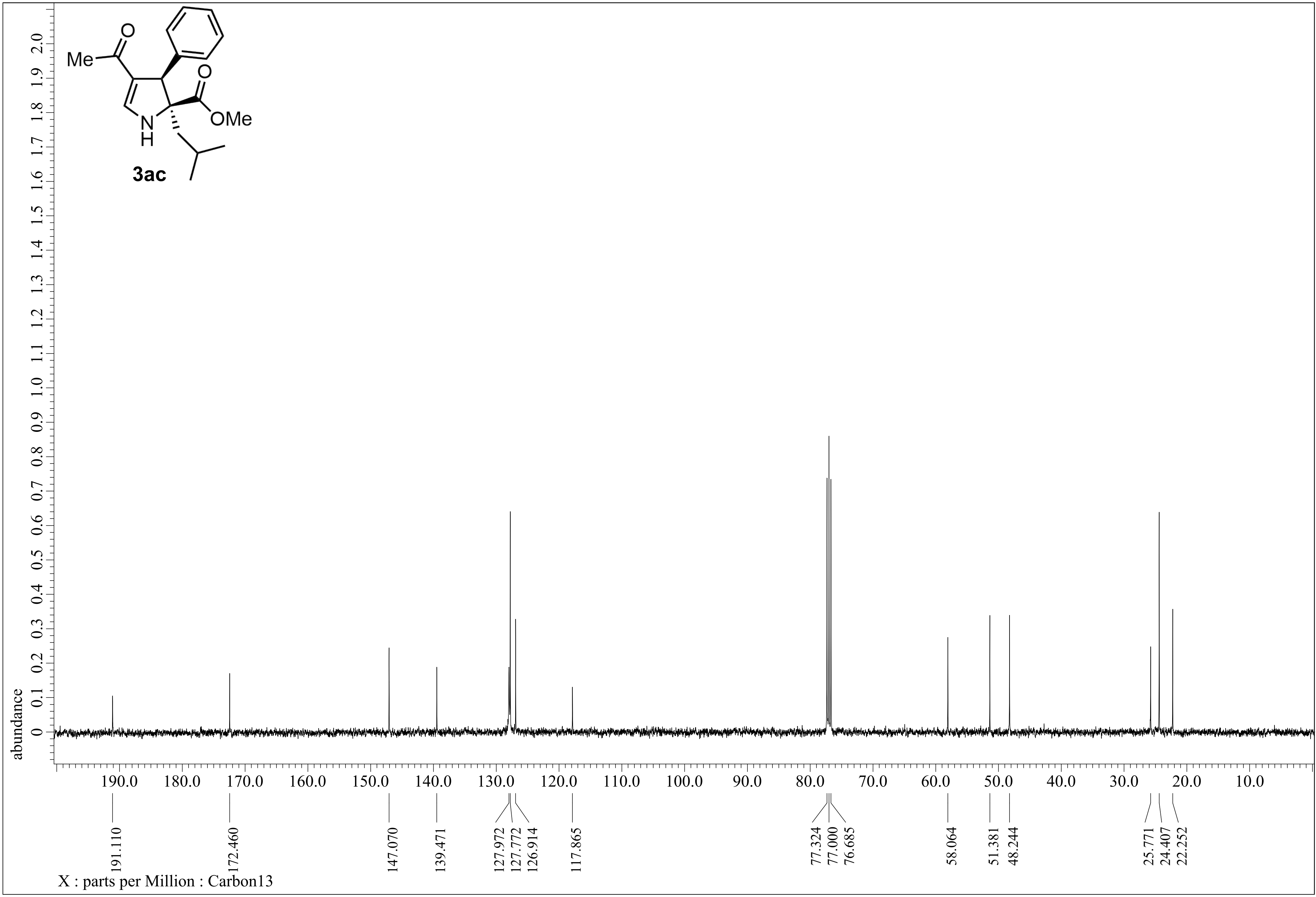
Me
O
O
OMe
N
H
3ac
abundance
X : parts per Million : Carbon13
191.110
172.460
147.070
139.471
127.972
127.772
126.914
117.865
77.324
77.000
76.685
58.064
51.381
48.244
25.771
24.407
22.252

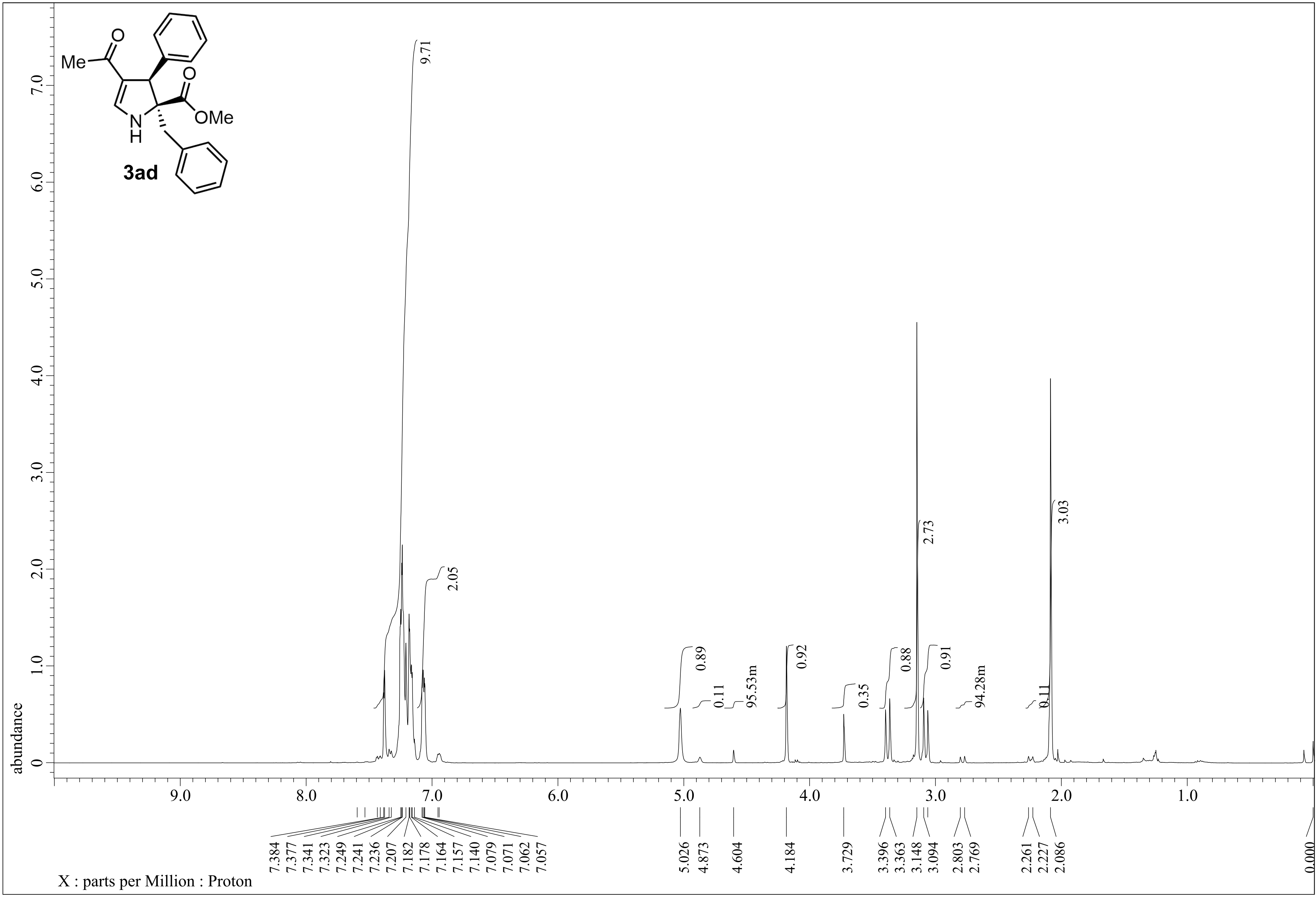
Me
O
O
OMe
N
H
3ad
abundance
X : parts per Million : Proton
9.71
2.05
0.89
0.11
95.53m
0.92
0.35
0.88
2.73
0.91
94.28m
0.11
3.03
7.384 7.377 7.341 7.323 7.249 7.241 7.236 7.207 7.182 7.178 7.164 7.157 7.140 7.079 7.071 7.062 7.057
5.026 4.873 4.604 4.184 3.729 3.396 3.363 3.148 3.094 2.803 2.769 2.261 2.227 2.086 0.000

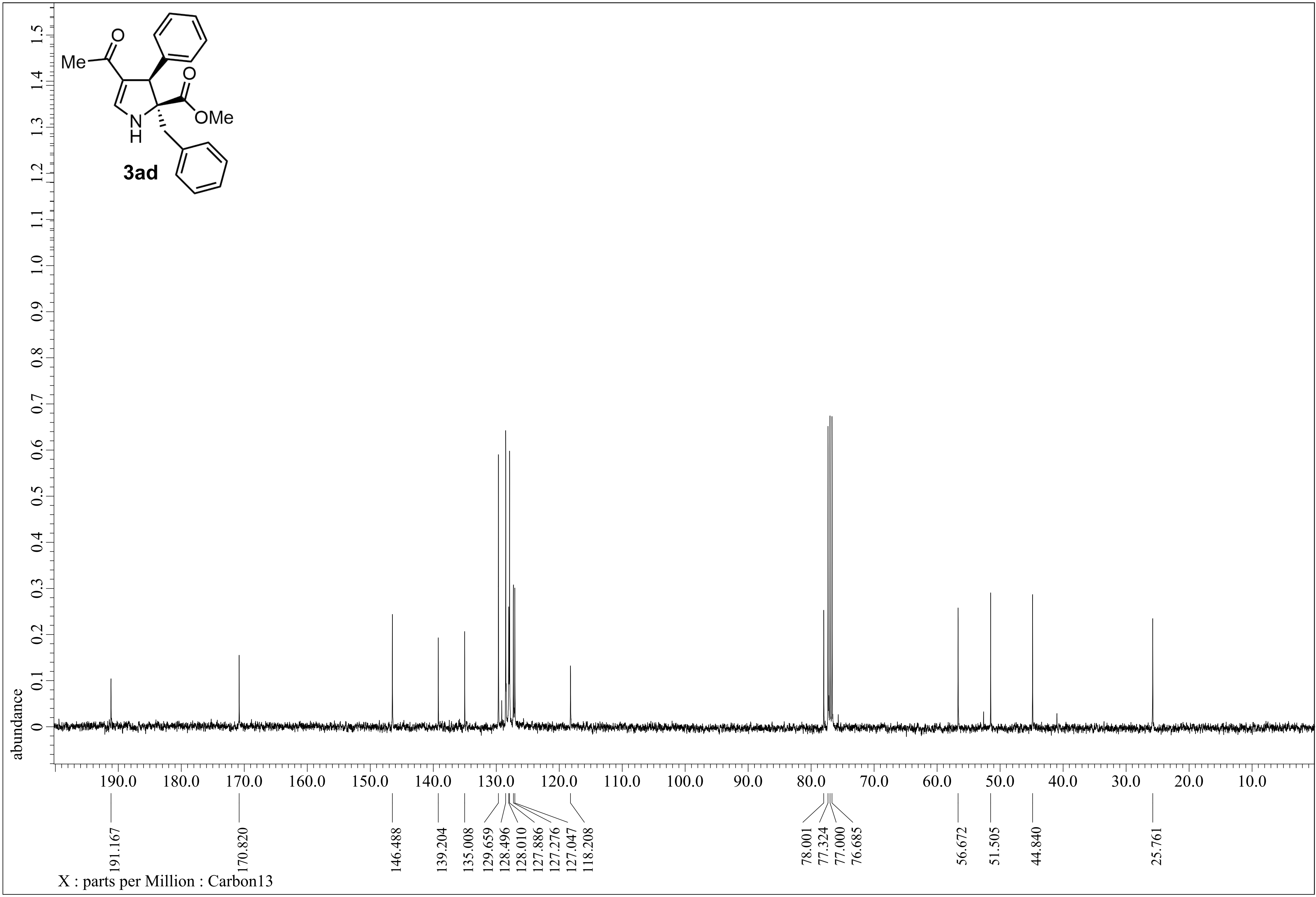

Me
O
O
OMe
N
H
3ad
abundance
X : parts per Million : Carbon13
191.167
170.820
146.488
139.204
135.008
129.659
128.496
128.010
127.886
127.276
127.047
118.208
78.001
77.324
77.000
76.685
56.672
51.505
44.840
25.761

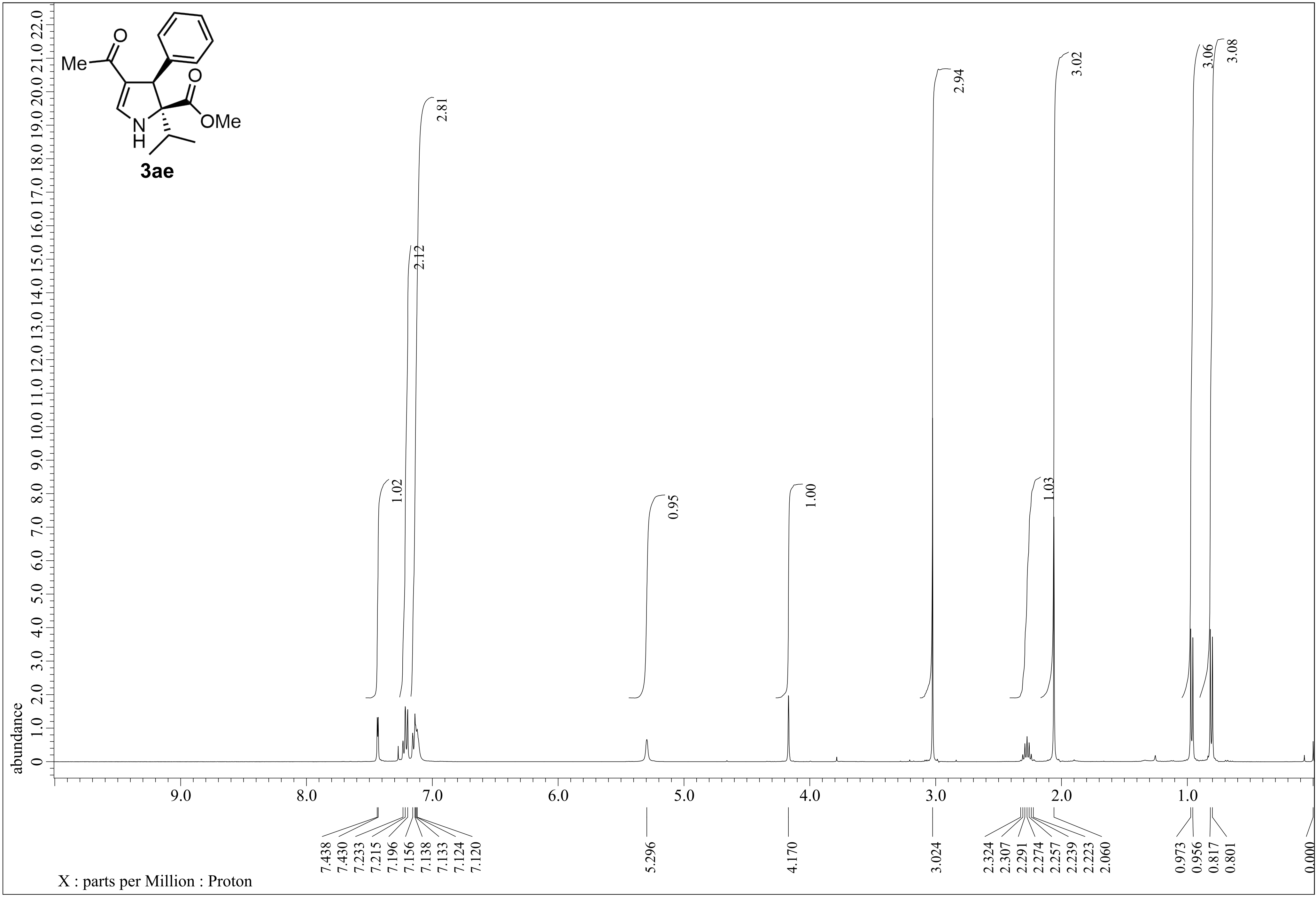
Me
O
O
N
H
OMe
3ae
abundance
X : parts per Million : Proton
1.02
2.12
2.81
0.95
1.00
2.94
1.03
3.02
3.06
3.08
7.438
7.430
7.233
7.215
7.196
7.156
7.138
7.133
7.124
7.120
5.296
4.170
3.024
2.324
2.307
2.291
2.274
2.257
2.239
2.223
2.060
0.973
0.956
0.817
0.801
0.000

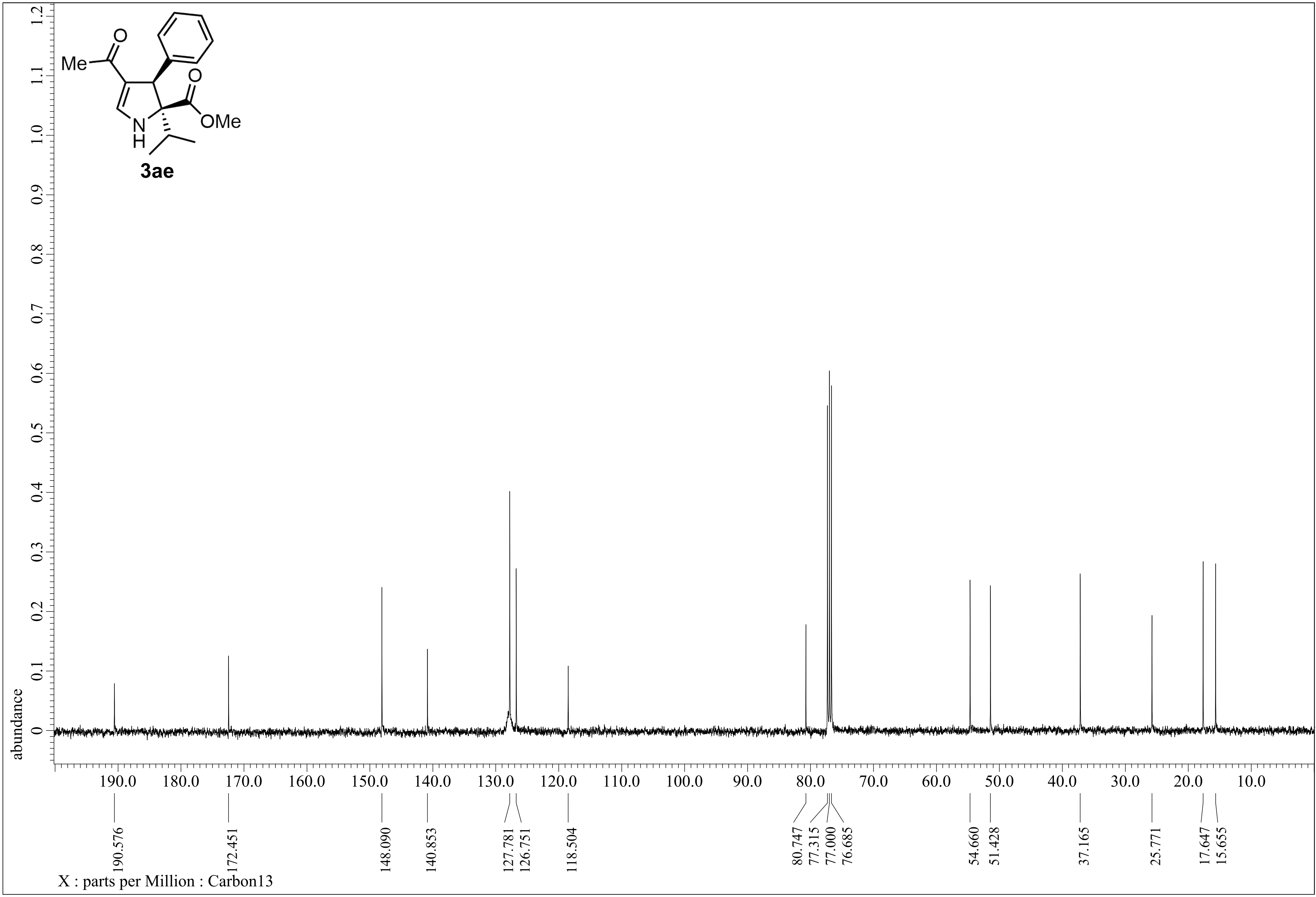

Me
O
O
OMe
N
H
3ae
abundance
X : parts per Million : Carbon13
190.576
172.451
148.090
140.853
127.781
126.751
118.504
80.747
77.315
77.000
76.685
54.660
51.428
37.165
25.771
17.647
15.655

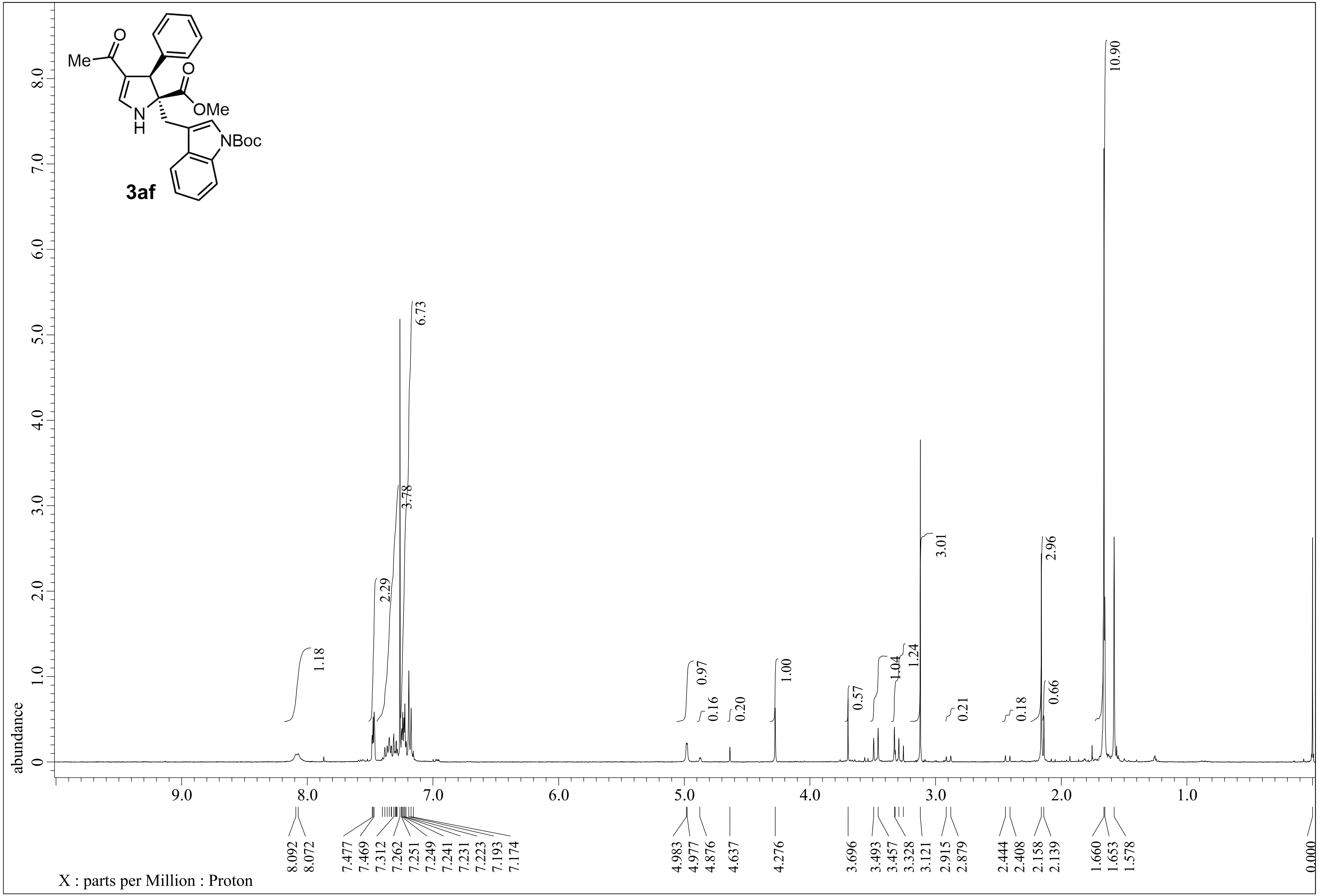
Me
O
O
OMe
N
H
NBoc
3af
abundance
X : parts per Million : Proton
1.18
2.29
3.78
6.73
0.97
0.16
0.20
1.00
0.57
1.04
1.24
3.01
0.21
0.18
2.96
0.66
10.90
8.092
8.072
7.477
7.469
7.312
7.262
7.251
7.249
7.241
7.231
7.223
7.193
7.174
4.983
4.977
4.876
4.637
4.276
3.696
3.493
3.457
3.328
3.121
2.915
2.879
2.444
2.408
2.158
2.139
1.660
1.653
1.578
0.000

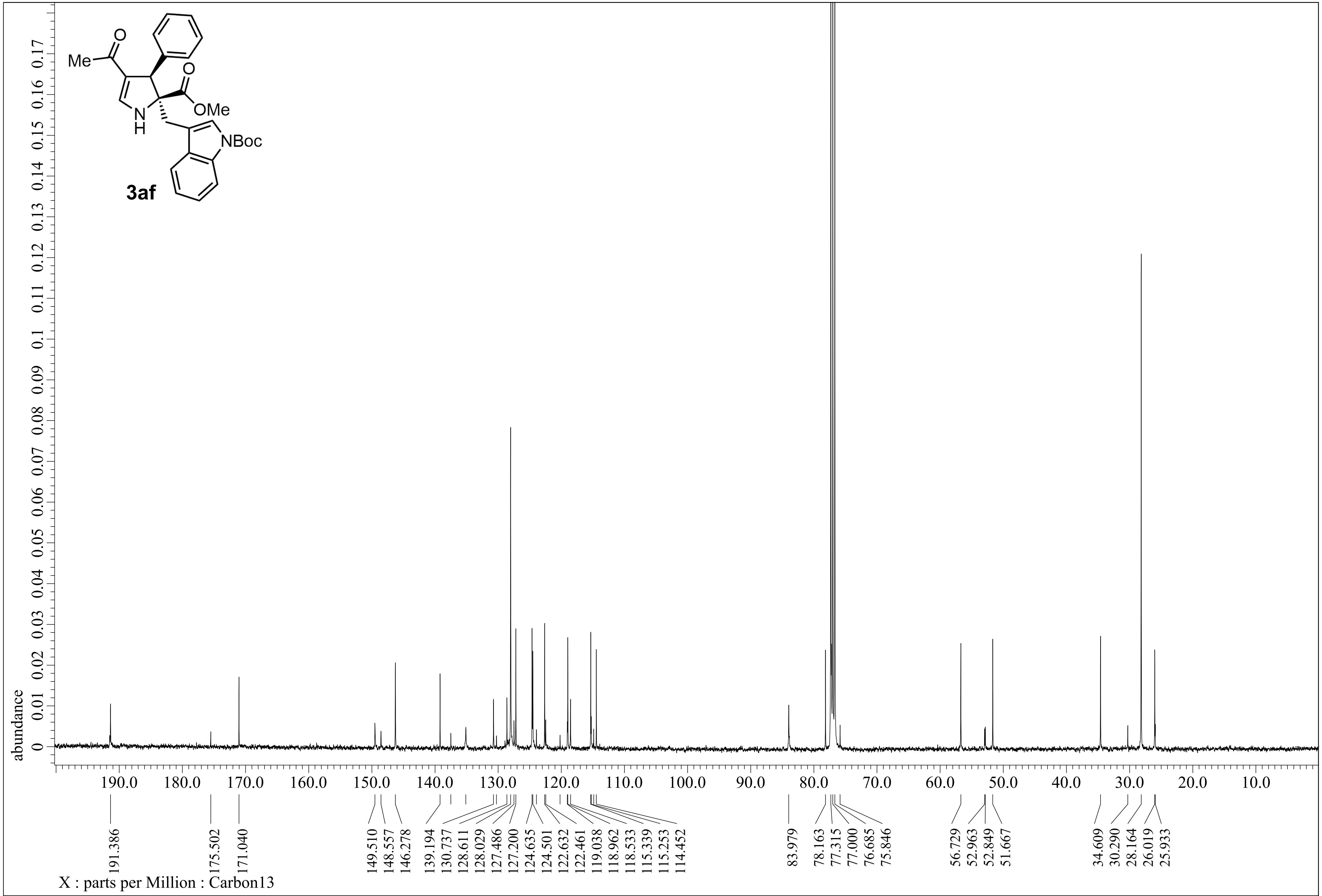
Me
O
O
OMe
N
H
NBoc
3af
abundance
0.17 0.16 0.15 0.14 0.13 0.12 0.11 0.1 0.09 0.08 0.07 0.06 0.05 0.04 0.03 0.02 0.01 0
190.0 180.0 170.0 160.0 150.0 140.0 130.0 120.0 110.0 100.0 90.0 80.0 70.0 60.0 50.0 40.0 30.0 20.0 10.0
191.386
175.502
171.040
149.510
148.557
146.278
139.194
130.737
128.611
128.029
127.486
127.200
124.635
124.501
122.632
122.461
119.038
118.962
118.533
115.339
115.253
114.452
83.979
78.163
77.315
77.000
76.685
75.846
56.729
52.963
52.849
51.667
34.609
30.290
28.164
26.019
25.933
X : parts per Million : Carbon13

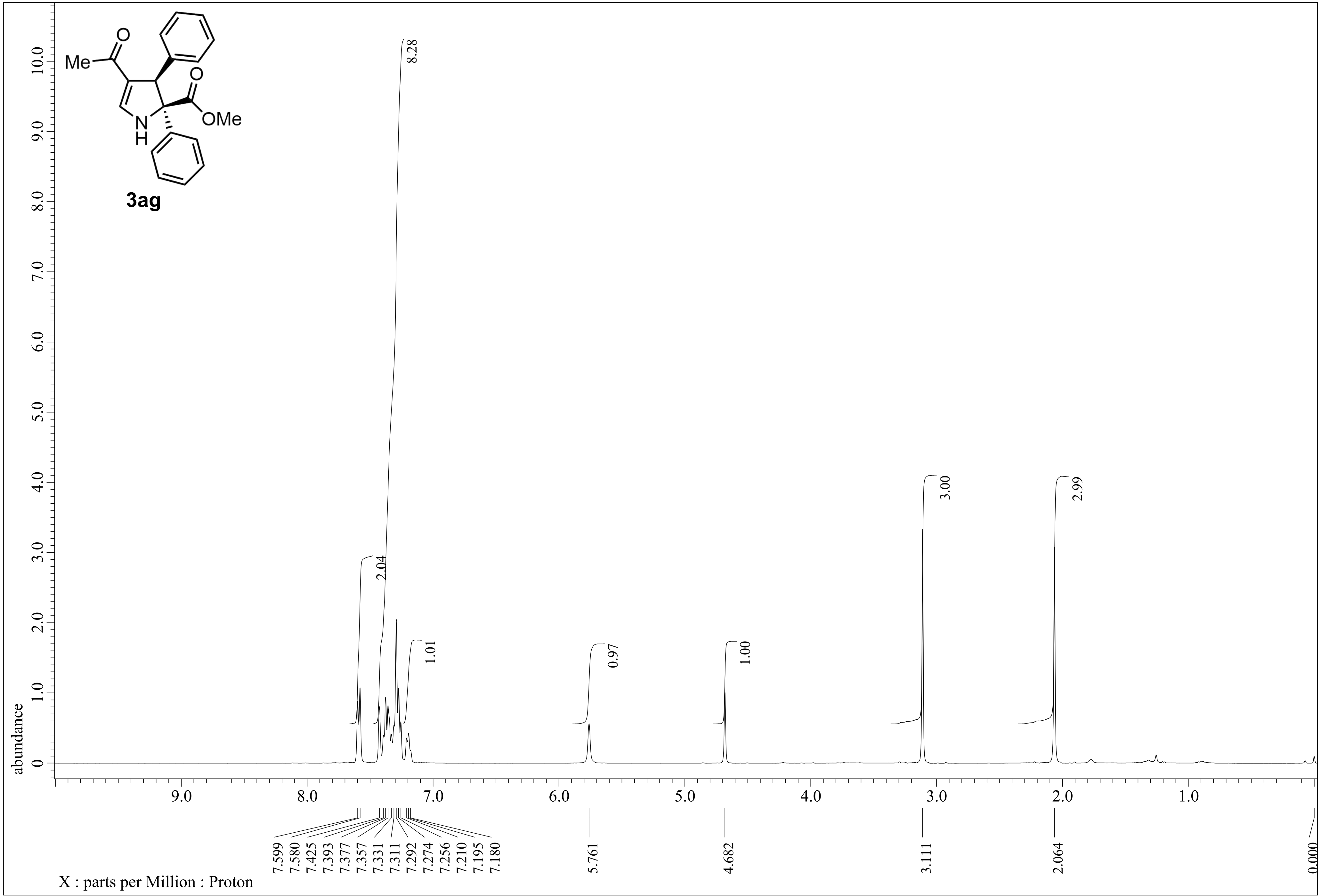
Me
O
O
OMe
N
H
3ag
abundance
10.0
9.0
8.0
7.0
6.0
5.0
4.0
3.0
2.0
1.0
0
8.28
2.04
1.01
0.97
1.00
3.00
2.99
9.0
8.0
7.0
6.0
5.0
4.0
3.0
2.0
1.0
7.599
7.580
7.425
7.393
7.377
7.357
7.331
7.311
7.292
7.274
7.256
7.210
7.195
7.180
5.761
4.682
3.111
2.064
0.000
X : parts per Million : Proton

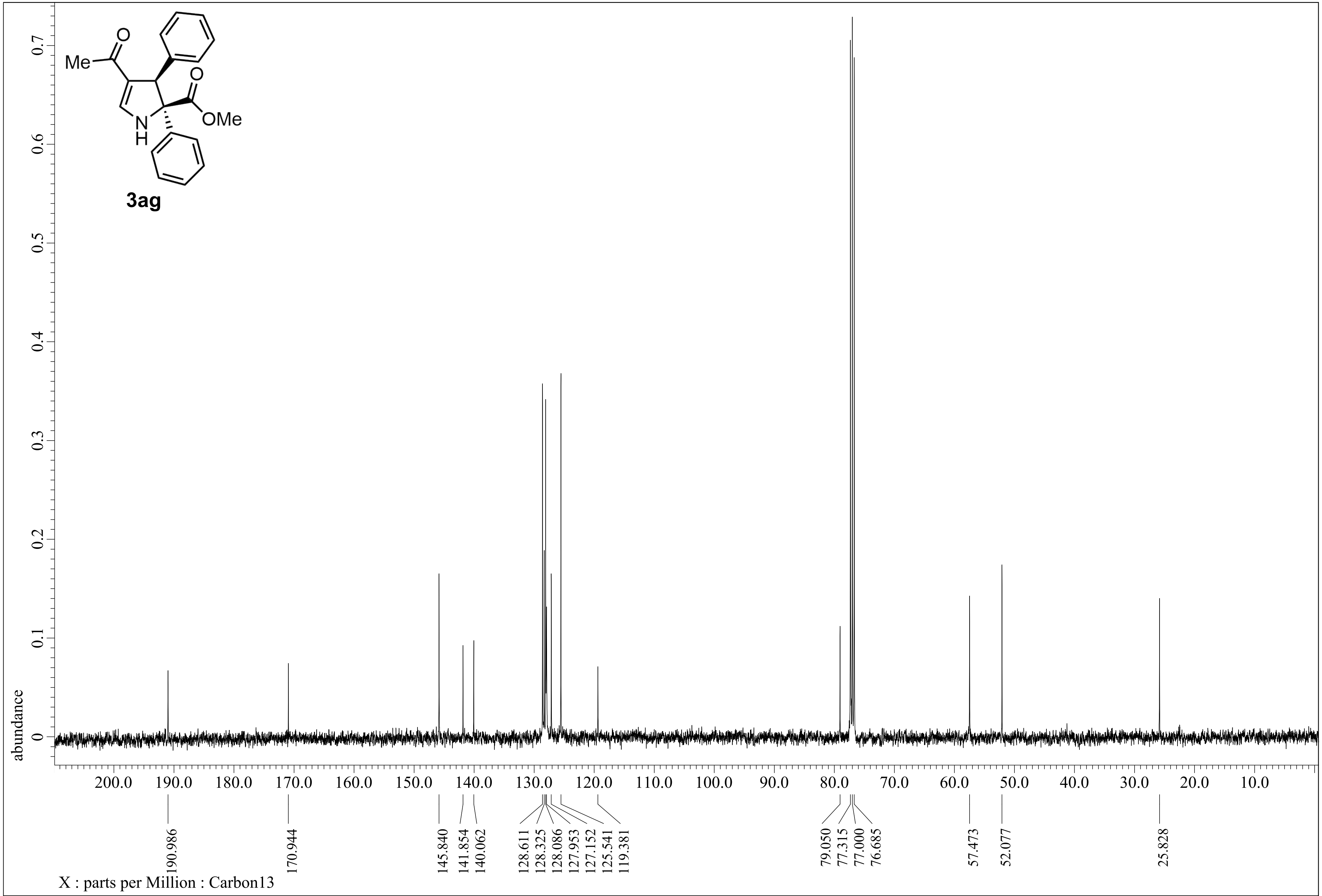
Me
O
O
OMe
N
H
3ag
abundance
0.7
0.6
0.5
0.4
0.3
0.2
0.1
0
200.0 190.0 180.0 170.0 160.0 150.0 140.0 130.0 120.0 110.0 100.0 90.0 80.0 70.0 60.0 50.0 40.0 30.0 20.0 10.0
190.986
170.944
145.840
141.854
140.062
128.611
128.325
128.086
127.953
127.152
125.541
119.381
79.050
77.315
77.000
76.685
57.473
52.077
25.828
X : parts per Million : Carbon13

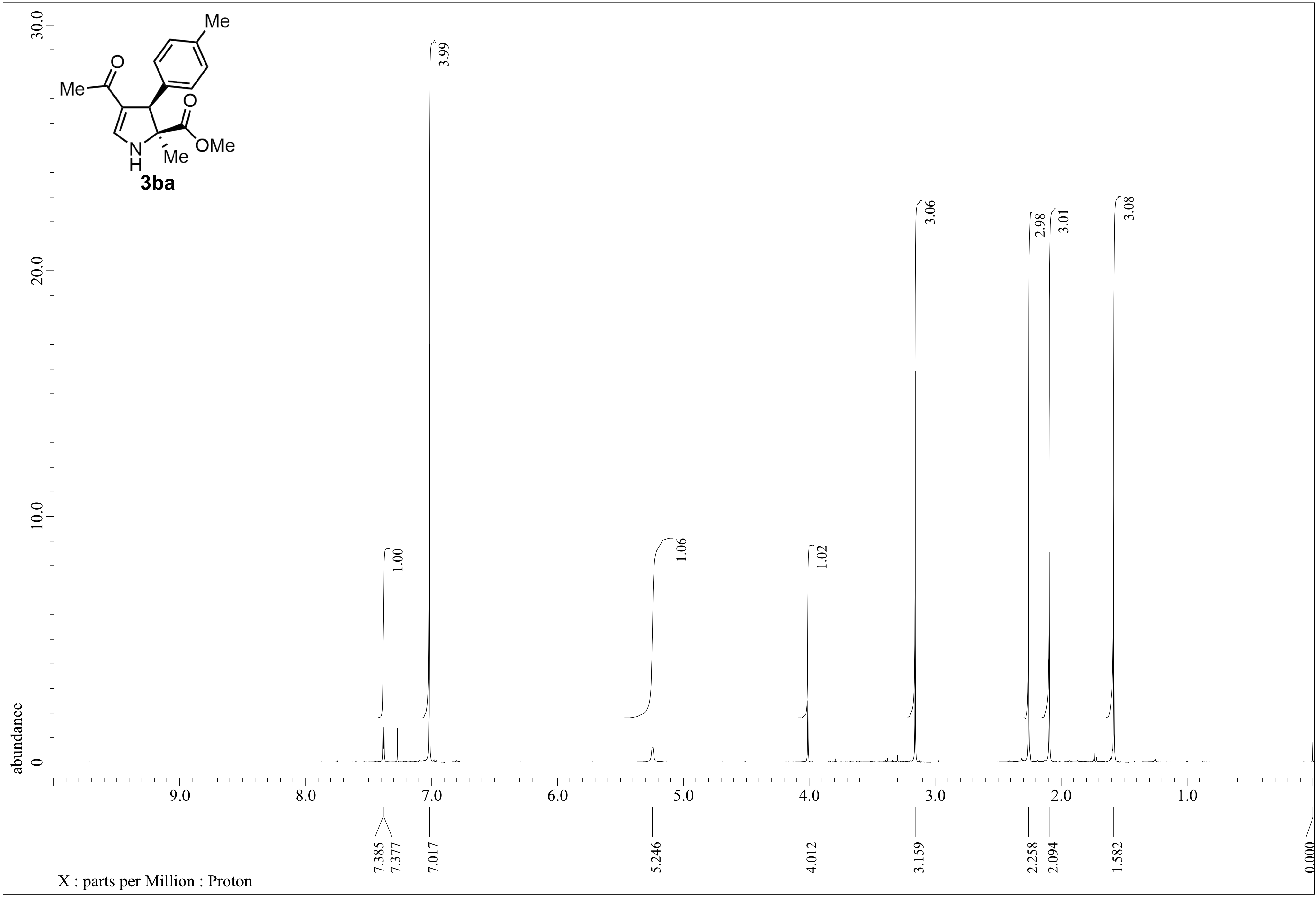

Me
O
Me
O
OMe
N
H
Me
3ba
30.0
20.0
10.0
0
abundance
1.00
3.99
1.06
1.02
3.06
2.98
3.01
3.08
9.0
8.0
7.0
6.0
5.0
4.0
3.0
2.0
1.0
7.385
7.377
7.017
5.246
4.012
3.159
2.258
2.094
1.582
0.000
X : parts per Million : Proton

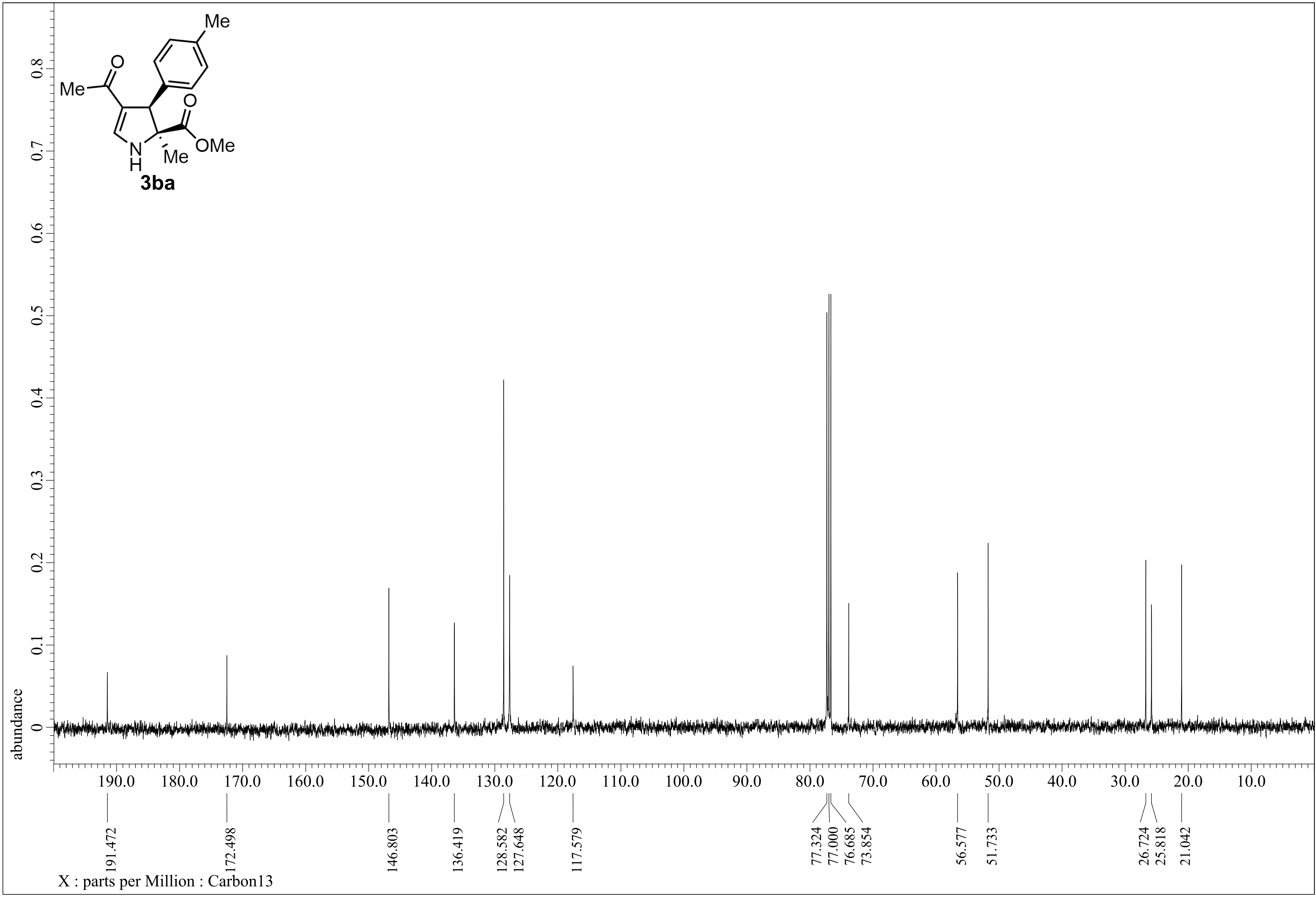
Me
O
Me
O
OMe
N
H
Me
3ba
abundance
0.8
0.7
0.6
0.5
0.4
0.3
0.2
0.1
0
190.0
180.0
170.0
160.0
150.0
140.0
130.0
120.0
110.0
100.0
90.0
80.0
70.0
60.0
50.0
40.0
30.0
20.0
10.0
191.472
172.498
146.803
136.419
128.582
127.648
117.579
77.324
77.000
76.685
73.854
56.577
51.733
26.724
25.818
21.042
X : parts per Million : Carbon13

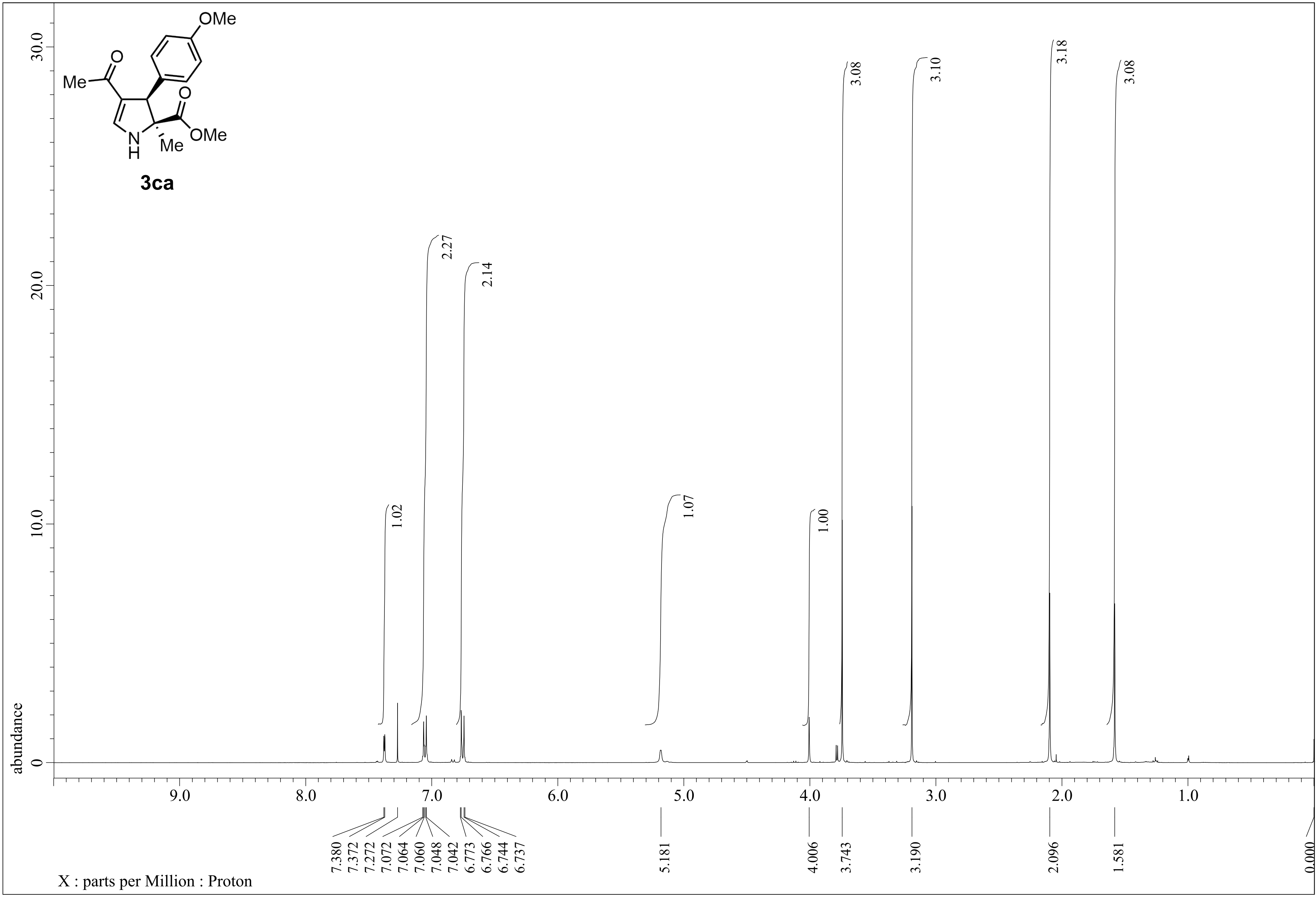
OMe
Me
O
O
OMe
N
H
Me
3ca
30.0
20.0
10.0
0
abundance
3.08
3.10
3.18
3.08
2.27
2.14
1.02
1.07
1.00
9.0
8.0
7.0
6.0
5.0
4.0
3.0
2.0
1.0
7.380
7.372
7.272
7.072
7.064
7.060
7.048
7.042
6.773
6.766
6.744
6.737
5.181
4.006
3.743
3.190
2.096
1.581
0.000
X : parts per Million : Proton

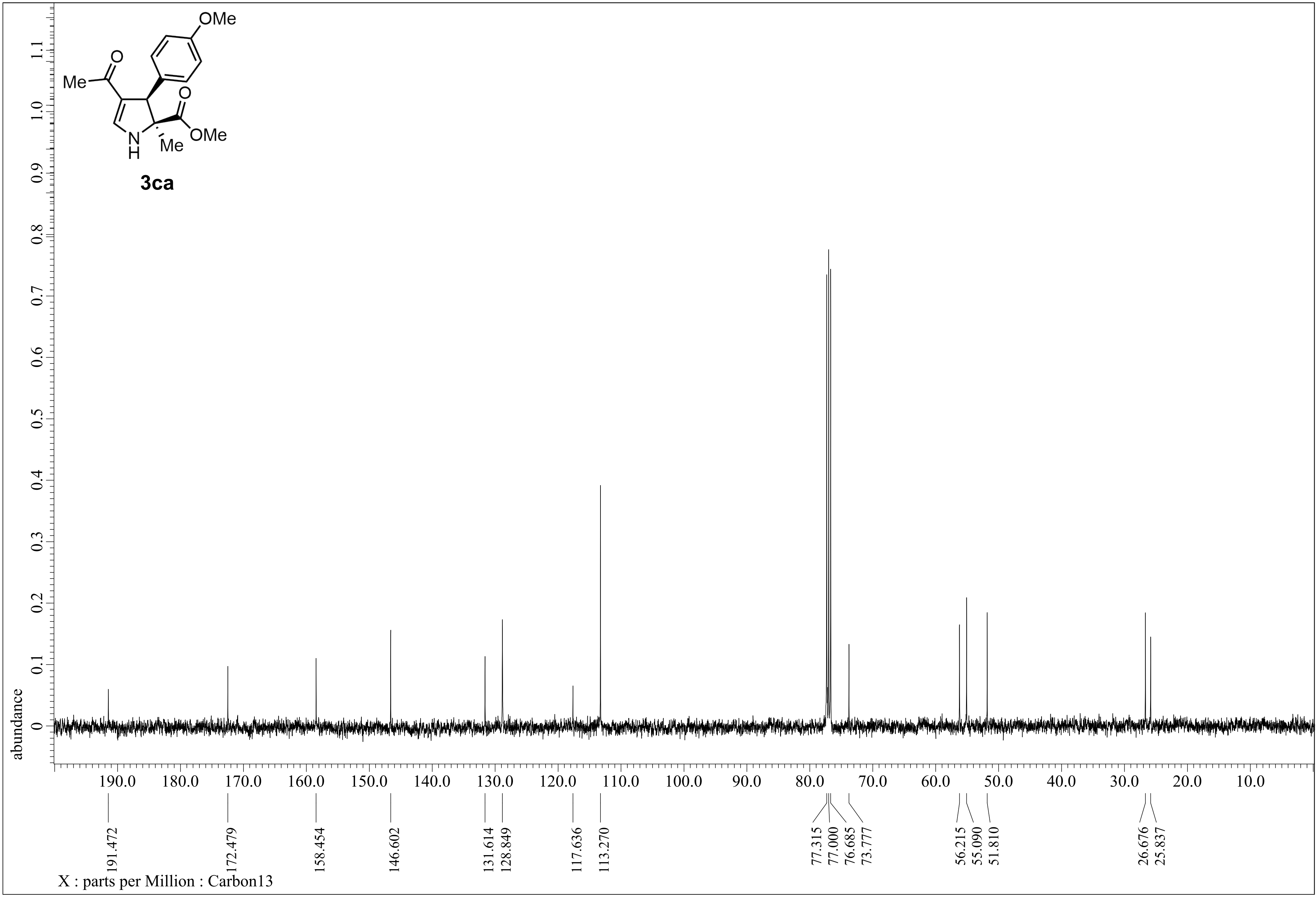
OMe
Me
O
O
N
H
Me
OMe
3ca
abundance
1.1
1.0
0.9
0.8
0.7
0.6
0.5
0.4
0.3
0.2
0.1
0
190.0
180.0
170.0
160.0
150.0
140.0
130.0
120.0
110.0
100.0
90.0
80.0
70.0
60.0
50.0
40.0
30.0
20.0
10.0
191.472
172.479
158.454
146.602
131.614
128.849
117.636
113.270
77.315
77.000
76.685
73.777
56.215
55.090
51.810
26.676
25.837
X : parts per Million : Carbon13

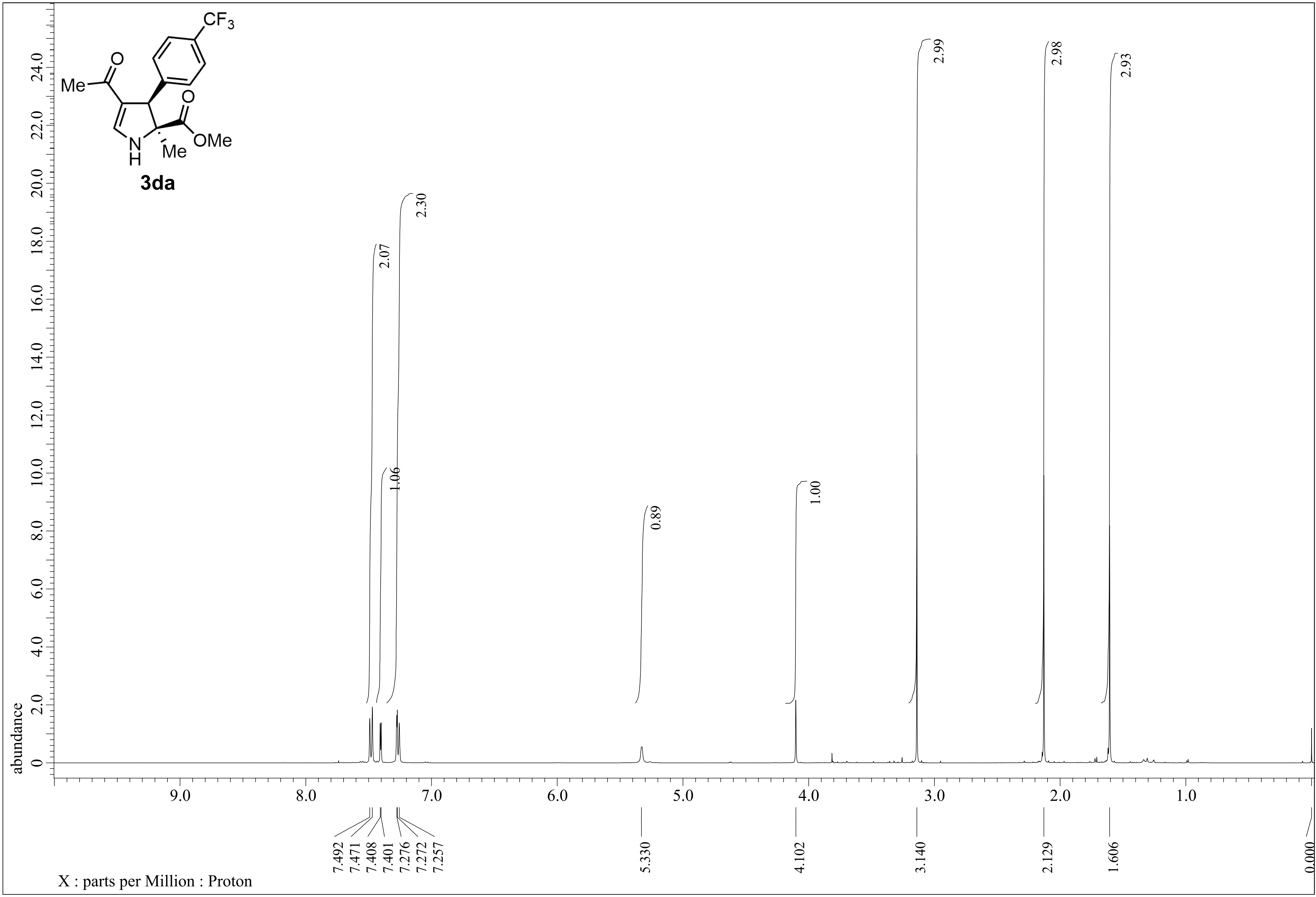
CF3
Me
O
O
OMe
N
H
Me
3da
abundance
24.0
22.0
20.0
18.0
16.0
14.0
12.0
10.0
8.0
6.0
4.0
2.0
0
2.99
2.98
2.93
2.30
2.07
1.06
0.89
1.00
9.0
8.0
7.0
6.0
5.0
4.0
3.0
2.0
1.0
7.492
7.471
7.408
7.401
7.276
7.272
7.257
5.330
4.102
3.140
2.129
1.606
0.000
X : parts per Million : Proton

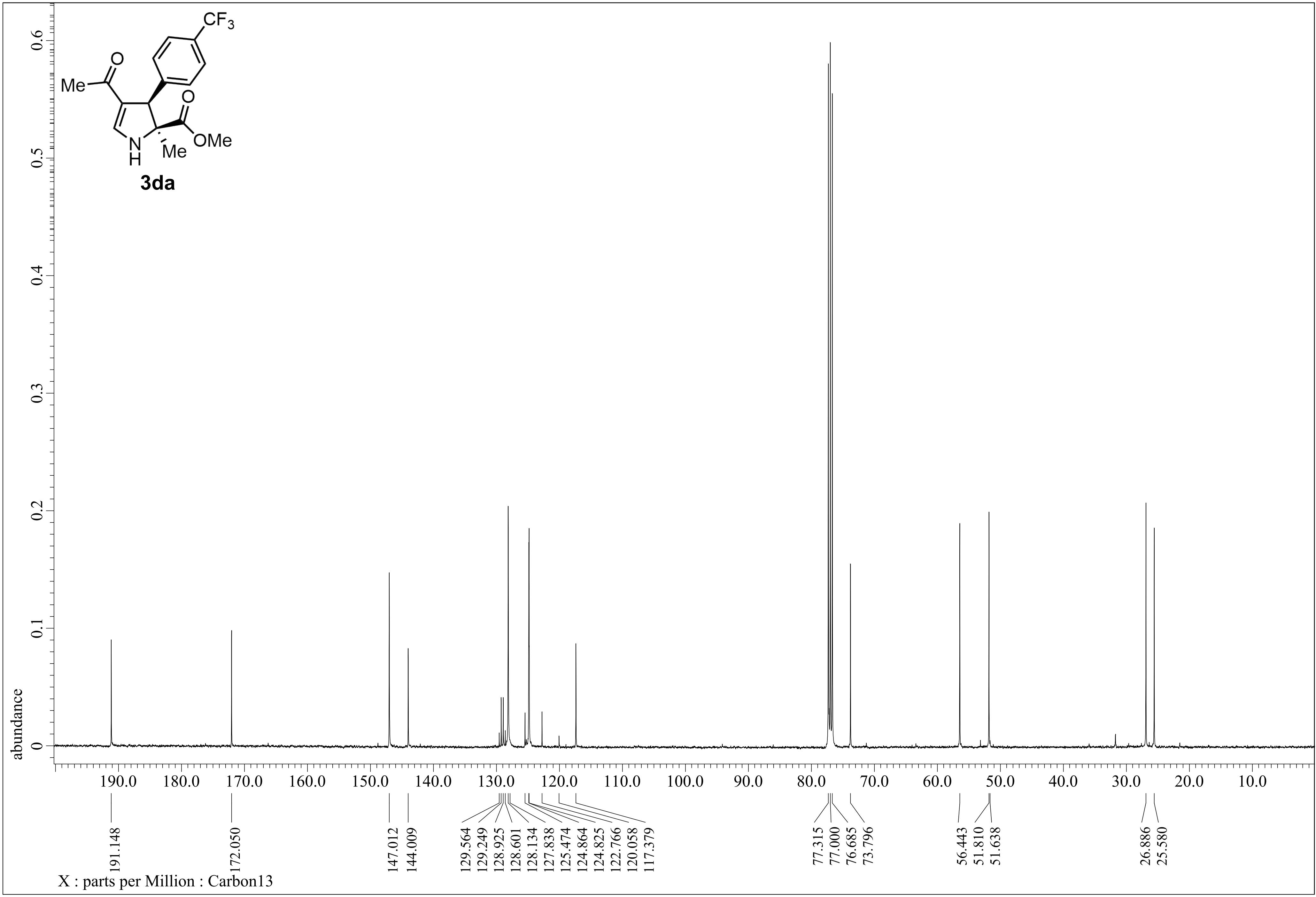
CF3
O
Me
O
N
H
Me
OMe
3da
abundance
0.6
0.5
0.4
0.3
0.2
0.1
0
190.0 180.0 170.0 160.0 150.0 140.0 130.0 120.0 110.0 100.0 90.0 80.0 70.0 60.0 50.0 40.0 30.0 20.0 10.0
191.148
172.050
147.012
144.009
129.564
129.249
128.925
128.601
128.134
127.838
125.474
124.864
124.825
122.766
120.058
117.379
77.315
77.000
76.685
73.796
56.443
51.810
51.638
26.886
25.580
X : parts per Million : Carbon13

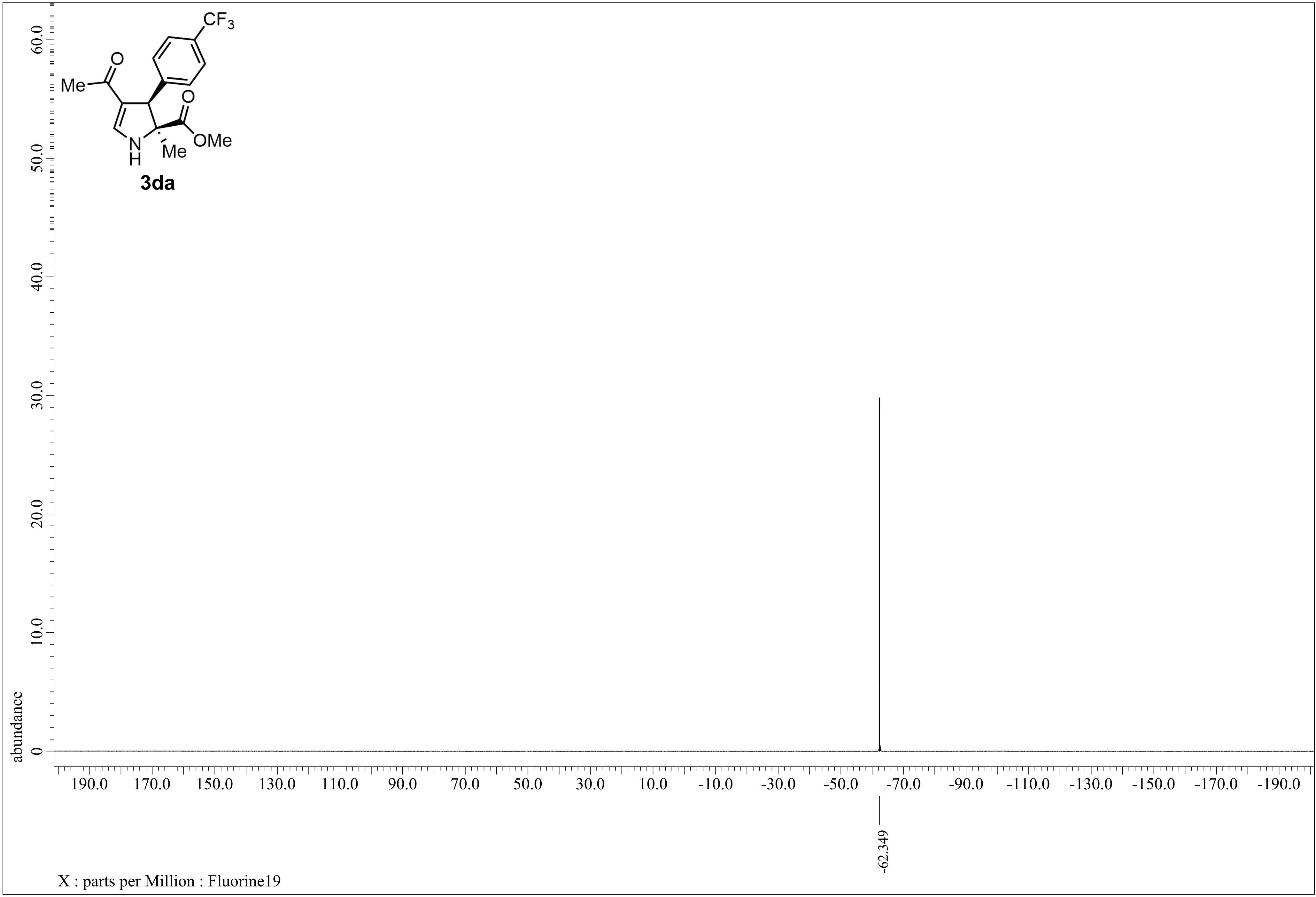
3da
abundance
X : parts per Million : Fluorine19
-62.349

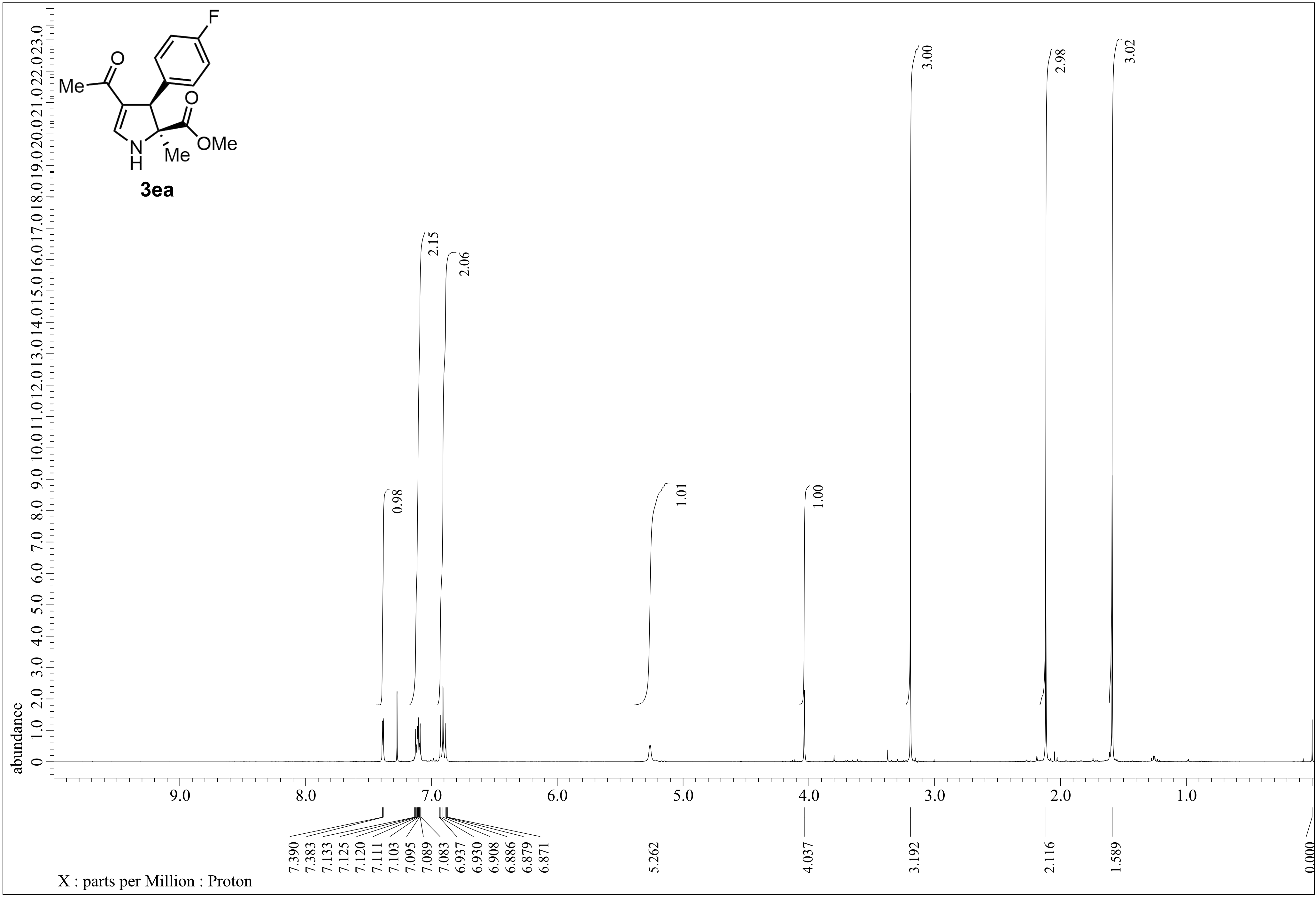
3ea
abundance
X : parts per Million : Proton
9.0
8.0
7.0
6.0
5.0
4.0
3.0
2.0
1.0
0.98
2.15
2.06
1.01
1.00
3.00
2.98
3.02
7.390
7.383
7.133
7.125
7.120
7.111
7.103
7.095
7.089
7.083
6.937
6.930
6.908
6.886
6.879
6.871
5.262
4.037
3.192
2.116
1.589
0.000

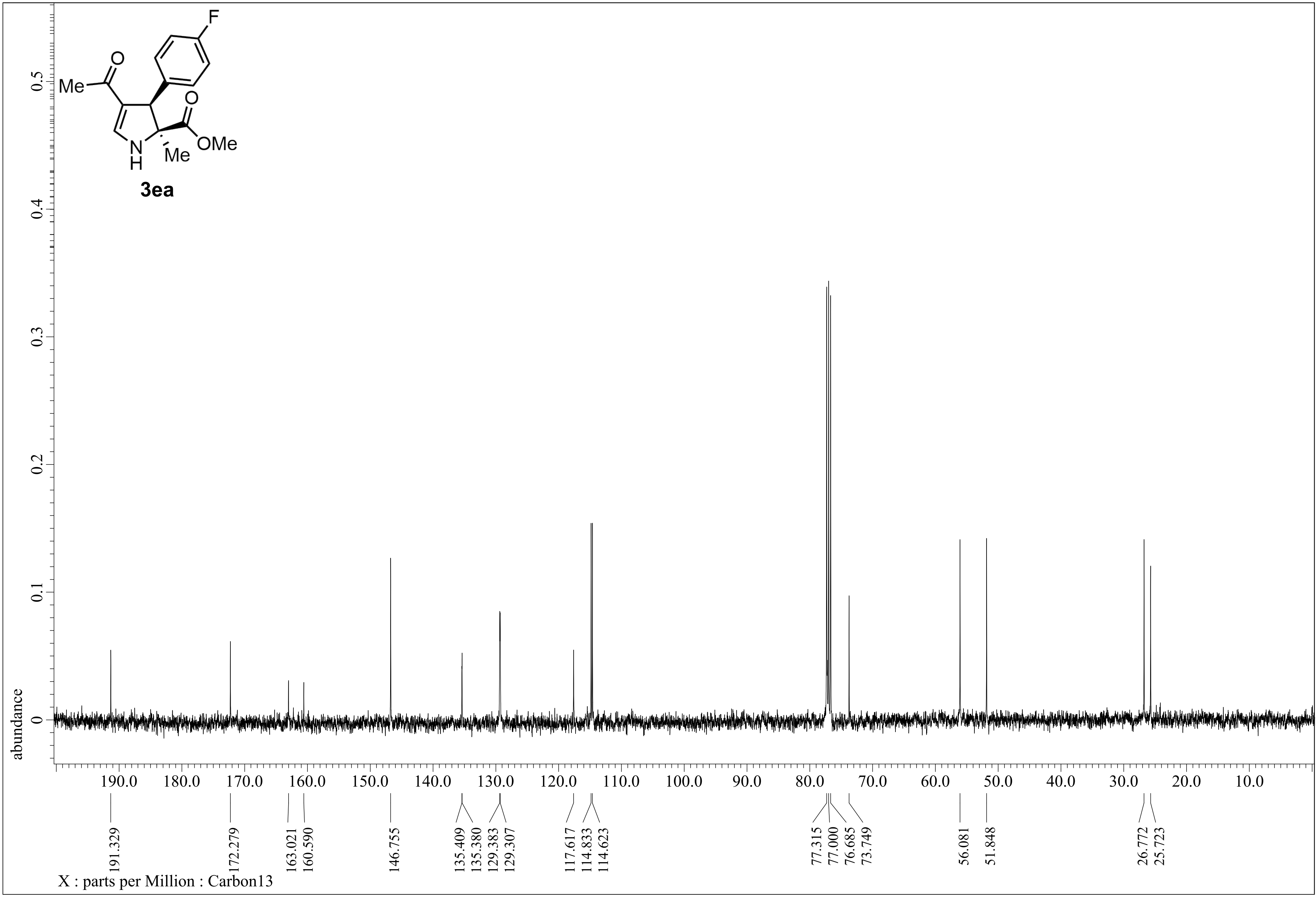

F
O
Me
O
N
H
Me
OMe
3ea
abundance
0.5
0.4
0.3
0.2
0.1
0
190.0 180.0 170.0 160.0 150.0 140.0 130.0 120.0 110.0 100.0 90.0 80.0 70.0 60.0 50.0 40.0 30.0 20.0 10.0
191.329
172.279
163.021
160.590
146.755
135.409
135.380
129.383
129.307
117.617
114.833
114.623
77.315
77.000
76.685
73.749
56.081
51.848
26.772
25.723
X : parts per Million : Carbon13

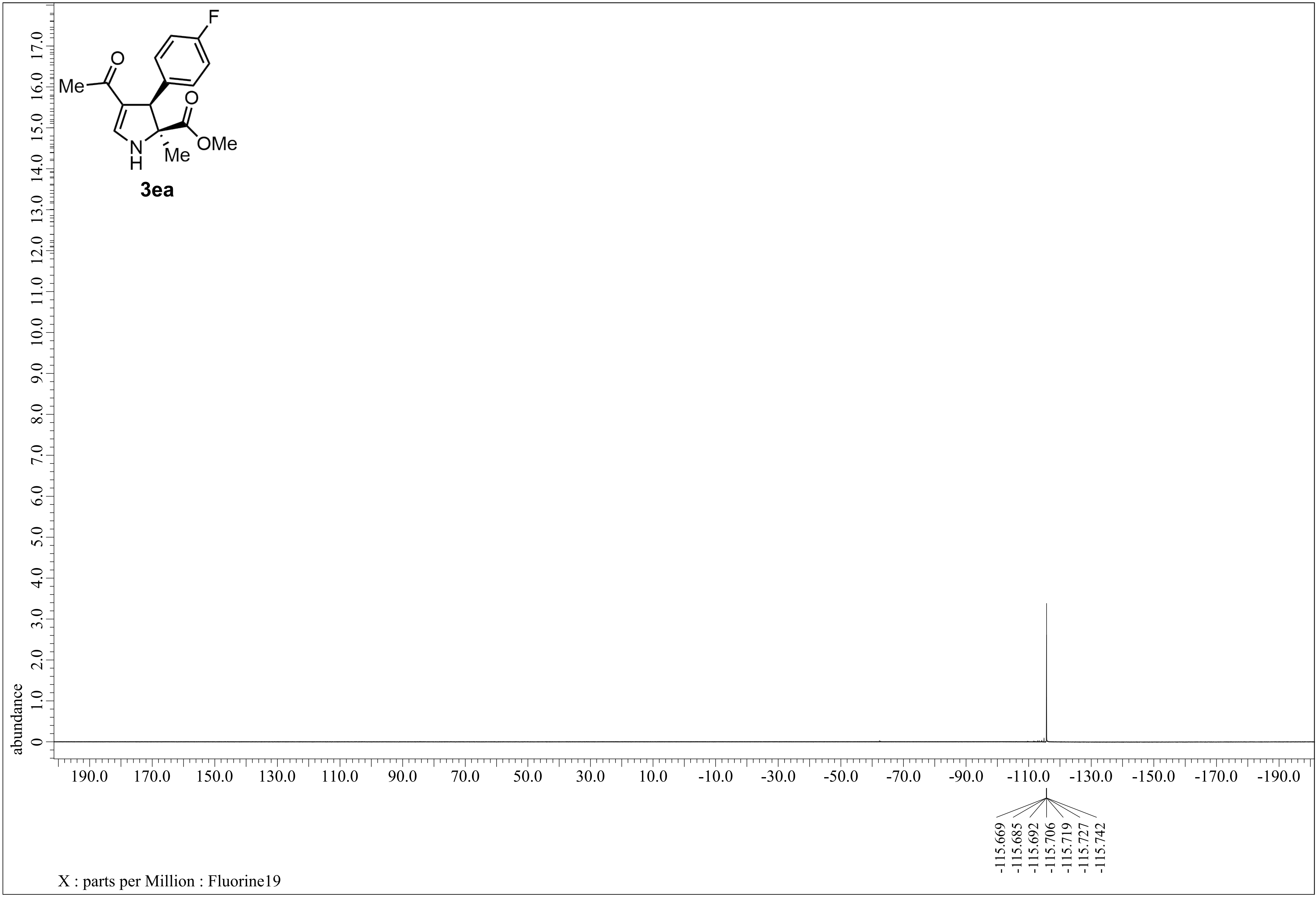
3ea
abundance
-115.669
-115.685
-115.692
-115.706
-115.719
-115.727
-115.742
X : parts per Million : Fluorine19

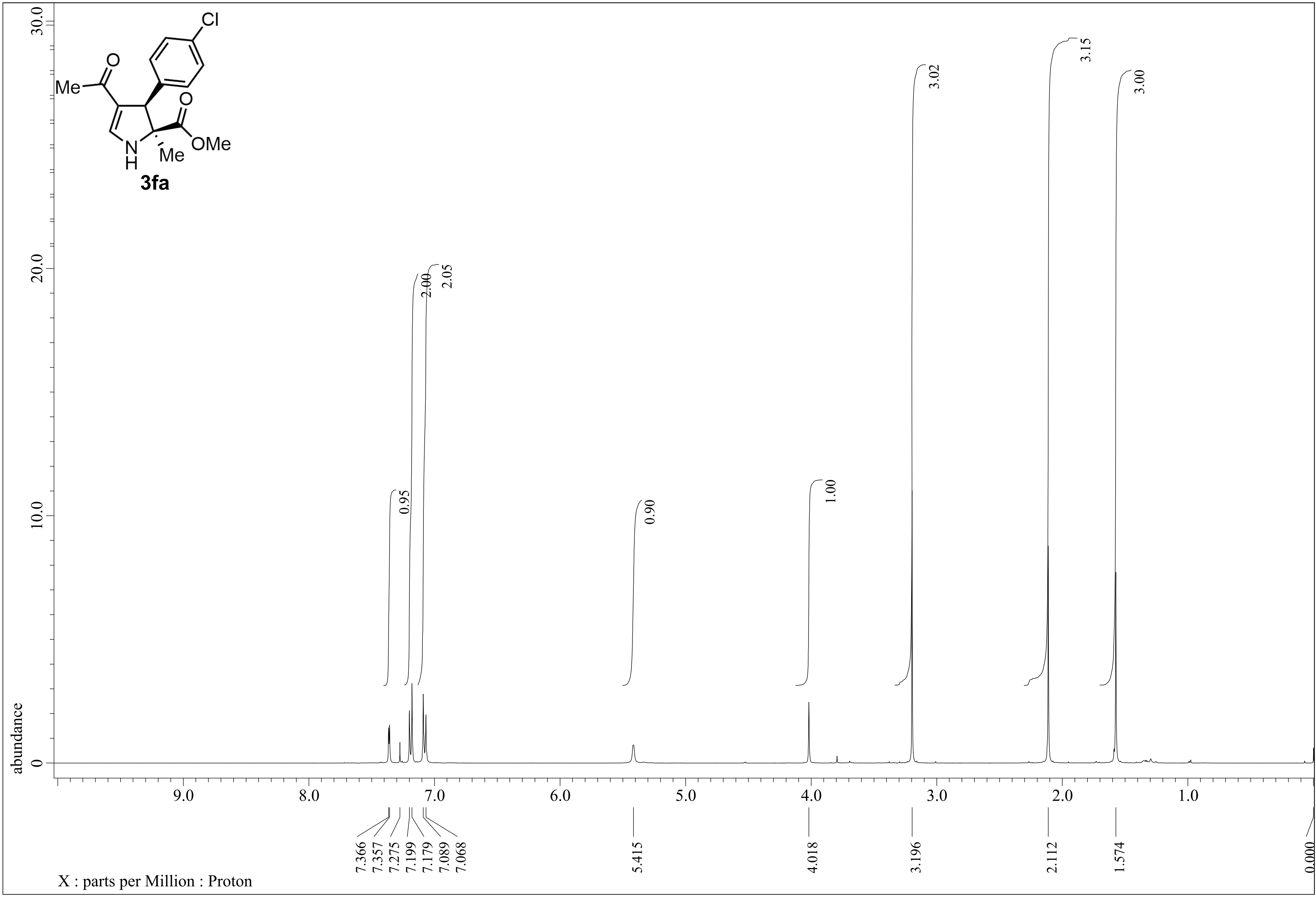
Cl
O
Me
O
NH
Me
OMe
3fa
30.0
20.0
10.0
0
abundance
0.95
2.00
2.05
0.90
1.00
3.02
3.15
3.00
9.0
8.0
7.0
6.0
5.0
4.0
3.0
2.0
1.0
7.366
7.357
7.275
7.199
7.179
7.089
7.068
5.415
4.018
3.196
2.112
1.574
0.000
X : parts per Million : Proton

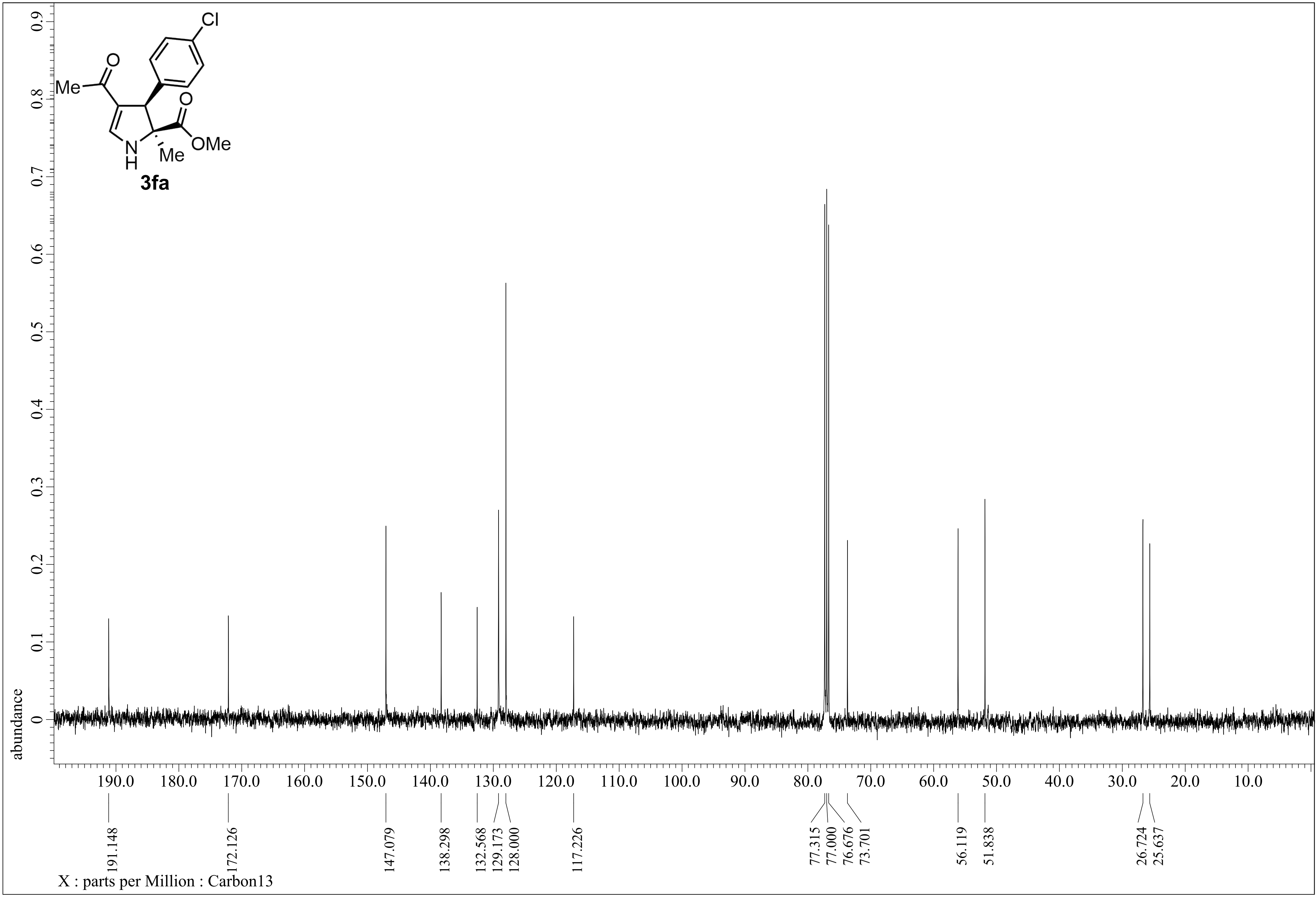
Cl
Me
O
O
OMe
N
H
Me
3fa
abundance
0
0.1
0.2
0.3
0.4
0.5
0.6
0.7
0.8
0.9
190.0
180.0
170.0
160.0
150.0
140.0
130.0
120.0
110.0
100.0
90.0
80.0
70.0
60.0
50.0
40.0
30.0
20.0
10.0
191.148
172.126
147.079
138.298
132.568
129.173
128.000
117.226
77.315
77.000
76.676
73.701
56.119
51.838
26.724
25.637
X : parts per Million : Carbon13

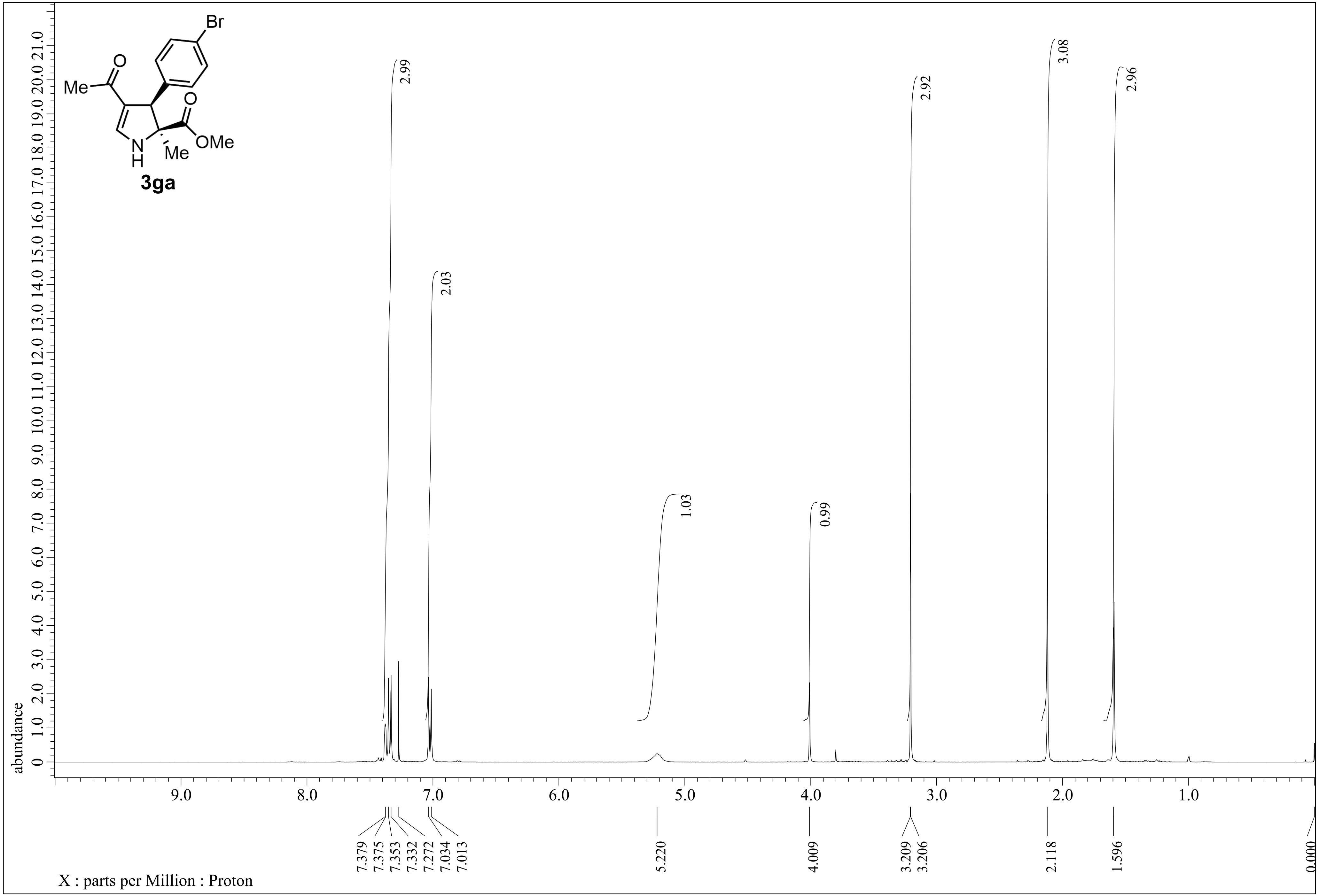
3ga
Br
O
Me
O
OMe
N
H
Me
abundance
X : parts per Million : Proton
9.0
8.0
7.0
6.0
5.0
4.0
3.0
2.0
1.0
2.99
2.03
1.03
0.99
2.92
3.08
2.96
7.379
7.375
7.353
7.332
7.272
7.034
7.013
5.220
4.009
3.209
3.206
2.118
1.596
0.000

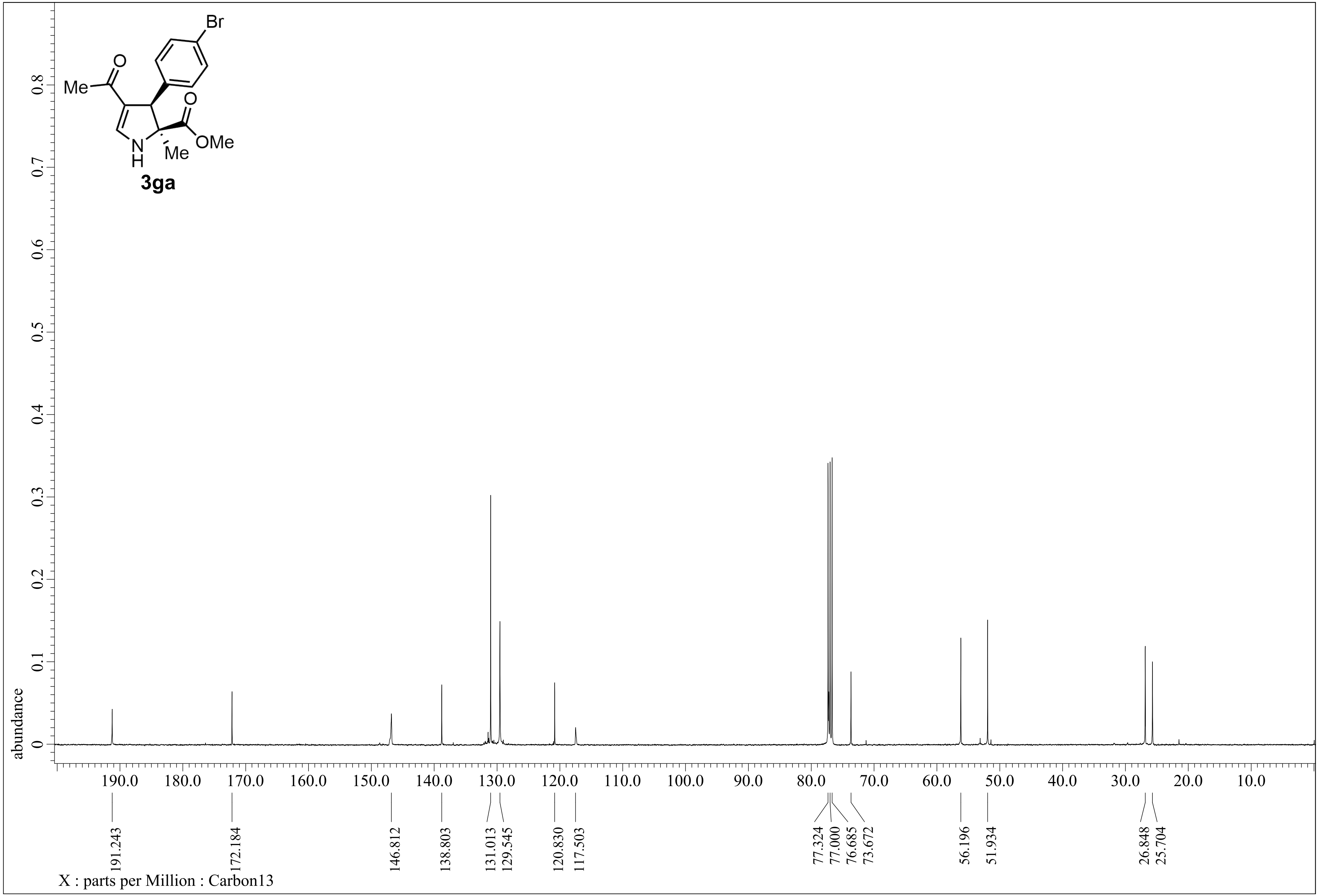

Br
O
Me
O
OMe
N
H
Me
3ga
0.8
0.7
0.6
0.5
0.4
0.3
0.2
0.1
0
abundance
190.0
180.0
170.0
160.0
150.0
140.0
130.0
120.0
110.0
100.0
90.0
80.0
70.0
60.0
50.0
40.0
30.0
20.0
10.0
191.243
172.184
146.812
138.803
131.013
129.545
120.830
117.503
77.324
77.000
76.685
73.672
56.196
51.934
26.848
25.704
X : parts per Million : Carbon13

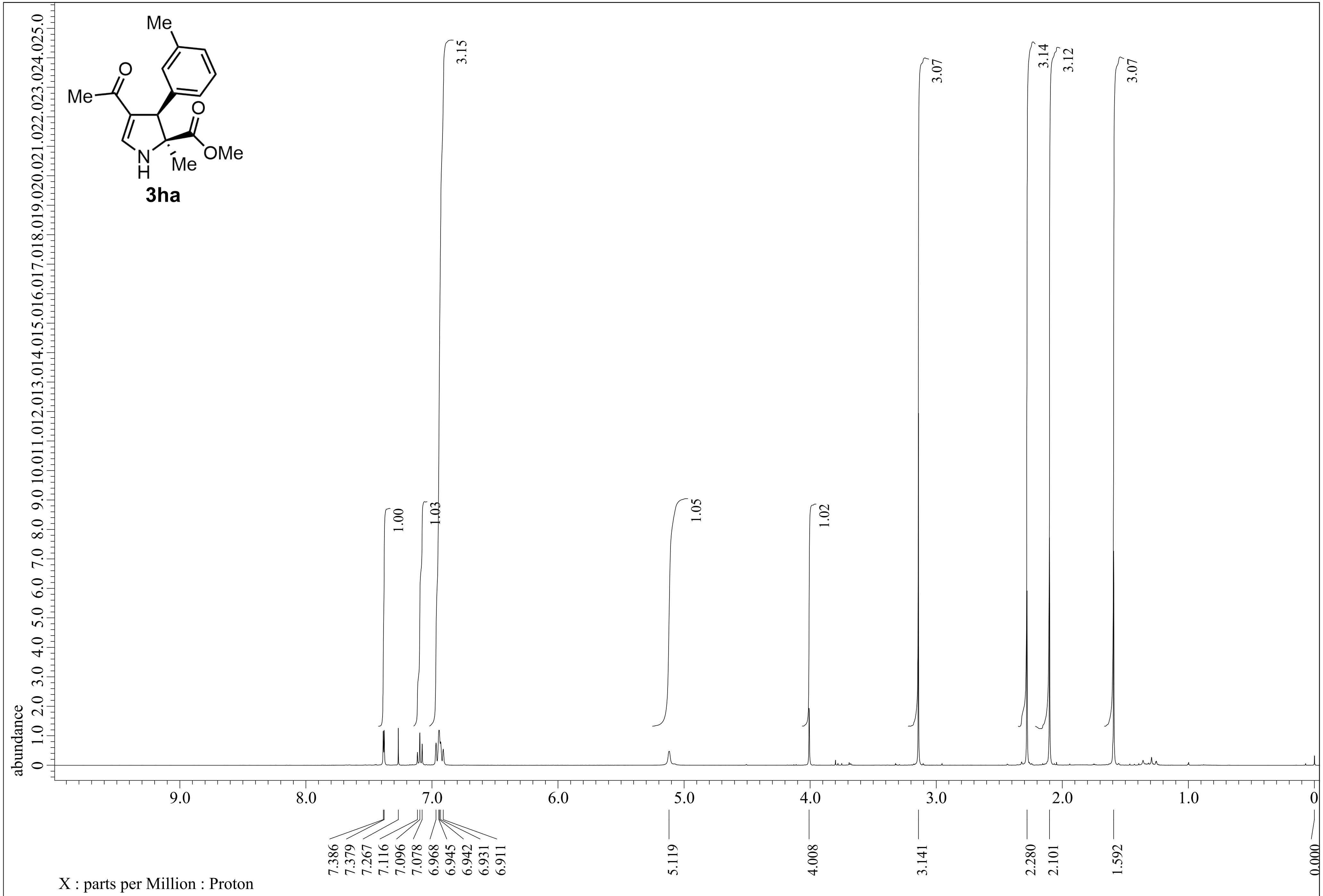
Me
O
Me
O
OMe
N
H
Me
3ha
abundance
3.15
1.00
1.03
1.05
1.02
3.07
3.14
3.12
3.07
9.0
8.0
7.0
6.0
5.0
4.0
3.0
2.0
1.0
0
7.386
7.379
7.267
7.116
7.096
7.078
6.968
6.945
6.942
6.931
6.911
5.119
4.008
3.141
2.280
2.101
1.592
0.000
X : parts per Million : Proton

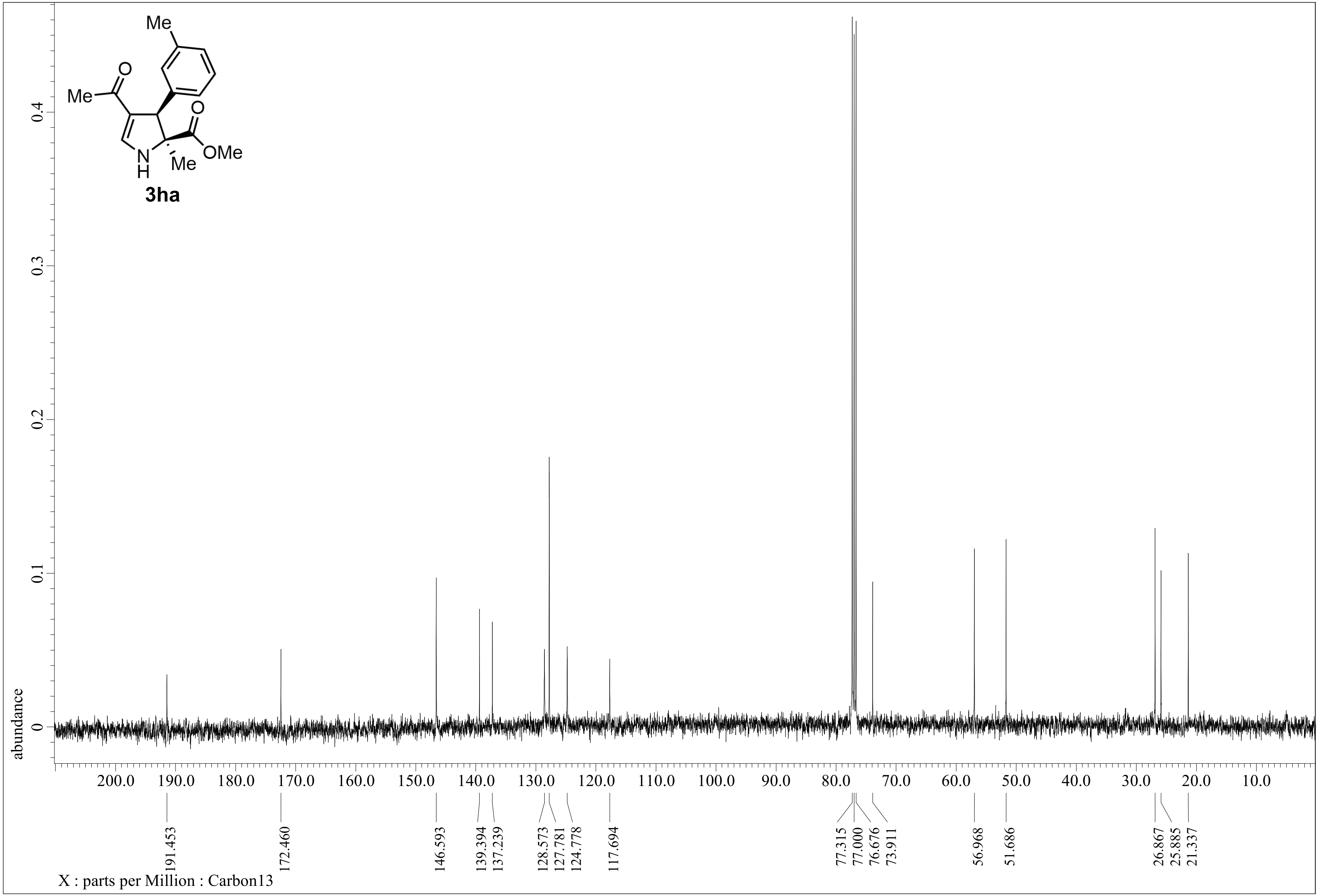
Me
O
Me
O
OMe
N
H
Me
3ha
abundance
0
0.1
0.2
0.3
0.4
200.0
190.0
180.0
170.0
160.0
150.0
140.0
130.0
120.0
110.0
100.0
90.0
80.0
70.0
60.0
50.0
40.0
30.0
20.0
10.0
191.453
172.460
146.593
139.394
137.239
128.573
127.781
124.778
117.694
77.315
77.000
76.676
73.911
56.968
51.686
26.867
25.885
21.337
X : parts per Million : Carbon13

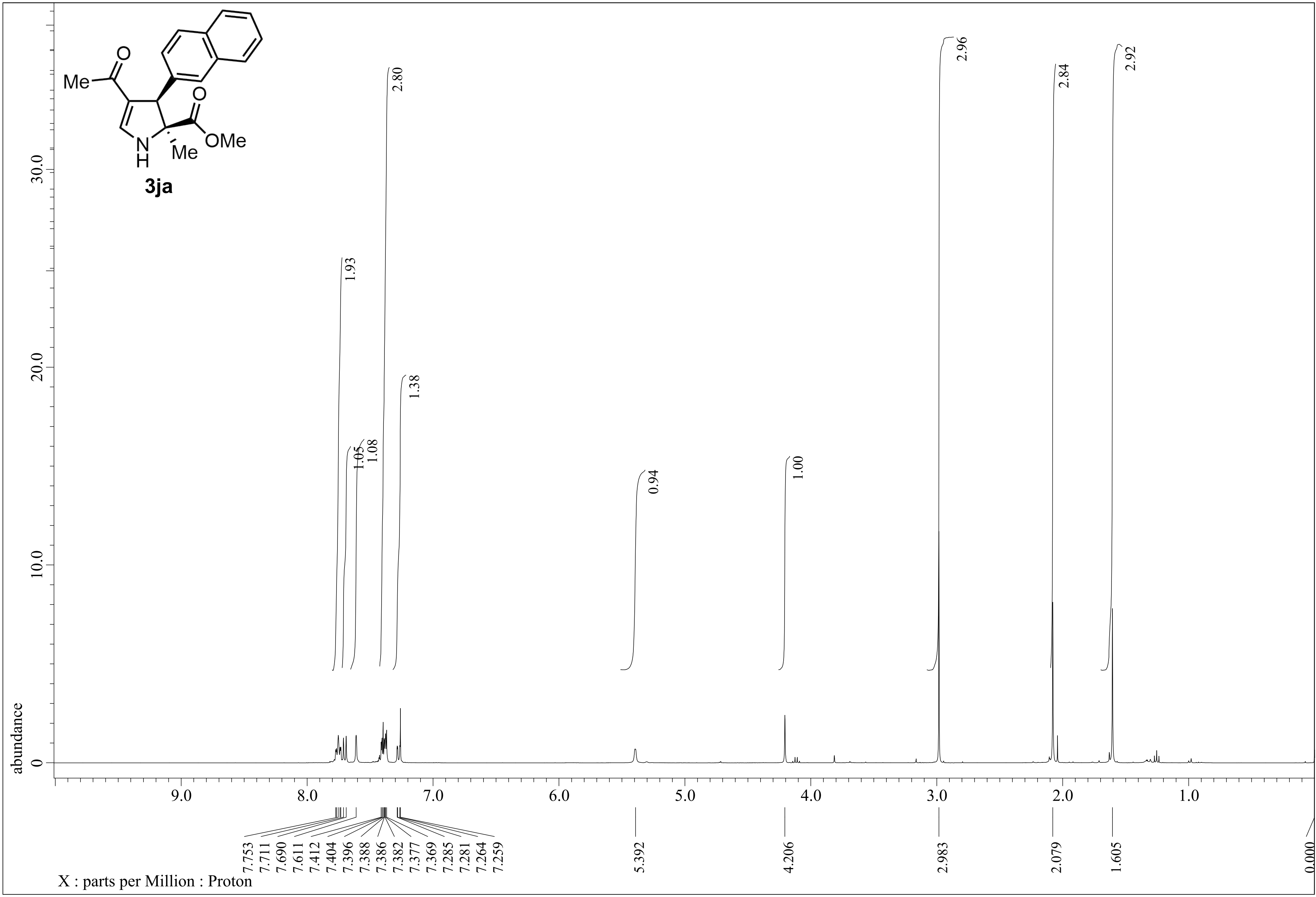
Me
O
O
N
H
Me
OMe
3ja
abundance
X : parts per Million : Proton
9.0
8.0
7.0
6.0
5.0
4.0
3.0
2.0
1.0
30.0
20.0
10.0
0
1.93
1.05
1.08
2.80
1.38
0.94
1.00
2.96
2.84
2.92
7.753
7.711
7.690
7.611
7.412
7.404
7.396
7.388
7.386
7.382
7.377
7.369
7.285
7.281
7.264
7.259
5.392
4.206
2.983
2.079
1.605
0.000

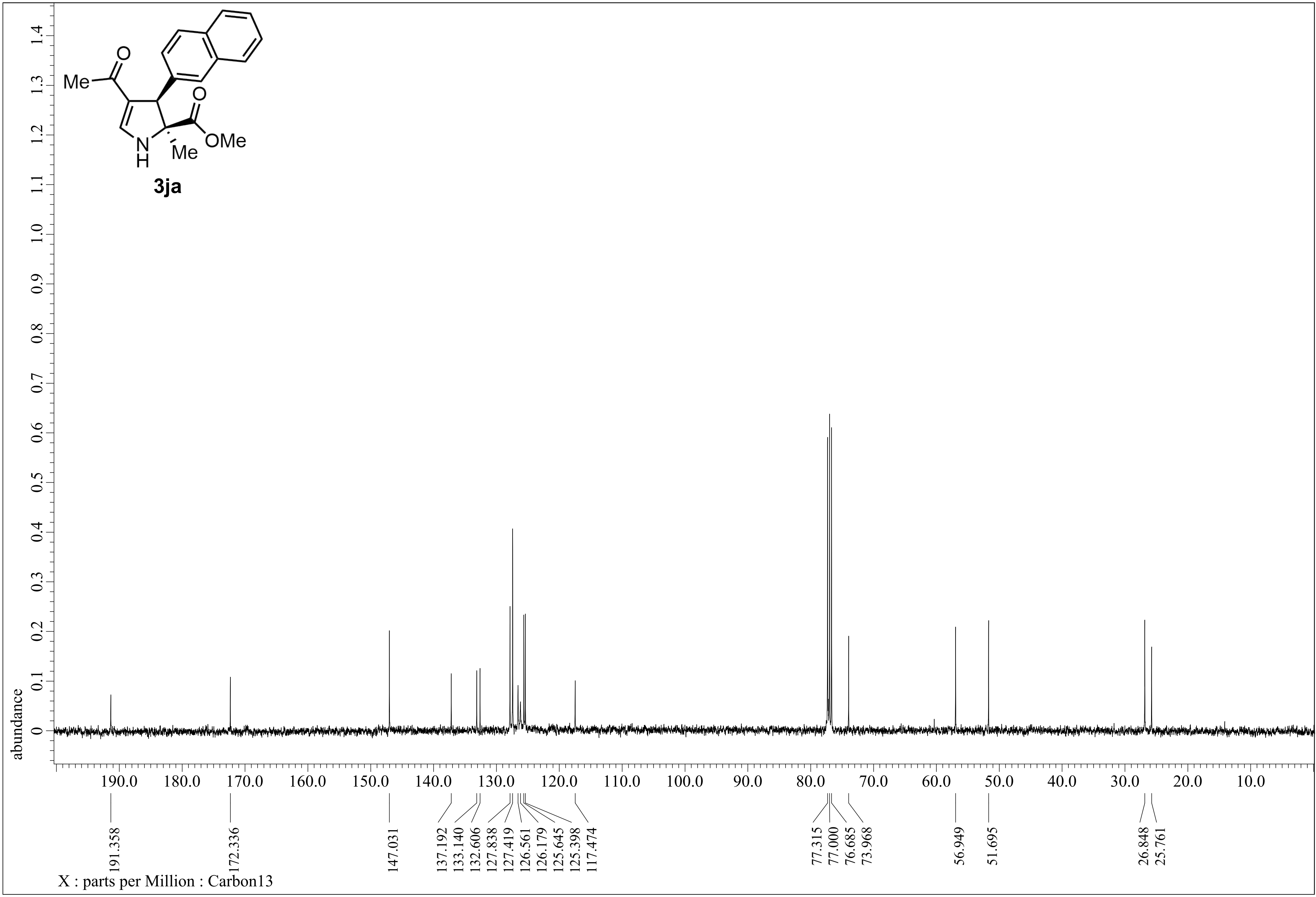

3ja
abundance
X : parts per Million : Carbon13
191.358
172.336
147.031
137.192
133.140
132.606
127.838
127.419
126.561
126.179
125.645
125.398
117.474
77.315
77.000
76.685
73.968
56.949
51.695
26.848
25.761

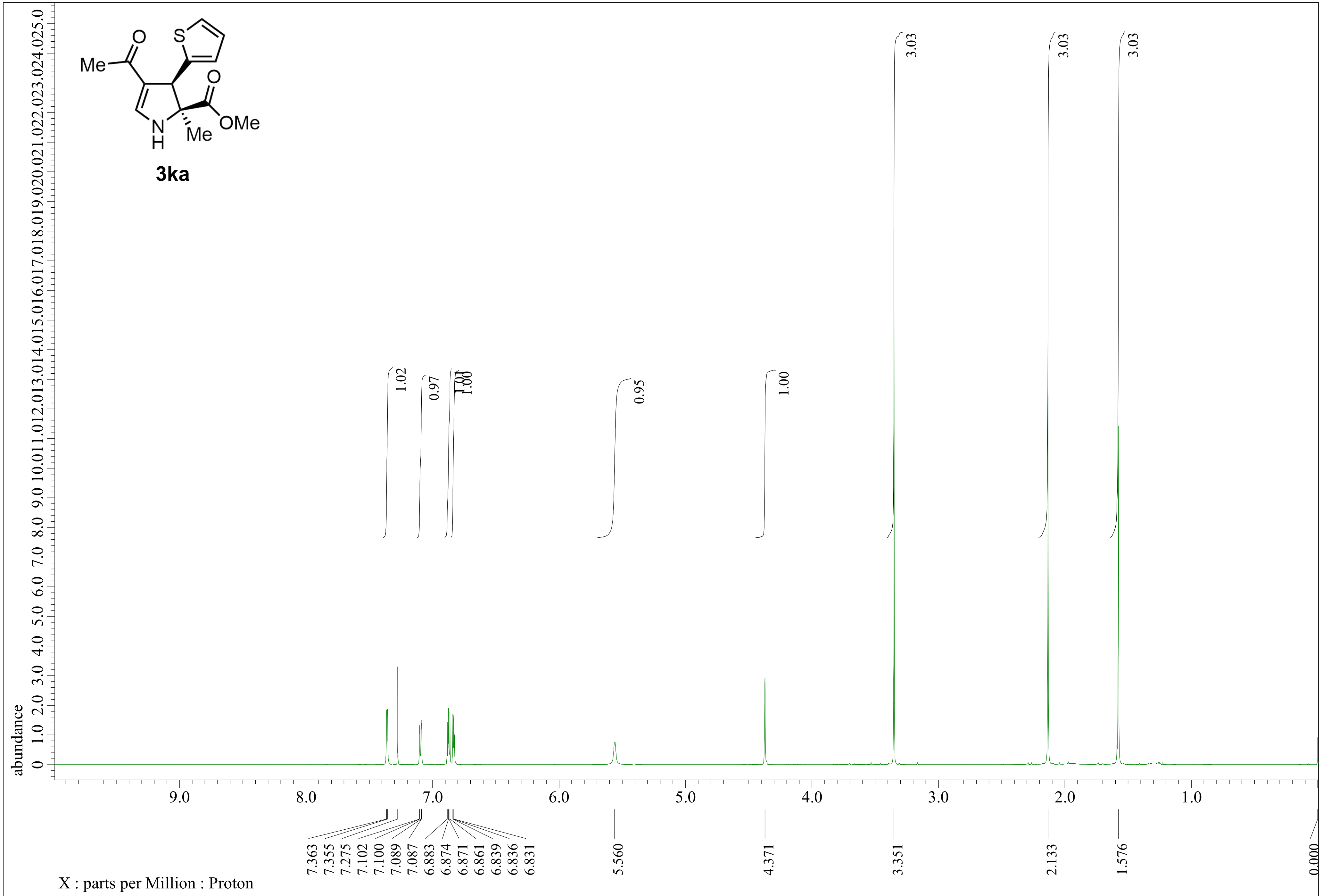
Me
O
S
O
OMe
N
H
Me
3ka
3.03
3.03
3.03
1.02
0.97
1.01
1.00
0.95
1.00
abundance
9.0
8.0
7.0
6.0
5.0
4.0
3.0
2.0
1.0
7.363
7.355
7.275
7.102
7.100
7.089
7.087
6.883
6.874
6.871
6.861
6.839
6.836
6.831
5.560
4.371
3.351
2.133
1.576
0.000
X : parts per Million : Proton

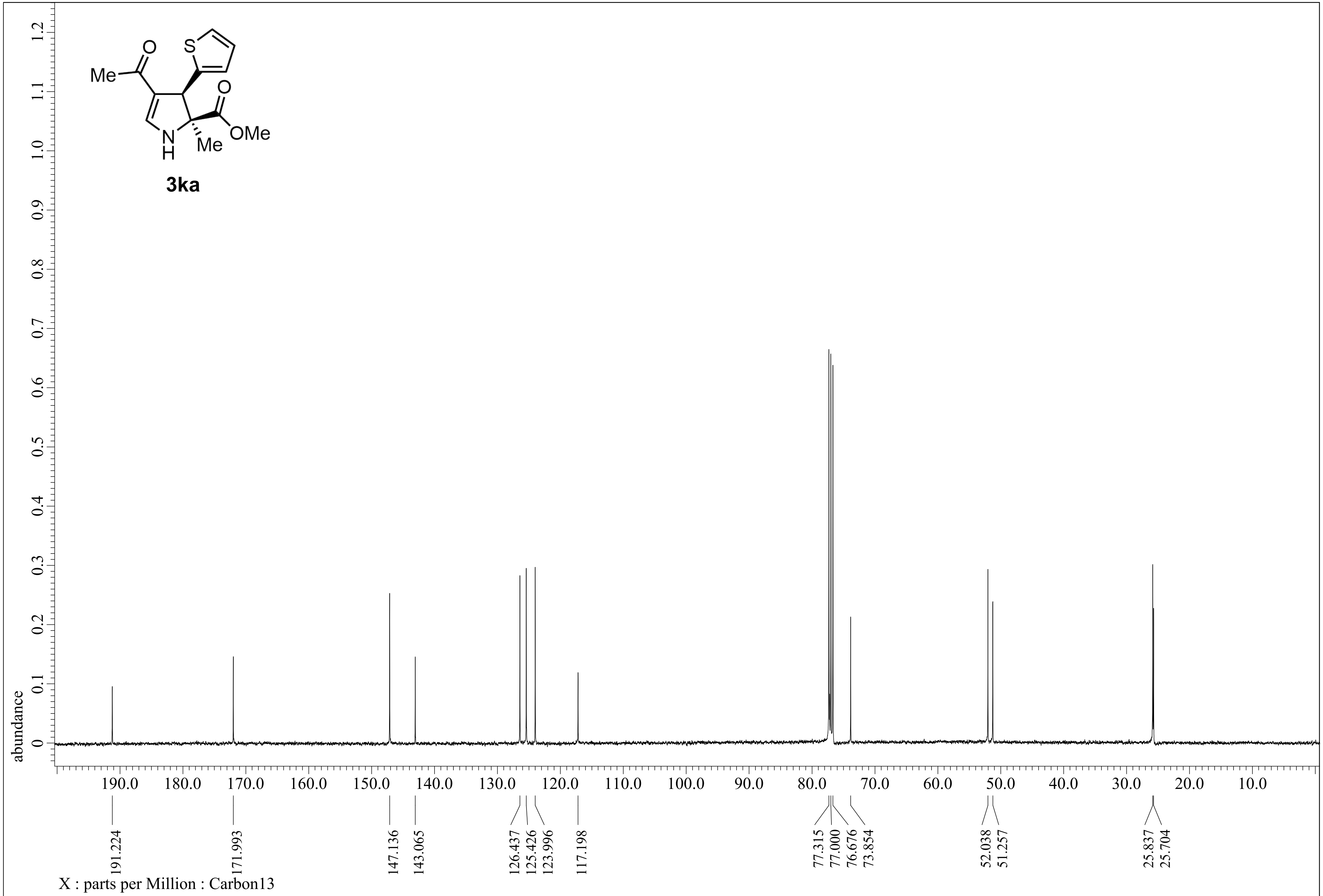
S
O
Me
O
OMe
N
H
Me
3ka
abundance
0
0.1
0.2
0.3
0.4
0.5
0.6
0.7
0.8
0.9
1.0
1.1
1.2
190.0
180.0
170.0
160.0
150.0
140.0
130.0
120.0
110.0
100.0
90.0
80.0
70.0
60.0
50.0
40.0
30.0
20.0
10.0
191.224
171.993
147.136
143.065
126.437
125.426
123.996
117.198
77.315
77.000
76.676
73.854
52.038
51.257
25.837
25.704
X : parts per Million : Carbon13

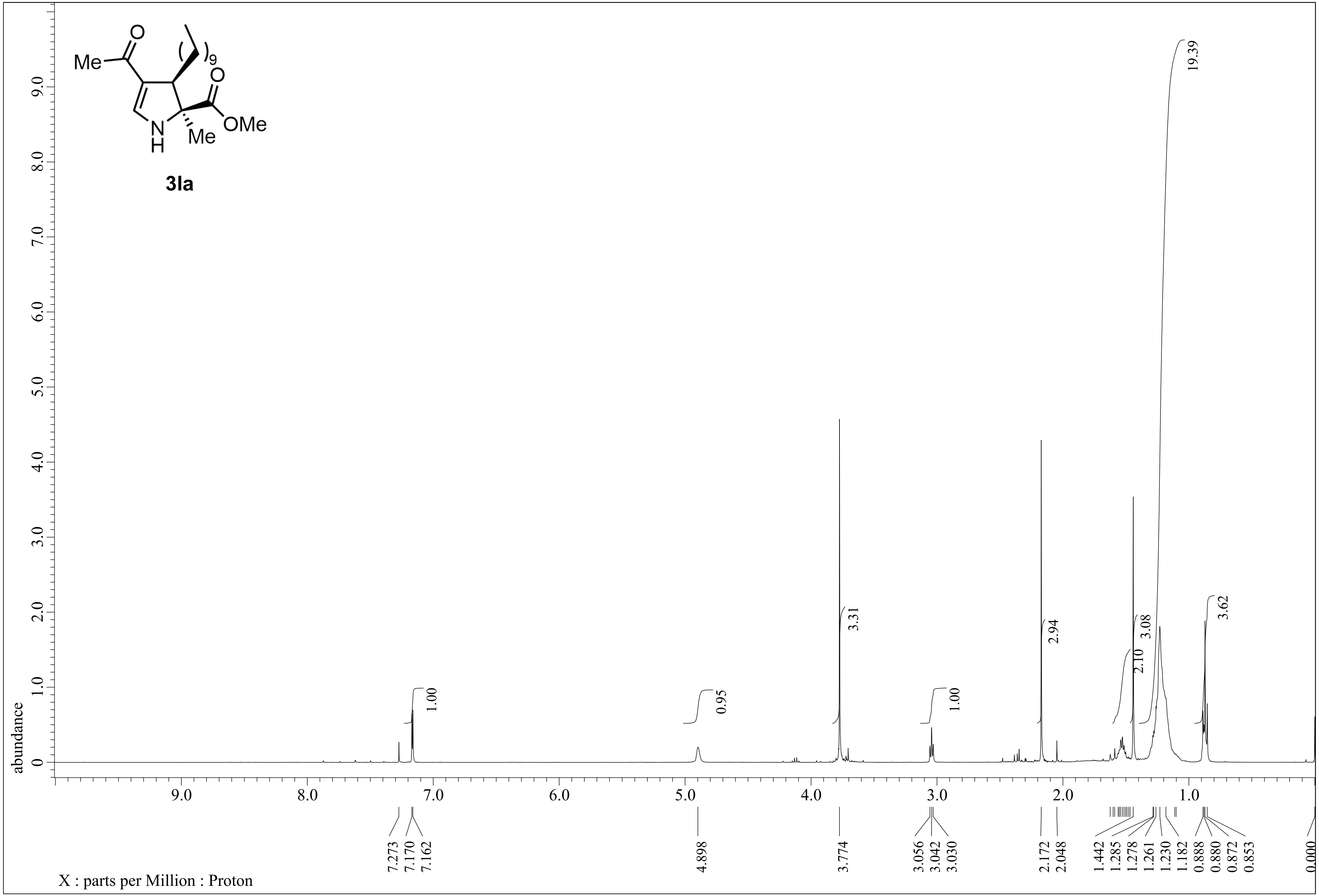
Me
O
O
OMe
N
H
Me
9
9.0
8.0
7.0
6.0
5.0
4.0
3.0
2.0
1.0
0
abundance
1.00
0.95
3.31
1.00
2.94
2.10
3.08
19.39
3.62
7.273
7.170
7.162
4.898
3.774
3.056
3.042
3.030
2.172
2.048
1.442
1.285
1.278
1.261
1.230
1.182
0.888
0.880
0.872
0.853
0.000
X : parts per Million : Proton

3la

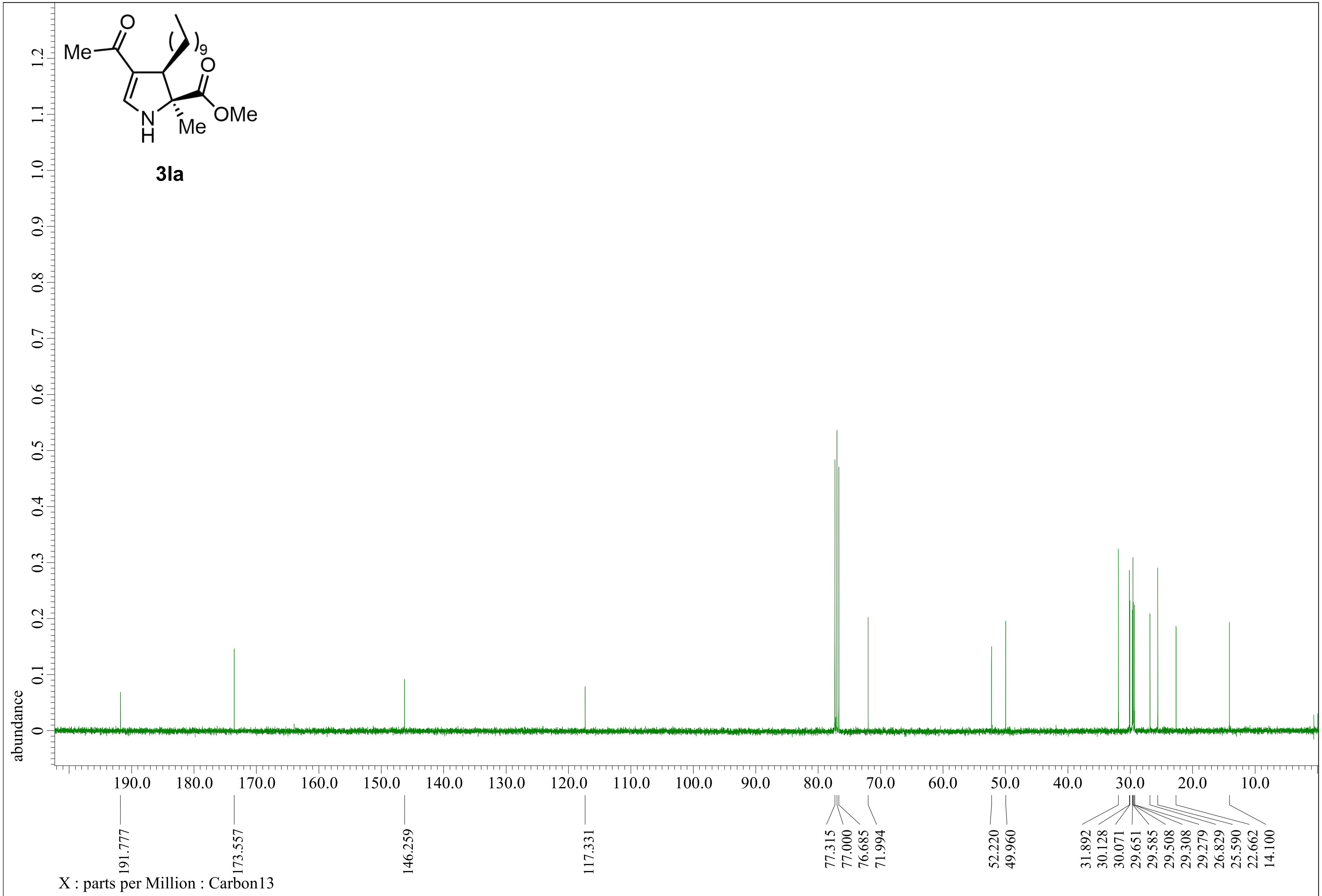
Me
O
( )9
O
OMe
N
H
Me
3la
abundance
1.2
1.1
1.0
0.9
0.8
0.7
0.6
0.5
0.4
0.3
0.2
0.1
0
190.0 180.0 170.0 160.0 150.0 140.0 130.0 120.0 110.0 100.0 90.0 80.0 70.0 60.0 50.0 40.0 30.0 20.0 10.0
191.777
173.557
146.259
117.331
77.315
77.000
76.685
71.994
52.220
49.960
31.892
30.128
30.071
29.651
29.585
29.508
29.308
29.279
26.829
25.590
22.662
14.100
X : parts per Million : Carbon13

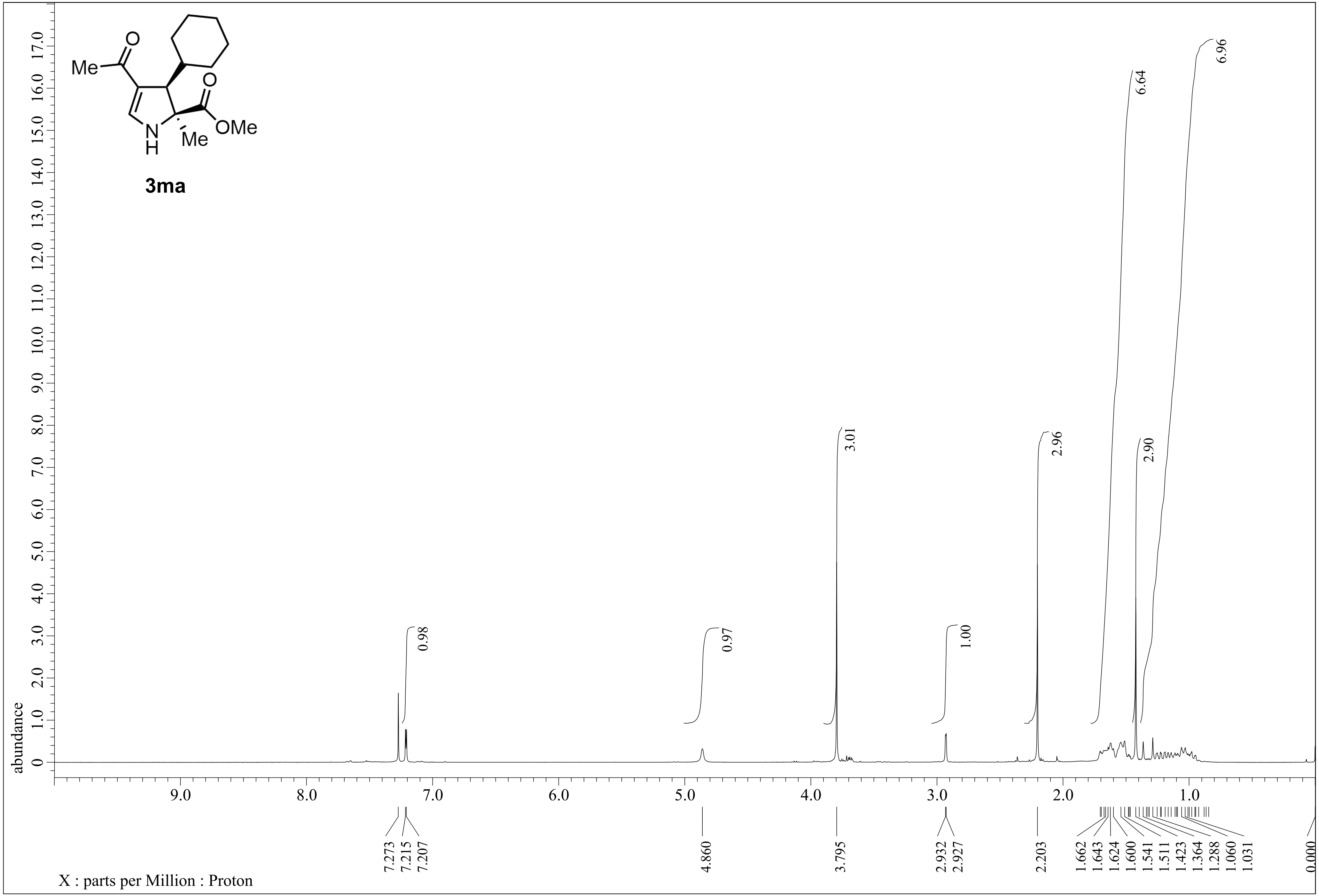

3ma
abundance
X : parts per Million : Proton

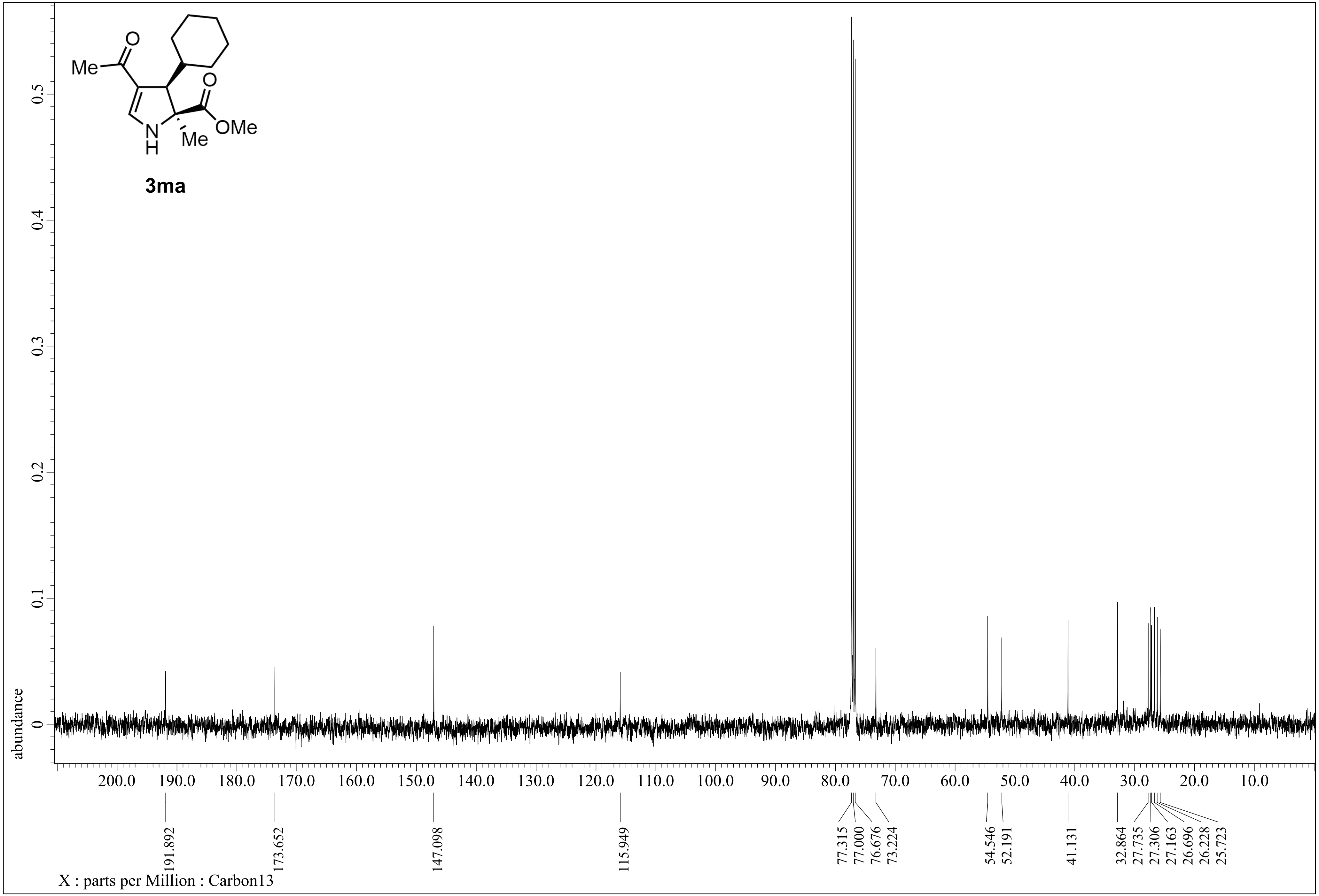
Me
O
O
OMe
N
H
Me
3ma
0.5
0.4
0.3
0.2
0.1
0
abundance
200.0 190.0 180.0 170.0 160.0 150.0 140.0 130.0 120.0 110.0 100.0 90.0 80.0 70.0 60.0 50.0 40.0 30.0 20.0 10.0
191.892
173.652
147.098
115.949
77.315
77.000
76.676
73.224
54.546
52.191
41.131
32.864
27.735
27.306
27.163
26.696
26.228
25.723
X : parts per Million : Carbon13

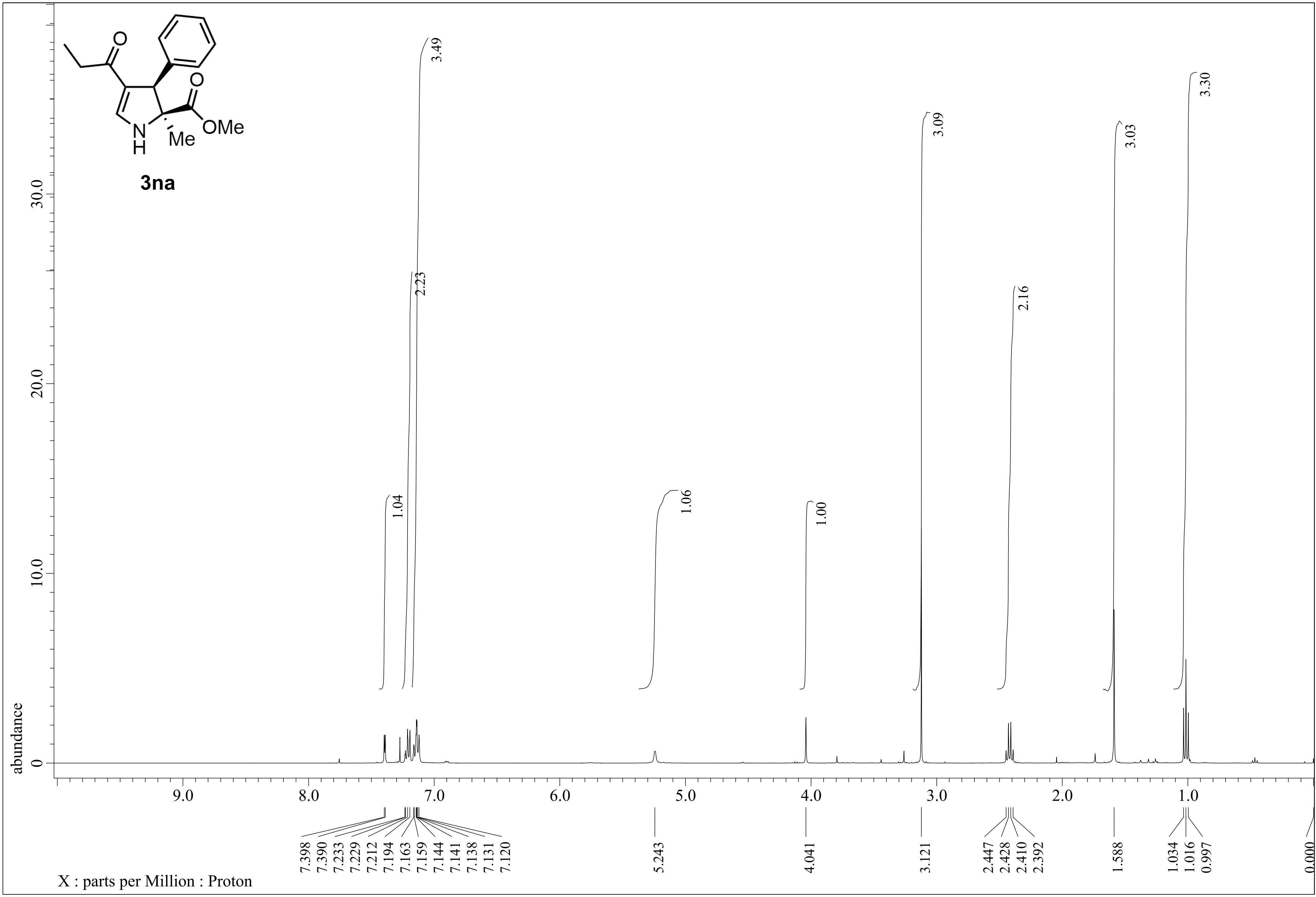

3na
abundance
X : parts per Million : Proton
30.0
20.0
10.0
0
9.0
8.0
7.0
6.0
5.0
4.0
3.0
2.0
1.0
1.04
2.23
3.49
1.06
1.00
3.09
2.16
3.03
3.30
7.398
7.390
7.233
7.229
7.212
7.194
7.163
7.159
7.144
7.141
7.138
7.131
7.120
5.243
4.041
3.121
2.447
2.428
2.410
2.392
1.588
1.034
1.016
0.997
0.000

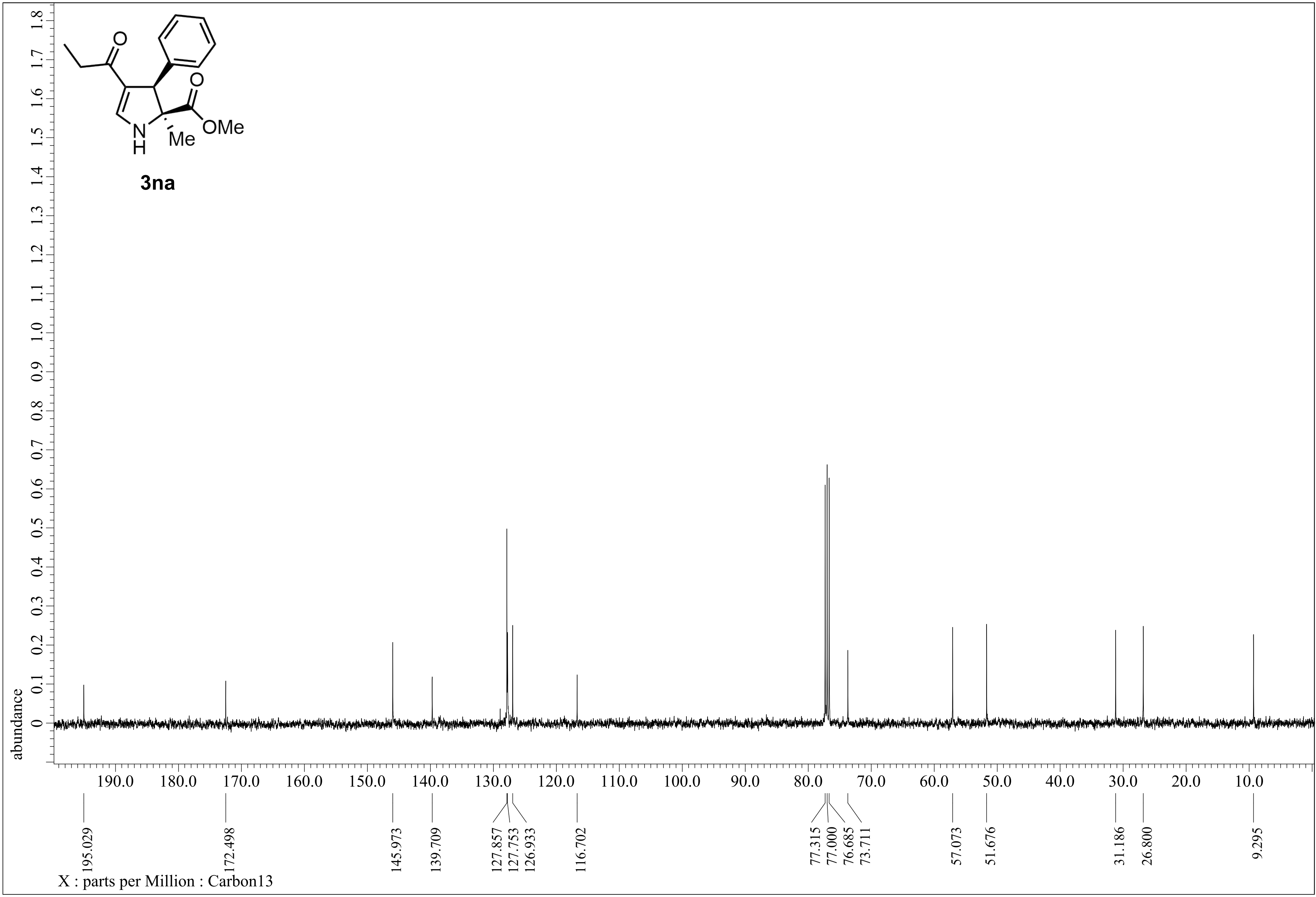
O
O
OMe
N
H
Me
3na
abundance
195.029
172.498
145.973
139.709
127.857
127.753
126.933
116.702
77.315
77.000
76.685
73.711
57.073
51.676
31.186
26.800
9.295
X : parts per Million : Carbon13

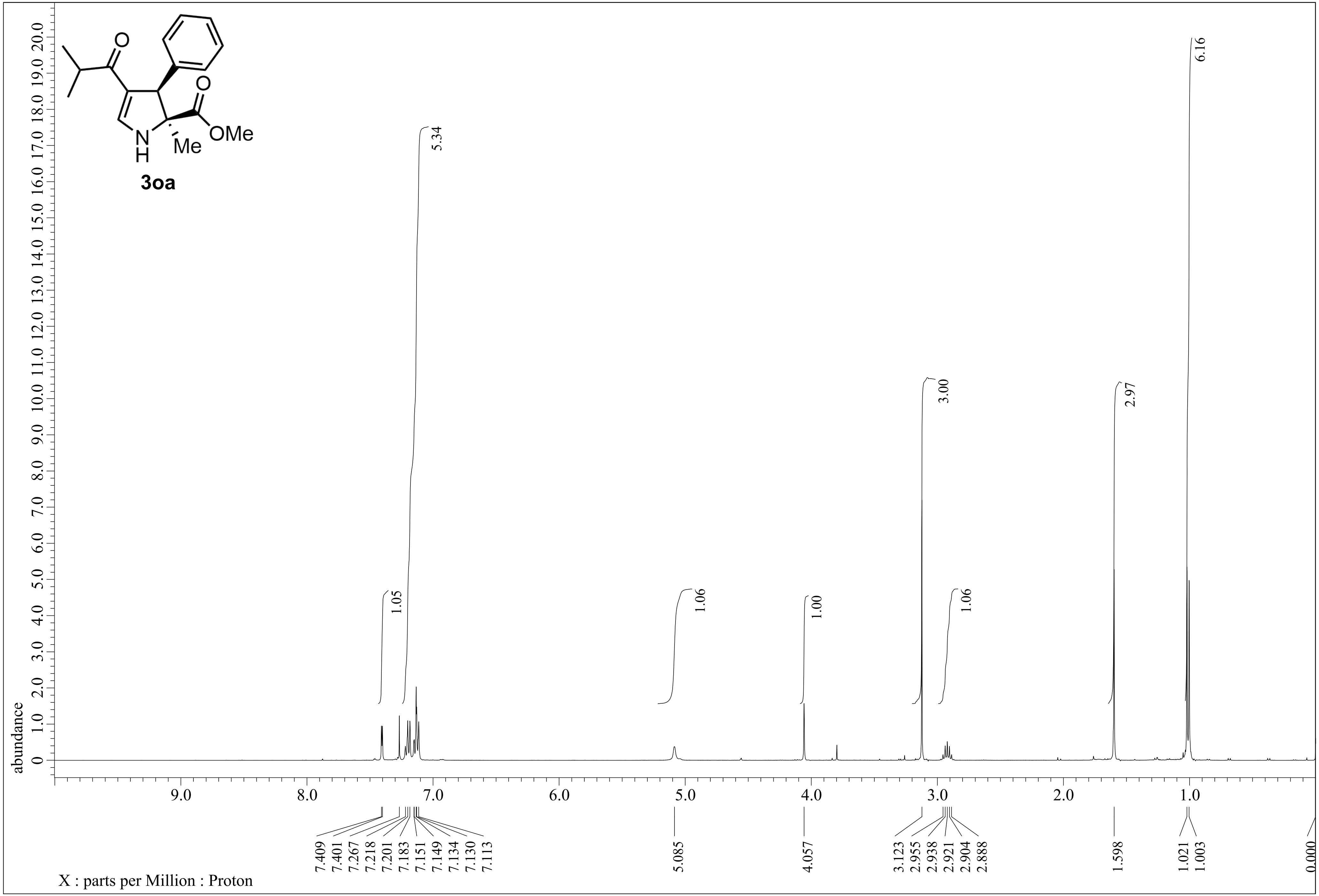
3oa
abundance
X : parts per Million : Proton
5.34
1.05
1.06
1.00
3.00
1.06
2.97
6.16
7.409
7.401
7.267
7.218
7.201
7.183
7.151
7.149
7.134
7.130
7.113
5.085
4.057
3.123
2.955
2.938
2.921
2.904
2.888
1.598
1.021
1.003
0.000

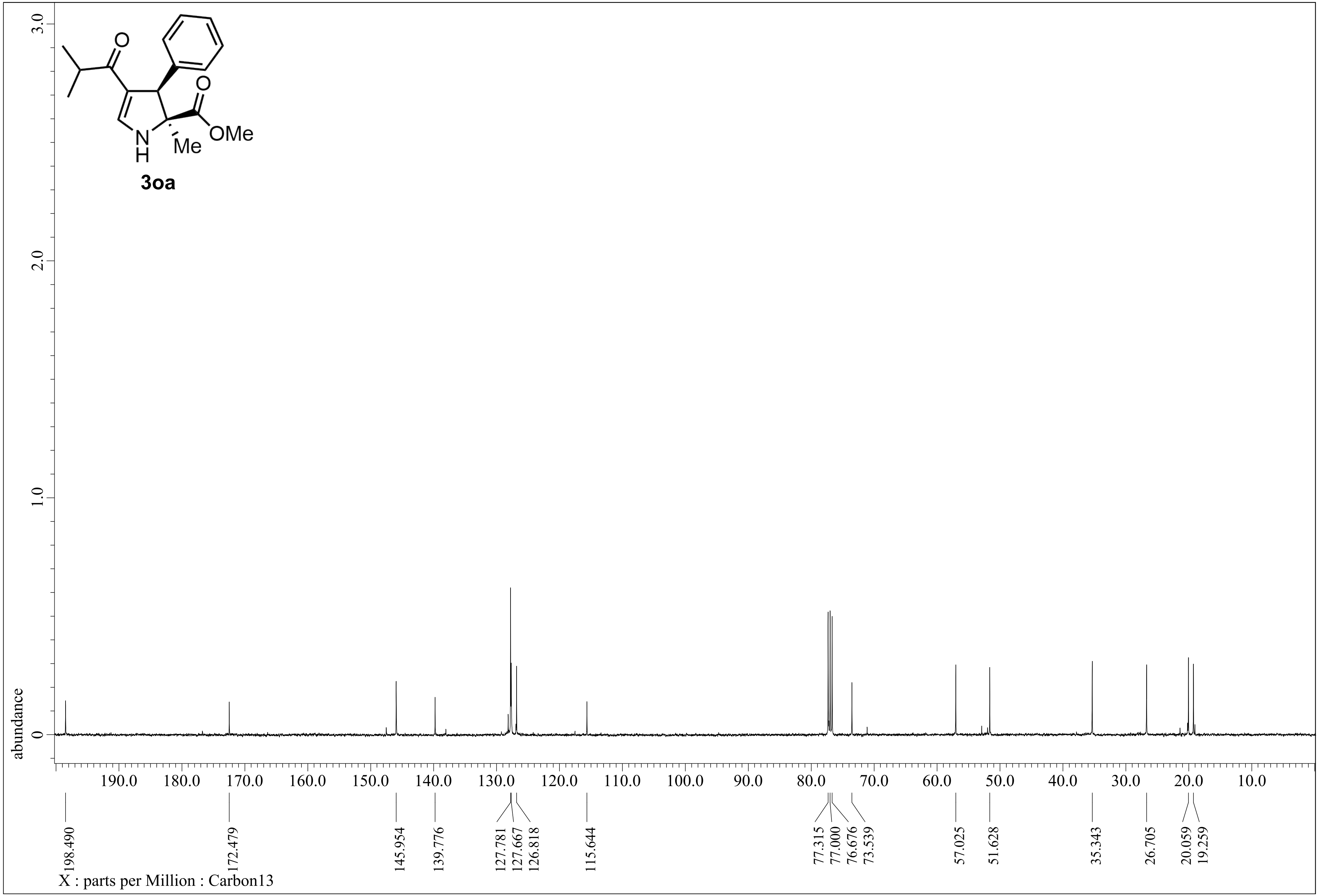

O
O
OMe
N
H
Me
3oa
3.0
2.0
1.0
0
abundance
190.0 180.0 170.0 160.0 150.0 140.0 130.0 120.0 110.0 100.0 90.0 80.0 70.0 60.0 50.0 40.0 30.0 20.0 10.0
198.490
172.479
145.954
139.776
127.781
127.667
126.818
115.644
77.315
77.000
76.676
73.539
57.025
51.628
35.343
26.705
20.059
19.259
X : parts per Million : Carbon13

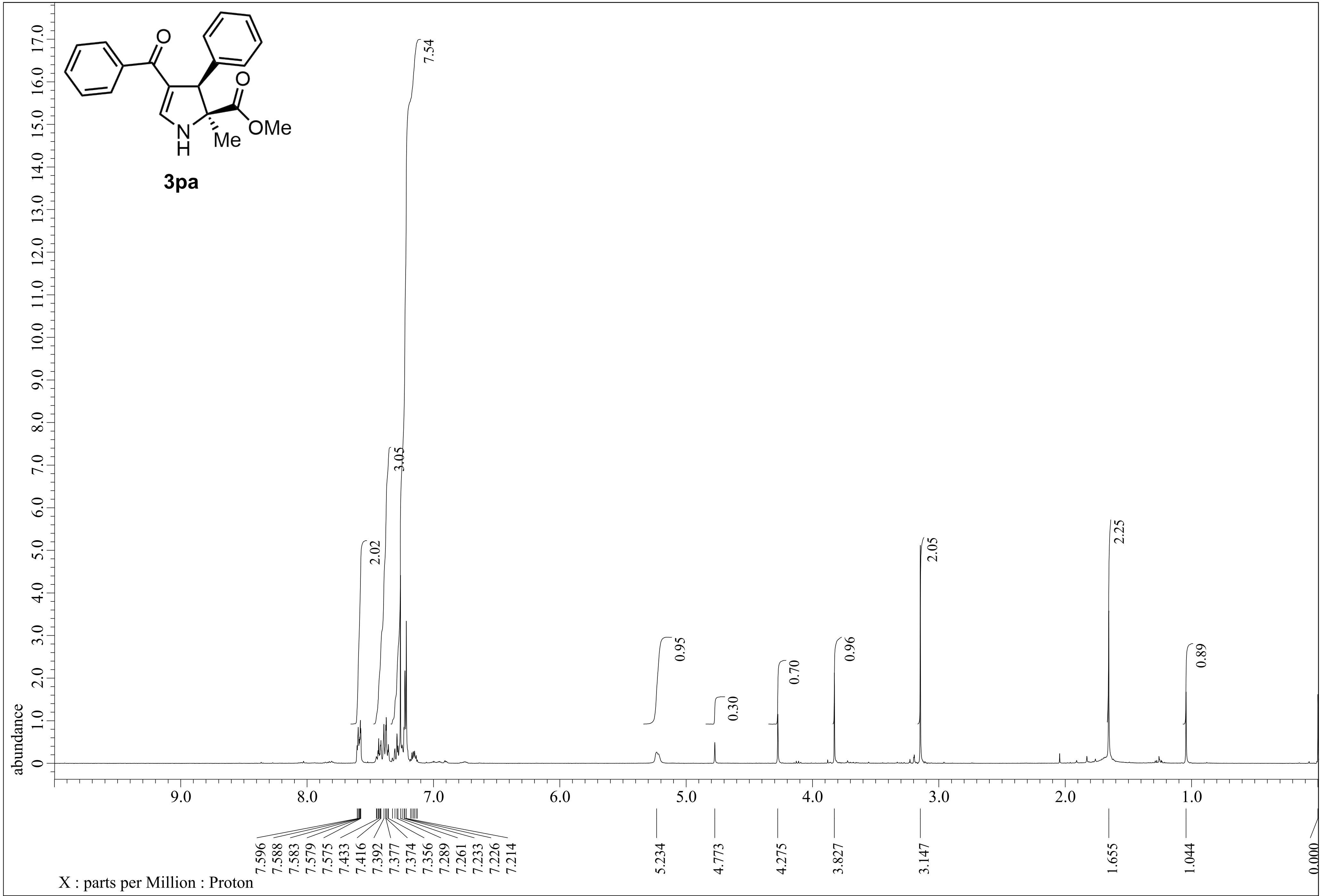
O
O
OMe
N
H
Me
3pa
abundance
0
1.0
2.0
3.0
4.0
5.0
6.0
7.0
8.0
9.0
10.0
11.0
12.0
13.0
14.0
15.0
16.0
17.0
7.54
3.05
2.02
0.95
0.30
0.70
0.96
2.05
2.25
0.89
9.0
8.0
7.0
6.0
5.0
4.0
3.0
2.0
1.0
7.596
7.588
7.583
7.579
7.575
7.433
7.416
7.392
7.377
7.374
7.356
7.289
7.261
7.233
7.226
7.214
5.234
4.773
4.275
3.827
3.147
1.655
1.044
0.000
X : parts per Million : Proton

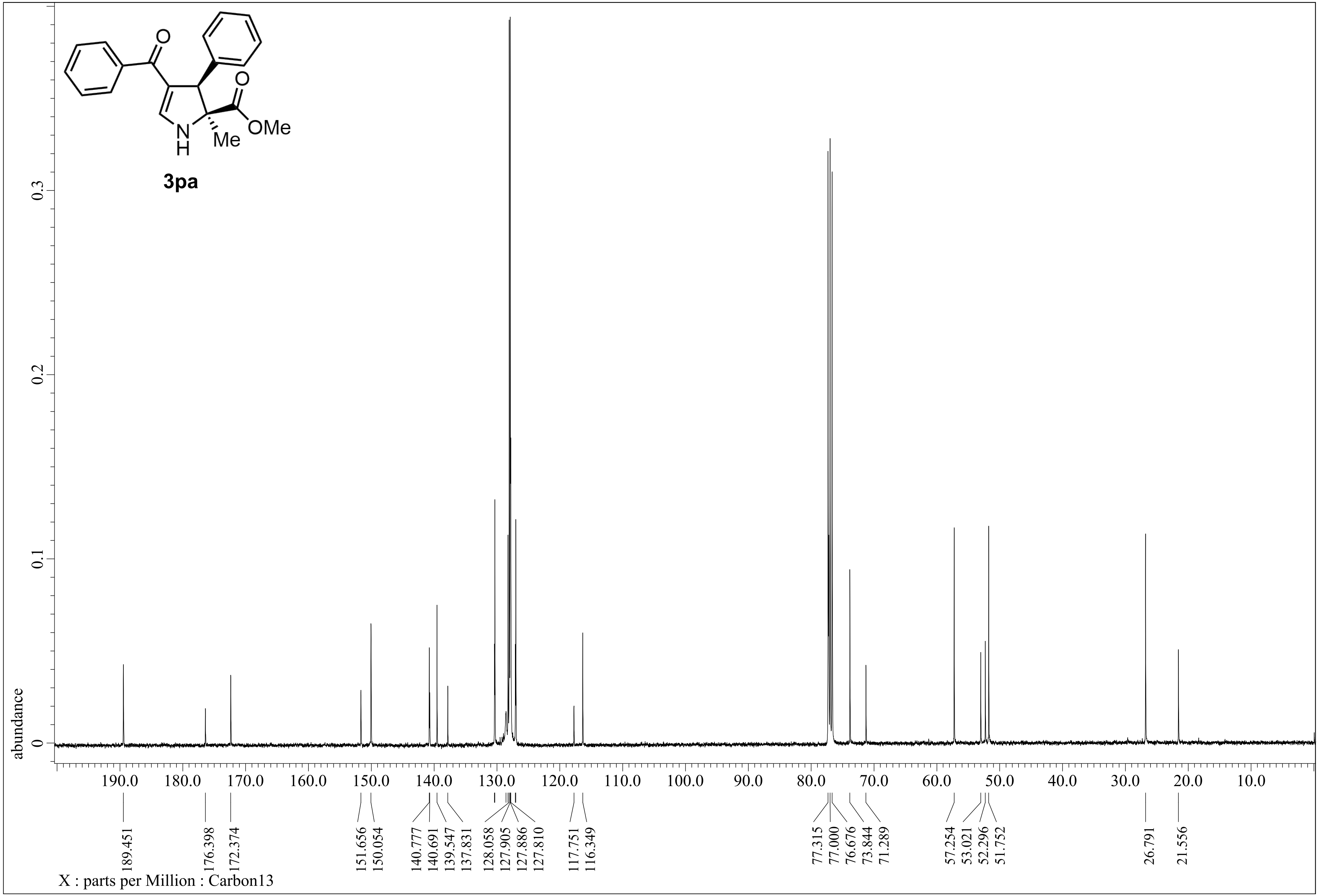
O
O
N
H
Me
OMe
3pa
abundance
0.3
0.2
0.1
0
190.0 180.0 170.0 160.0 150.0 140.0 130.0 120.0 110.0 100.0 90.0 80.0 70.0 60.0 50.0 40.0 30.0 20.0 10.0
189.451
176.398
172.374
151.656
150.054
140.777
140.691
139.547
137.831
128.058
127.905
127.886
127.810
117.751
116.349
77.315
77.000
76.676
73.844
71.289
57.254
53.021
52.296
51.752
26.791
21.556
X : parts per Million : Carbon13

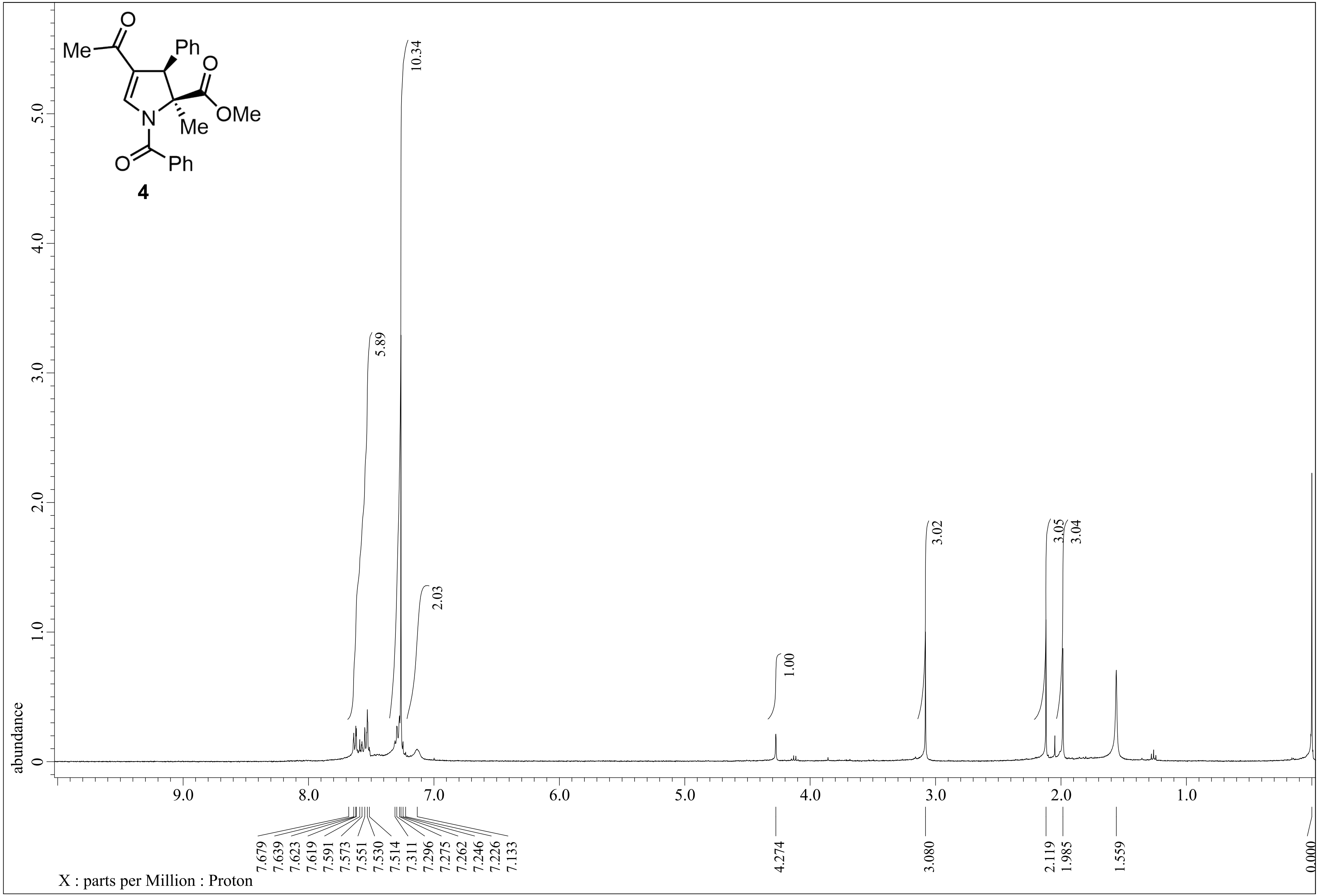

Me
Ph
O
OMe
Me
N
O
Ph
4
10.34
5.89
2.03
1.00
3.02
3.05
3.04
abundance
9.0
8.0
7.0
6.0
5.0
4.0
3.0
2.0
1.0
7.679
7.639
7.623
7.619
7.591
7.573
7.551
7.530
7.514
7.311
7.296
7.275
7.262
7.246
7.226
7.133
4.274
3.080
2.119
1.985
1.559
0.000
X : parts per Million : Proton

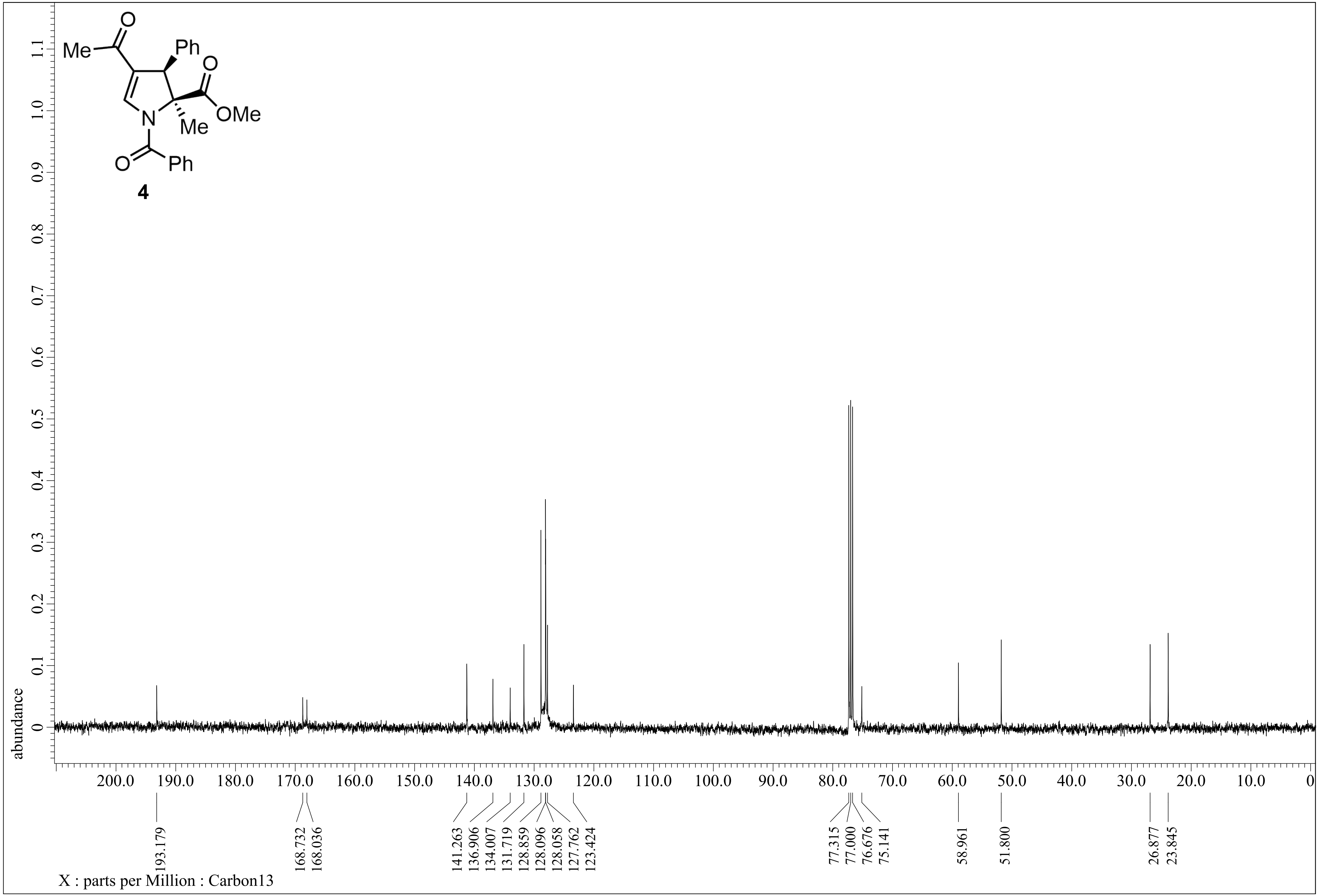

4
abundance
X : parts per Million : Carbon13
193.179
168.732
168.036
141.263
136.906
134.007
131.719
128.859
128.096
128.058
127.762
123.424
77.315
77.000
76.676
75.141
58.961
51.800
26.877
23.845

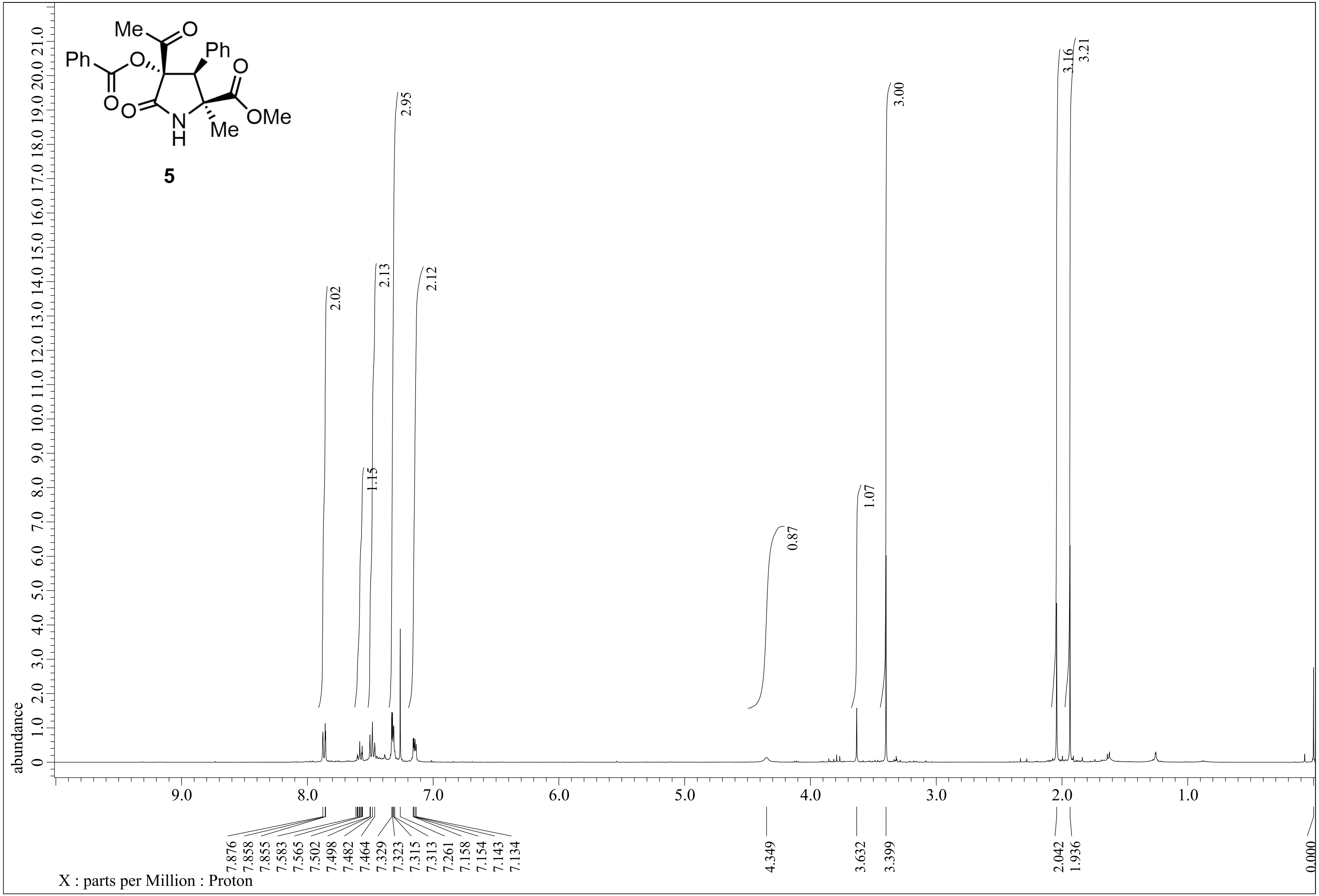
Me
Ph
Ph
O
O
O
O
N
H
Me
OMe
5
abundance
2.02
1.15
2.13
2.95
2.12
0.87
1.07
3.00
3.16
3.21
7.876
7.858
7.855
7.583
7.565
7.502
7.498
7.482
7.464
7.329
7.323
7.315
7.313
7.261
7.158
7.154
7.143
7.134
4.349
3.632
3.399
2.042
1.936
0.000
X : parts per Million : Proton

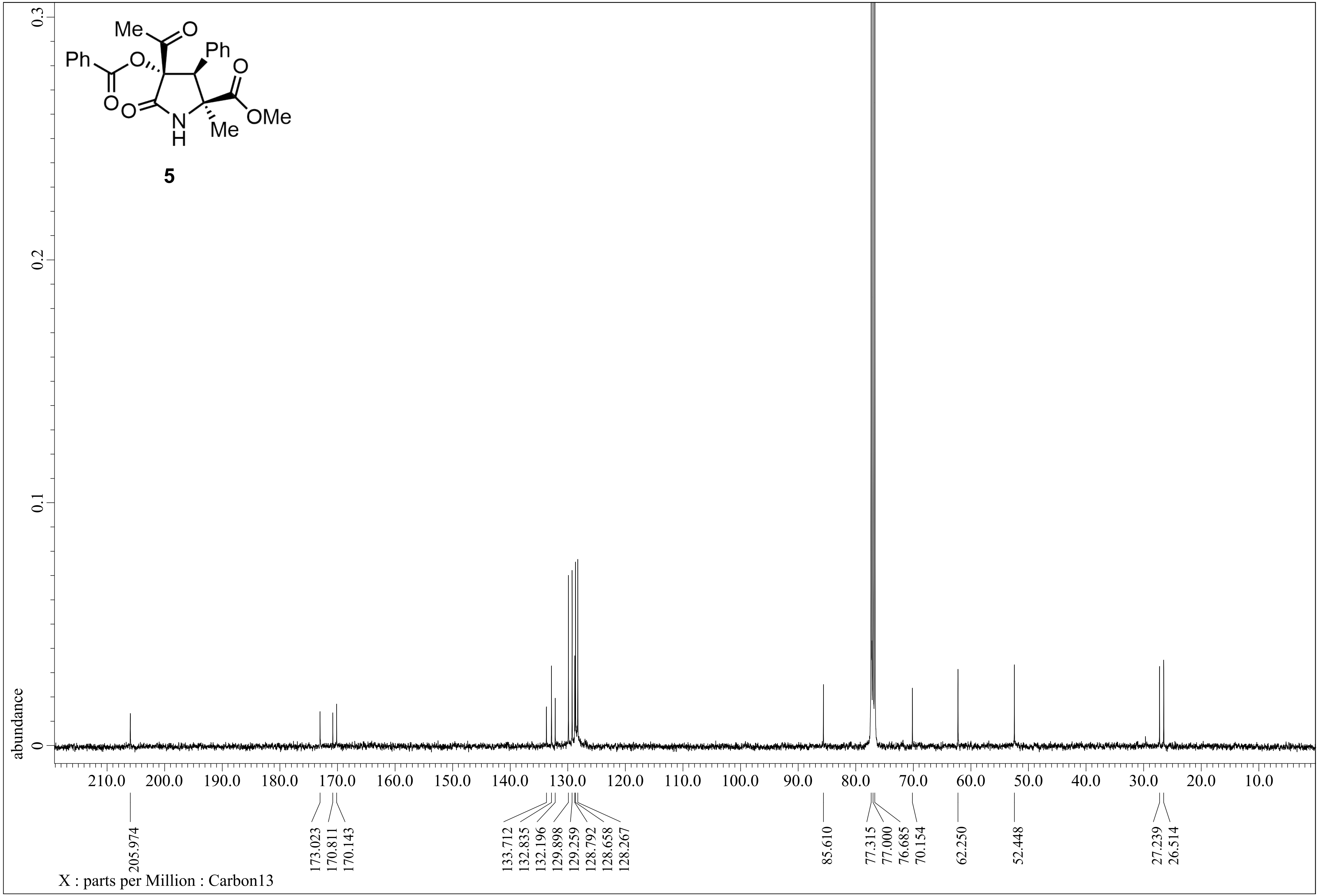
Me
O
Ph
Ph
O
O
O
O
N
H
Me
OMe
5
abundance
0.3
0.2
0.1
0
210.0 200.0 190.0 180.0 170.0 160.0 150.0 140.0 130.0 120.0 110.0 100.0 90.0 80.0 70.0 60.0 50.0 40.0 30.0 20.0 10.0
205.974
173.023
170.811
170.143
133.712
132.835
132.196
129.898
129.259
128.792
128.658
128.267
85.610
77.315
77.000
76.685
70.154
62.250
52.448
27.239
26.514
X : parts per Million : Carbon13

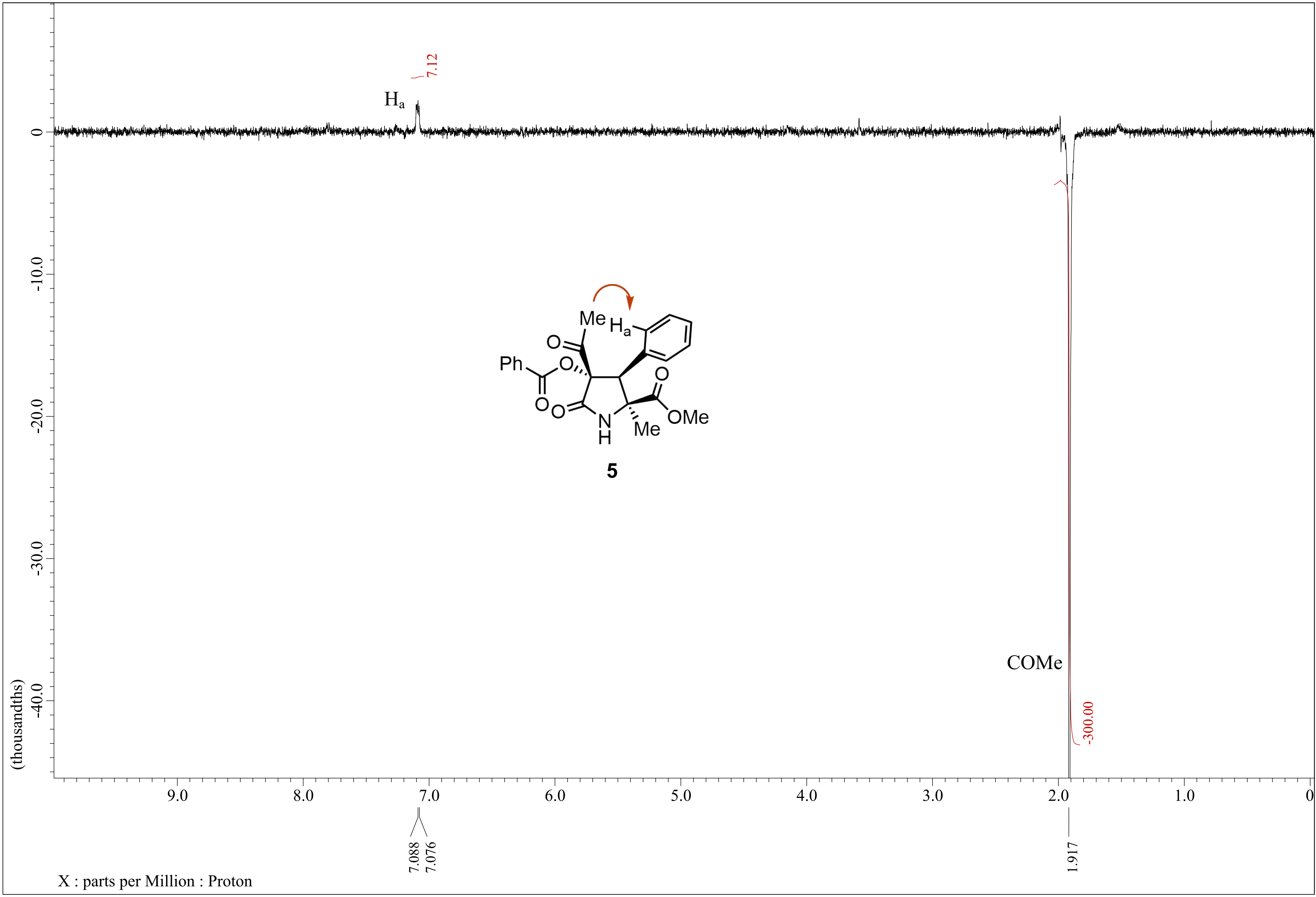
7.12
Ha
0
-10.0
-20.0
-30.0
-40.0
(thousandths)
Me
Ha
O
Ph
O
O
O
N
H
Me
O
OMe
5
COMe
-300.00
9.0
8.0
7.0
6.0
5.0
4.0
3.0
2.0
1.0
0
7.088
7.076
1.917
X : parts per Million : Proton

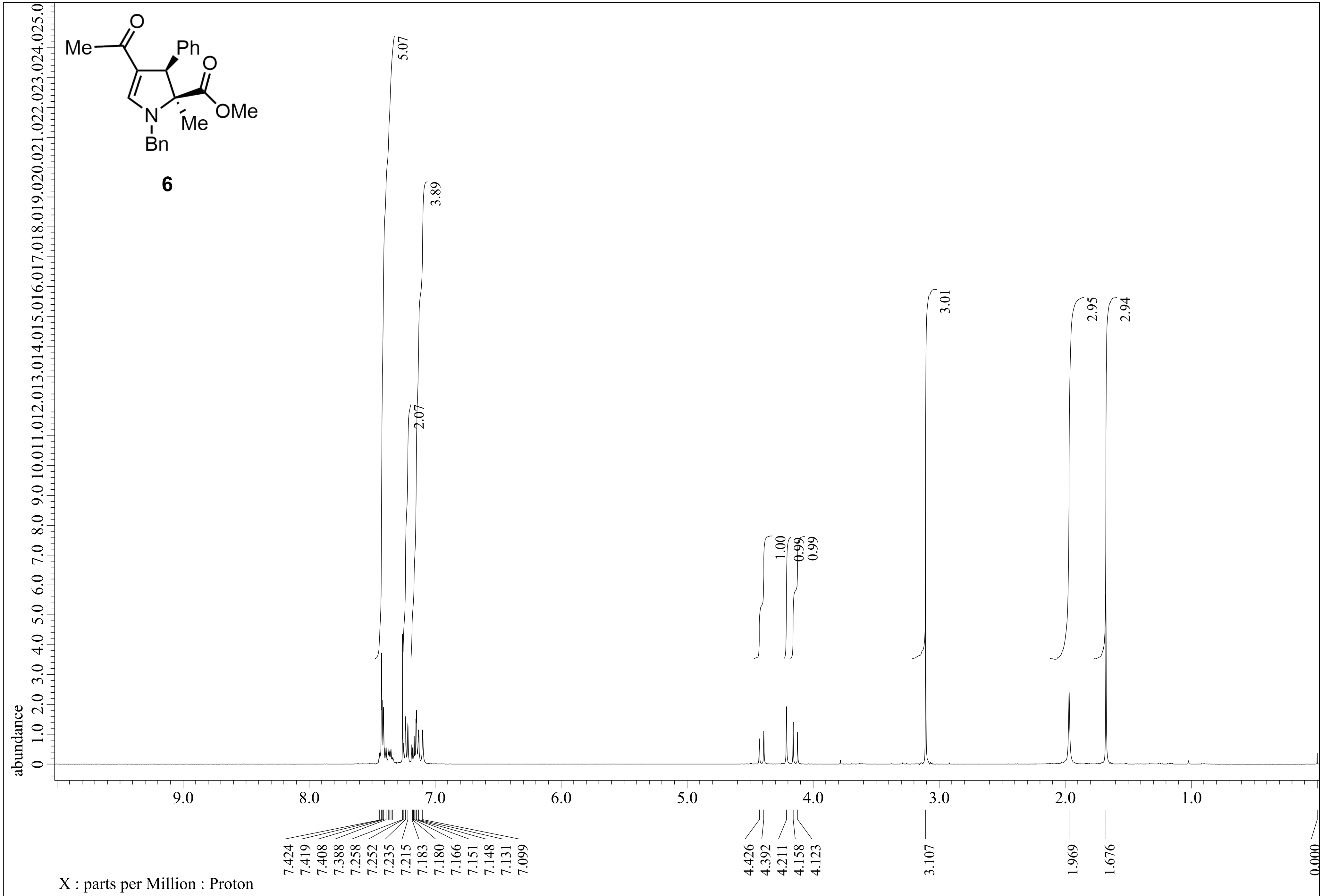
Me
Ph
O
O
N
OMe
Me
Bn
abundance
X : parts per Million : Proton
5.07
3.89
2.07
1.00
0.99
0.99
3.01
2.95
2.94
7.424
7.419
7.408
7.388
7.258
7.252
7.235
7.215
7.183
7.180
7.166
7.151
7.148
7.131
7.099
4.426
4.392
4.211
4.158
4.123
3.107
1.969
1.676
0.000

6

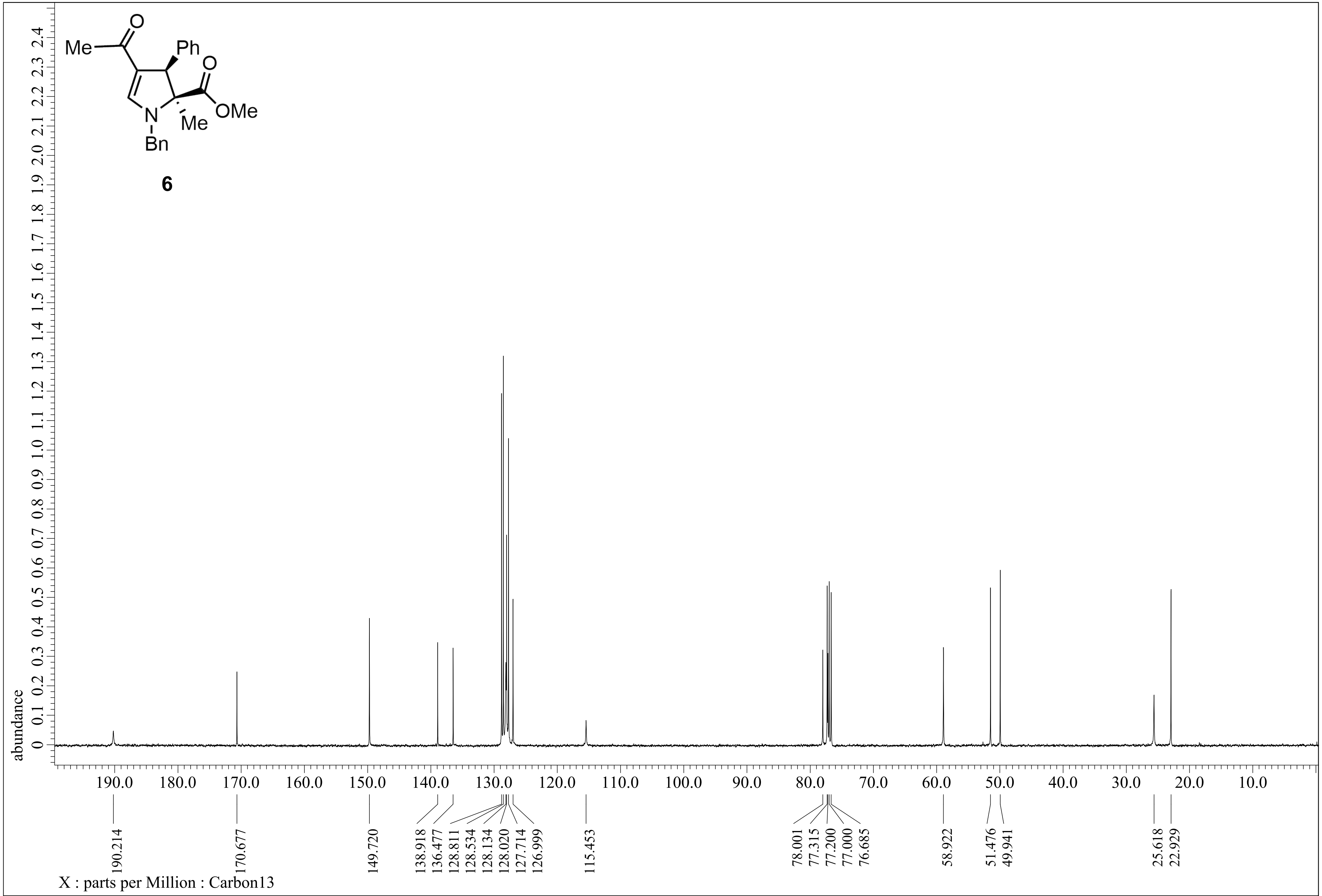
Me
O
Ph
O
OMe
N
Me
Bn
6
abundance
X : parts per Million : Carbon13
190.214
170.677
149.720
138.918
136.477
128.811
128.534
128.134
128.020
127.714
126.999
115.453
78.001
77.315
77.200
77.000
76.685
58.922
51.476
49.941
25.618
22.929

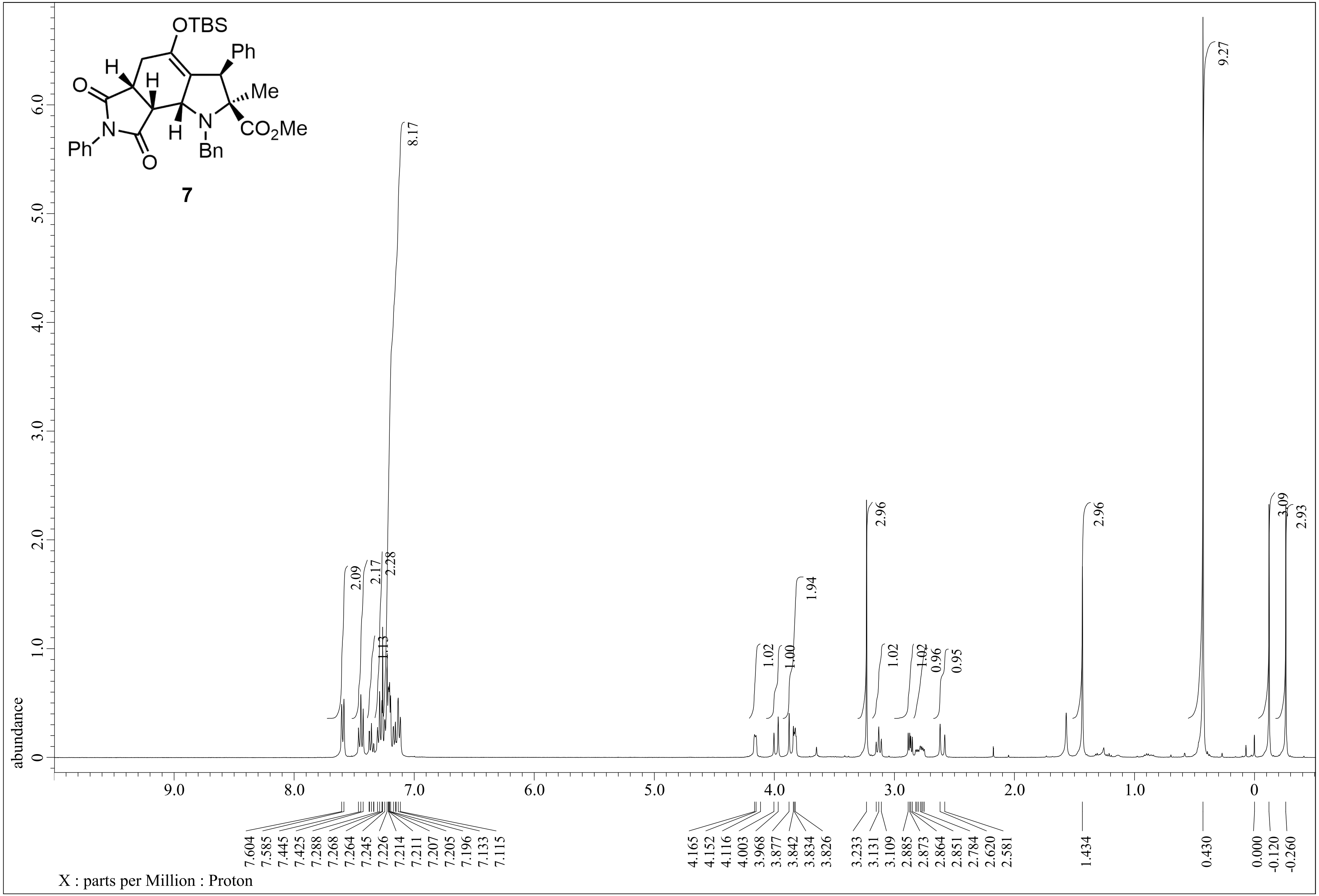
OTBS
Ph
Me
CO2Me
Bn
7
X : parts per Million : Proton
abundance

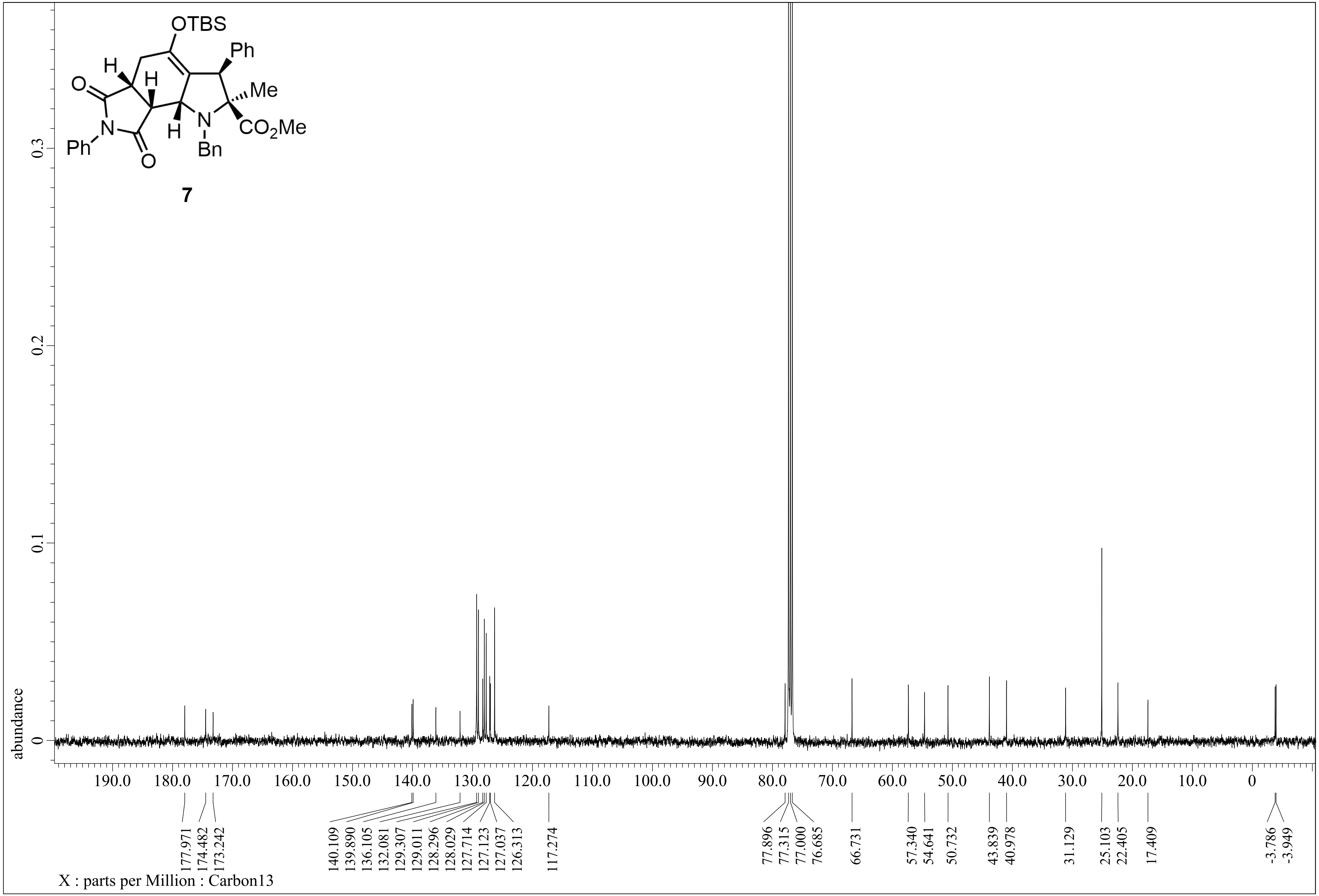
OTBS
Ph
H
H
O
Me
N
CO2Me
Ph
H
N
Bn
O
7
abundance
0
0.1
0.2
0.3
X : parts per Million : Carbon13
190.0 180.0 170.0 160.0 150.0 140.0 130.0 120.0 110.0 100.0 90.0 80.0 70.0 60.0 50.0 40.0 30.0 20.0 10.0 0
177.971
174.482
173.242
140.109
139.890
136.105
132.081
129.307
129.011
128.296
128.029
127.714
127.123
127.037
126.313
117.274
77.896
77.315
77.000
76.685
66.731
57.340
54.641
50.732
43.839
40.978
31.129
25.103
22.405
17.409
-3.786
-3.949

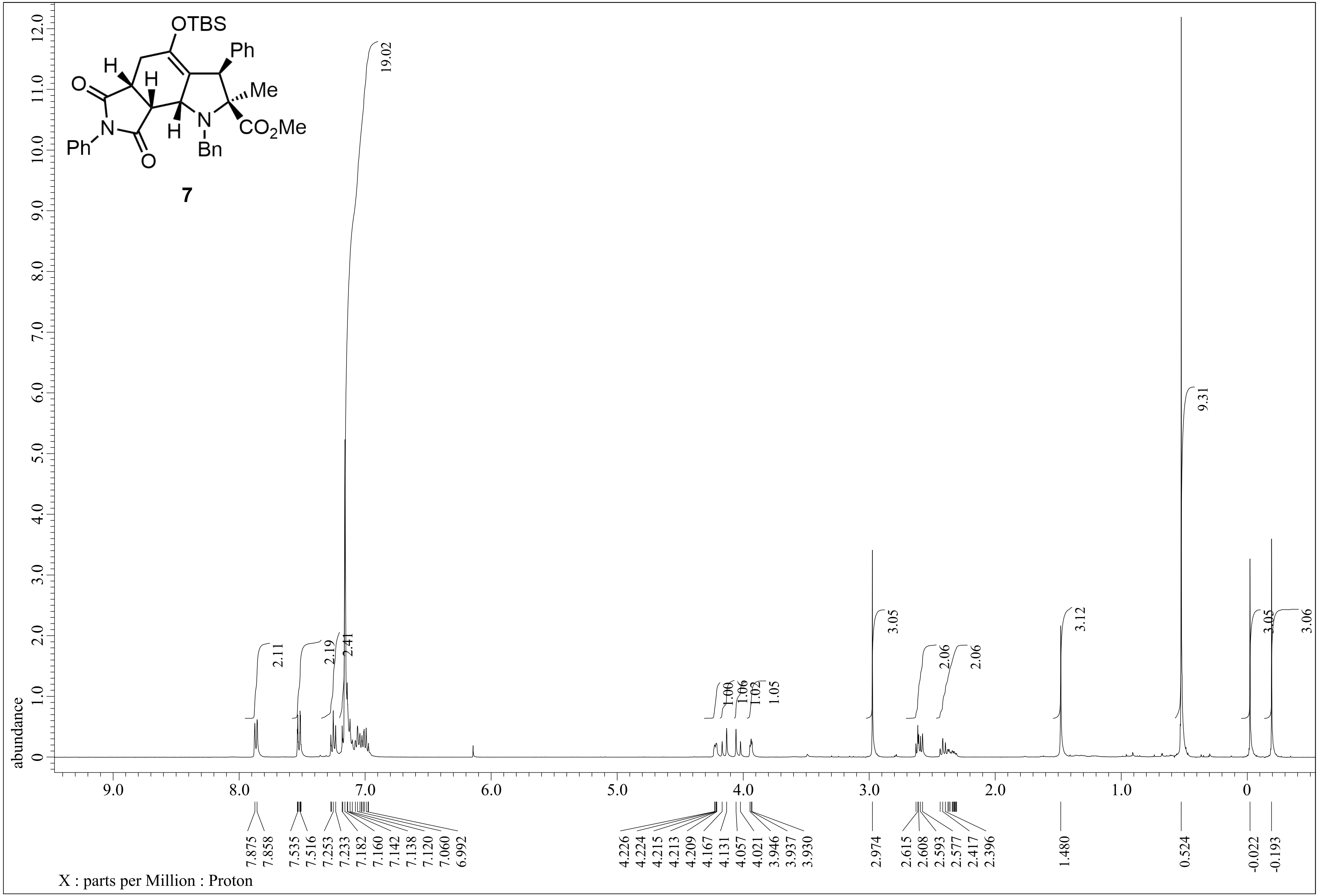
OTBS
Ph
H
H
Me
O
N
N
CO2Me
Ph
H
Bn
O
7
abundance
X : parts per Million : Proton
19.02
9.31
3.05
3.12
3.05
3.06
2.06
2.06
2.11
2.19
2.41
1.00
1.06
1.02
1.05
7.875
7.858
7.535
7.516
7.253
7.233
7.182
7.160
7.142
7.138
7.120
7.060
6.992
4.226
4.224
4.215
4.213
4.209
4.167
4.131
4.057
4.021
3.946
3.937
3.930
2.974
2.615
2.608
2.593
2.577
2.417
2.396
1.480
0.524
-0.022
-0.193

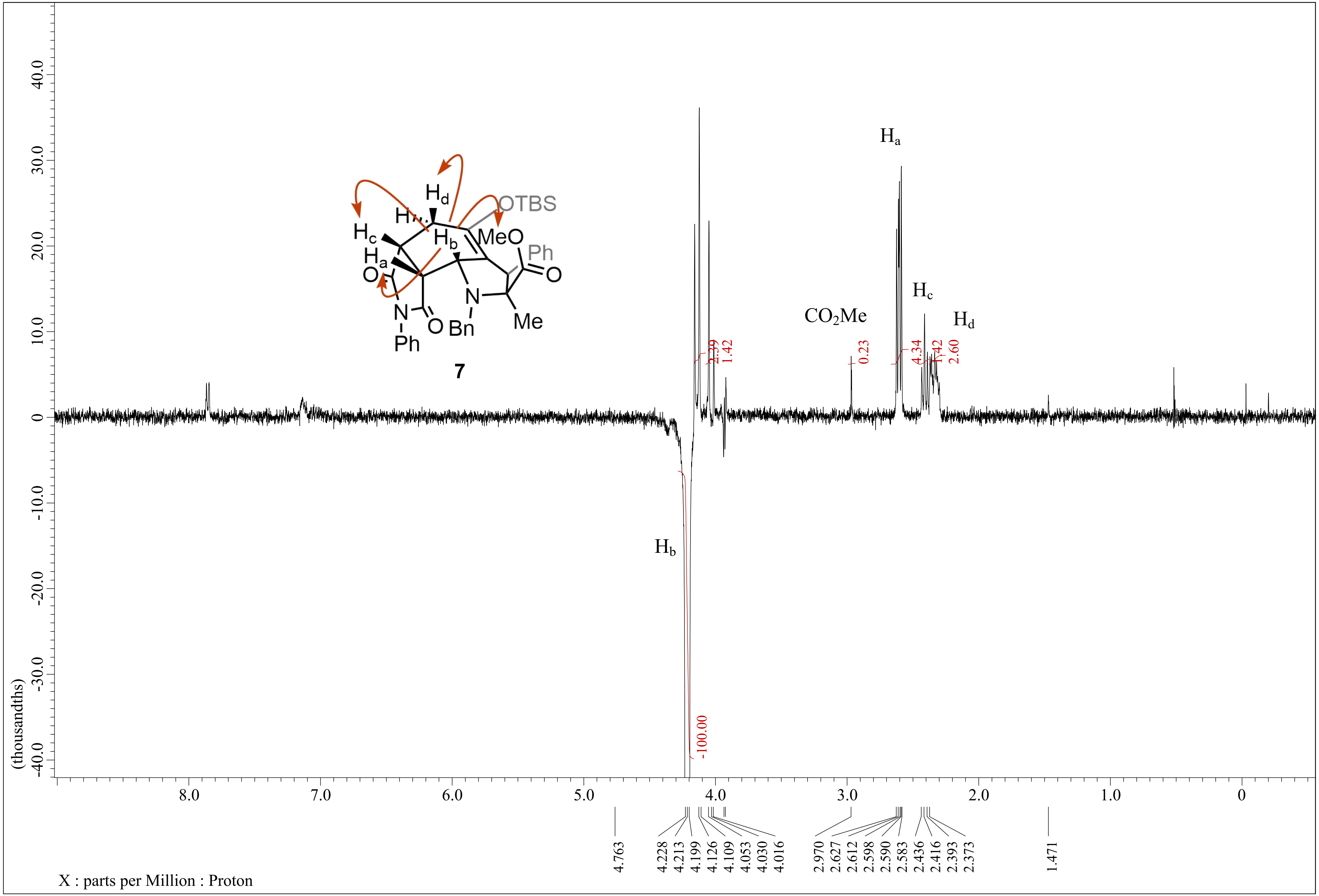

OTBS
MeO
Ph
O
H d
H
H c
H b
H a
O
N
O
Ph
N
Bn
Me
7
H a
H c
H d
CO2Me
H b
X : parts per Million : Proton
(thousandths)

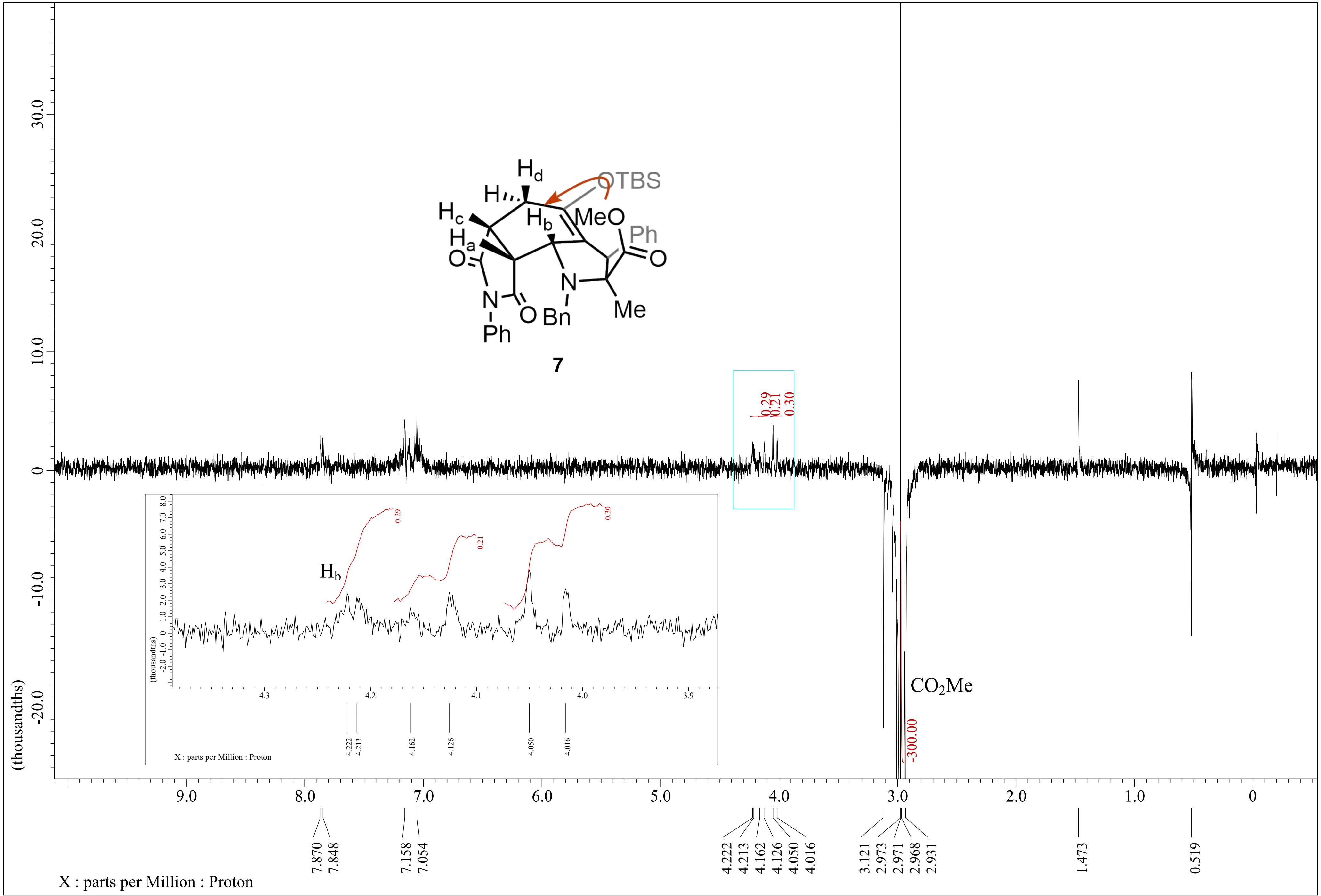
Hd
OTBS
H
Hc
Hb
MeO
Ha
Ph
O
O
N
N
O
Bn
Me
Ph
7
0.29
0.21
0.30
Hb
CO2Me
-300.00
(thousandths)
X : parts per Million : Proton
7.870
7.848
7.158
7.054
4.222
4.213
4.162
4.126
4.050
4.016
3.121
2.973
2.971
2.968
2.931
1.473
0.519

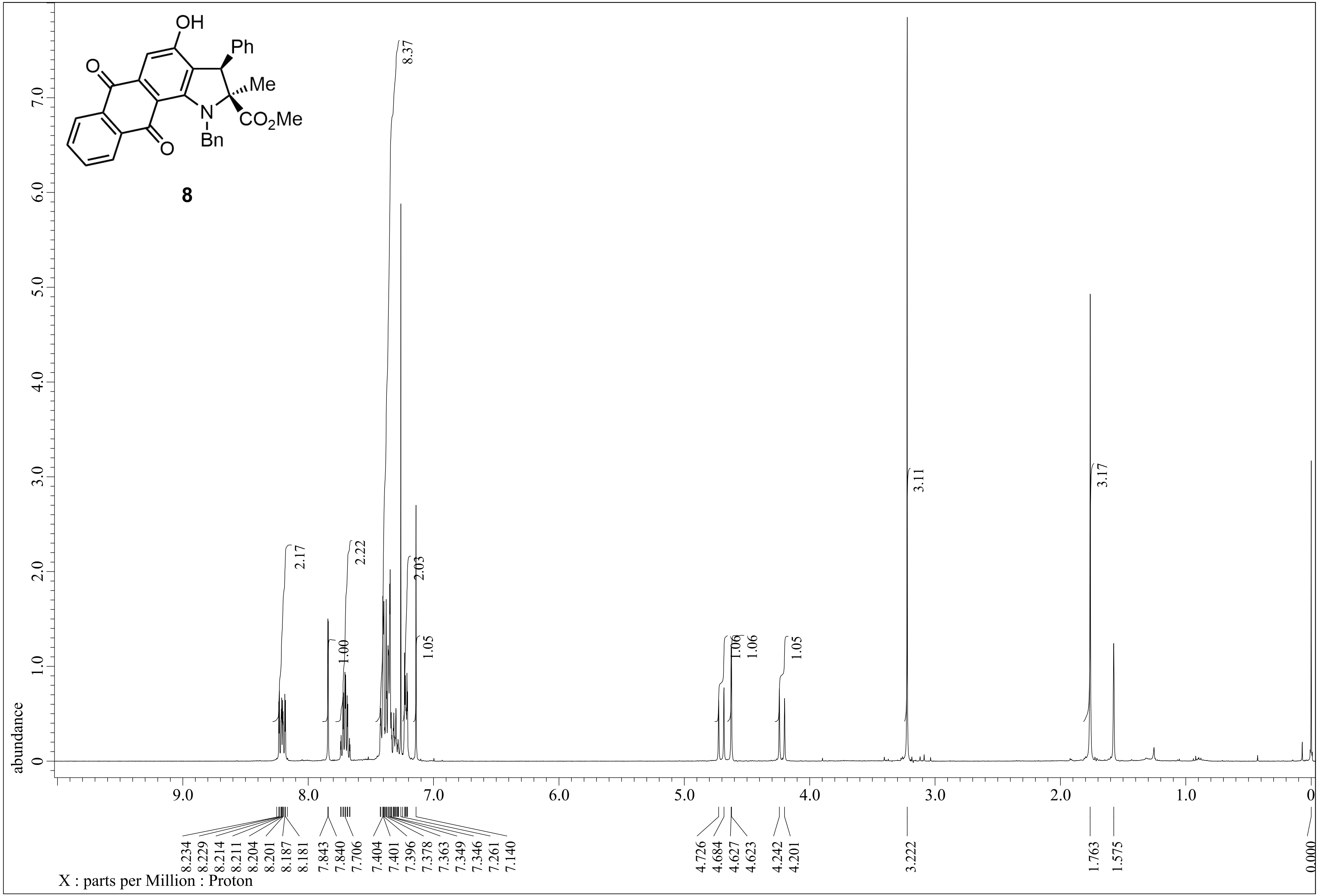
OH
Ph
O
Me
N
CO2Me
Bn
O
8
8.37
2.17
2.22
2.03
1.00
1.05
1.06
1.06
1.05
3.11
3.17
abundance
8.234
8.229
8.214
8.211
8.204
8.201
8.187
8.181
7.843
7.840
7.706
7.404
7.401
7.396
7.378
7.363
7.349
7.346
7.261
7.140
4.726
4.684
4.627
4.623
4.242
4.201
3.222
1.763
1.575
0.000
X : parts per Million : Proton

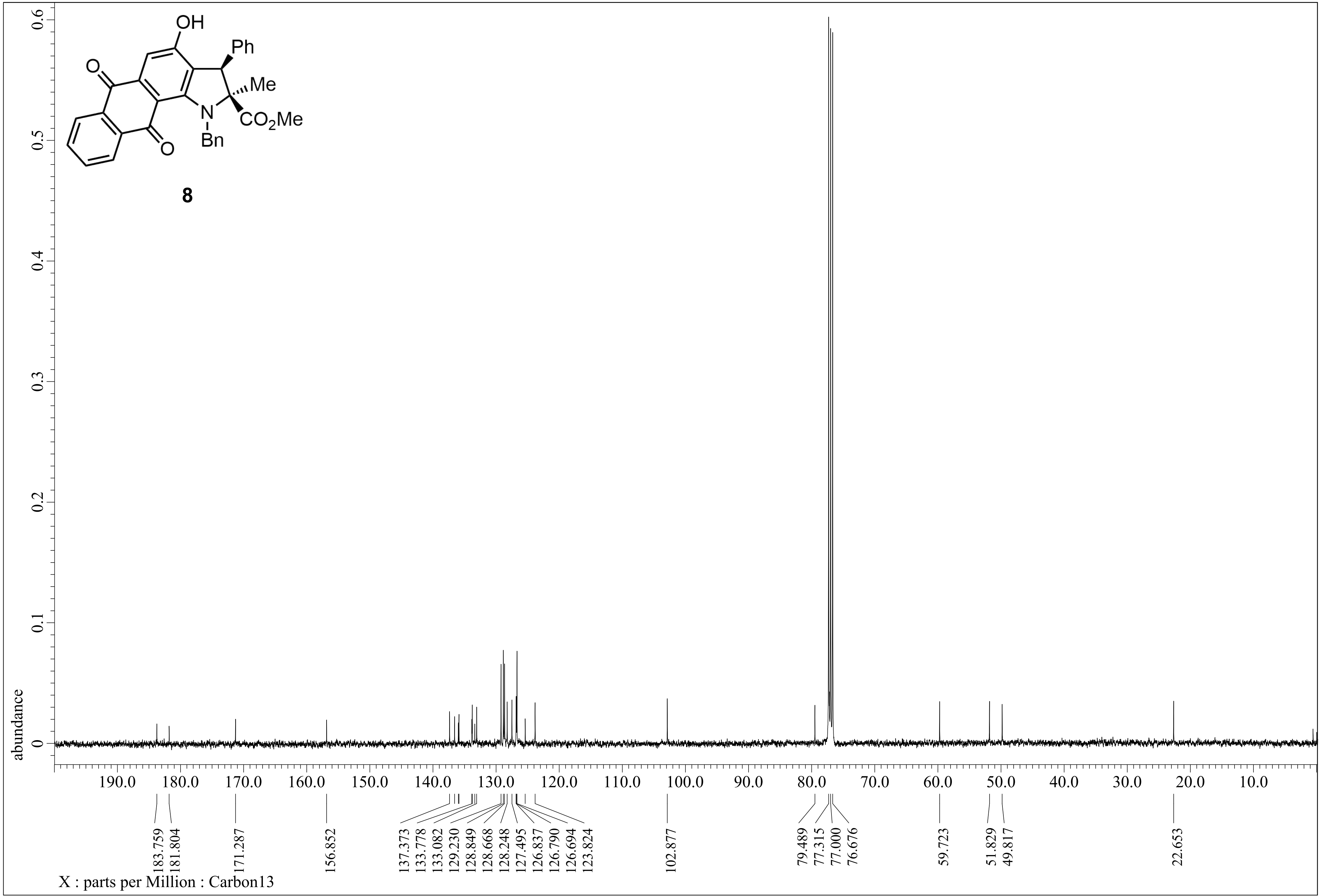
OH
Ph
O
Me
N
CO2Me
Bn
O
8
0.6
0.5
0.4
0.3
0.2
0.1
0
abundance
190.0 180.0 170.0 160.0 150.0 140.0 130.0 120.0 110.0 100.0 90.0 80.0 70.0 60.0 50.0 40.0 30.0 20.0 10.0
183.759
181.804
171.287
156.852
137.373
133.778
133.082
129.230
128.849
128.668
128.248
127.495
126.837
126.790
126.694
123.824
102.877
79.489
77.315
77.000
76.676
59.723
51.829
49.817
22.653
X : parts per Million : Carbon13

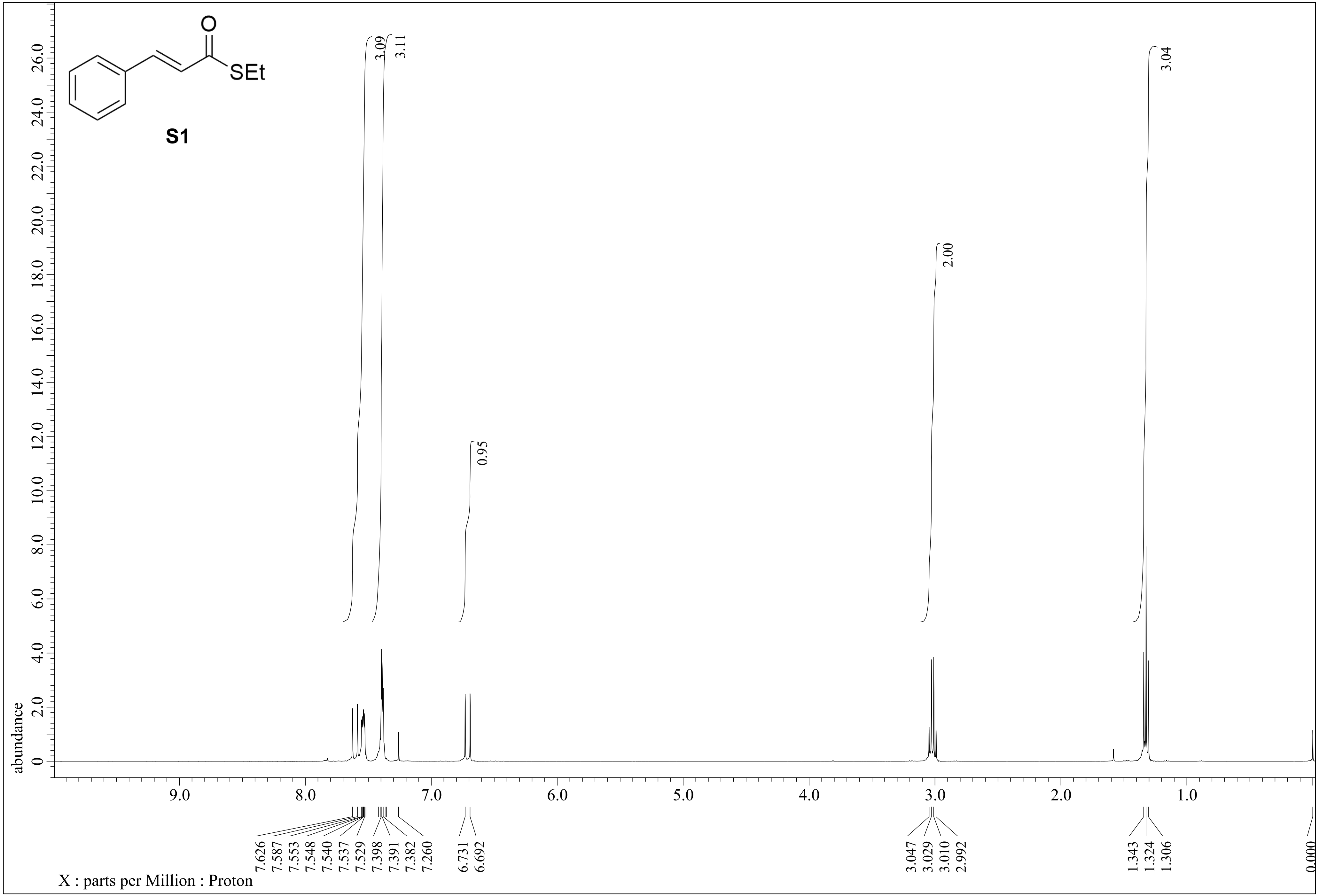

O
SEt
S1
3.09
3.11
0.95
2.00
3.04
abundance
26.0
24.0
22.0
20.0
18.0
16.0
14.0
12.0
10.0
8.0
6.0
4.0
2.0
0
9.0
8.0
7.0
6.0
5.0
4.0
3.0
2.0
1.0
7.626
7.587
7.553
7.548
7.540
7.537
7.529
7.398
7.391
7.382
7.260
6.731
6.692
3.047
3.029
3.010
2.992
1.343
1.324
1.306
0.000
X : parts per Million : Proton

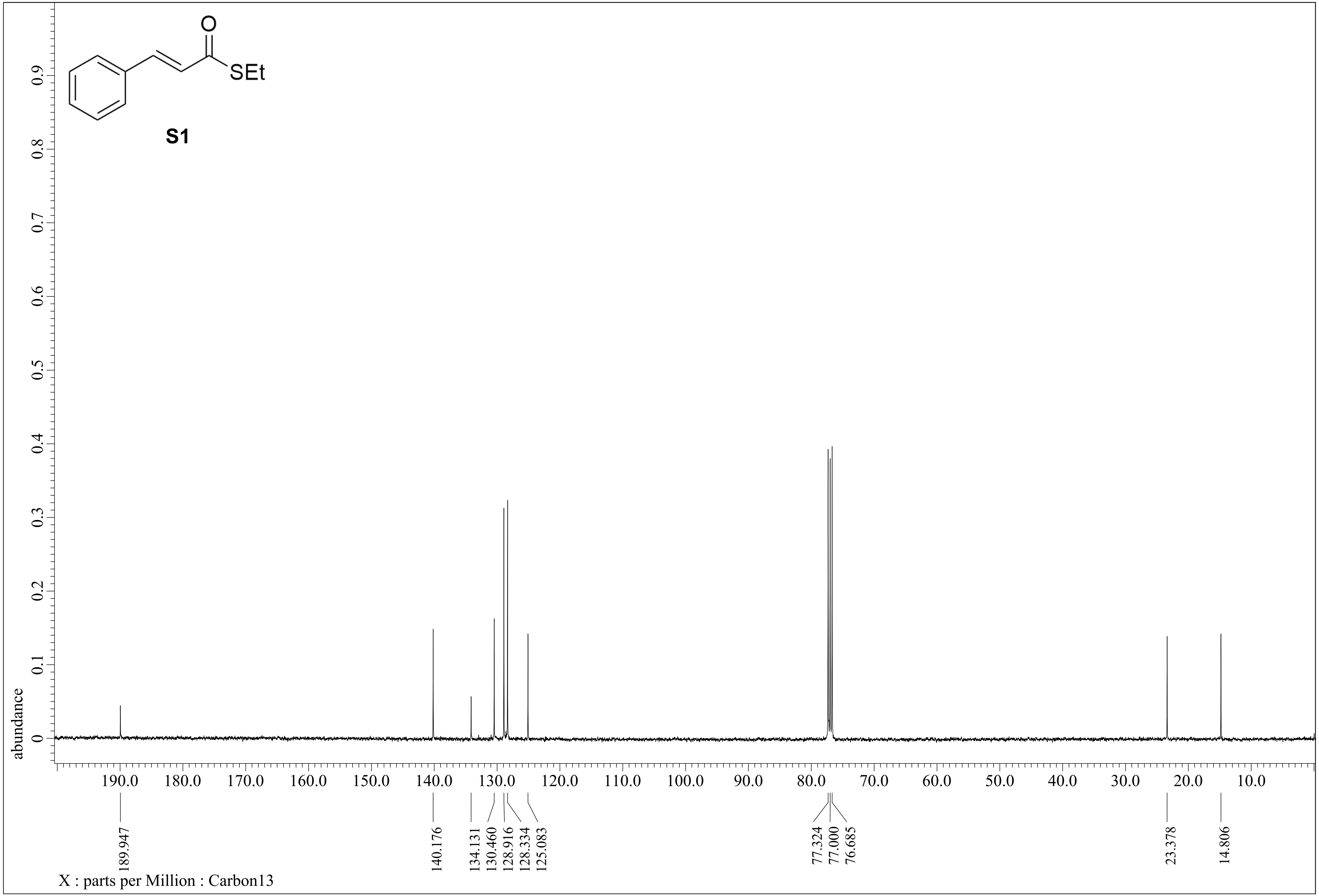

O
SEt
S1
abundance
0.9
0.8
0.7
0.6
0.5
0.4
0.3
0.2
0.1
0
190.0
180.0
170.0
160.0
150.0
140.0
130.0
120.0
110.0
100.0
90.0
80.0
70.0
60.0
50.0
40.0
30.0
20.0
10.0
189.947
140.176
134.131
130.460
128.916
128.334
125.083
77.324
77.000
76.685
23.378
14.806
X : parts per Million : Carbon13

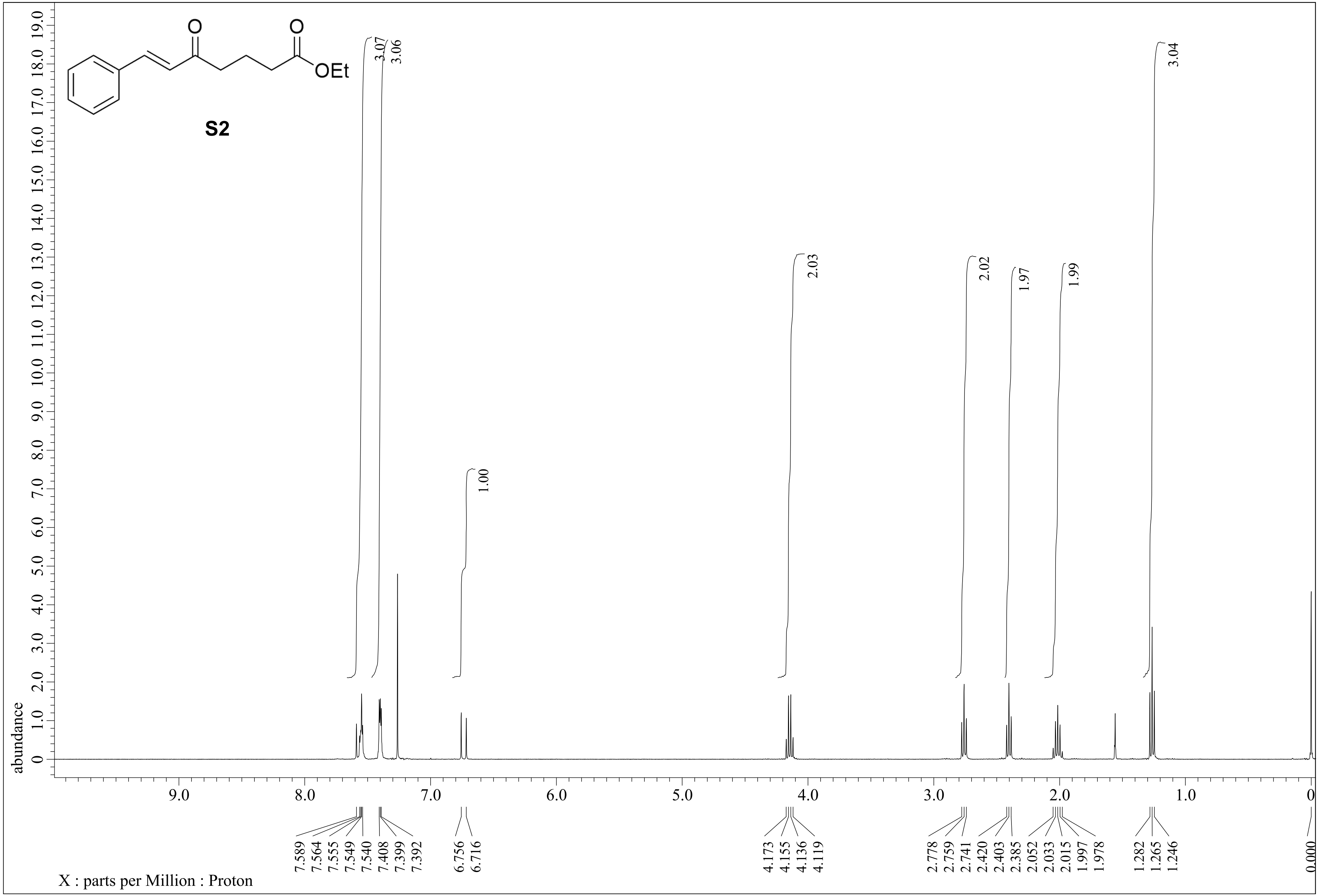
S2
OEt
abundance
X : parts per Million : Proton
3.07
3.06
1.00
2.03
2.02
1.97
1.99
3.04
7.589
7.564
7.555
7.549
7.540
7.408
7.399
7.392
6.756
6.716
4.173
4.155
4.136
4.119
2.778
2.759
2.741
2.420
2.403
2.385
2.052
2.033
2.015
1.997
1.978
1.282
1.265
1.246
0.000

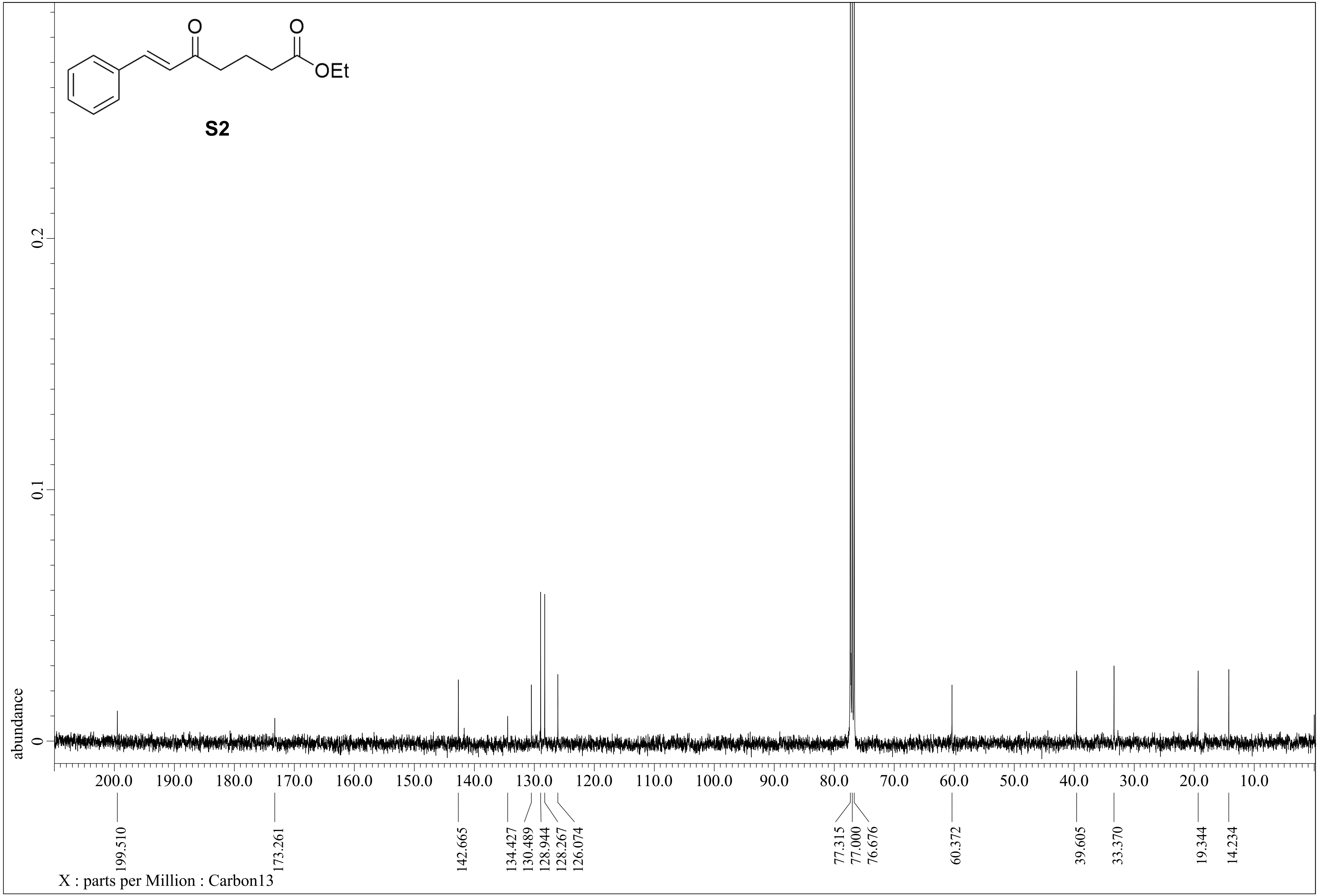
O
O
OEt
S2
0.2
0.1
0
abundance
200.0
190.0
180.0
170.0
160.0
150.0
140.0
130.0
120.0
110.0
100.0
90.0
80.0
70.0
60.0
50.0
40.0
30.0
20.0
10.0
199.510
173.261
142.665
134.427
130.489
128.944
128.267
126.074
77.315
77.000
76.676
60.372
39.605
33.370
19.344
14.234
X : parts per Million : Carbon13

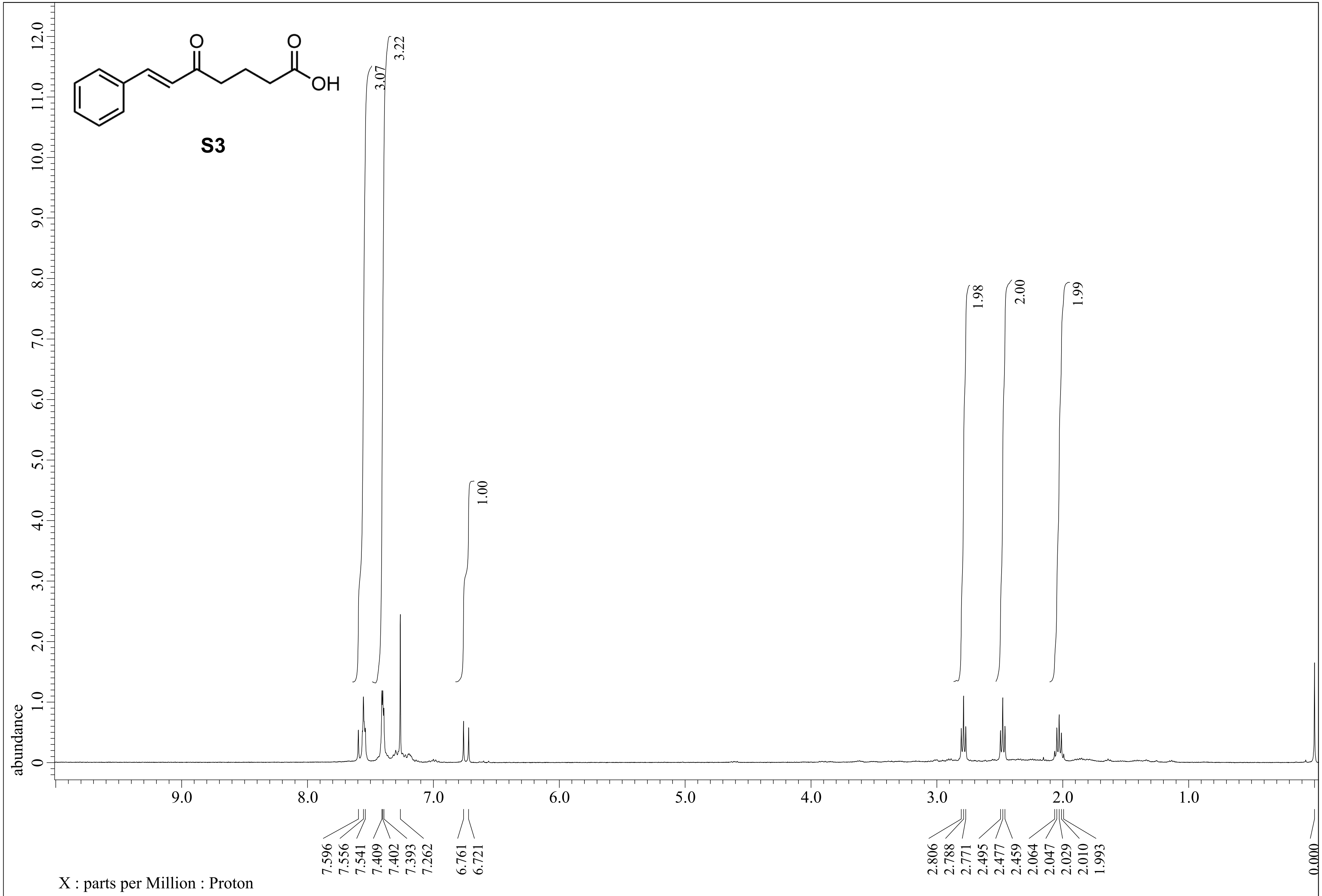
S3
OH
O
O
abundance
X : parts per Million : Proton
12.0
11.0
10.0
9.0
8.0
7.0
6.0
5.0
4.0
3.0
2.0
1.0
0
9.0
8.0
7.0
6.0
5.0
4.0
3.0
2.0
1.0
3.07
3.22
1.00
1.98
2.00
1.99
7.596
7.556
7.541
7.409
7.402
7.393
7.262
6.761
6.721
2.806
2.788
2.771
2.495
2.477
2.459
2.064
2.047
2.029
2.010
1.993
0.000

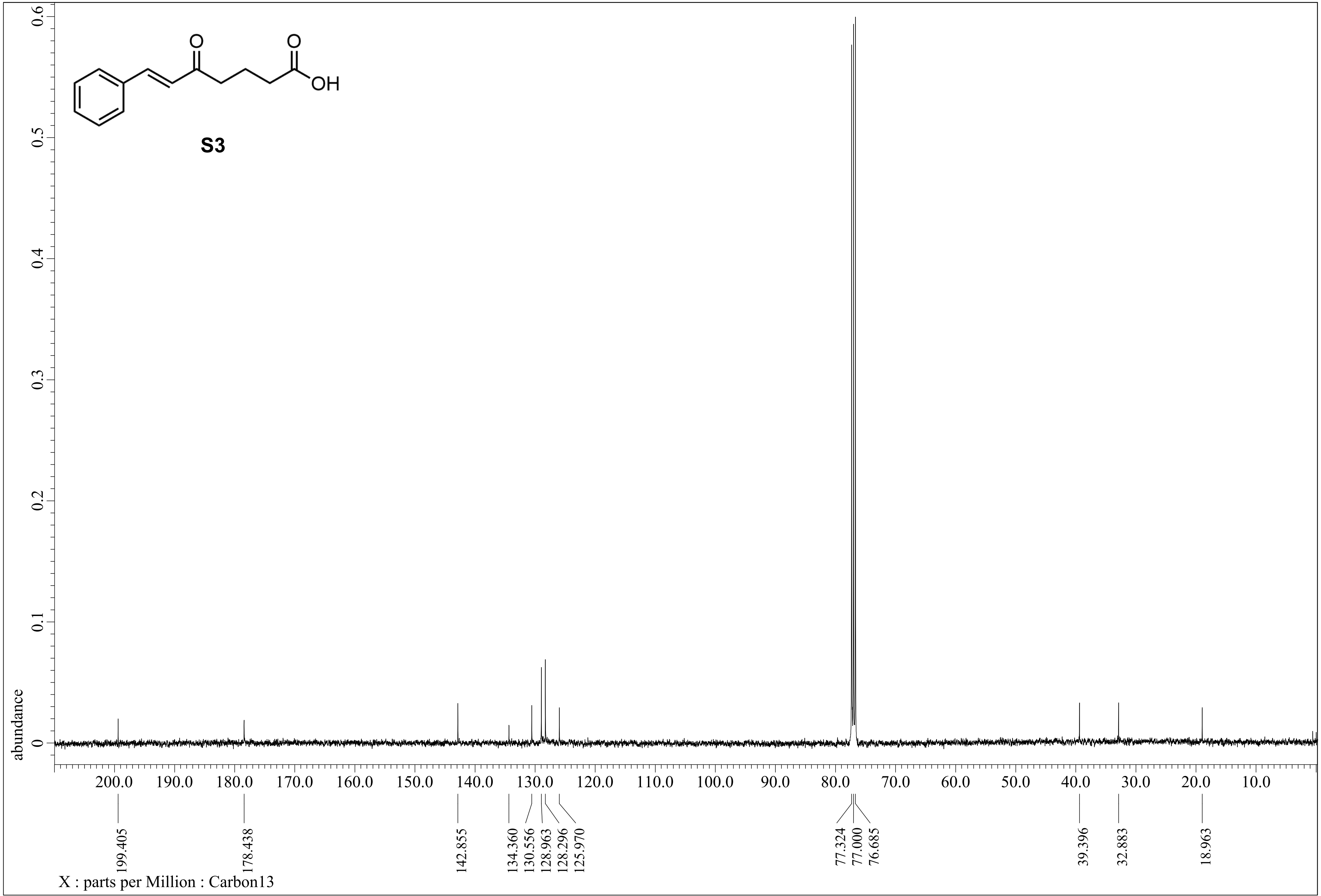
S3
OH
abundance
0.6
0.5
0.4
0.3
0.2
0.1
0
200.0 190.0 180.0 170.0 160.0 150.0 140.0 130.0 120.0 110.0 100.0 90.0 80.0 70.0 60.0 50.0 40.0 30.0 20.0 10.0
199.405
178.438
142.855
134.360
130.556
128.963
128.296
125.970
77.324
77.000
76.685
39.396
32.883
18.963
X : parts per Million : Carbon13

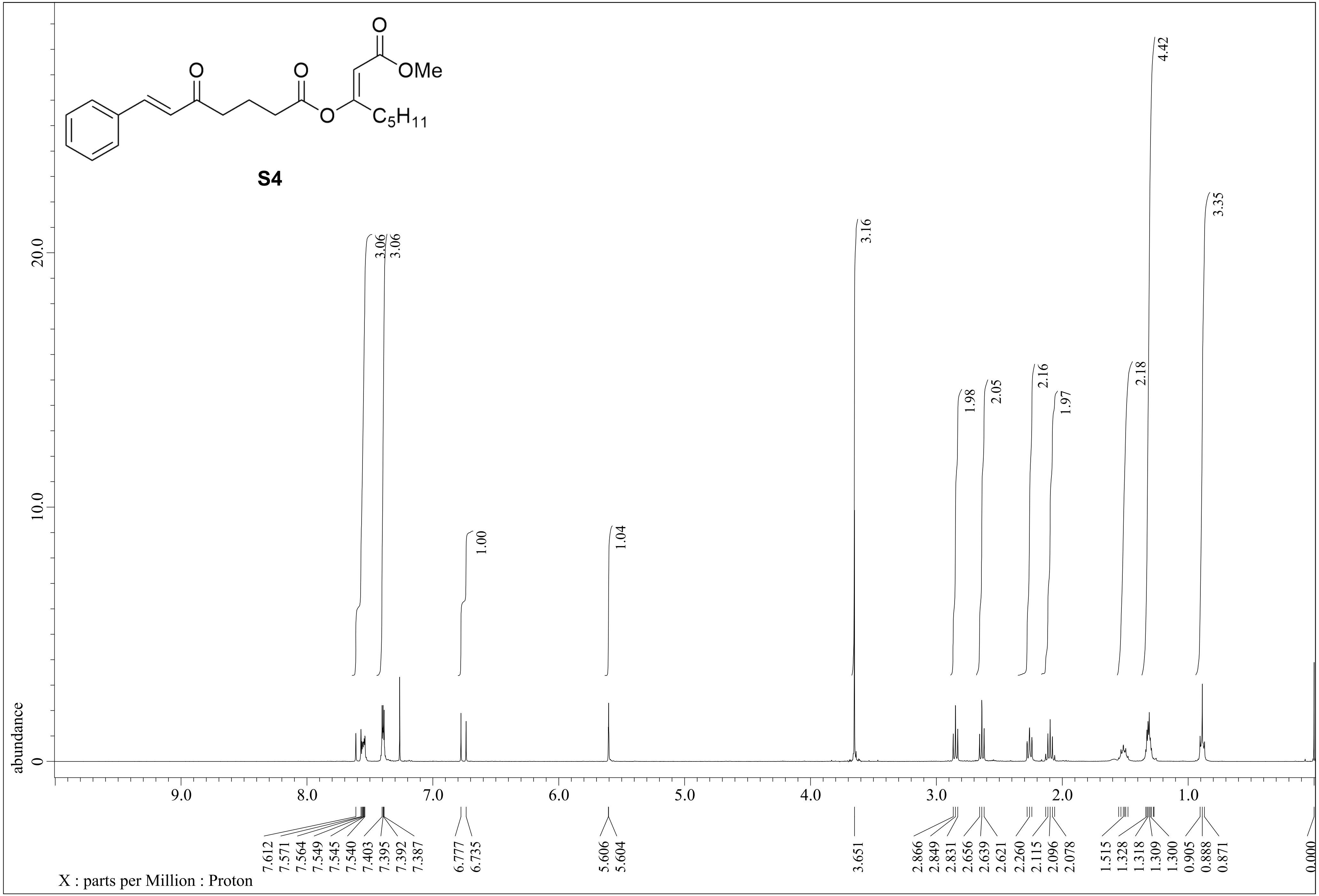

O
O
O
OMe
O
C_5H_{11}
S4
20.0
10.0
0
abundance
3.06
3.06
1.00
1.04
3.16
1.98
2.05
2.16
1.97
2.18
4.42
3.35
9.0
8.0
7.0
6.0
5.0
4.0
3.0
2.0
1.0
7.612
7.571
7.564
7.549
7.545
7.540
7.403
7.395
7.392
7.387
6.777
6.735
5.606
5.604
3.651
2.866
2.849
2.831
2.656
2.639
2.621
2.260
2.115
2.096
2.078
1.515
1.328
1.318
1.309
1.300
0.905
0.888
0.871
0.000
X : parts per Million : Proton

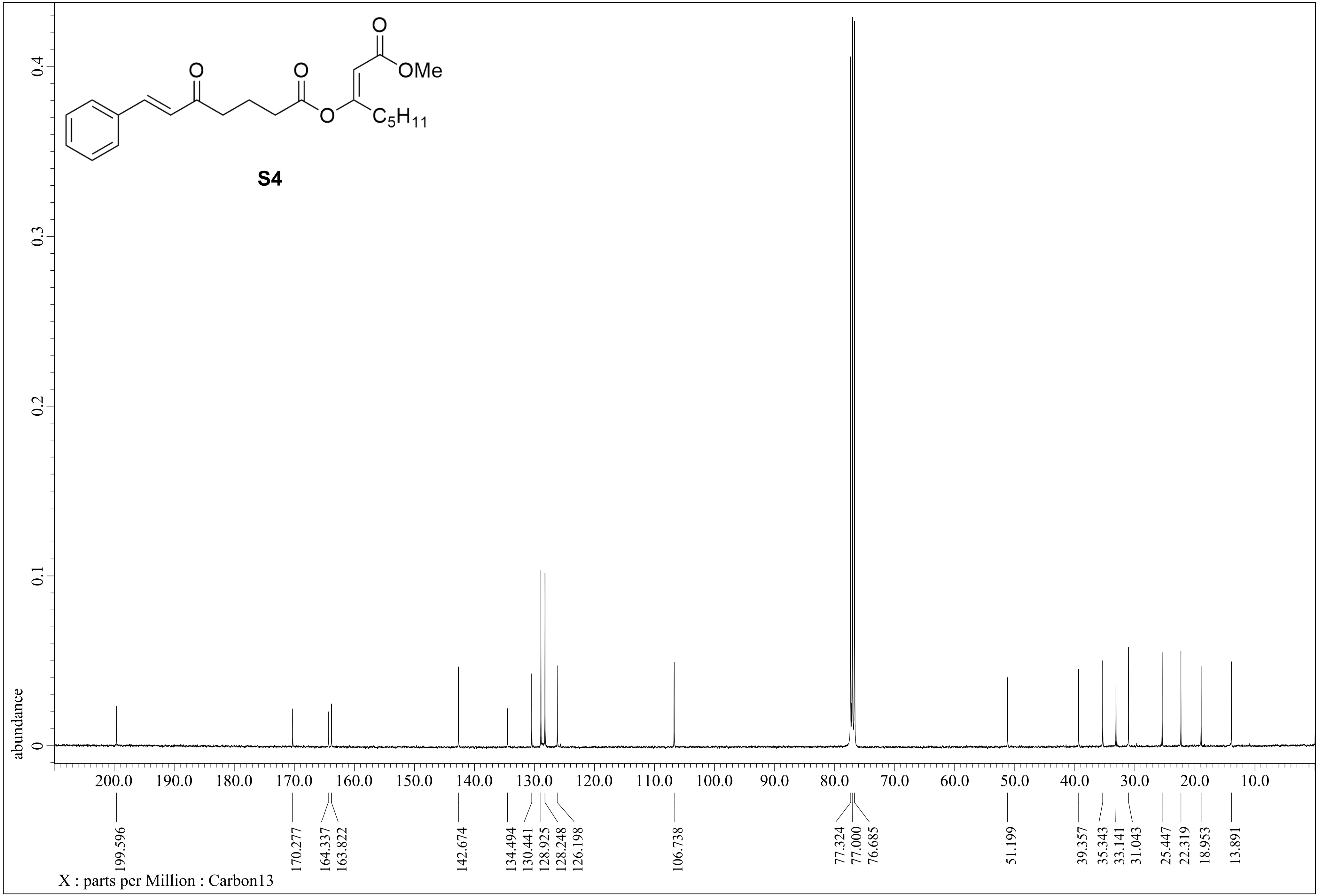
O
O
O
OMe
O
C_5H_{11}
S4
abundance
0.4
0.3
0.2
0.1
0
200.0 190.0 180.0 170.0 160.0 150.0 140.0 130.0 120.0 110.0 100.0 90.0 80.0 70.0 60.0 50.0 40.0 30.0 20.0 10.0
199.596
170.277
164.337
163.822
142.674
134.494
130.441
128.925
128.248
126.198
106.738
77.324
77.000
76.685
51.199
39.357
35.343
33.141
31.043
25.447
22.319
18.953
13.891
X : parts per Million : Carbon13

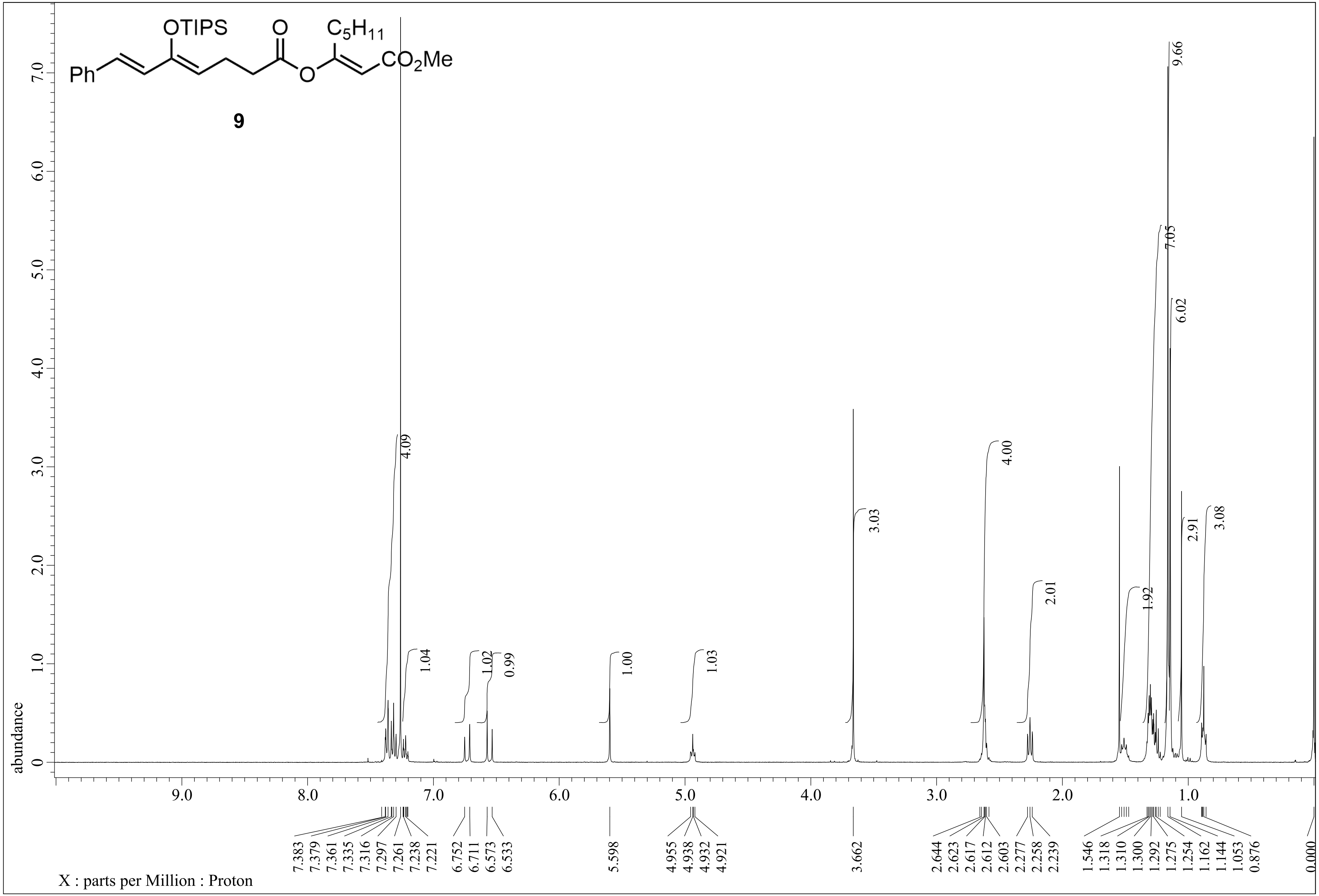
OTIPS
O
C5H11
Ph
O
CO2Me
9
abundance
X : parts per Million : Proton
4.09
1.04
1.02
0.99
1.00
1.03
3.03
4.00
2.01
1.92
7.05
6.02
9.66
2.91
3.08
7.383
7.379
7.361
7.335
7.316
7.297
7.261
7.238
7.221
6.752
6.711
6.573
6.533
5.598
4.955
4.938
4.932
4.921
3.662
2.644
2.623
2.617
2.612
2.603
2.277
2.258
2.239
1.546
1.318
1.310
1.300
1.292
1.275
1.254
1.162
1.144
1.053
0.876
0.000

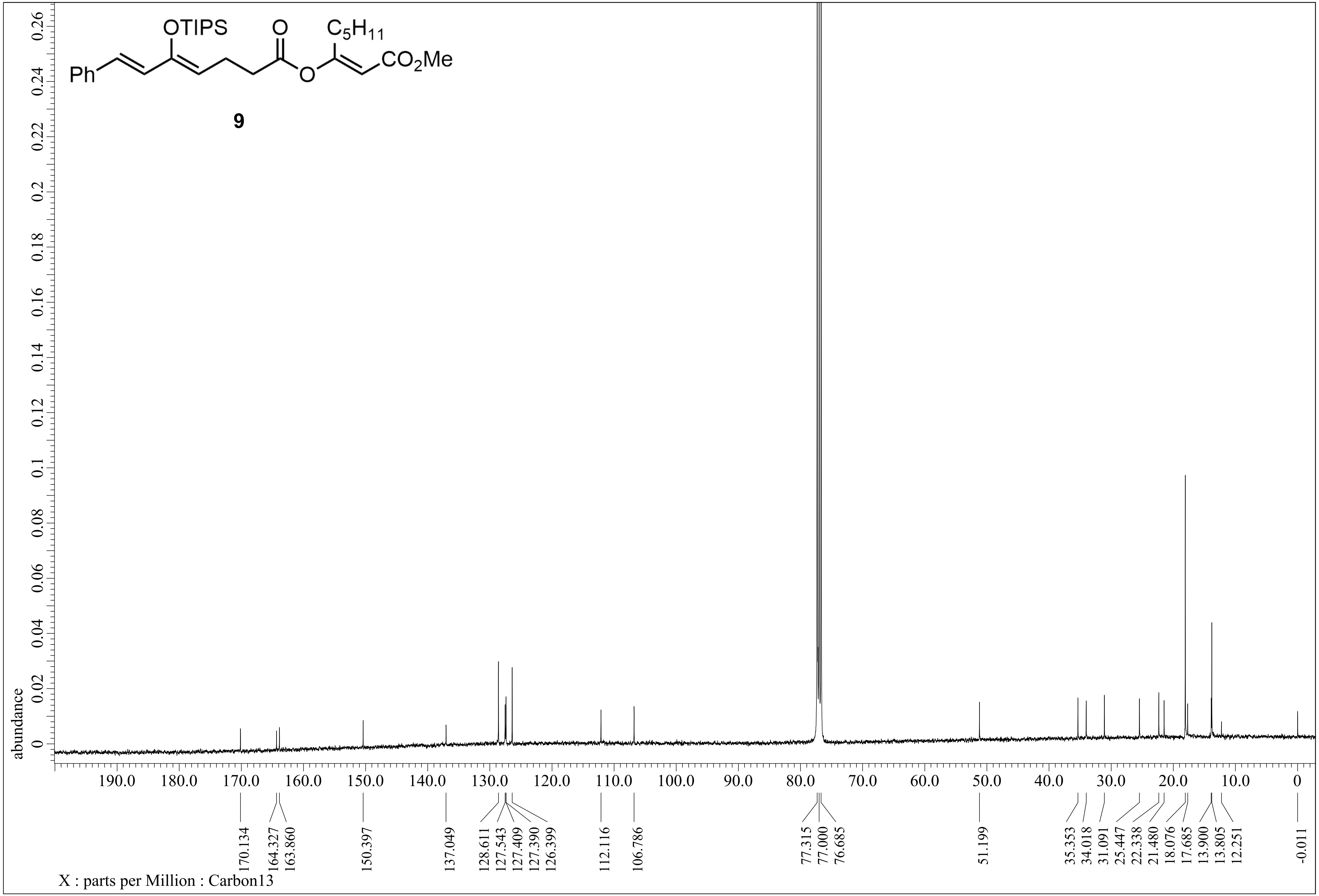
OTIPS
O
C5H11
Ph
O
CO2Me
9
abundance
X : parts per Million : Carbon13
170.134
164.327
163.860
150.397
137.049
128.611
127.543
127.409
127.390
126.399
112.116
106.786
77.315
77.000
76.685
51.199
35.353
34.018
31.091
25.447
22.338
21.480
18.076
17.685
13.900
13.805
12.251
-0.011

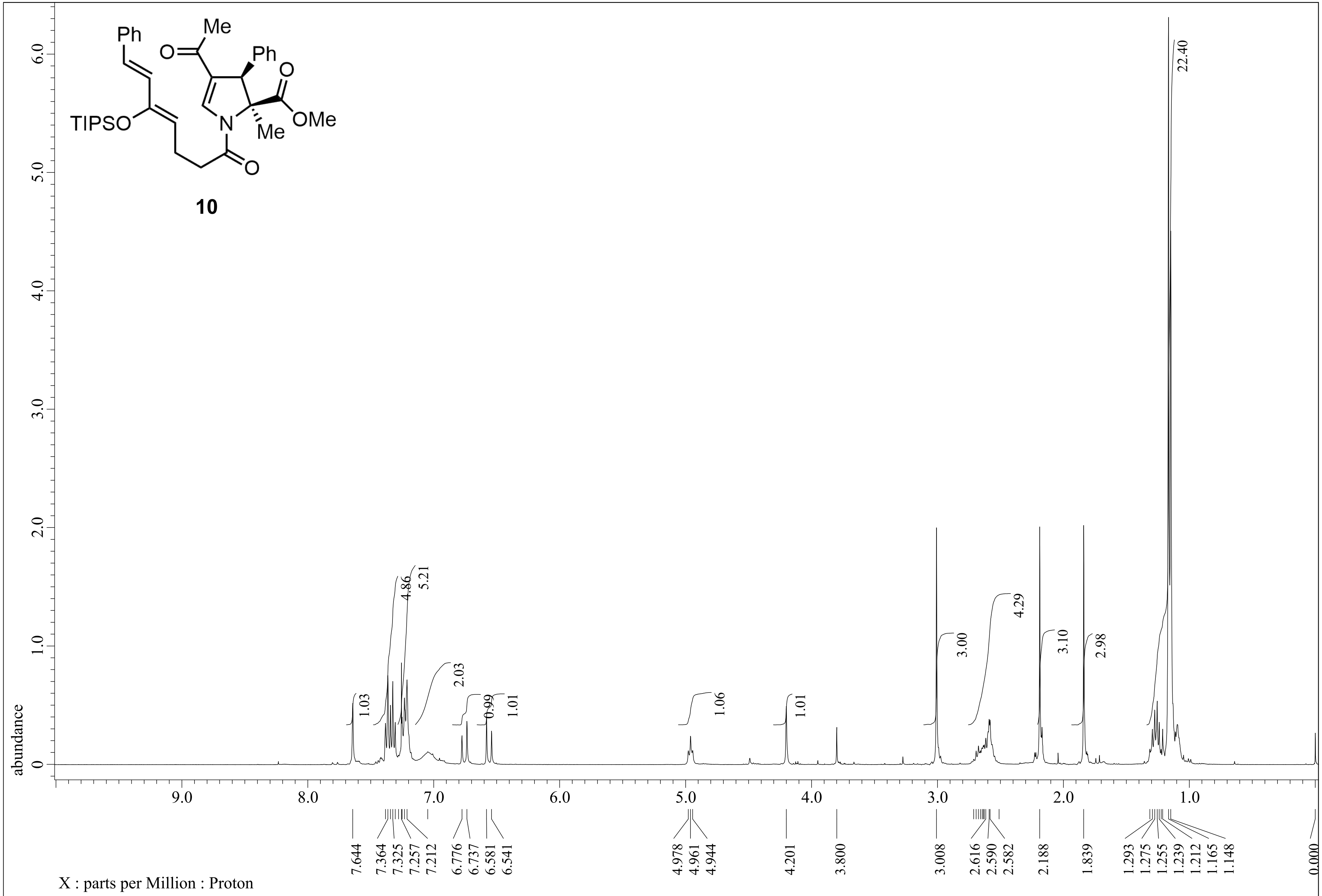

Ph
Me
O
Ph
O
TIPSO
N
Me
OMe
O
10
abundance
X : parts per Million : Proton

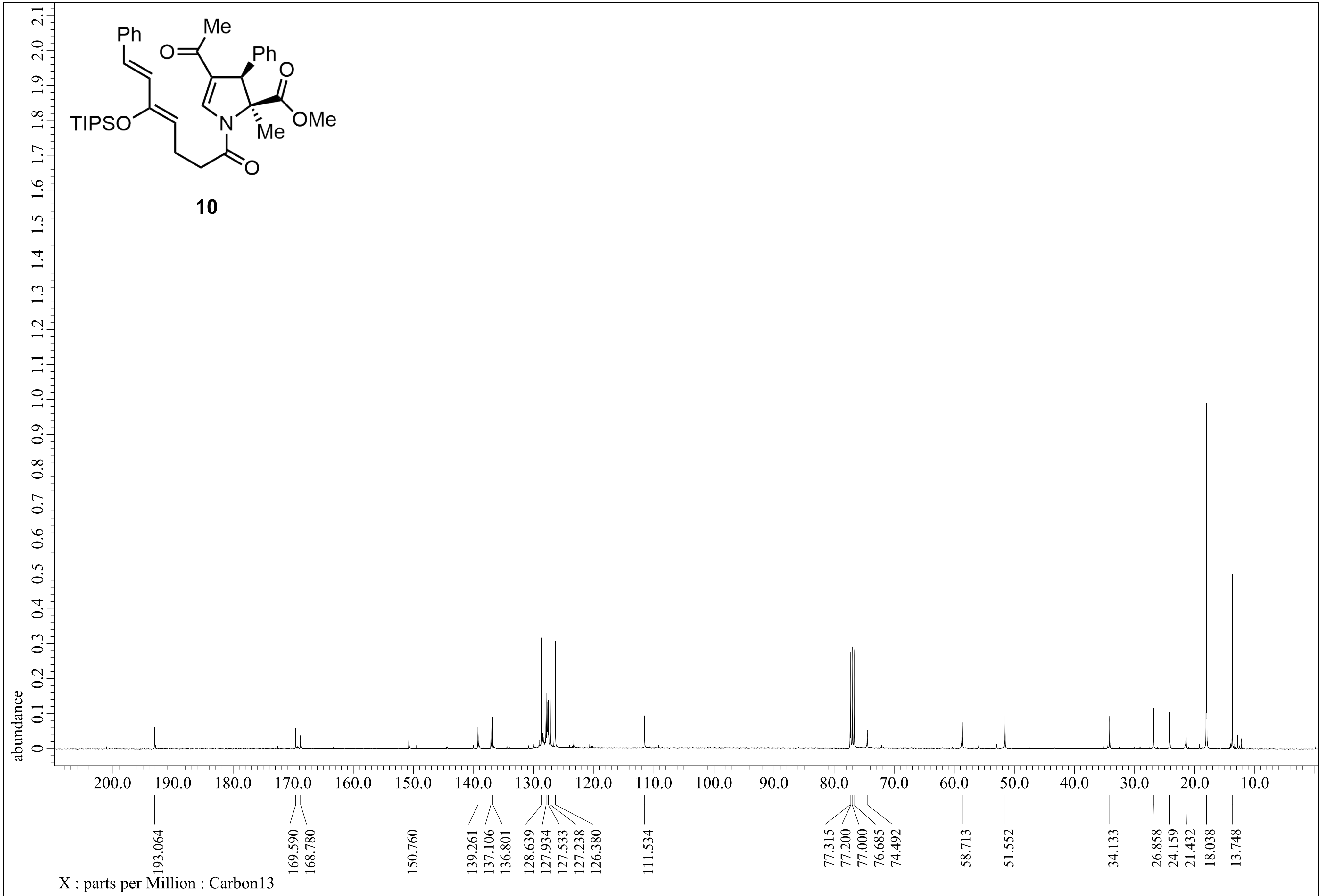

Ph
Me
O
Ph
O
TIPSO
N
Me
OMe
O
10
abundance
0 0.1 0.2 0.3 0.4 0.5 0.6 0.7 0.8 0.9 1.0 1.1 1.2 1.3 1.4 1.5 1.6 1.7 1.8 1.9 2.0 2.1
200.0 190.0 180.0 170.0 160.0 150.0 140.0 130.0 120.0 110.0 100.0 90.0 80.0 70.0 60.0 50.0 40.0 30.0 20.0 10.0
193.064
169.590
168.780
150.760
139.261
137.106
136.801
128.639
127.934
127.533
127.238
126.380
111.534
77.315
77.200
77.000
76.685
74.492
58.713
51.552
34.133
26.858
24.159
21.432
18.038
13.748
X : parts per Million : Carbon13

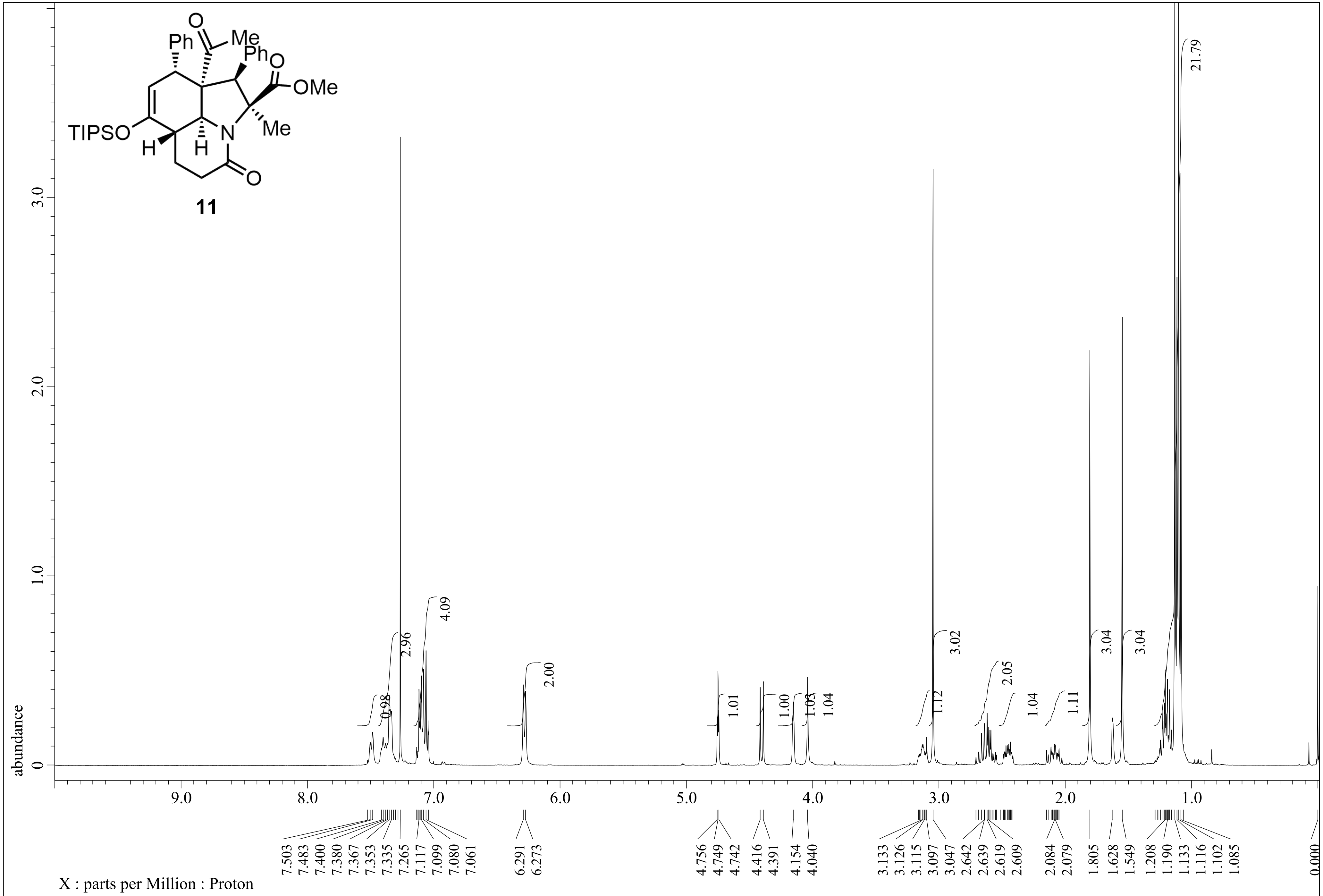
Ph
O
Me
Ph
O
OMe
TIPSO
N
Me
H
H
O
11
abundance
3.0
2.0
1.0
0
9.0
8.0
7.0
6.0
5.0
4.0
3.0
2.0
1.0
X : parts per Million : Proton
0.98
2.96
4.09
2.00
1.01
1.00
1.03
1.04
1.12
3.02
2.05
1.04
1.11
3.04
3.04
21.79
7.503
7.483
7.400
7.380
7.367
7.353
7.335
7.265
7.117
7.099
7.080
7.061
6.291
6.273
4.756
4.749
4.742
4.416
4.391
4.154
4.040
3.133
3.126
3.115
3.097
3.047
2.642
2.639
2.619
2.609
2.084
2.079
1.805
1.628
1.549
1.208
1.190
1.133
1.116
1.102
1.085
0.000

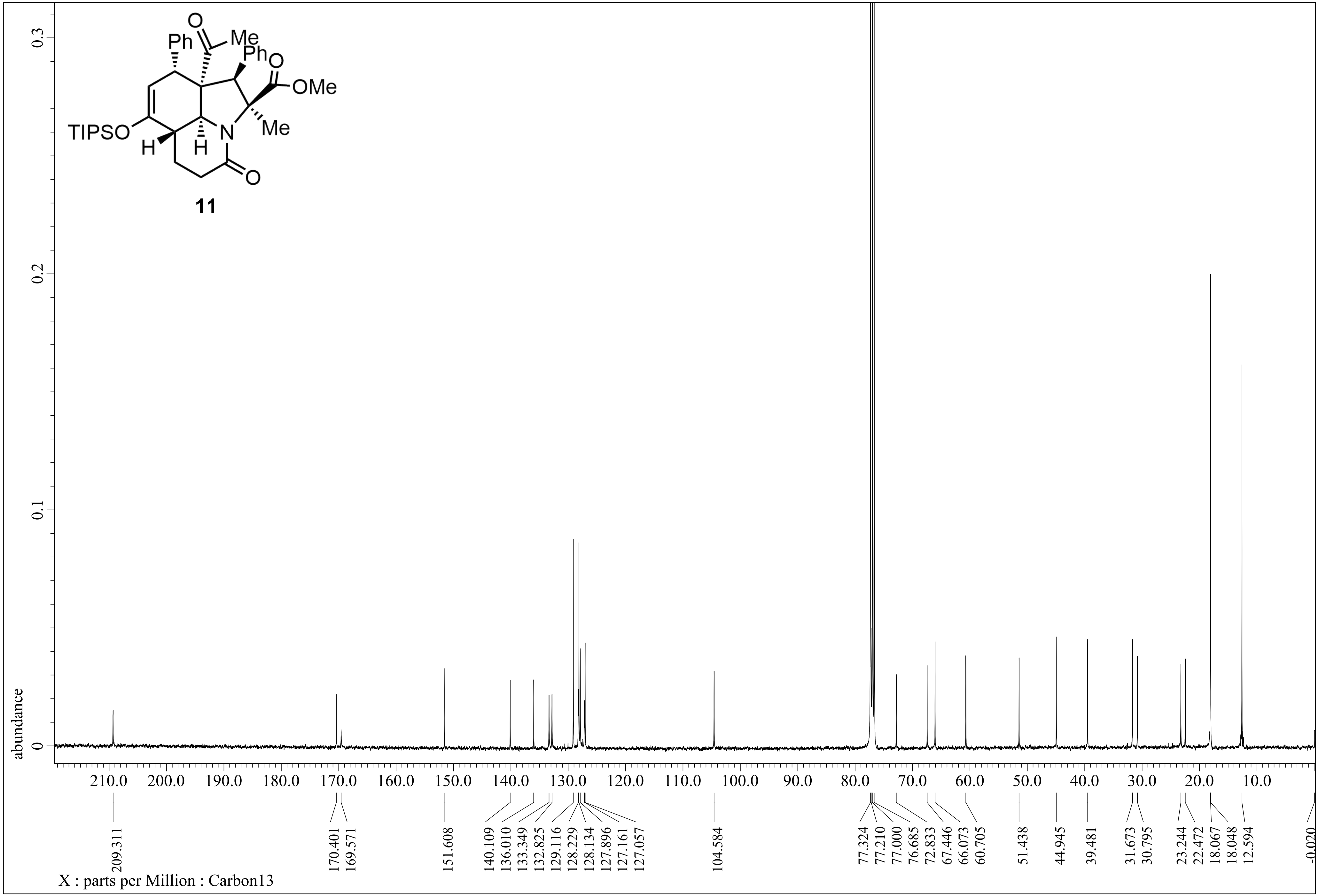
Ph
O
Me
Ph
O
OMe
TIPSO
H
H
N
Me
O
11
0.3
0.2
0.1
0
abundance
210.0 200.0 190.0 180.0 170.0 160.0 150.0 140.0 130.0 120.0 110.0 100.0 90.0 80.0 70.0 60.0 50.0 40.0 30.0 20.0 10.0
209.311
170.401
169.571
151.608
140.109
136.010
133.349
132.825
129.116
128.229
128.134
127.896
127.161
127.057
104.584
77.324
77.210
77.000
76.685
72.833
67.446
66.073
60.705
51.438
44.945
39.481
31.673
30.795
23.244
22.472
18.067
18.048
12.594
-0.020
X : parts per Million : Carbon13

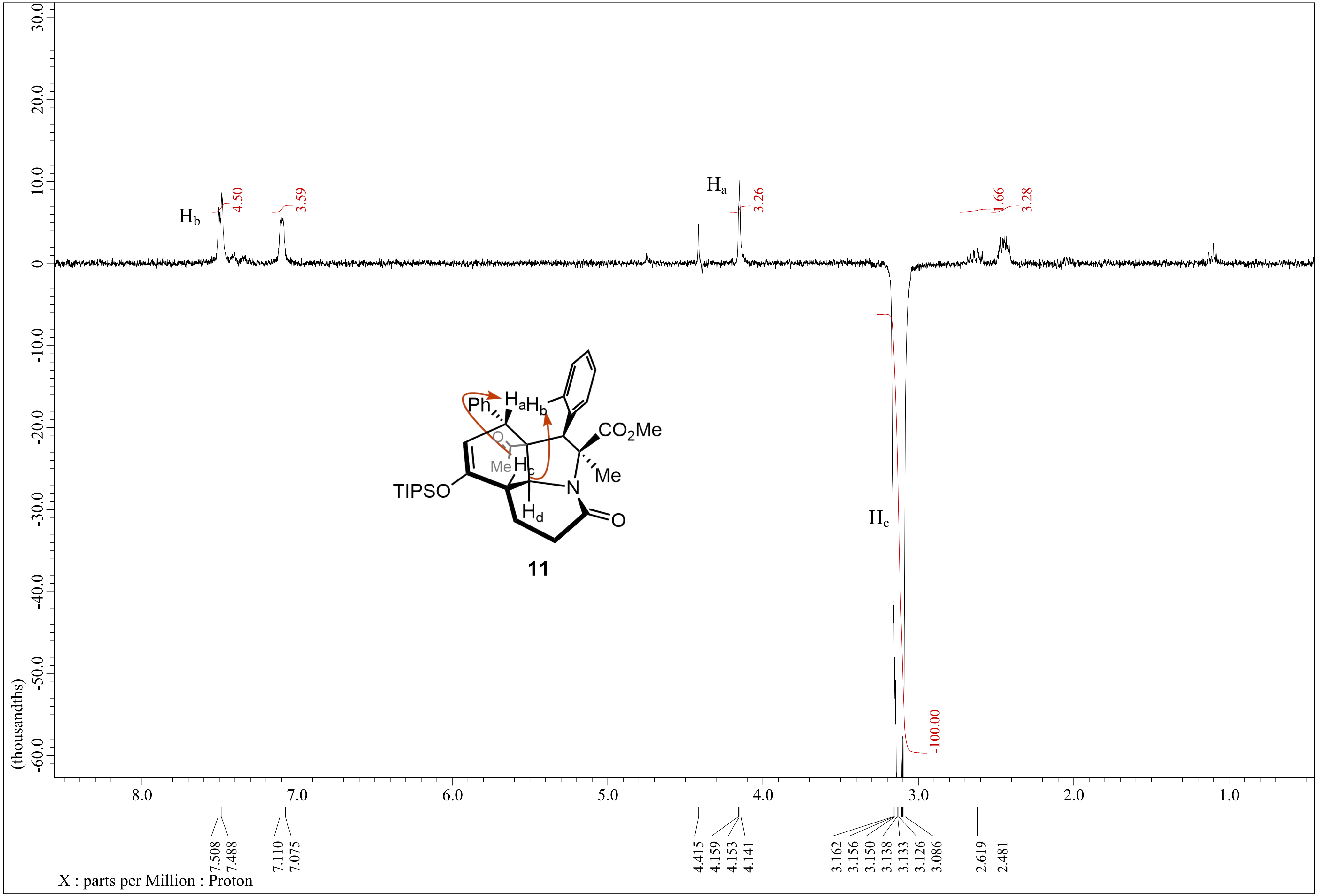

H
b
4.50
3.59
H
a
3.26
1.66
3.28
H
c
-100.00
Ph
H
a
H
b
CO
2
Me
O
Me
H
c
Me
TIPSO
N
H
d
O
11
(thousandths)
X : parts per Million : Proton
7.508
7.488
7.110
7.075
4.415
4.159
4.153
4.141
3.162
3.156
3.150
3.138
3.133
3.126
3.086
2.619
2.481

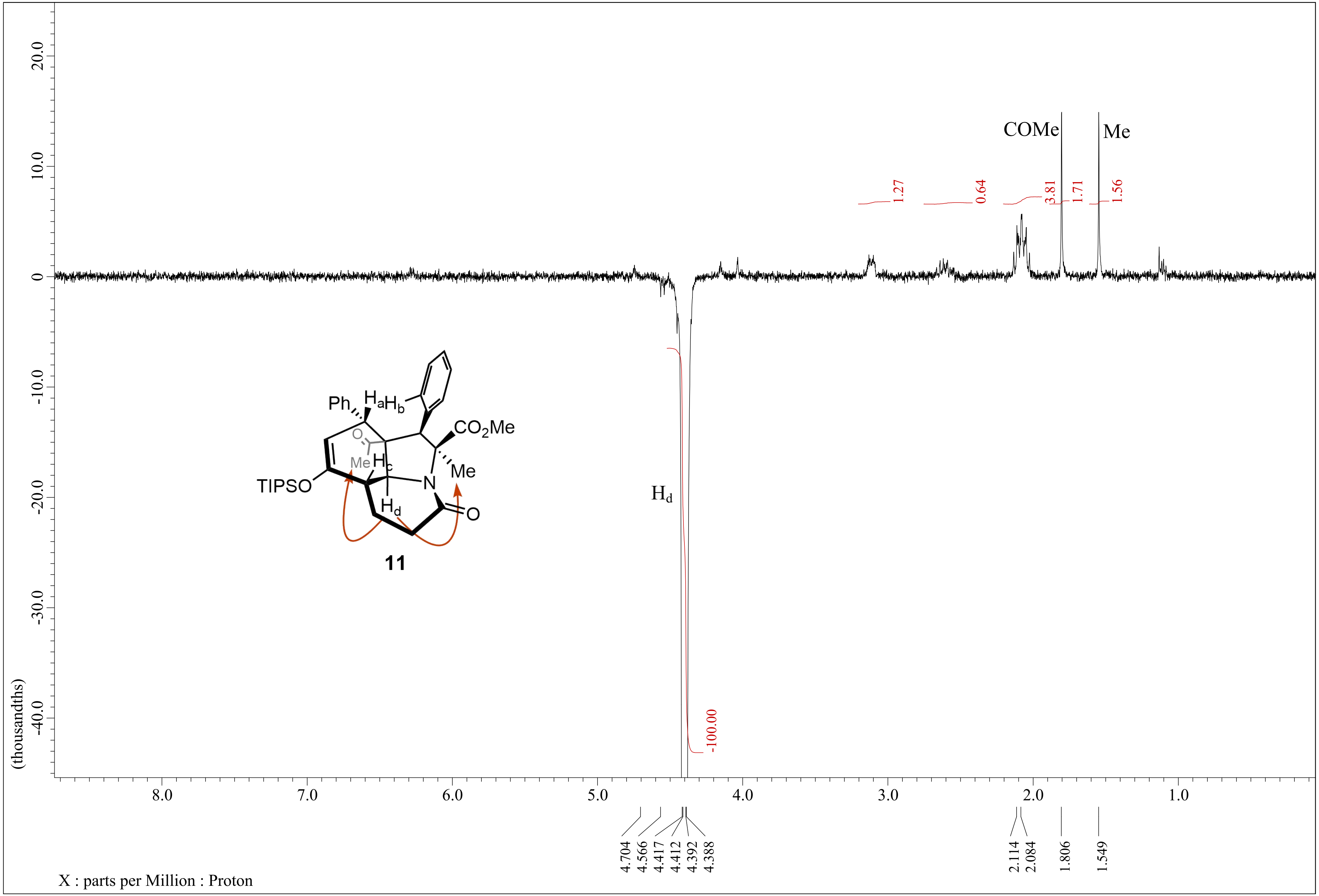
COMe
Me
1.27
0.64
3.81
1.71
1.56
-100.00
H$_d$
Ph
H$_a$H$_b$
CO$_2$Me
Me
TIPSO
H$_c$
H$_d$
N
O
11
20.0
10.0
0
-10.0
-20.0
-30.0
-40.0
(thousandths)
8.0
7.0
6.0
5.0
4.0
3.0
2.0
1.0
4.704
4.566
4.417
4.412
4.392
4.388
2.114
2.084
1.806
1.549
X : parts per Million : Proton

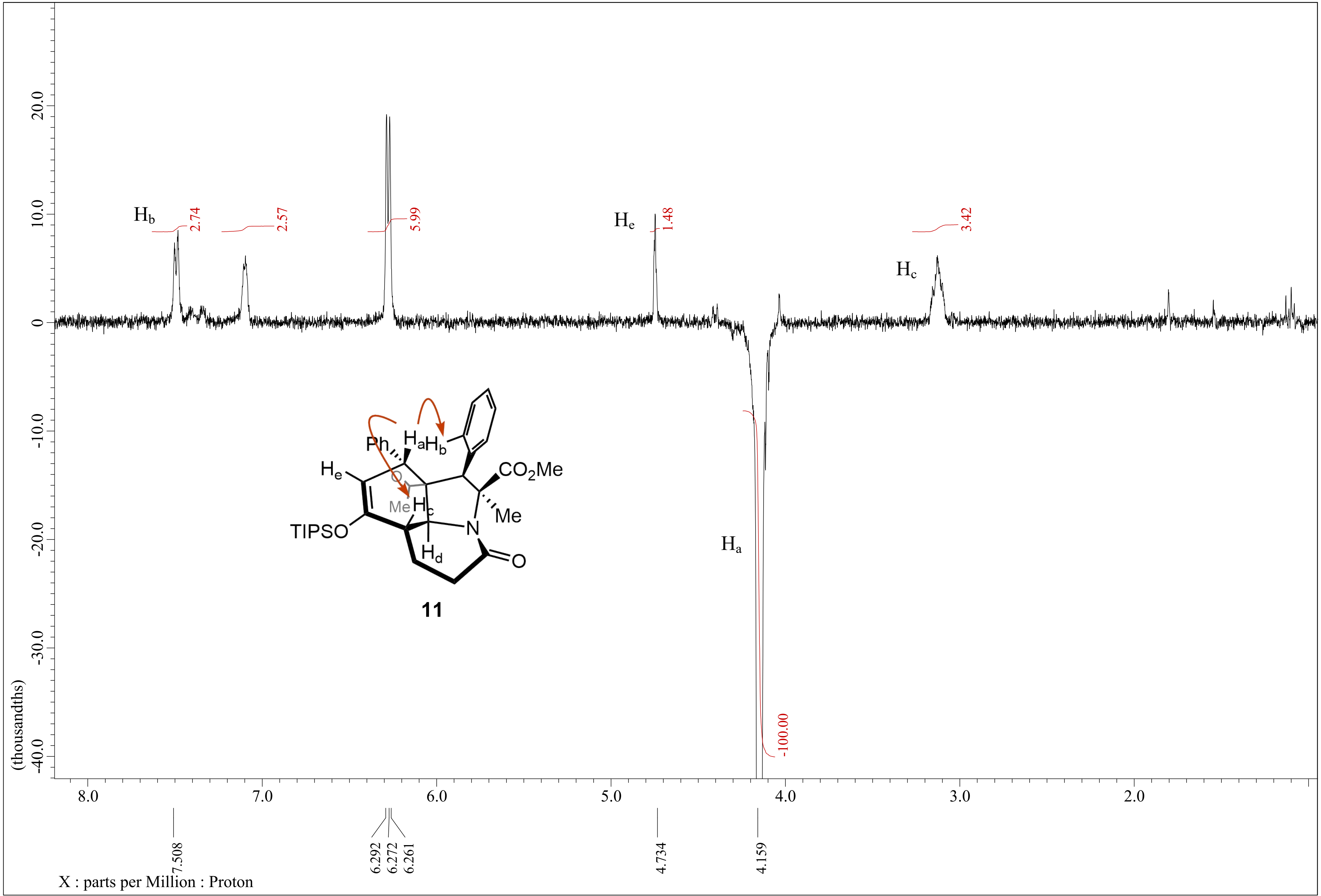

Hb
2.74
2.57
5.99
He
1.48
3.42
Hc
Ha
-100.00
Ph
HaHb
CO2Me
He
O
Me
Hc
Me
TIPSO
N
Hd
O
11
20.0
10.0
0
-10.0
-20.0
-30.0
-40.0
(thousandths)
8.0
7.0
6.0
5.0
4.0
3.0
2.0
7.508
6.292
6.272
6.261
4.734
4.159
X : parts per Million : Proton

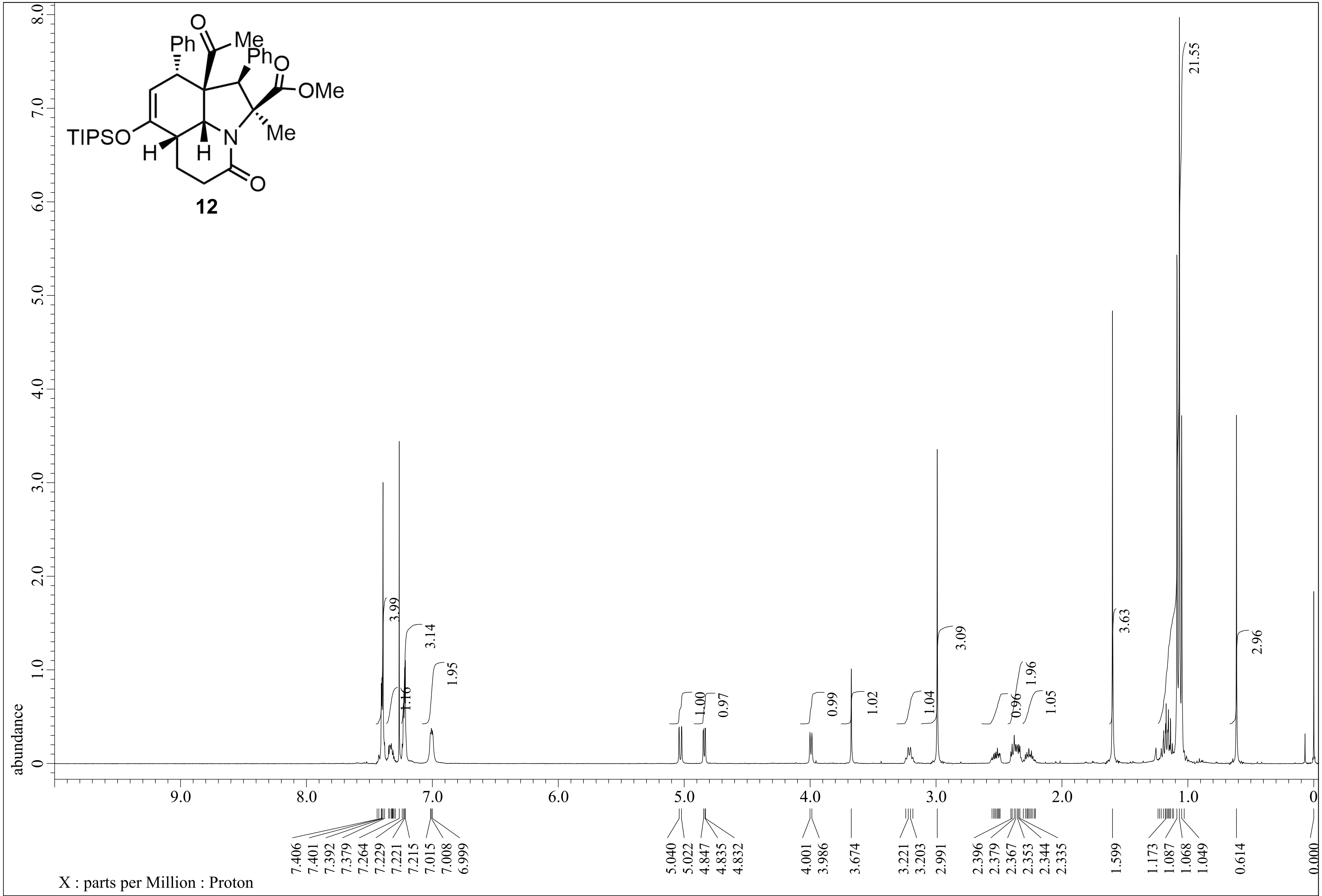
TIPSO
Ph
Me
Ph
OMe
Me
N
H
H
12
abundance
X : parts per Million : Proton
3.99
1.16
3.14
1.95
1.00
0.97
0.99
1.02
1.04
3.09
0.96
1.96
1.05
3.63
21.55
2.96
7.406
7.401
7.392
7.379
7.264
7.229
7.221
7.215
7.015
7.008
6.999
5.040
5.022
4.847
4.835
4.832
4.001
3.986
3.674
3.221
3.203
2.991
2.396
2.379
2.367
2.353
2.344
2.335
1.599
1.173
1.087
1.068
1.049
0.614
0.000

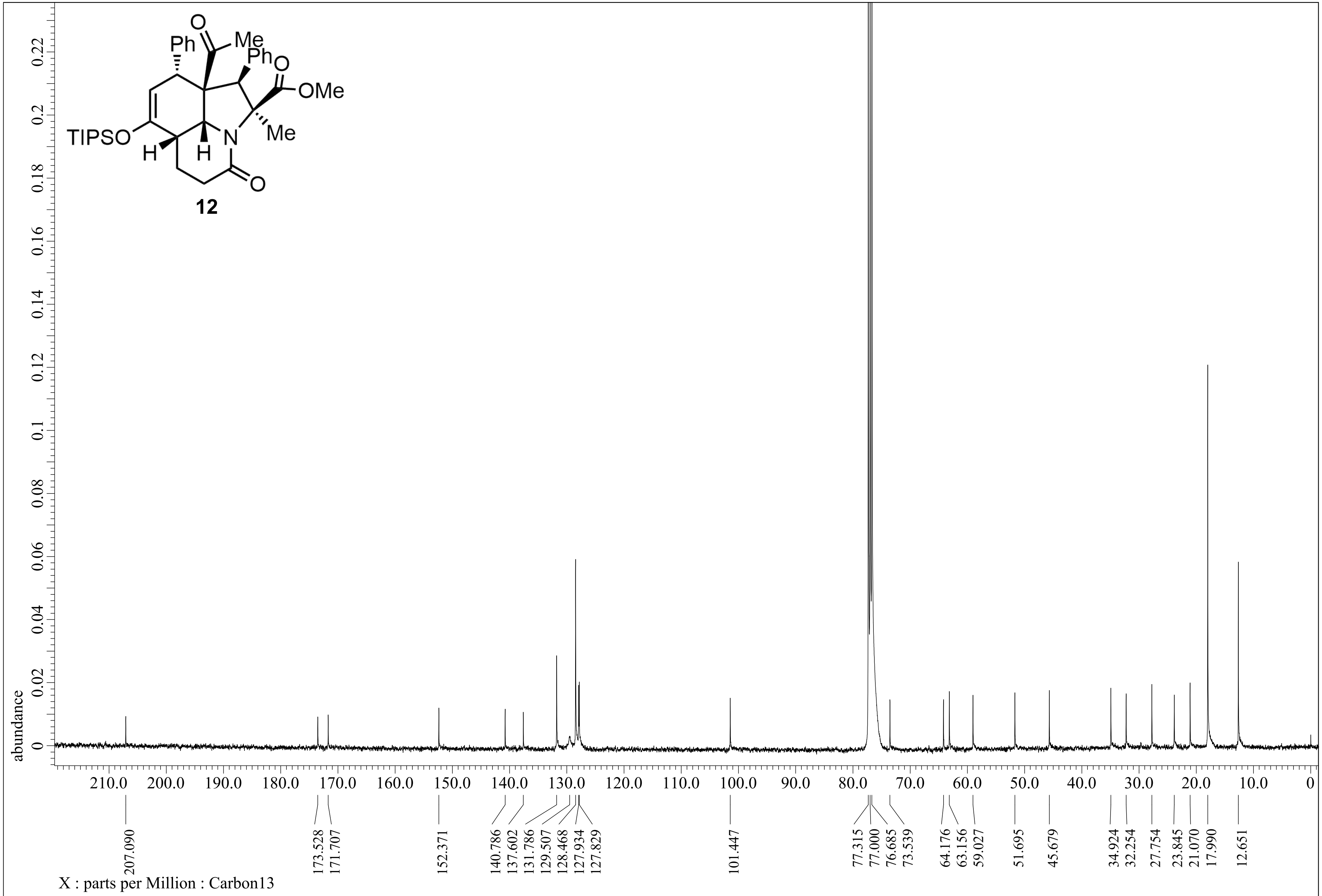
TIPSO
Ph
O
Me
Ph
O
OMe
Me
H
H
N
O
12
abundance
0.22
0.2
0.18
0.16
0.14
0.12
0.1
0.08
0.06
0.04
0.02
0
210.0 200.0 190.0 180.0 170.0 160.0 150.0 140.0 130.0 120.0 110.0 100.0 90.0 80.0 70.0 60.0 50.0 40.0 30.0 20.0 10.0 0
207.090
173.528
171.707
152.371
140.786
137.602
131.786
129.507
128.468
127.934
127.829
101.447
77.315
77.000
76.685
73.539
64.176
63.156
59.027
51.695
45.679
34.924
32.254
27.754
23.845
21.070
17.990
12.651
X : parts per Million : Carbon13

2D NOESY

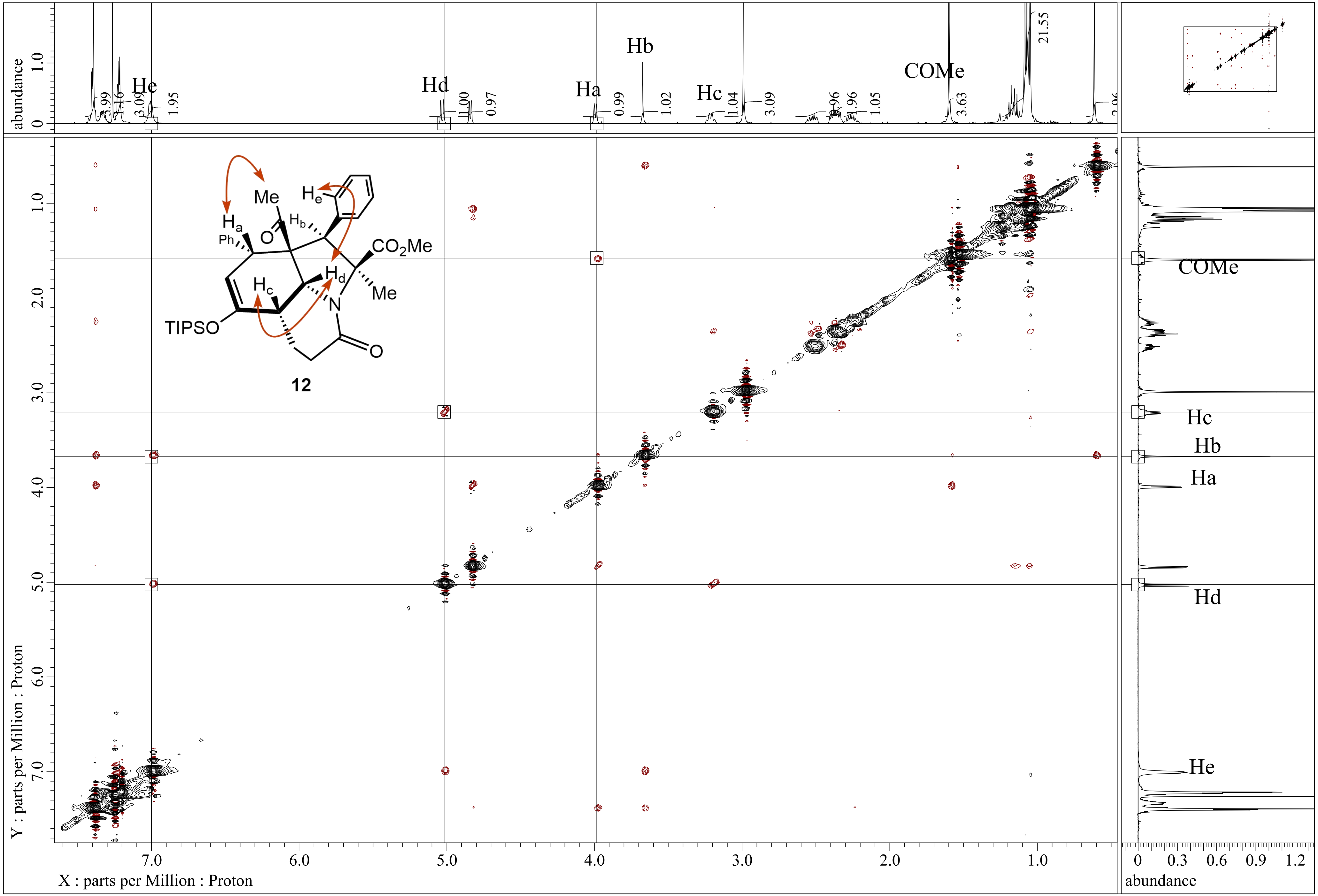

He
Hd
Ha
Hb
Hc
COMe
21.55
abundance
Me
Ha
Ph
O
Hb
He
CO2Me
Hd
Hc
Me
N
TIPSO
O
12
COMe
Hc
Hb
Ha
Hd
He
Y : parts per Million : Proton
X : parts per Million : Proton
abundance
